# Total Synthesis of a Mycolic Acid from *Mycobacterium tuberculosis*

**DOI:** 10.1002/anie.202000523

**Published:** 2020-03-10

**Authors:** Nabil Tahiri, Peter Fodran, Dhineshkumar Jayaraman, Jeffrey Buter, Martin D. Witte, Tonatiuh A. Ocampo, D. Branch Moody, Ildiko Van Rhijn, Adriaan J. Minnaard

**Affiliations:** ^1^ Stratingh Institute for Chemistry University of Groningen Nijenborgh 7 9747 AG Groningen The Netherlands; ^2^ Brigham and Women's Hospital, Division of Rheumatology, Inflammation, and Immunity Harvard Medical School, Hale BTM 60 Fenwood Road Boston MA 02115 USA; ^3^ Department of Infectious Diseases and Immunology Faculty of Veterinary Medicine Utrecht University Yalelaan 1 3584 CL Utrecht The Netherlands

**Keywords:** CD1b, cross coupling, mycolic acid, total synthesis, tuberculosis

## Abstract

In *Mycobacterium tuberculosis,* mycolic acids and their glycerol, glucose, and trehalose esters (“cord factor”) form the main part of the mycomembrane. Despite their first isolation almost a century ago, full stereochemical evaluation is lacking, as is a scalable synthesis required for accurate immunological, including vaccination, studies. Herein, we report an efficient, convergent, gram‐scale synthesis of four stereo‐isomers of a mycolic acid and its glucose ester. Binding to the antigen presenting protein CD1b and T cell activation studies are used to confirm the antigenicity of the synthetic material. The absolute stereochemistry of the *syn*‐methoxy methyl moiety in natural material is evaluated by comparing its optical rotation with that of synthetic material.

## Graphical abstract

**The total synthesis** of four methoxy mycolic acid diastereomers is described. Key steps involve a Suzuki–Fu cross‐coupling, an *anti*‐selective Abiko–Masamune asymmetric aldol reaction, and an enantioselective Charette cyclopropanation. CD1b‐free mycolic acid tetramer staining experiments clearly favoured one diastereomer over the other three.

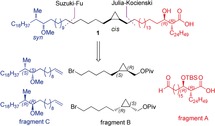

## Introduction

*Mycobacterium tuberculosis* (*Mtb)*, the causative agent of tuberculosis, is by far the most lethal member of the family of Mycobacteriaceae, causing around 10 million new infections yearly.^1^ The difficult diagnosis and treatment of tuberculosis is reflected in high mortality rates (1.5 million in 2018), which makes it the leading cause of death by a single infectious agent worldwide.^1^ This difficult treatment is in part caused by the presence of the mycomembrane in the cell envelope of *Mtb*, which consists largely of α‐alkyl, β‐hydroxy long‐chain (C70–C90) fatty acids, known as mycolic acids.^2^, ^3^ These highly lipophilic fatty acids are categorized according to the presence of unsaturations or cyclopropyl groups (“α‐mycolic acid”), a methoxy, or a keto function in the main chain. As the number of methylene units between these substituents is variable, mycolic acids are found as an inseparable mixture of homologues. Mycolic acids are found either as the free acids or esterified to the arabinogalactan layer, trehalose, glucose (glucose monomycolate; GMM) or glycerol.

**Figure 1:**
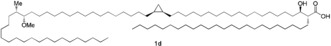
The structure of the major methoxy mycolic acid homologue.

**Figure 2:**
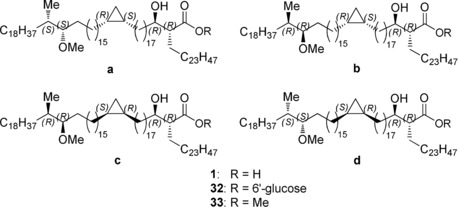
Overview of the four diastereomeric mycolic acids (**1 a**–**d**), glucose mycolates (**32 a**–**d**), and methyl esters (**33 a**–**d**).

The overall molecular structure of mycolic acids has been elucidated in the late 1960s,^7^, ^8^, ^9^ and several members have been prepared by the group of Baird^10^, ^11^, ^12^, ^13^ including two methoxy mycolic acid diastereomers (Figure 2, **1 c** and **1 d**). The complete elucidation of their stereochemistry, however, has so far not been achieved. Moody and co‐workers demonstrated that *R,R* stereochemistry of the α‐alkyl, β‐hydroxy carboxylic acid is crucial for T cell recognition of glucose monomycolate.^14^ The stereochemistry of the α‐methyl methoxy moiety in methoxy mycolic acids was inferred by Asselineau et al. to be *S,S* by comparison of molar optical rotations of natural samples with those of reference compounds.^7^ For this, the authors applied the principle of optical superposition, originally hypothesized by Van′t Hoff.^15^ This hypothesis states that stereocenters that are remotely located do not influence each other's optical rotation, which therefore can be added up to provide the overall optical rotation. The analysis was supported by the synthesis work of Baird et al., but it remained uncertain whether this hypothesis was valid for mycolic acids as the optical rotation of the *cis*‐cyclopropyl ring in model systems was immeasurably small, making the determination of its absolute stereochemistry using this strategy impossible. Due to the lack of other viable analytical techniques, the stereochemistry of the cyclopropyl ring still remains to be determined.

Methoxy mycolic acids (Figure 1) appear, with some exceptions, to be present only in pathogenic mycobacteria^3^ and are antigenic in serological assays^4^ and T cell assays.^5^, ^6^ Upon binding to the antigen‐presenting protein CD1b, mycolic acids and their glucose mycolates activate T cells.^16^, ^17^ For glucose monomycolates the type and length of the mycolate does not influence the T cell stimulatory capacity.^17^ Crystallography of the trimolecular complex of CD1b, glucose monomycolate and T cell receptor showed that the glucose forms the T cell epitope on top of the CD1b protein and stabilizes the complex.^18^ No crystallographic data of CD1b–mycolic acid are available, but it is known that the type of functional groups in the chain and its length determine the level of T cell activation.^5^, ^6^ We hypothesized that the stereochemistry of the cyclopropyl and the α‐methyl methoxy moiety of mycolic acid might influence its capacity to bind CD1b and activate T cells.

**Scheme 1:**
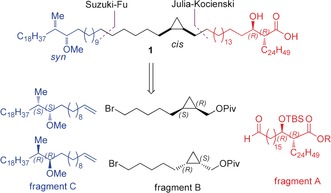
Retrosynthetic analysis.

The development of mycolic acids and glucose monomycolates as potential antigens in vaccine development and diagnostics requires synthetic material strictly free from immunologically‐active impurities. This was our incentive to undertake the total synthesis of four stereoisomers of methoxy mycolic acid (Scheme 1, so *S,S* and *R,R*‐stereochemistry in the α‐methyl methoxy moiety plus *R,S* and *S,R*‐stereochemistry in the *cis*‐cyclopropyl moiety) and their glucose esters. This endeavour required a drastic decrease in the number of synthetic transformations compared to the previous synthesis by Baird et al.^11^ Therefore we opted for incorporation of the Suzuki–Fu cross‐coupling on several places in the design. Although the Suzuki‐Fu cross‐coupling^19^ has hardly been applied in natural product synthesis, we advocate the use of this Pd‐catalysed sp^3^–sp^3^ cross‐coupling reaction as it avoids the various functional group transformations and basic reaction conditions required for Wittig‐type and Julia‐type reactions.

In order to gain access to the four diastereomers in a convergent manner and on gram scale, we retrosynthetically dissected the molecule in three fragments, each containing a chiral segment (Scheme 1). Both enantiomers of fragments B and C had to be prepared, and combined with the natural enantiomer of fragment A. Whereas fragment B and C were planned to be connected via a Suzuki–Fu cross‐coupling reaction, unification of C–B to A using this reaction could not be achieved since, as model studies showed, with a cyclopropylmethyl bromide the reaction failed. A Julia–Kocienski olefination was projected instead.

## Results and Discussion

### Synthesis of the Fragments

**Scheme 2:**
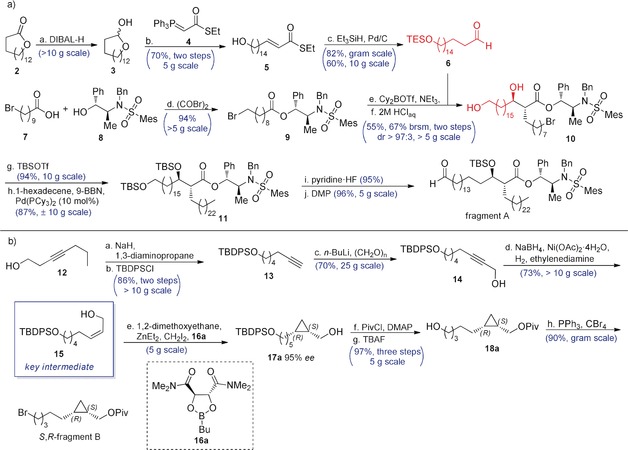
**a**. The synthesis of fragment A. a) DIBAL, CH_2_Cl_2_, −78 °C, 1 h; b) **4**, THF/toluene (2:1), 75 °C, 16 h, (70 % over two steps); c) Et_3_SiH, Pd/C, acetone, 0 °C to rt, 6 h, (82 %); d) (COBr)_2_, **8**, pyridine, CH_2_Cl_2_, 0 °C to rt, 3 h (94 %); e) Cy_2_BOTf, NEt_3_, THF, −78 °C, then **6**, 16 h; f) 2 m HCl_aq_, THF, rt, 2 h (55 % over two steps, 67 % yield brsm); g) TBSOTf, 2,6‐lutidine, CH_2_Cl_2_, 0 °C to rt, 3 h (94 %); h) 1‐hexadecene, 9‐BBN, Pd(PCy_3_)_2_ (10 mol %), K_3_PO_4_⋅H_2_O, THF, rt, 16 h (87 %); i) pyridine⋅HF, THF, 0 °C to rt, 4 h (95 %); j) DMP, CH_2_Cl_2_, rt 1 h (96 %). **b**. Synthesis of fragment B. a) NaH, 1,3‐diaminopropane, 70 °C, 1.5 h; b) TBDPSCl, CH_2_Cl_2_, 0 °C to rt, 16 h (86 % over two steps); c) *n*‐BuLi, paraformaldehyde, THF, 0 °C to rt, 16 h (70 %); d) NaBH_4_, Ni(OAc)_2_⋅4 H_2_O, H_2_, ethylenediamine, EtOH, rt, 15 min (73 %); e) 1,2‐dimethoxyethane, diethylzinc, diiodomethane, **16**, CH_2_Cl_2_, −15 °C to rt, 16 h; f) PivCl, DMAP, pyridine, CH_2_Cl_2_, 0 °C to rt, 16 h; g) TBAF, THF, 0 °C to rt, 16 h ( 97 % over three steps); h) CBr_4_, PPh_3_, CH_2_Cl_2_, 0 °C to rt, 3 h (90 %).^22^ DIBAL=diisobutylaluminium hydride, THF=tetrahydrofurane, TBSOTf=*tert*‐Butyldimethylsilyltrifluoromethanesulfonate, 9‐BBN=9‐borabicyclo(3.3.1)nonane, DMP=Dess–Martin periodinane, TBDPSCl=*tert*‐Butyldiphenylsilyl chloride, PivCl=pivaloyl chloride, TBAF=tetrabutylammonium fluoride.

The α‐branched β‐hydroxy acid moiety in fragment A was installed via an *anti*‐selective Abiko–Masamune asymmetric aldol reaction^20^ between aldehyde **6** and chiral ester **9** (Scheme 2 a). The synthesis of **6** started with a DIBAL reduction of commercially available **2** to the corresponding lactol **3**, directly followed by a Wittig olefination with **4** 21 resulting in **5** in 70 % yield over these two steps. The α,β‐unsaturated thioester **5** was then silylated, saturated, and reduced to the aldehyde in a single step in 82 % yield by a Fukuyama reduction. Esterification of chiral auxiliary **8** with the acyl bromide of **7** gave **9** in nearly quantitative yield. The subsequent Abiko–Masamune aldol reaction of **9** and **6** resulted in a rewarding *dr* of 9:1, and partial hydrolysis of the TES ether. Therefore, the crude was treated with aqueous HCl in THF and subsequently purified by column chromatography to provide diol **10** in 55 % yield and *dr* >97:3. Bis‐TBS protection of **10**, followed by a Suzuki‐Fu coupling reaction^19^ with 1‐hexadecene resulted in **11** in good yields, and effectively installed the required α‐chain. Selective deprotection of the primary TBS ether with pyridine⋅HF, followed by DMP oxidation of the primary alcohol to the corresponding aldehyde resulted in fragment A.

The synthesis of fragment B started with the alkyne zipper reaction^23^ applied to commercial **12**, followed by protection of the alcohol to provide terminal alkyne **13** in 86 % yield (Scheme 2 b). Deprotonation of **13** with *n*‐BuLi at 0 °C followed by addition to paraformaldehyde resulted in **14** in 70 % yield. In our hands, Lindlar reduction of **14** 24 to *cis* allylic alcohol **15** resulted in a mixture with the *trans* isomer, inseparable on silica and Ag‐impregnated silica. Although this problem was avoided by subjecting **14** to a P‐2 nickel reduction,^25^ this time ^1^H‐NMR indicated concomitant over‐reduction. Fortunately, separation of **15** from approximately 15 % of the aliphatic alcohol succeeded with a single run on a Ag‐impregnated silica column in 73 % yield. Alkene **15** was cyclopropanated with 95 % *ee* (see SI), mediated by dioxaborolane **16 a** or **16 b** according to Charette's procedure yielding the enantiomeric alcohols **17 a** and **17 b,** respectively.^26^ The alcohols **17 a** and **17 b** were subsequently converted into their pivaloyl esters, and desilylated using TBAF to yield both enantiomers of **18** in excellent yield over three steps,^22^ and bromination resulted in both enantiomers of fragment B. Fragment B decomposed upon extended storage (longer than one month) at 0 °C, and was therefore used within one week after synthesis.

**Scheme 3:**
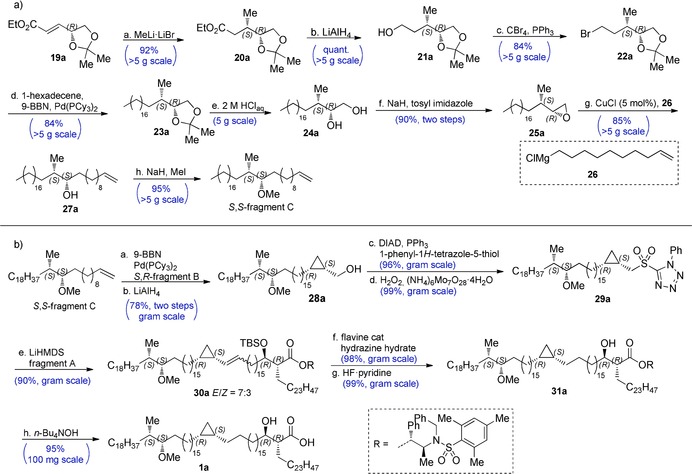
a) The synthesis of fragment C. a: MeLi⋅LiBr, Et_2_O, −78 °C, 4 h (92 %); b: LiAlH_4_, THF, 0 °C, 1 h (quant.); c: CBr_4_, PPh_3_, CH_2_Cl_2_, 0 °C to rt, 4 h (84 %); d: 9‐BBN, 1‐hexadecene, K_3_PO_4_, cat. Pd(PCy_3_)_2_, THF, 16 h (84 %); e: 2 m HClaq, THF, 95 °C, 16 h; f: NaH, tosylimidazole, THF, rt, 16 h (90 % over two steps); g: **26**, CuCl (5 mol %), THF, −10 °C, 2 h (85 %); h: MeI, NaH, THF, rt, 16 h (95 %).^22^ b) The endgame synthesis. a: 9‐BBN, 1‐hexadecene, fragment B, K_3_PO_4_, Pd(PCy_3_)_2_ (7 mol %), THF, 16 h; b: LiAlH_4_, THF, 0 °C, 15 min (78 % over two steps); c: phenyl‐1*H*‐tetrazole‐5‐thiol, PPh_3_, DIAD, THF, 0 °C to rt, 1 h (96 %); d: H_2_O_2_ (30 %), (NH_4_)_6_Mo_7_O_28_⋅4 H_2_O, THF, EtOH, *n*‐BuOH, rt, 5 d (99 %); e: LiHMDS, fragment A, −35 °C, 1 h (90 %); f: flavine cat.,^30^ hydrazine hydrate, THF, EtOH, *n*‐BuOH, rt, 5–7 d (98 %); g: HF⋅pyridine, CHCl_3_, 0 °C, 2 h (99 %); h: *n*‐Bu_4_NOH, (1.5 m in water), THF, rt, 16 h (95 %).^22^ Tosylimidazole=*p*‐toluenesulfonylimidazole, DIAD=diisopropylazodicarboxylate, LiHMDS=lithium hexamethyldisilazane.

The synthesis of fragment C (Scheme 3 a) started with a diastereoselective conjugate addition of MeLi to **19 a** and its enantiomer **19 b**.^11^, ^27^ Then, LiAlH_4_ reduction followed by bromination of **21** resulted in bromide **22** in good yields over two steps. Chain extension was achieved by subjecting **22** to a Suzuki‐Fu cross‐coupling^19^ with 1‐hexadecene. Acid hydrolysis of the acetal, followed by a one‐pot tosylation of the primary alcohol and intramolecular S_N_2 reaction went smoothly, and yielded the corresponding epoxide **25** in excellent yield over two steps. Epoxide opening with Grignard reagent **26** using catalytic CuCl minimized the formation of halohydrin by‐products^28^ and afforded the secondary alcohol **27** in good yield. Finally, methylation of the secondary hydroxyl using an excess of MeI and NaH resulted in both enantiomers of fragment C in nearly quantitative yields.

### Endgame of the Synthesis

With all three fragments in hand, we initiated the endgame by yet another Suzuki‐Fu cross‐coupling to connect the fragments B and C (Scheme 3 b). Reductive removal of the pivaloate, and a subsequent Mitsunobu reaction with 1‐phenyl‐1*H*‐tetrazole‐5‐thiol afforded the thio‐ether in good yields over three steps. Unfortunately, ammonium heptamolybdate/H_2_O_2_ oxidation to sulfone **29** proved to be very sluggish due to limited solubility of the starting material in EtOH/THF, whereas oxidation with *m*‐CPBA resulted in a complex mixture of unidentifiable products. Although oxidation with RuO_4_ was possible,^29^ concomitant oxidation of the methoxy function to the ketone was hard to suppress. Fortunately, acceptable rates in the ammonium heptamolybdate/H_2_O_2_ oxidation were achieved by addition of *n*‐BuOH as co‐solvent, resulting in nearly quantitative yields of the sulfone. Subsequently, we applied a Julia–Kocienski olefination for the final coupling of **29** with fragment A. Although initially excellent yields (85–90 %) were achieved in this reaction, at some point we suffered from irreproducible yields, even when we applied starting materials that had provided satisfactory yields earlier. We could circumvent this problem by using an excess of lithiated **29**, which was nearly quantitatively recovered. With the complete backbone in place, we subjected **30** to a diimide reduction^30^ of the olefin, followed by TBS deprotection with pyridine⋅HF in excellent yield. The use of *n*‐BuOH as cosolvent proved again to be essential for acceptable conversion rates. As we were reluctant to store large quantities of the mycolic acids because of the potentially sensitive hydroxy acid moiety, the final step was carried out portion‐wise at sub‐gram scale. Removal of the chiral auxiliary with LiOH, LiOH/H_2_O_2_ or hydrogenolysis were unsuccessful, but hydrolysis with 2 equiv of tetrabutylammonium hydroxide (*n*‐Bu_4_NOH) in THF at rt resulted in complete and clean conversion, and yielded the desired mycolic acids in sufficient purity and quantitative yields after a straightforward pentane/acetonitrile extraction. Overall, the total synthesis of mycolic acid **1 a** was achieved in a longest linear sequence of just 17 steps, in 15 % yield. The corresponding glucose esters (Figure 2, **32 a**–**d**) were obtained in two steps using a method developed by Prandi^31^ (see SI for experimental details and yields).

### The Stereochemistry of Natural Methoxy Mycolic Acid

With all four diastereomers in hand, it was now possible to prove or disprove the ascription of the absolute stereochemistry (*S*,*S*) of the *syn*‐methoxy methyl moiety by Asselineau et al.^7^ An aliquot of the carboxylic acids was treated with (trimethylsilyl)diazomethane in toluene/methanol to provide the methyl esters (see SI). The [α]D of both methyl esters with *S,S* configuration in the α‐methyl methoxy segment (**1 a** and **1 d**) was 0°, which corresponds with the specific rotation of the natural sample reported before.^7^ Consequently, also the specific molar rotations [Φ]_D_ were 0°. This supports the previous assignment of the stereochemistry by Asselineau, and Baird (for **1 d**). The [α]_D_ for the diastereomers with *R*,*R* configuration in the α‐methyl methoxy segment was +7.8° for **1 b** and +7.9° for **1 c** and similar to those reported by Baird and co‐workers for (synthetic) diastereomer **1 c**.^11^ The [Φ]_D_ of **1 b** and **1 c** was +99° and +100°, respectively, and comparable with the sum of the values of the [Φ]_D_ of the α‐methyl methoxy and the β‐hydroxy acid segments prepared earlier by Asselineau (+90°). Therefore, it is reasonable to conclude that the remotely located stereocenters in methoxy mycolic acid do not influence each other's optical rotation. The principle of optical superposition is therefore applicable here and the assignment of the absolute stereochemistry by Asselineau is correct, although the stereochemistry in the cyclopropyl ring remains to be determined (see below).

**Figure 3:**
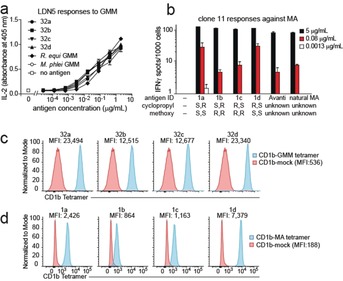
Biological validation of synthetic glucose methoxy mycolates (a,c) and methoxy mycolic acids (b,d). Methods are provided in the Supporting Information.

Next, we desired to determine whether our synthetic methoxy mycolic acids are biologically active as antigens for human T cells. In the presence of CD1b‐expressing antigen‐presenting cells, all four synthetic stereoisomers of glucose monomycolate activated the LDN5 T cell line in a dose‐dependent manner, as determined by release of the cytokine Interleukine‐2 (IL‐2) (Figure 3 a). The level of activation by the four synthetic diastereomers, the natural long glucose mycolate from *Mycobacterium phlei*, and the natural short glucose monomycolate from *Rodococcus equi*, were comparable. This was consistent with previous observations that glucose monomycolate‐specific T cells are insensitive to variations in the distal parts of mycolic acid, but highly sensitive to changes in or near the glucose.^17^

As for the T cell activation assays with free mycolic acid, it is known from studies with natural mycolic acid isolates that a T cell clone is typically more potently activated by a certain type of mycolic acid (α‐, keto‐, or methoxy‐) than by another. The pattern of antigen potency is different for each T cell clone. In the presence of CD1b‐expressing antigen‐presenting cells, all four synthetic mycolic acids stimulated the T cell clone 11 well at the highest concentration tested, 5 μg mL^−1^ (Figure 3 b). The two synthetic mycolic acids with the *S,S* configuration at the α‐methyl methoxy segment, however, were better agonists than those with *R,R* configuration, and better than the natural mixture of *Mtb*‐derived mycolic acids when tested at the suboptimal concentration of 0.08 μg mL^−1^. No significant difference was found for the stereochemistry at the cyclopropyl group.

As an alternative bioassay, mycolic acid and glucose monomycolate can be loaded into fluorescently labelled CD1b tetramers in vitro. Tetramerized, fluorescently labelled CD1b–lipid complexes (“tetramers”) can be used to study glucose mycolate^32^ and mycolic acid^6^ specific T cells at single‐cell level, in combination with fluorescently labelled antibodies, in flow cytometry. The fluorescence level of the cells is an indication of the strength of the interaction between T cell receptor and antigenic target, CD1b–mycolate.

All synthetic glucose mycolates, when loaded into CD1b tetramers, stained LDN5 well (Figure 3 c). A minor but noticeable difference in the mean fluorescence intensity (MFI) is observed depending on the stereochemistry of the α‐methyl methoxy unit. The MFI with the GMMs with the *S,S* configuration is twice as high as in those with the *R,R* configuration. No noticeable difference is seen between the tetramers loaded with the glucose mycolates that differ in the stereochemistry of their cyclopropyl group.

For the mycolic acids, however, more distinct differences are observed (Figure 3 d). Tetramers loaded with mycolic acids with the *S,S*‐methoxy methyl unit give much higher MFIs than their *R,R* counterparts.

In addition to the effects of the configuration of the methoxy methyl group, mycolic acids with an *R,S*‐cyclopropyl unit have a moderately increased MFI compared to their *S,R* counterparts (MFI compound **1 d**>**1 a** and compound **1 c**>**1 b** in Figure 3 d). If we assume that the naturally occurring configuration of the cyclopropyl provides the best CD1b loading or the highest affinity for the T cell receptor, this observation suggests the *R,S*‐stereochemistry of the cyclopropyl group in natural mycolic acids.

## Conclusion

In conclusion, the synthetic route presented here provides access to gram amounts of methoxy mycolic acid. The successful repetitive application of the Suzuki–Fu cross‐coupling and the *anti*‐selective Abiko–Masamune asymmetric aldol reaction in this natural product synthesis are instrumental to this success. The availability of all four diastereomers allowed to confirm the *S,S* stereochemistry of the methoxy methyl function in natural methoxy mycolic acid, as inferred by Asselineau and Baird. The T cell receptor is sensitive to variations in the stereochemistry of the side chain of free mycolic acid, and not in that of glucose monomycolate. Although the absolute stereochemistry of the *cis*‐cyclopropyl function could not be established, the CD1b‐free mycolic acid tetramer staining experiments favour *R,S* stereochemistry. The availability of large amounts of fully synthetic methoxy mycolic acid and glucose mycolate (GMM) allows further study into their application in vaccine development and diagnostics.

## Conflict of interest

The authors declare no conflict of interest.

## Supporting information

As a service to our authors and readers, this journal provides supporting information supplied by the authors. Such materials are peer reviewed and may be re‐organized for online delivery, but are not copy‐edited or typeset. Technical support issues arising from supporting information (other than missing files) should be addressed to the authors.

Supplementary
