# Supplementary material for: Total Synthesis of a Mycolic Acid from Mycobacterium tuberculosis
Source: Angew Chem Int Ed Engl. 2020 Mar 10;59(19):7555–60. doi: 10.1002/anie.202000523 (PMC7216993; doi:10.1002/anie.202000523)
Supplement: Supplementary file 1 — Supplementary [file ANIE-59-7555-s001.pdf]

Angewandte Chemie
Eine Zeitschrift der Gesellschaft Deutscher Chemiker

Supporting Information

# Total Synthesis of a Mycolic Acid from *Mycobacterium tuberculosis*

*Nabil Tahiri, Peter Fodran, Dhineshkumar Jayaraman, Jeffrey Buter, Martin D. Witte, Tonatiuh A. Ocampo, D. Branch Moody, Ildiko Van Rhijn, and Adriaan J. Minnaard**

anie_202000523_sm_miscellaneous_information.pdf

# Contents

**Experimental Procedures** .......... 3

**General info** .......... 3

**Biological validation of methoxymycolic acids** .......... 3

**Fragment A:** .......... 4

**Fragment B:** .......... 7

**Fragment C:** .......... 12

**Endgame:** .......... 18

**References** .......... 32

**NMR spectra** .......... 32

## Experimental Procedures

### General info

All moisture sensitive reactions were performed using flame-dried glassware under nitrogen atmosphere using standard Schlenk techniques and anhydrous solvents. Reaction temperatures below 0 °C refer to internal temperatures, while reaction temperatures higher than rt refer to heating bath temperatures. Anhydrous solvents were taken from a MBraun solvent purification system (SPS-800). All other reagents were purchased from Sigma-Aldrich, Acros, TCI Europe, Strem chemicals or Fluorochem and used without further purification unless noted otherwise.
$CsHCO_3$ used for the formation of GMMs was dried overnight under high vacuum (0.01 mbar) at 120 °C and stored in the glovebox for later use.
Solid LiHMDS was obtained from Sigma-Aldrich, or by addition of HMDS (1.1 equiv.) to BuLi (1.6 M in hexanes, 1.0 equiv.) at -20 °C. The formed crystals were filtered under inert atmosphere and stored in the glovebox for later use.
TLC analysis was performed with Merck silica gel 60/Kieselguhr F245, 0.25 mm. Compounds were visualized using either a $KMnO_4$ stain ($K_2CO_3$ (40 g), $KMnO_4$ (6 g), water (600 ml) and 10% NaOH (5 ml)), anisaldehyde stain (EtOH (135 ml), $H_2SO_4$ (5 ml), AcOH (1.5 ml), p-anisaldehyde (3.7 ml)), PMA stain (phosphomolybdic acid (10 g) in ethanol (100 ml)) or elemental iodine.
Flash chromatography was performed using SiliCycle silica gel type SiliaFlash P60 (230-400 mesh) as obtained from Screening Devices.
$^1$H- and $^{13}$C-NMR spectra were recorded on an Agilent MR400 (400 and 100 MHz, respectively) or a Bruker Avance NEO 600 (600 and 150 MHz, respectively). $CDCl_3$ was used as solvent unless stated otherwise. Chemical shift values are reported in ppm with the solvent resonance as the internal standard ($CDCl_3$: δ7.26 for $^1$H, δ77.16 for $^{13}$C). Data are reported as follows: chemical shifts, multiplicity (s = singlet, d = doublet, dd = double doublet, ddd = double double doublet, dt = double triplte, td = triple doublet, t = triplet, q = quartet, p = pentet, b = broad, m = multiplet), coupling constants *J* (Hz), and integration.
Enantiomeric excesses were determined by Chiral HPLC analysis using a Shimadzu LC-10ADVP HPLC instrument equipped with a Shimadzu SPD-M10AVP diode-array detector. Integration was performed at 254 nm and retention times are given in min.
High-resolution mass spectra (HRMS) were recorded on a Thermo Scientific LTQ Orbitrap XL (ESI+, ESI-and APCI).
Optical rotations were measured on a Schmidt+Haensch polarimeter (Polartronic MH8) with a 10 cm cell (c given in g/mL) at ambient temperature (±20 °C).

### Biological validation of methoxymycolic acids

Glucose monomycolates were tested in cellular activation using a T cell line named LDN5 and a CD1b transfected C1R cell line (C1R.CD1b) as an antigen presenting cell. 100,000 LDN5 T cells were co-incubated with 50,000 C1R.CD1b cells in T cell medium[1] containing glucose monomycolate in concentrations between 2.0 - 0.016 µg/ml or medium without glucose monomycolate. The following day the supernatant was examined for the presence of the cytokine IL-2. Mycolic acids were tested in an IFNɣ ELISPOT assay using a monoclonal cell line named clone 11, derived from human blood.[2] 800 Clone 11 T cells were co-incubated with 50,000 C1R.CD1b cells in T cell medium containing mycolic acid in concentrations of 5.0, 0.08, and 0.0013 µg/ml. All conditions were tested in biological duplicates. Organic solvent was evaporated in a stream of $N_2$ gas and lipids were sonicated into T cell medium at room temperature. Along with the synthetic mycolates, natural long and short chain glucose monomycolates were isolated from *Mycobacterium phlei* and *Rhodococcus equi* respectively (isolated in house as described[3]), purchased methoxy mycolic acid from Avanti Lipids (#79128), and natural long chain mycolic acid isolated from *Mycobacterium tuberculosis* (Sigma) were included as positive controls in these functional assays.

For tetramer studies, glucose monomycolates and mycolic acids were loaded into monomeric, biotinylated CD1b protein (NIH tetramer facility). Organic solvent was evaporated in a stream of $N_2$ gas and lipids were sonicated in tetramer loading buffer at 37 °C for 2 hours. Glucose monomycolates were loaded in a citrate buffer of pH 7.4 with 0.5 % 3-[(3-cholamidopropyl)dimethylammonio]-1-propanesulfonate (CHAPS) detergent (Sigma) as previously described.[4] Mycolic acids were loaded in a citrate buffer of pH 4.5 with 0.6% CHAPS detergent. CD1b protein was added and incubated overnight at 37 °C. After loading mycolic acids, the pH was adjusted to 7.4 using 1 M tris(hydroxymethyl)aminomethane pH 8.5 as previously described.[2] Lipid-loaded CD1b monomers were then tetramerized with streptavidin conjugated to a phycoerythrin (PE) (Molecular probes). Glucose monomycolate or mycolic acid tetramers were used to stain LDN5 or Clone 11 cells, respectively, and were analyzed in a 5-laser BD LSRFortessa flow cytometer.

**Fragment A:**

**Compound 5:**

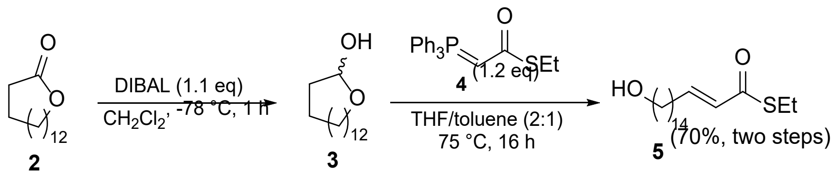

To a cooled solution of lactone **2** (14.0 g, 58.2 mmol) in $CH_2Cl_2$ (290 ml) at -80 °C was added DIBAL (1 M in $CH_2Cl_2$, 64 ml, 64 mmol, 1.1 equiv.) at such a rate that the temperature stayed below -75 °C. When the reaction was complete according to TLC (1.5 h), the reaction was quenched by careful addition of water (2.56 ml), $NaOH_{aq}$ (15%, 2.56 ml) and water (6.4 ml). The mixture was allowed to reach rt by removal of the cooling bath. After 15 min stirring at rt, anhydrous $MgSO_4$ was added and stirring was continued for 15 additional min. Then the mixture was filtered and concentrated *in vacuo*, yielding the crude lactol **3** (11.2 g, 46.2 mmol,) as a white solid.

*The obtained lactol was submitted into the HWE reaction in two batches of 5.2 g:*

To a solution of the crude lactol **3** (5.2 g, 23 mmol) in toluene (80 ml) at 75 °C was added **4** (10 g, 28 mmol, 1.2 equiv.),[5] followed by sufficient THF to make the reaction completely homogeneous (around 40 ml). After overnight stirring, TLC indicated complete consumption of the lactol. The reaction mixture was allowed to cool to rt and absorbed on Celite. Purification by flash column chromatography using pentane/ether (6:4) yielded a yellow solid (13.39 g, 40.77 mmol, 70% combined yield, over two steps).

**$^1$H-NMR** (400 MHz, $CDCl_3$) δ 6.89 (dt, *J* = 14.6, 6.9 Hz, 1H), 6.10 (d, *J* = 15.5 Hz, 1H), 3.64 (t, *J* = 6.6 Hz, 2H), 2.94 (q, *J* = 7.5 Hz, 2H), 2.18 (q, *J* = 7.3 Hz, 2H), 1.56 (p, *J* = 6.8 Hz, 2H), 1.49 – 1.19 (m, 26H). **$^{13}$C-NMR** (101 MHz, $CDCl_3$) δ 190.31, 145.55, 128.71, 63.02, 32.86, 32.24, 29.70, 29.67, 29.57, 29.52, 29.44, 29.23, 28.06, 25.84, 23.09, 14.89. **HRMS (ESI)** Calcd. for $C_{19}H_{36}NaO_2S$ ($[M + Na]^+$): 351.2328, found: 351.2335.

**Compound 6:**

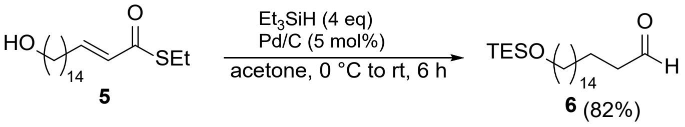

Thioester **5** (1.0 g, 3.0 mmol) was dissolved in acetone (30 ml) and cooled to 0 °C using an ice-water bath, and Pd/C (10 weight%, 161 mg, 0.15 mmol, 5 mol%) was added in one portion. Triethylsilane (1.94 ml, 12.1 mmol, 4 equiv.) was added over 15 min, after which the cooling bath was removed and the reaction was stirred at rt for 5 h. Then, celite was added, and the volatiles were removed *in vacuo*. Flash column purification using 2-4% ether in pentane yielded a colorless liquid (0.95 g, 2.46 mmol, 82% yield).
**$^1$H-NMR** (400 MHz, $CDCl_3$) δ 9.76 (t, *J* = 1.9 Hz, 1H), 3.59 (t, *J* = 6.7 Hz, 2H), 2.41 (td, *J* = 7.4, 1.8 Hz, 2H), 1.62 (q, *J* = 7.2 Hz, 2H), 1.56 – 1.48 (m, 2H), 1.37 – 1.21 (m, 24H), 0.96 (t, *J* = 7.9 Hz, 9H), 0.59 (q, *J* = 8.0 Hz, 6H). **$^{13}$C NMR** (101 MHz, $CDCl_3$) δ 202.96, 63.09, 44.00, 33.03, 29.77, 29.75, 29.72, 29.69, 29.58, 29.54, 29.46, 29.27, 25.94, 22.19, 6.85, 6.67, 5.91, 4.53. **HRMS (ESI)** Calcd. for $C_{23}H_{48}NaO_2Si$ ($[M + Na]^+$): 407.3316, found: 407.3325.

*Note: the reaction could be scaled up to a 12 gram scale, with a reduced yield of approximately 60%.*

**Compound 9:**

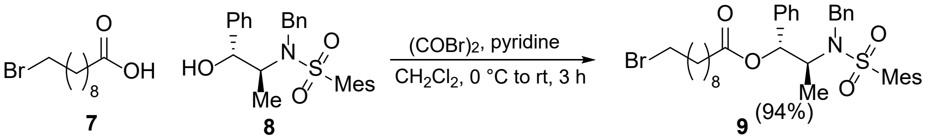

To a solution, cooled at 0 °C, of oxalyl bromide (2 M in $CH_2Cl_2$, 8.9 ml, 17.8 mmol, 1.4 equiv.) was added 10-bromodecanoic acid (**7**) (4.47 g, 17.8 mmol, 1.4 equiv.) in small portions. After complete addition, the RM was allowed to warm up to rt and stirred at this temperature until gas formation ceased (around 1 h). Quenching of a small sample with methanol and elution on TLC (pentane /ethyl acetate 8:2) showed complete conversion into the methyl ester, and thus complete conversion of the carboxylic acid into the acid bromide.

In a separate Schlenk flask, **8** (5.38 g, 12.7 mmol, 1.0 equiv.) was dissolved in $CH_2Cl_2$ (60 ml), followed by the addition of pyridine (3.02 g, 38.1 mmol, 3.1 ml, 3.0 equiv.) and the mixture was cooled to 0 °C using an ice-water bath. Then, the freshly made acid bromide solution was carefully added after which the reaction was allowed to stir at rt for 2 h. After TLC confirmed complete consumption of **8**, the reaction mixture was diluted with $CH_2Cl_2$ (25 ml) and washed with saturated aqueous $NaHCO_3$ (100 ml). The aqueous layer was back extracted with $CH_2Cl_2$ (2× 75 ml) and the combined organic layer was washed with 1 M $HCl_{aq}$ (150 ml), water (150 ml), brine (150 ml), dried over $MgSO_4$ and concentrated *in vacuo*. The crude was purified by flash column chromatography with 5-10% ethyl acetate in pentane to yield a viscous colorless oil (7.87 g, 12.0 mmol, 94% yield).

**$^1$H-NMR** (400 MHz, $CDCl_3$) δ 7.32 (d, *J* = 7.0 Hz, 2H), 7.28 – 7.22 (m, 2H), 7.22 – 7.15 (m, 4H), 6.95 – 6.80 (m, 4H), 5.83 (d, *J* = 4.0 Hz, 1H), 4.73 (d, *J* = 16.6 Hz, 1H), 4.59 (d, *J* = 16.6 Hz, 1H), 4.10 – 3.99 (m, 1H), 3.39 (t, *J* = 6.8 Hz, 2H), 2.51 (s, 6H), 2.28 (s, 3H), 2.23 – 2.05 (m, 2H), 1.83 (p, *J* = 6.9 Hz, 2H), 1.50 (p, *J* = 7.5 Hz, 2H), 1.40 (p, *J* = 7.0 Hz, 2H), 1.33 – 1.17 (m, 8H), 1.13 (d, *J* = 6.9 Hz, 3H). **$^{13}$C-NMR** (101 MHz, $CDCl_3$) δ 171.95, 142.50, 140.16, 138.69, 138.60, 133.37, 132.15, 128.37, 128.34, 127.80, 127.37, 127.08, 125.94, 77.93, 56.71, 48.15, 34.12, 34.01, 32.76, 29.18, 29.09, 28.97, 28.63, 28.08, 24.61, 22.99, 20.88, 12.85. **HRMS (ESI)** Calcd. for $C_{35}H_{46}BrNNaO_4S$ ($[M + Na]^+$): 678.2223 and 680.2203, found: 678.2227 and 680.2207.

**Compound 10:**

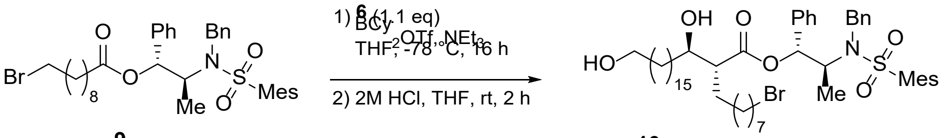


**9**

**10** (55%, two steps, *dr* > 97:3)

To a solution of **9** (10.78 g, 16.44 mmol) and triethylamine (5.5 ml, 2.4 equiv.) in $CH_2Cl_2$ (50 ml) at -80 °C was added a solution of the dicyclohexylboron triflate (12.22 g, 36.35 mmol, 2.2 equiv.) in $CH_2Cl_2$ (30 ml) via syringe pump over 2 h (maintaining an internal temperature of -78 °C). After complete addition, enol formation was allowed to take place by stirring at this temperature for an additional 5 h. Then, a solution of aldehyde **6** (7.07 g, 18.4 mmol, 1.1 equiv.) in $CH_2Cl_2$ (20 ml) was added slowly via syringe pump at a rate of 24 ml/h, and the reaction was stirred overnight at -78 °C. The reaction was quenched at -78 °C by addition of phosphate buffer (pH 7, 65 ml), MeOH (160 ml) and aqueous $H_2O_2$ (30%, 20 ml), allowed to reach rt, and stirred for another 24 h. Brine (300 ml) was added and the mixture was extracted with ether (4× 100 ml). The combined organic layer was washed with brine (200 ml), dried over $MgSO_4$ and concentrated *in vacuo* yielding a yellowish thick oil (17.33 g).
The crude was dissolved in THF (100 ml), 2 M $HCl_{aq}$ (20 ml) was added and the mixture was stirred at rt for 1 h. The mixture was neutralized by the addition of saturated aqueous $NaHCO_3$ (200 ml) and extracted with ether (3× 150 ml). The combined organic layer was dried over $MgSO_4$ and concentrated *in vacuo*. Careful flash column chromatography applying 40% ether in pentane during 6 column volumes followed by 45% ether in pentane yielded recovered **9** (2.0 g, 3.05 mmol) and **10** (8.29 g, 8.94 mmol, 55% yield, 67% yield brsm, *dr* > 97:3)

*Note: Because the diastereomers separate very poorly on TLC, all fractions with a dr > 95:5 as judged by NMR were combined. Fractions with dr < 95:5 were combined, concentrated in vacuo, and re-purified using the same conditions. The reported yield is the result of three columns.*

**$^1$H NMR** (400 MHz, $CDCl_3$) δ7.31 – 7.17 (m, 6H), 7.16 – 7.10 (m, 2H), 6.87 – 6.80 (m, 4H), 5.82 (d, J = 5.7 Hz, 1H), 4.75 (d, J = 16.3 Hz, 1H), 4.50 (d, J = 16.4 Hz, 1H), 4.16 (p, J = 6.8, 6.2 Hz, 1H), 3.63 (app. t, J = 6.7 Hz, 3H), 3.38 (t, J = 6.8 Hz, 2H), 2.42 (s, 7H), 2.36 (d, J = 7.4 Hz, 1H), 2.28 (s, 3H), 1.86 – 1.76 (m, 2H), 1.60 – 0.93 (m, 46H). **$^{13}$C NMR** (101 MHz, $CDCl_3$) δ 174.64, 142.51, 140.33, 138.39, 137.94, 133.15, 132.10, 128.31, 128.22, 128.06, 127.25, 126.69, 77.97, 77.36, 72.29, 62.90, 56.40, 51.31, 48.09, 35.30, 33.97, 32.78, 32.74, 29.68, 29.66, 29.64, 29.62, 29.59, 29.47, 29.34, 29.07, 28.51, 28.07, 27.07, 25.79, 25.59, 22.89, 20.91, 14.44. **HRMS (ESI)** Calcd. for $C_{52}H_{80}BrNNaO_6S$ ([M + Na]+): 948.4782 and 950.4780, found: 948.4790 and 950.4780.

**Compound 11:**

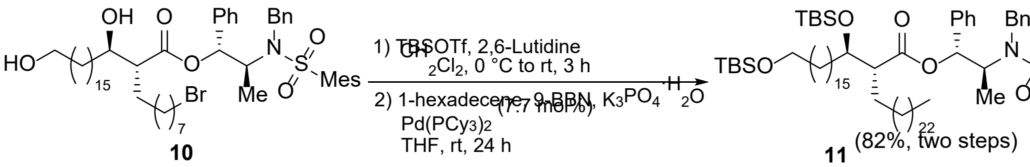


Bis-TBS protection:

To a solution of **10** cooled at 0 °C (8.30 g, 8.95 mmol, 1.0 equiv.) and 2,6-lutidine (5.76 g, 53.71 mmol, 6.2 ml, 6.0 equiv.) in $CH_2Cl_2$ (30 ml) was added TBSOTf (9.47 g, 35.8 mmol, 4.0 equiv.). After complete addition, the cooling bath was removed and the reaction mixture was allowed to stir at rt for 3 h. Then, the reaction mixture was diluted with $CH_2Cl_2$ (50 ml) and washed with 0.5 M $HCl_{aq}$ (300 ml). The layers were separated and the aqueous layer was extracted with $CH_2Cl_2$ (3× 75 ml). The combined organic layer was washed with brine (150 ml), dried over $MgSO_4$ and concentrated *in vacuo*, which yielded an orange oil. The crude was purified by column chromatography using 6% ether in pentane yielding a yellowish oil (9.68 g, 8.38 mmol, 94% yield).

**$^1$H-NMR** (400 MHz, $CDCl_3$) δ 7.35 – 7.31 (m, 2H), 7.26 – 7.16 (m, 4H), 7.12 – 7.06 (m, 2H), 6.85 – 6.78 (m, 4H), 5.72 (d, *J* = 6.2 Hz, 1H), 4.80 (d, *J* = 16.2 Hz, 1H), 4.42 (d, *J* = 16.2 Hz, 1H), 4.16 (p, *J* = 6.7 Hz, 1H), 3.91 – 3.83 (m, 1H), 3.60 (t, *J* = 6.6 Hz, 2H), 3.38 (t, *J* = 6.8 Hz, 2H), 2.52 – 2.45 (m, 1H), 2.39 (s, 6H), 2.29 (s, 3H), 1.85 – 1.76 (m, 2H), 1.55 – 1.42 (m, 4H), 1.40 – 1.06 (m, 39H), 0.96 (t, *J* = 7.1 Hz, 2H), 0.90 (s, 9H), 0.87 (s, 9H), 0.07 – 0.02 (m, 12H). **$^{13}$C-NMR** (101 MHz, $CDCl_3$) δ 172.65, 142.41, 140.40, 138.28, 133.18, 132.13, 128.38, 128.27, 128.16, 127.91, 127.37, 126.87, 77.82, 77.36, 72.77, 63.34, 56.47, 51.69, 48.22, 33.86, 33.56, 32.96, 32.82, 29.76, 29.75, 29.72, 29.70, 29.65, 29.63, 29.52, 29.12, 28.60, 28.12, 27.63, 26.91, 26.07, 25.96, 25.88, 25.01, 22.91, 20.94, 18.41, 18.13, 15.03, -4.31, -4.53, -5.17. **HRMS (ESI)** Calcd. for $C_{64}H_{108}BrNNaO_6SSi_2$ ([M + Na]$^+$): 1176.6512 and 1178.6491, found: 1176.6497 (100%) and 1178.6471 (100%).

Hydroboration:

To a solution of 1-hexadecene (2.73 g, 3.5 ml, 12.2 mmol, 1.0 equiv.) in THF (6 ml) was added 9-BBN dimer (1.73 g, 0.58 equiv.) and the resulting solution was stirred overnight at rt. NMR indicated complete conversion of the olefin (no detectable olefinic signals).

Cross-coupling:

To the preformed solution of the alkylborane (1.5 equiv.) was added $KH_2PO_4\cdot H_2O$ (2.87 g, 12.5 mmol, 1.5 equiv.) followed by $Pd(PCy_3)_2$ (425 mg, 0.64 mmol, 7.7 mol%), and the reaction mixture was stirred at rt for 30 min, which resulted in a clear yellow solution. Then, the product from the bis-TBS protection (9.59 g, 8.30 mmol, 1.0 equiv.) as a solution in THF (18 ml) was added, and the reaction mixture was stirred at rt overnight. The reaction mixture was absorbed on Celite and purified by flash column chromatography using 1-4% ether in pentane yielding **11** as a colorless oil (9.45 g, 7.26 mmol, 87% yield).

*Note: the product contained some apolar impurities which, although on TLC separable, co-eluted with the product during column purification. The product after 1 column purification was used as such in the next step since the impurities did not interfere with the reaction.*

**Fragment A:**

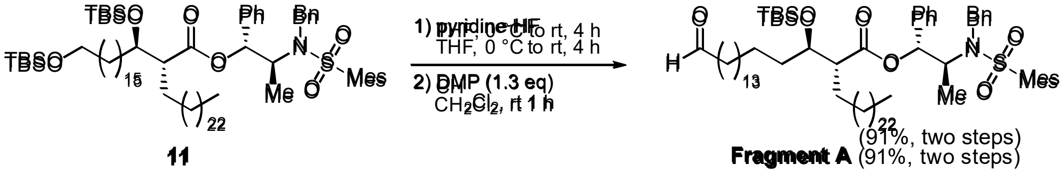


HF Stock solution:

Commercially available HF·pyridine (~70 % HF, 4.85 ml) was diluted with anhydrous THF (24 ml) and cooled to 0 °C. Then, anhydrous pyridine (9.7 ml) was added and stirred briefly at this temperature before it was used without further manipulation.

Selective deprotection:

Bis-TBS ether **11** (3.81 g, 2.93 mmol, 1.0 equiv.) was dissolved in THF (18 ml) and cooled down to 0 °C using an ice-water bath. The previously formed HF stock solution (11 ml) was added, and the mixture was allowed to reach rt by

removing the ice-water bath. The reaction was monitored closely by TLC, and quenched as soon as full conversion was reached (around 3.5 h) by careful addition of saturated aqueous $NaHCO_3$ solution (150 ml, gas formation!) while cooling with an ice-water bath to 0 °C. The mixture was extracted with $CH_2Cl_2$ (4× 30 ml), and the combined organic layer was washed with, 1 M $HCl_{aq}$ (75 ml), brine (75 ml), dried over $MgSO_4$ and concentrated *in vacuo* yielding a yellow/orange oil. The crude was purified by flash column chromatography applying a 30-60% ether in pentane gradient, which yielded a yellow oil (3.30 g, 2.78 mmol, 95% yield).

**$^1$H-NMR** (400 MHz, $CDCl_3$) δ 7.36 – 7.30 (m, 2H), 7.25 – 7.14 (m, 4H), 7.12 – 7.05 (m, 2H), 6.85 – 6.77 (m, 4H), 5.70 (d, *J* = 6.2 Hz, 1H), 4.80 (d, *J* = 16.2 Hz, 1H), 4.42 (d, *J* = 16.2 Hz, 1H), 4.15 (p, *J* = 6.8 Hz, 1H), 3.90 – 3.81 (m, 1H), 3.64 (q, *J* = 6.5 Hz, 2H), 2.48 (dt, *J* = 9.4, 4.9 Hz, 1H), 2.39 (s, 6H), 2.28 (s, 3H), 1.62 – 1.51 (m, 2H), 1.54 – 1.41 (m, 2H), 1.39 – 1.03 (m, 74H), 1.01 – 0.92 (m, 2H), 0.92 – 0.82 (m, 12H), 0.04 (s, 3H), 0.03 (s, 3H). **$^{13}$C-NMR** (101 MHz, $CDCl_3$) δ 172.81, 142.50, 140.50, 138.36, 138.32, 133.22, 132.20, 128.44, 128.32, 128.21, 127.95, 127.42, 126.91, 77.92, 72.84, 63.14, 56.55, 51.74, 48.29, 33.63, 32.94, 32.06, 29.84, 29.83, 29.81, 29.80, 29.79, 29.76, 29.74, 29.72, 29.70, 29.65, 29.58, 29.49, 29.47, 27.78, 27.02, 26.01, 25.88, 25.09, 22.97, 22.81, 20.99, 18.19, 15.04, 14.24, -4.27, -4.49. **HRMS (ESI)** Calcd. for $C_{74}H_{127}NNaO_6SSi$ ($[M + Na]^+$): 1208.9046, found: 1208.9032.

<u>DMP oxidation:</u>
The alcohol from the previous step (5.53 g, 4.66 mmol 1.0 equiv.) was dissolved in $CH_2Cl_2$ (30 ml) and the resulting solution was cooled to 0 °C using an ice-water bath. Then, Dess-Martin periodinane (DMP) (2.53 g, 5.97 mmol, 1.3 equiv.) was added, and the reaction was stirred at rt. After 2.5 h, TLC (40% ether in pentane) still indicated the presence of small amounts of starting material. Additional DMP reagent (500 mg, 1.18 mmol, 0.25 equiv.) was added, and the reaction was allowed to stir at rt for 1h, after which TLC indicated full consumption of the alcohol. The reaction mixture was concentrated *in vacuo*, redissolved in pentane (250 ml) and reagents were washed out with acetonitrile (3× 150 ml). The combined acetonitrile layers were back extracted with pentane (100 ml), and the combined pentane layers were washed with saturated aqueous $NaHCO_3$ solution (200 ml), brine (200 ml), dried over $MgSO_4$ and concentrated *in vacuo* yielding a yellowish oil. Flash column chromatography using a 10-20 % ether in pentane gradient yielded **1** as a colorless oil (5.30 g, 4.47 mmol, 96% yield).

**$^1$H-NMR** (400 MHz, $CDCl_3$) δ 9.74 (t, *J* = 2.1 Hz, 1H), 7.38 – 7.32 (m, 2H), 7.27 – 7.19 (m, 3H), 7.19 – 7.14 (m, 1H), 7.13 – 7.04 (m, 2H), 6.85 – 6.79 (m, 4H), 5.73 (d, *J* = 6.2 Hz, 1H), 4.82 (d, *J* = 16.2 Hz, 1H), 4.44 (d, *J* = 16.2 Hz, 1H), 4.16 (p, *J* = 6.7 Hz, 1H), 3.94 – 3.84 (m, 1H), 2.50 (dt, *J* = 9.5, 4.9 Hz, 1H), 2.45 – 2.34 (m, 8H), 2.28 (s, 3H), 1.68 – 1.57 (m, 2H), 1.56 – 1.04 (m, 73H), 1.02 – 0.93 (m, 2H), 0.92 – 0.84 (m, 12H), 0.05 (d, *J* = 3.1 Hz, 6H). **$^{13}$C-NMR** (101 MHz, $CDCl_3$) δ 202.92, 172.80, 142.51, 140.50, 138.38, 138.33, 133.23, 132.20, 128.45, 128.31, 128.22, 127.95, 127.42, 126.90, 77.93, 72.84, 56.56, 51.73, 48.30, 44.04, 33.63, 32.06, 29.85, 29.84, 29.81, 29.80, 29.78, 29.76, 29.74, 29.73, 29.71, 29.66, 29.57, 29.50, 29.48, 29.30, 27.79, 27.01, 26.01, 25.10, 22.98, 22.82, 22.21, 21.00, 18.20, 15.03, 14.25, -4.26, -4.49. **HRMS (ESI)** Calcd. for $C_{74}H_{129}N_2O_6SSi$ ($[M + NH_4]^+$): 1201.9335, found: 1201.9322.

**Fragment B:**

**Compound 13:**

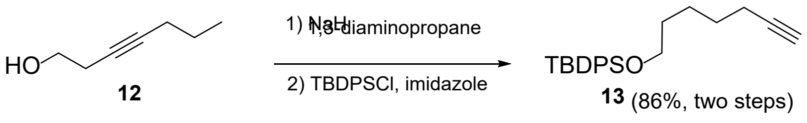


<u>Alkyne zipper reaction:</u>
NaH (60% dispersion in mineral oil, 16.0 g, 400 mmol, 3.65 equiv.) was washed with pentane (3x 50 ml) and 1,3-diaminopropane (182 ml) was added to the white solid residue. The resulting mixture was warmed to 70 °C and stirred at this temperature for 30 min (reaction turned into a dark brown solution). Then, the reaction mixture was cooled to 0 °C with an ice-water bath after which alcohol **12** (12.28 g, 109.5 mmol, 1.0 equiv.) was added neat via syringe. Upon complete addition of the alcohol, the ice-water bath was removed, and the reaction was stirred at rt for 1.5 h. Then, the reaction was cooled to 0 °C and quenched with ice-water (300 ml). The solution was extracted with ether (5× 75 ml), and the combined organic layer was washed with 2 M $HCl_{aq}$ (3× 200 ml), water (1× 200 ml), brine (1× 200 ml), dried over $MgSO_4$ and concentrated in vacuo to yield a yellow oil (8.55 g, 76.2 mmol, 70%). The water layers were back-extracted with ether to provide additional material as a dark orange oil (2.58 g, 23 mmol, 21% yield).

<u>TBDPS protection:</u>
A solution of the crude alcohol (8.55 g, 76.2, 1.0 equiv.) in $CH_2Cl_2$ (170 ml) was cooled to 0 °C using an ice-water bath, followed by the addition of imidazole (10.8 g, 158 mmol, 2.1 equiv.). After a brief stirring at 0 °C, TBDPSCl (25.14 g, 91.47 mmol, 23.8 ml, 1.2 equiv.) was added via syringe over 10 min, and the reaction mixture was stirred at 0 °C for 15 min after which it was allowed to warm up to rt overnight by removing the ice-water bath. The reaction was quenched with water

(200 ml), and the layers were separated. The water layer was extracted with $CH_2Cl_2$ (3× 75 ml). The combined organic layer was washed with brine (100 ml), dried over $MgSO_4$ and concentrated *in vacuo* yielding a yellow oil. The crude was purified by flash column chromatography by eluting with pure pentane for roughly 2 column volumes, after which a 1% ether in pentane mobile phase was applied, yielding a colorless oil (25.15 g, 71.75 mmol, 94% yield, 86% yield over two steps).

**$^1$H-NMR** (400 MHz, $CDCl_3$) δ 7.71 – 7.63 (m, 4H), 7.48 – 7.33 (m, 6H), 3.67 (t, *J* = 6.3 Hz, 2H), 2.17 (tdd, *J* = 6.8, 2.6, 1.2 Hz, 2H), 1.93 (td, *J* = 2.6, 1.2 Hz, 1H), 1.63 – 1.42 (m, 6H), 1.05 (s, 9H). **$^{13}$C-NMR** (101 MHz, $CDCl_3$) δ 135.70, 134.20, 129.65, 127.73, 84.67, 68.34, 63.84, 32.15, 28.35, 27.02, 25.12, 19.36, 18.53. **HRMS (ESI)** Calcd. for $C_{23}H_{30}NaOSi$ ([M + Na]$^+$): 373.1958, found: 373.1957.

**Compound 14:**

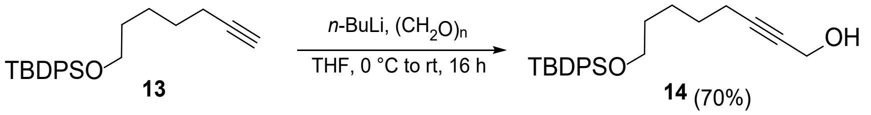


To a solution of **13** (25.10 g, 71.60 mmol, 1.0 equiv.) in THF (250 ml) at 0 °C was added *n*-BuLi (1.6 M in hexane, 82 ml, 131 mmol, 1.8 equiv.) dropwise, and the mixture was stirred at 0 °C for 30 min. Paraformaldehyde (11.3 g, 358 mmol, 5.0 equiv.) was added in portions and stirring was continued at 0 °C for 30 min. The ice-water bath was removed and the reaction was allowed to stir at rt overnight. The reaction was quenched with 1:1 saturated aqueous $NH_4Cl$/water (100 ml) and the THF was removed *in vacuo*. The aqueous residue was extracted with ether (3× 100 ml) and the combined organic layer was washed with brine (1× 200 ml), dried over $MgSO_4$ and concentrated *in vacuo*. The crude was purified by flash column chromatography using a 15-40% ether in pentane gradient yielding a colorless oil (19.15 g, 50.33 mmol, 70% yield).

**$^1$H-NMR** (400 MHz, $CDCl_3$) δ 7.70 – 7.63 (m, 4H), 7.45 – 7.32 (m, 6H), 4.29 – 4.17 (m, 2H), 3.66 (t, *J* = 6.4 Hz, 2H), 2.24 – 2.16 (m, 2H), 1.61 – 1.39 (m, 7H), 1.05 (s, 9H). **$^{13}$C-NMR** (101 MHz, $CDCl_3$) δ 135.70, 134.20, 129.66, 127.72, 86.58, 78.54, 63.85, 51.54, 32.16, 28.43, 27.01, 25.19, 19.36, 18.85. **HRMS (ESI)** Calcd. for $C_{24}H_{32}NaO_2Si$ ([M + Na]$^+$): 403.2064, found: 403.2061.

**Compound 15:**

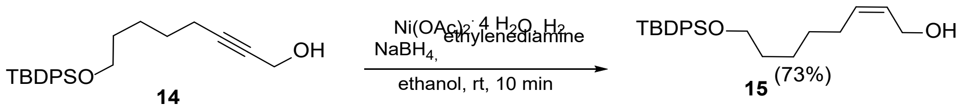


$NaBH_4$ (1.18 g, 31.3 mmol, 1.0 equiv.) was added in portions to a stirring solution of $Ni(OAc)_2{\cdot}4H_2O$ (7.78 g, 31.3 mmol, 1.0 equiv.) in absolute ethanol (150 ml) under a hydrogen blanket (1 atm) at rt, forming a black heterogeneous suspension. After 15 min stirring, ethylenediamine (3.76 g, 4.2 ml, 62.5 mmol, 2.0 equiv.) was added followed by **14** (11.90 g, 31.27 mmol, 1.0 equiv.) dissolved in EtOH (40 ml) while maintaining the hydrogen blanket (1 atm). TLC (4% ether in toluene, the TLC was run twice) showed complete conversion immediately after complete addition of **14**, after which the reaction mixture was filtered over a plug of silica. Eluting was continued with ether until TLC indicated complete recovery of the product, yielding a colorless oil (11.38 g). The crude was purified by column chromatography in two batches over $AgNO_3$ impregnated (20 wt%) silicagel (250 g), which was prepared by eluting a concentrated $AgNO_3$ solution in acetonitrile ($AgNO_3$ (50 g) dissolved in a minimal amount of acetonitrile at rt) over a dry silica column covered with aluminium household foil in order to exclude light. The column was dried by blowing compressed air through the column for 2h. Then, the column was flushed with 30% ether in pentane, the crude was loaded on the column and elution was continued with 30% ether in pentane. When most of the product had eluted from the column, the last traces of product were removed by flushing with 100% ether. All fractions with purity > 94% according to $^1$H-NMR were combined and evaporated (integral of the olefin hydrogen signal higher than 0.94 with respect to the aliphatic signal was used as a guideline for the desired purity. Spectra were recorded while applying a relaxation time of 10 sec to allow for accurate integration.). Before purifying the second batch, the column was reloaded with an additional $AgNO_3$ (35 g) by repeating the aforementioned process. Purification over two batches yielded a colorless oil which was dissolved in ether (500 ml) and washed with 12% aqueous ammonia (3× 75 ml) in order to remove any leached Ag. The combined organic layer was washed with water (2× 200 ml), dried over $MgSO_4$ and concentrated *in vacuo* to yield a colorless oil (8.70 g, 22.7 mmol, 73% yield).

**$^1$H-NMR** (600 MHz, $CDCl_3$) δ 7.62 – 7.54 (m, 4H), 7.38 – 7.26 (m, 6H), 5.58 – 5.47 (m, 1H), 5.47 – 5.39 (m, 1H), 4.11 (d, *J* = 6.0 Hz, 2H), 3.58 (t, *J* = 6.5 Hz, 2H), 2.03 – 1.94 (m, 2H), 1.53 – 1.44 (m, 2H), 1.33 – 1.25 (m, 4H), 1.10 (br. s, 1H), 0.98 (s, 9H). **$^{13}$C-NMR** (151 MHz, $CDCl_3$) δ 135.67, 134.22, 133.01, 129.62, 128.62, 127.69, 63.98, 58.66, 32.48, 29.42, 27.49, 27.01, 25.50, 19.34. **HRMS (ESI)** Calcd. for $C_{24}H_{34}NaO_2Si$ ([M + Na]$^+$): 405.2220, found: 405.2216.

**Compound 17a:**

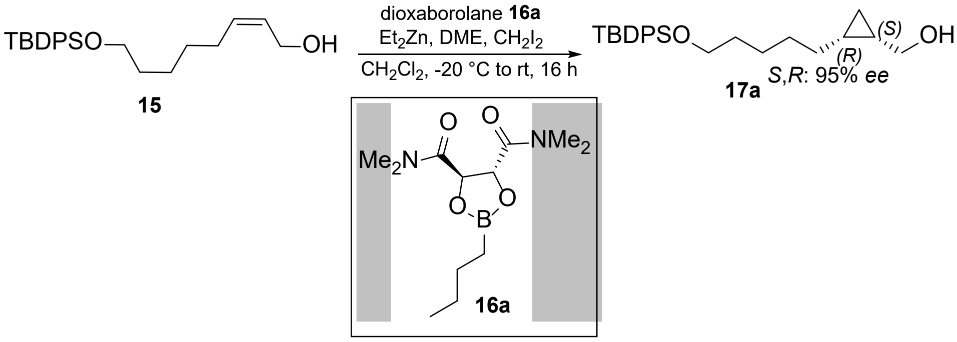


A three-neck flask was connected to a vacuum/nitrogen line, a thermometer, and the remaining neck was stoppered with a septum. The flask was charged with $CH_2Cl_2$ (60 ml) followed by 1,2-dimethoxyethane (2.13 g, 5.5 ml, 23.6 mmol, 2.0 equiv.).[6] The resulting solution was cooled to -10 °C, followed by the addition of diethylzinc (2.91 g, 2.4 ml, 23.6 mmol, 2.0 equiv.). Then, diiodomethane (12.6 g, 3.8 ml, 47.2 mmol, 4.0 equiv.) was added while keeping the internal temperature between -16 °C and -13 °C. After complete addition of the diiodomethane the reaction was left at -15 °C for 15 min. Then, dioxaborolane **16a**[6] (3.82 g, 14.2 mmol, 1.2 equiv.) as a solution in $CH_2Cl_2$ (10 ml) was added while keeping the internal temperature below -10 °C. Immediately after complete addition of **16a**, a preformed solution of alcohol **15** (4.51 g, 11.8 mmol, 1.0 equiv.) in $CH_2Cl_2$ (10 ml) was added and the reaction mixture was allowed to stir for 15 min at -15 °C. After 15 min, the cooling bath was removed and the reaction was stirred overnight at rt. The reaction was quenched by addition of saturated aqueous $NH_4Cl$ (15 ml) followed by 2 M $HCl_{aq}$ (75 ml). The mixture was diluted with ether (100 ml) and transferred to a separatory funnel. The reaction flask was rinsed with ether (25 ml) and 2 M $HCl_{aq}$ (20 ml), and both solutions were transferred to the separatory funnel. The layers were separated, and the aqueous layer was extracted with ether (2× 20 ml). The combined organic layer was transferred to an Erlenmeyer flask, and a solution containing 2 M $NaOH_{aq}$ (80 ml) and aqueous 30% $H_2O_2$ (10 ml) was added in one portion. The resulting biphasic solution was stirred vigorously for 5 min. The layers were separated and the organic layer was washed successively with 2 M $HCl_{aq}$ (80 ml), aqueous saturated sodium sulfite (80 ml), aqueous saturated sodium bicarbonate (80 ml) and brine (80 ml). The organic layer was dried over $MgSO_4$ and concentrated *in vacuo* yielding a colorless oil. The crude was purified by flash column chromatography with 30% ether in pentane yielding a colourless oil (5.11 g, 12.9 mmol, 109% yield).

**$^1$H-NMR** (400 MHz, $CDCl_3$) δ 7.74 – 7.63 (m, 4H), 7.52 – 7.32 (m, 6H), 3.71 – 3.54 (m, 4H), 1.63 – 1.52 (m, 2H), 1.46 – 1.16 (m, 7H), 1.06 (s, 9H), 0.97 – 0.81 (m, 2H), 0.75 – 0.67 (m, 1H), -0.04 (q, *J* = 5.4 Hz, 1H). **$^{13}$C-NMR** (101 MHz, $CDCl_3$) δ 135.68, 134.26, 129.60, 127.68, 64.05, 63.41, 32.67, 30.00, 28.63, 27.00, 25.81, 19.35, 18.24, 16.21, 9.62. **HRMS (ESI)** Calcd. for $C_{25}H_{36}NaO_2Si$ ($[M + Na]^+$): 419.2377, found: 419.2368.

*Note: the purified material still contained n-BuOH.*

**Compound 17b:**

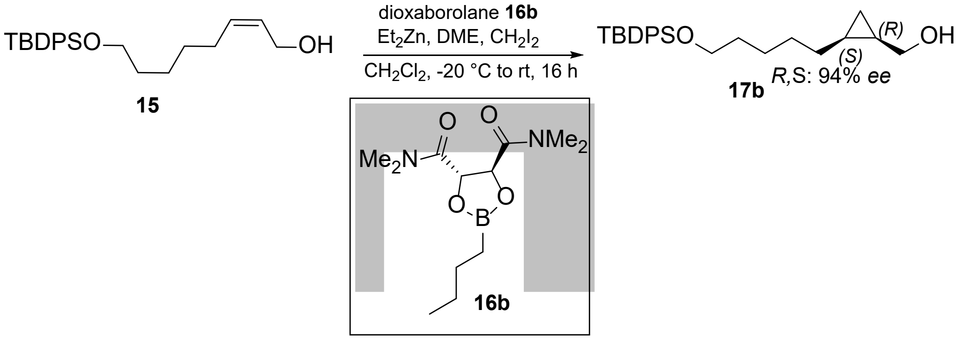


The aforementioned procedure using **15** (2.34 g, 6.13 mmol, 1.0 equiv.) and ligand **16b**[6] (2.03 g, 7.52 mmol, 1.2 equiv.) resulted in a colorless oil (2.49 g, 6.29 mmol, 103% yield) after flash column chromatography purification.

**$^1$H-NMR** (400 MHz, $CDCl_3$) δ 7.69 – 7.64 (m, 4H), 7.45 – 7.34 (m, 6H), 3.69 – 3.58 (m, 4H), 1.62 – 1.53 (m, 2H), 1.49 – 1.26 (m, 4H), 1.25 – 1.14 (m, 2H), 1.14 – 1.06 (m, 1H), 1.05 (s, 9H), 0.95 – 0.79 (m, 2H), 0.70 (td, *J* = 8.3, 4.5 Hz, 1H), -0.05 (q, *J* = 5.2 Hz, 1H). **$^{13}$C-NMR** (101 MHz, $CDCl_3$) δ 135.65, 134.22, 129.57, 127.66, 64.03, 63.31, 32.64, 29.97, 28.60, 26.98, 25.78, 19.31, 18.19, 16.17, 9.60. **HRMS (ESI)** Calcd. for $C_{25}H_{36}NaO_2Si$ ($[M + Na]^+$): 419.2377, found: 419.2375.

*Note: the purified material still contained n-BuOH.*

***Ee* determination:**

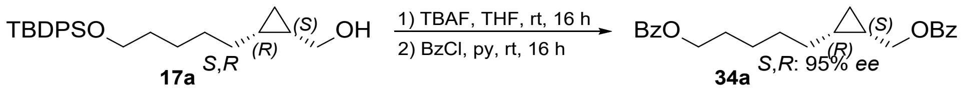


<u>Compound **34a**:</u>
Alcohol **17a** (135 mg, 0.34 mmol, 1 equiv.) was dissolved in 1 M TBAF in THF (1.0 ml, 1.02 mmol, 3.0 equiv.) and the resulting mixture was stirred overnight. The reaction mixture was concentrated *in vacuo* and purified by flash column chromatography in 100% EtOAc, which yielded the product as a colorless oil (54 mg, 0.34 mmol, quantitative yield).
The obtained diol (28 mg, 0.18 mmol, 1.0 equiv.) was dissolved in $CH_2Cl_2$ (0.3 ml), and pyridine (114 µl, 1.42 mmol, 8.0 equiv.) and benzoyl chloride (82 µl, 0.71 mmol, 4.0 equiv.) were subsequently added. After 1 h stirring at rt, TLC showed complete conversion, and *N,N*-dimethyl-1,3-diaminopropane (67 µl, 0.5 mmol, 3.0 equiv.) was added in order to quench the excess benzoyl chloride. After 1 h of stirring at rt, $CH_2Cl_2$ (10 ml) was added to the reaction mixture and the organic layer was washed successively with 1 M $HCl_{aq}$ (5 ml), saturated aqueous $NaHCO_3$ solution (5 ml), brine (5 ml), dried over $MgSO_4$ and concentrated *in vacuo* to yield the product (60 mg, 0.16 mmol, 93% yield) as a pale yellow oil.

**$^1$H-NMR** (400 MHz, $CDCl_3$) δ 8.10 – 8.01 (m, 4H), 7.59 – 7.52 (m, 2H), 7.46 – 7.40 (m, 4H), 4.50 (dd, *J* = 11.7, 6.8 Hz, 1H), 4.30 (t, *J* = 6.6 Hz, 2H), 4.15 (dd, *J* = 11.7, 8.9 Hz, 1H), 1.81 – 1.70 (m, 2H), 1.56 – 1.44 (m, 5H), 1.36 – 1.22 (m, 2H), 0.99 – 0.89 (m, 1H), 0.81 (td, *J* = 8.4, 4.8 Hz, 1H), 0.12 (q, *J* = 5.3 Hz, 1H). **$^{13}$C-NMR** (101 MHz, $CDCl_3$) δ 166.83, 166.76, 132.92, 132.91, 130.65, 130.62, 129.68, 129.64, 128.44, 128.43, 65.98, 65.12, 29.81, 28.89, 28.69, 26.07, 16.38, 14.41, 10.03. **HRMS (APCI)** Calcd. for $C_{23}H_{26}NaO_4$ ($[M + Na]^+$): 389.1723, found: 389.1719.

<u>Compound **34b**:</u>

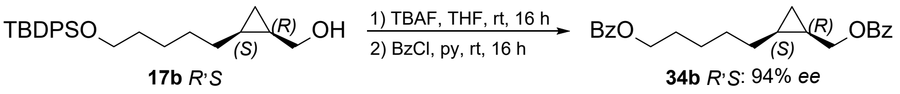


Compound **34b** could be obtained in comparable yield by subjecting alcohol **17b** to the aforementioned procedure.

**$^1$H-NMR** (400 MHz, $CDCl_3$) δ 8.09 – 8.01 (m, 4H), 7.58 – 7.52 (m, 2H), 7.47 – 7.41 (m, 4H), 4.50 (dd, *J* = 11.7, 6.8 Hz, 1H), 4.30 (t, *J* = 6.6 Hz, 2H), 4.15 (dd, *J* = 11.7, 8.9 Hz, 1H), 1.81 – 1.70 (m, 2H), 1.57 – 1.42 (m, 5H), 1.41 – 1.22 (m, 2H), 1.00 – 0.88 (m, 1H), 0.81 (td, *J* = 8.4, 4.8 Hz, 1H), 0.12 (q, *J* = 5.3 Hz, 1H). **$^{13}$C-NMR** (101 MHz, $CDCl_3$) δ 166.85, 166.78, 132.94, 132.92, 130.67, 130.63, 129.69, 129.66, 128.46, 128.45, 66.00, 65.14, 29.83, 28.91, 28.70, 26.09, 16.40, 14.42, 10.05. **HRMS (APCI)** Calcd. for $C_{23}H_{26}NaO_4$ ($[M + Na]^+$): 389.1723, found: 389.1709.

*Note: on several occasions an impurity was formed, which proved to be very hard to remove from the product by column chromatography (4% ether in toluene, stains with $KMnO_4$). This impurity only contained aromatic signals, and was also formed if the reaction was not quenched with N,N-*dimethyl-1,3-diaminopropane. *It is therefore assumed that this impurity is directly formed from the benzoyl chloride during the reaction. Although not tested in practice, the use of benzoyl anhydride instead of benzoyl chloride might prevent this issue.*

Separation of the enantiomers could be achieved using a Chiracel OD-H column, using an isocratic mobile phase consisting of 99:1 heptane/*i*-PrOH and a flow of 0.5 ml/min. Retention times are 16.8 min for **34b** (Figure 2) and 19.4 min for **34a** (Figure 1).

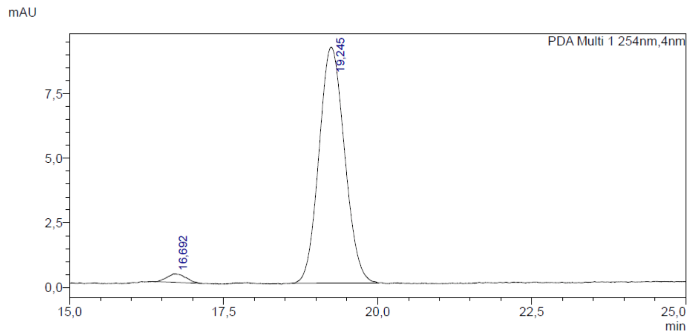


**<Peak Table>**
PDA Ch1 254nm

| Peak# | Ret. Time | Area | Height | Unit | Mark | Name | Area% |
|---|---|---|---|---|---|---|---|
| 1 | 16,692 | 7065 | 327 | | M | | 2,603 |
| 2 | 19,245 | 264386 | 9135 | | | | 97,397 |
| Total | | 271451 | 9462 | | | | 100,000 |

**Figure 1.** Chiral HPLC trace for **34a**.

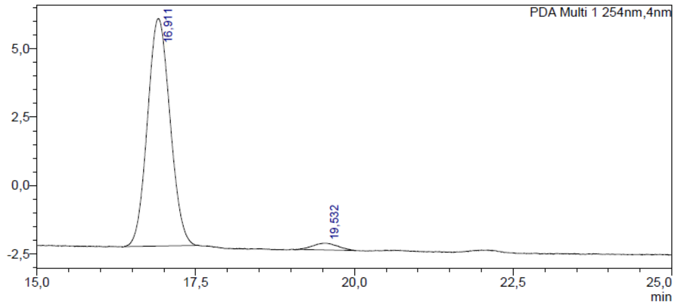


<Peak Table>

PDA Ch1 254nm

| Peak# | Ret. Time | Area | Height | Unit | Mark | Name | Area% |
|---|---|---|---|---|---|---|---|
| 1 | 16,911 | 206444 | 8322 | | | | 96,867 |
| 2 | 19,532 | 6677 | 249 | | M | | 3,133 |
| Total | | 213121 | 8571 | | | | 100,000 |

**Figure 2.** Chiral HPLC trace for **34b**.

**Compound 18a:**

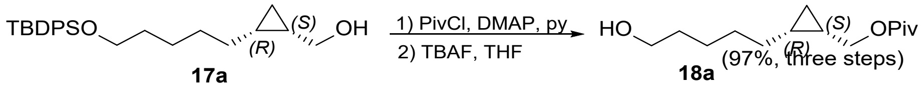


Pivaloyl protection:

To a solution of **17a** (5.10 g, 12.9 mmol, 1.0 equiv.) in $CH_2Cl_2$ (40 ml) at 0 °C were added DMAP (100 mg, 6.4 mol%) and pyridine (5.2 ml, 64 mmol, 5.0 equiv.). Then, pivaloyl chloride (3.1 g, 3.2 ml, 26 mmol, 2.0 equiv.) was added slowly via syringe. The ice-water bath was removed, and the reaction mixture was allowed to stir at rt overnight. The reaction mixture was poured into water (100 ml) and extracted with $CH_2Cl_2$ (3× 75 ml). The combined organic layer was washed with 1 M $HCl_{aq}$ (2× 100 ml), brine (1× 100 ml), dried over $MgSO_4$ and evaporated *in vacuo*. The crude was purified by flash column chromatography using 2% ether in pentane (the product was still contaminated with a considerable amount of pivaloyl chloride).

TBDPS deprotection:

The pivaloate ester was dissolved in commercial TBAF solution (1 M in THF, 25 ml, 25 mmol, 2.1 equiv.) while cooling at 0 °C with an ice-water bath. The ice-water bath was removed after 15 min, after which the reaction was allowed to stir at rt overnight. The reaction mixture was poured in water (200 ml) and extracted with $CH_2Cl_2$ (3× 75 ml). The combined organic layer was washed with brine (1× 200 ml), dried over $MgSO_4$ and concentrated *in vacuo* yielding a yellow oil. The crude was purified by flash column chromatography using a 30-50% ether in pentane gradient, affording the product (2.76, 11.4 mmol, 97% yield over three steps) as a colorless oil.

**$^1$H-NMR** (400 MHz, $CDCl_3$) δ 4.23 (dd, *J* = 11.7, 6.6 Hz, 1H), 3.86 (dd, *J* = 11.7, 9.0 Hz, 1H), 3.64 (t, *J* = 6.6 Hz, 2H), 1.62 – 1.33 (m, 8H), 1.31 – 1.23 (m, 1H), 1.21 (s, 9H), 1.18 – 1.08 (m, 1H), 0.93 – 0.81 (m, 1H), 0.73 (td, *J* = 8.4, 4.7 Hz, 1H), 0.01 (q, *J* = 5.3 Hz, 1H). **$^{13}$C-NMR** (101 MHz, $CDCl_3$) δ 178.82, 65.19, 62.74, 38.72, 32.74, 29.73, 28.58, 27.22, 25.70, 16.13, 14.15, 9.56. **HRMS (ESI)** Calcd. for $C_{14}H_{26}NaO_3$ ($[M + Na]^+$): 265.1774, found: 265.1769.

**Compound 18b:**

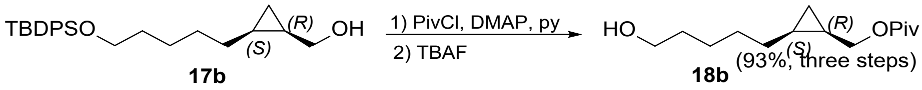


Compound **18b** (1.38 g, 5.70 mmol) was obtained in 93% yield over three steps (from **15**) after column purification by subjecting alcohol **17b** to the aforementioned procedure.

**$^1$H-NMR** (400 MHz, $CDCl_3$) δ 4.23 (dd, *J* = 11.7, 6.6 Hz, 1H), 3.86 (dd, *J* = 11.7, 8.9 Hz, 1H), 3.69 – 3.60 (m, 2H), 1.62 – 1.54 (m, 2H), 1.48 – 1.34 (m, 5H), 1.30 – 1.23 (m, 1H), 1.19 (s, 10H), 1.18 – 1.08 (m, 1H), 0.87 (h, *J* = 7.5, 7.1 Hz, 1H), 0.73 (td, *J* = 8.4, 4.7 Hz, 1H), 0.01 (q, *J* = 5.3 Hz, 1H). **$^{13}$C-NMR** (101 MHz, $CDCl_3$) δ 178.68, 65.07, 62.42, 38.59, 32.59, 29.63, 28.46, 27.09, 25.60, 16.01, 14.03, 9.43. **HRMS (ESI)** Calcd. for $C_{14}H_{26}NaO_3$ ($[M + Na]^+$): 265.1774, found: 265.1774.

***S*,*R*-fragment B:**

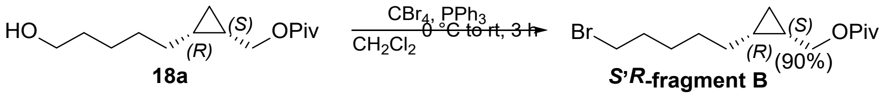

**18a** **S,R-fragment B**

To a cooled solution of alcohol **18a** (2.70 g, 11.1 mmol, 1.0 equiv.), and triphenyl phosphine (3.50 g, 13.4 mmol, 1.2 equiv.) in $CH_2Cl_2$ (40 ml) at 0 °C was added $CBr_4$ (4.06 g, 12.6 mmol, 1.1 equiv.) in portions. The ice-water bath was removed, and the reaction mixture was stirred at rt for 3 h. Then, celite (6.50 g) was added and the reaction mixture was evaporated to dryness. The residue was purified by flash column chromatography using a 2-4% ether in pentane gradient, yielding the product as a colorless oil (3.06 g, 10.0 mmol, 90% yield).

$^1$**H-NMR** (400 MHz, $CDCl_3$) δ 4.23 (dd, *J* = 11.7, 6.5 Hz, 1H), 3.85 (dd, *J* = 11.7, 9.0 Hz, 1H), 3.41 (t, *J* = 6.8 Hz, 2H), 1.86 (p, *J* = 6.9 Hz, 2H), 1.50 – 1.35 (m, 5H), 1.27 (t, *J* = 7.4 Hz, 1H), 1.21 (s, 9H), 1.18 – 1.09 (m, 1H), 0.93 – 0.79 (m, 1H), 0.74 (td, *J* = 8.4, 4.7 Hz, 1H), 0.02 (q, *J* = 5.3 Hz, 1H). $^{13}$**C-NMR** (101 MHz, $CDCl_3$) δ 178.68, 65.11, 38.77, 33.89, 32.88, 29.13, 28.47, 28.12, 27.30, 16.11, 14.24, 9.61. **HRMS (ESI)** Calcd. for $C_{14}H_{25}BrO_2$ ($[M + Na]^+$): 327.0930 and 329.0910, found: 327.0926 and 329.0905.

*Note: the product degraded upon prolonged storage (longer than one month) at 0 °C.*

***R*,*S*-fragment B:**

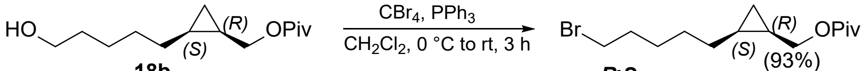

**18b** **R,S-fragment B**

***R*,*S*-fragment B** (1.48 g, 4.85 mmol) was obtained in 93% yield after column purification by subjecting alcohol **18b** (1.26 g, 5.20 mmol) to the aforementioned procedure.

$^1$**H-NMR** (400 MHz, $CDCl_3$) δ 4.23 (dd, *J* = 11.7, 6.5 Hz, 1H), 3.85 (dd, *J* = 11.7, 9.0 Hz, 1H), 3.41 (t, *J* = 6.8 Hz, 2H), 1.86 (p, *J* = 6.9 Hz, 2H), 1.52 – 1.36 (m, 5H), 1.27 (t, *J* = 7.4 Hz, 1H), 1.21 (s, 9H), 1.19 – 1.08 (m, 1H), 0.92 – 0.80 (m, 1H), 0.74 (td, *J* = 8.4, 4.7 Hz, 1H), 0.01 (q, *J* = 5.3 Hz, 1H). $^{13}$**C-NMR** (101 MHz, $CDCl_3$) δ 178.50, 64.99, 38.67, 33.76, 32.80, 29.05, 28.39, 28.04, 27.23, 16.04, 14.17, 9.52. **HRMS (ESI)** Calcd. for $C_{14}H_{25}BrO_2$ ($[M + Na]^+$): 327.0930 and 329.0910, found: 327.0929 and 329.0908.

*Note: the product degraded upon prolonged storage (longer than one month) at 0 °C.*

**Fragment C:**

**Compound 19a:**

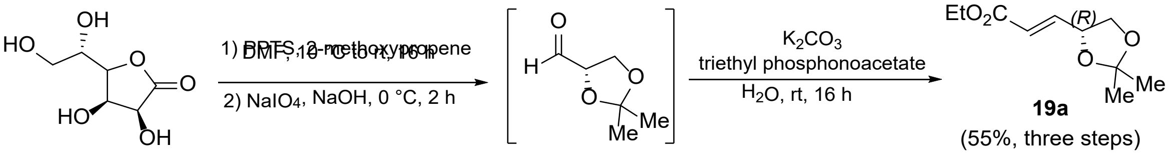

Compound **19a** was synthesized following a slightly modified literature procedure.[7] Pyridinium *p*-toluene sulfonate (1.69 g, 6.73 mmol, 5 mol%.) was added to a stirred solution of L-gulono-1,4-lactone (23.0 g, 129 mmol, 1.0 equiv.) in anhydrous DMF (200 ml) at 10 °C, followed by dropwise addition of 2-methoxypropene (12.10 g, 176.9 mmol, 1.3 equiv.). The cooling bath was removed and the reaction mixture was stirred overnight. Next day, sodium carbonate (30 g) was added, the suspension was stirred vigorously for 2 h and then filtered over Celite. The Celite was flushed with ether until TLC indicated complete recovery. The filtrate was evaporated at 45 °C and 10 mbar on the rotavap.
The yellow residue was stirred in water (190 ml) and then sodium metaperiodate (55.2 g, 258 mmol, 2.0 equiv.) was added in small portions at 0-5 °C, while maintaining the pH at 5.5 by the addition of 2 M $NaOH_{aq}$. After stirring at rt for 2 h, sodium chloride (28 g) was added. The precipitate was filtered and washed with water (100 ml). The filtrate was diluted with 10% aqueous sodium hydrogen carbonate solution (130 ml) followed by the addition of triethyl phosphonoacetate (57.8 g, 258 mmol, 2.0 equiv.) and a 6 M aqueous potassium carbonate solution (130 ml) at 5 °C. The mixture was stirred overnight at rt and extracted with $CH_2Cl_2$ (3× 200 ml). The combined organic layer was washed with water (1× 200 ml), brine (1× 200 ml), dried over $MgSO_4$ and evaporated to give a pale yellow oil. Flash column chromatography of the crude eluting with 5:2 pentane/ether, yielded **19a** (14.21 g, 70.96 mmol, 55% yield over three steps). The NMR spectra were in accordance with literature.[8]
The product's stereointegrity was analyzed by chiral HPLC, which unambiguously showed that no racemization had taken place during the oxidative cleavage and HWE sequence. The enantiomers could be separated on an OD-H column while eluting with an isocratic mobile phase consisting of heptane/isopropanol 98:2, giving **19a** at 11.6 min and **19b** at 16.6 min.

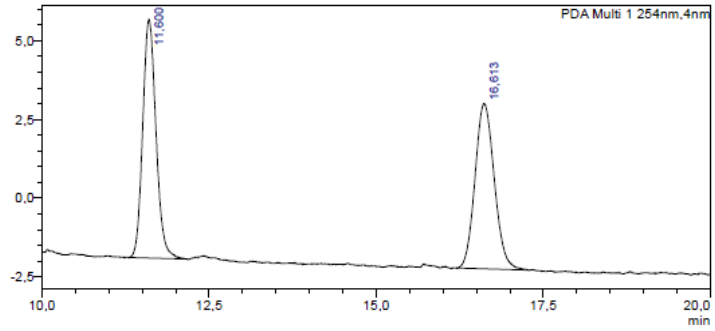


**<Peak Table>**

PDA Ch1 254nm

| Peak# | Ret. Time | Area | Height | Unit | Mark | Name | Area% |
|---|---|---|---|---|---|---|---|
| 1 | 11,600 | 104580 | 7565 | | M | | 49,853 |
| 2 | 16,613 | 105196 | 5240 | | M | | 50,147 |
| Total | | 209776 | 12806 | | | | 100,000 |

**Figure 3.** Chiral HPLC trace for racemic **19**.

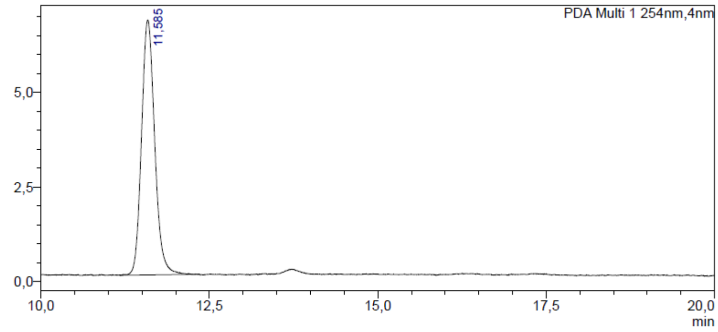


**<Peak Table>**

PDA Ch1 254nm

| Peak# | Ret. Time | Area | Height | Unit | Mark | Name | Area% |
|---|---|---|---|---|---|---|---|
| 1 | 11,585 | 91639 | 6725 | | M | | 100,000 |
| Total | | 91639 | 6725 | | | | 100,000 |

**Figure 4.** Chiral HPLC trace for **19a**, obtained from L-gulonolactone via a periodate cleavage/HWE sequence.

**Compound 19b:**

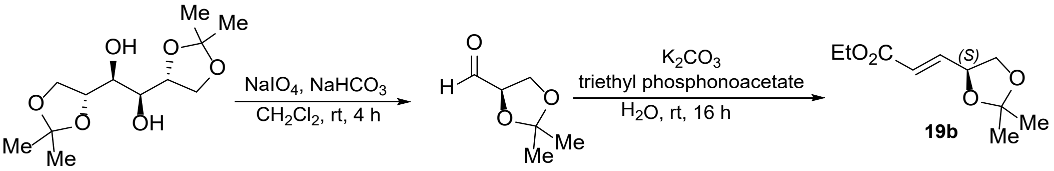


Compound **19b** was prepared following literature procedures for the oxidative cleavage[9] and the HWE[10] reaction. The product was obtained in similar yields and NMR spectra were in accordance with literature.

**Compound 20a:**

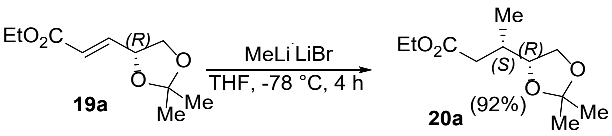


Compound **19a** (8.0 g, 40 mmol, 1.0 equiv.) was dissolved in dry $Et_2O$ (224 ml), and the solution was cooled to -78 °C. A solution of MeLi·LiBr (1.5 M, 40 ml, 1.5 equiv.) was added dropwise over 3 h. During the addition, the solution became turbid, but upon complete addition of MeLi a clear solution was obtained. The resulting yellow solution was stirred for 1 h at -78 °C. The reaction was quenched with EtOH (10 ml) and immediately poured into a 0 °C saturated aqueous $NH_4Cl$ solution (100 ml). The organic layer was separated, and the aqueous layer was extracted with $Et_2O$ (3× 100 ml). The combined organic layer was washed with brine (1× 200 ml) and concentrated *in vacuo*. The crude liquid residue was purified by flash chromatography using 12% $Et_2O$ in pentanes, yielding the product (7.99 g, 36.8 mmol, 92% yield) as a pale yellow oil.

$^1$**H-NMR** (400 MHz, $CDCl_3$) δ 4.09 (q, *J* = 7.2 Hz, 2H), 4.01 – 3.87 (m, 2H), 3.64 – 3.54 (m, 1H), 2.36 (dd, *J* = 14.6, 3.8 Hz, 1H), 2.21 – 2.04 (m, 2H), 1.36 (s, 3H), 1.30 (s, 3H), 1.22 (t, *J* = 7.1 Hz, 3H), 0.96 (d, *J* = 6.6 Hz, 3H). $^{13}$**C-NMR** (101 MHz, $CDCl_3$) δ 172.65, 108.95, 78.85, 66.81, 60.41, 37.62, 33.06, 26.42, 25.30, 15.44, 14.28.

**Compound 20b:**

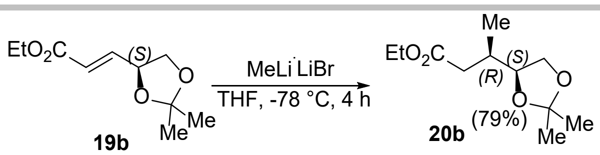


Compound **20b** (8.05 g, 37.2 mmol) could be obtained in 79% yield by subjecting **19b** (9.43 g, 47.1 mmol) to the aforementioned procedure.

**$^1$H-NMR** (400 MHz, $CDCl_3$) δ 4.14 (q, *J* = 6.9 Hz, 2H), 4.05 – 3.95 (m, 2H), 3.63 (app. t, *J* = 6.1 Hz, 1H), 2.40 (dd, *J* = 14.5, 3.7 Hz, 1H), 2.26 – 2.08 (m, 2H), 1.40 (s, 3H), 1.34 (s, 3H), 1.26 (t, *J* = 7.2 Hz, 3H), 1.00 (d, *J* = 6.4 Hz, 3H). **$^{13}$C-NMR** (101 MHz, $CDCl_3$) δ 172.58, 108.91, 78.83, 66.80, 60.36, 37.60, 33.04, 26.40, 25.28, 15.42, 14.26. Spectroscopic data were in agreement with literature.[11]

**Compound 21a:**

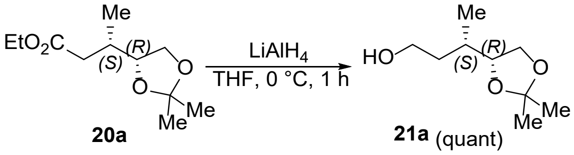


LiAlH$_4$ reduction:

Compound **20a** (8.16 g, 37.7 mmol, 1.0 equiv.) was dissolved in THF (400 ml). The solution was cooled to 0 °C using an ice-water bath. To this solution, $LiAlH_4$ (1.43 g, 37.7 mmol, 1.0 equiv.) was added in small portions over 5 min. The resulting suspension was stirred for 20 min until full conversion of the starting ester was evident by TLC. The reaction was quenched by careful addition of water (1.4 ml), followed by addition of 1 M $NaOH_{aq}$ (4.5 ml) and again water (4.5 ml). The suspension was stirred for 30 min at rt. $MgSO_4$ was added and the reaction was stirred for another 20 min. Then, the mixture was filtered and concentrated *in vacuo* using a bath temperature of 40 °C at 850 mbar to remove most of the solvent. Trace solvents were removed at 60 mbar for no longer than 10 min. The crude alcohol (6.58 g, 37.8 mmol, quantitative yield) was used as such in the next step.

**$^1$H-NMR** (400 MHz, $CDCl_3$) δ 4.05 – 3.95 (m, 2H), 3.78 – 3.71 (m, 1H), 3.69 – 3.61 (m, 2H), 1.93 – 1.77 (m, 2H), 1.70 – 1.60 (m, 1H), 1.48 – 1.38 (m, 4H), 1.35 (d, *J* = 0.8 Hz, 3H), 0.98 (d, *J* = 6.8 Hz, 3H). **$^{13}$C-NMR** (101 MHz, $CDCl_3$) δ 108.92, 79.79, 67.34, 60.58, 35.82, 32.93, 26.59, 25.49, 15.32. **HRMS (ESI)** Calcd. for $C_9H_{18}NaO_3$ ($[M + Na]^+$): 197.1148, found: 197.1149.

**Compound 22a:**

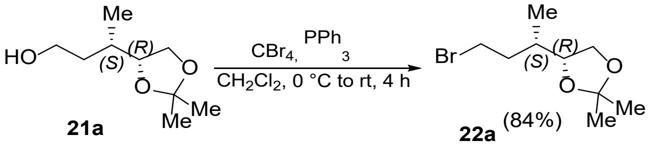


The crude alcohol **21a** (5.80 g, 33.3 mmol, 1.0 equiv.) was dissolved in $CH_2Cl_2$ (116 ml) and cooled to 0 °C using an ice-water bath. To this solution was added triphenylphosphine (10.48 g, 39.95 mmol, 1.2 equiv.) followed by $CBr_4$ (13.25 g, 39.95 mmol, 1.2 equiv.) in small portions over 10 min. After complete addition of the $CBr_4$, the ice-water bath was removed, and the reaction was allowed to stir at rt for 4 h. Then, the reaction mixture was passed through a short pad of silica in order to remove the triphenylphosphine oxide, and elution was continued with ether until TLC indicated complete recovery of the product. The solvent was carefully evaporated (water bath at 40 °C with a pressure not lower than 100 mbar), and purification of the residue by flash column chromatography in 4% ether in pentane afforded the product (6.71 g, 28.3 mmol, 84% yield) as a pale yellow oil.

**$^1$H-NMR** (400 MHz, $CDCl_3$) δ 4.08 – 3.92 (m, 2H), 3.64 (t, *J* = 6.3 Hz, 1H), 3.55 – 3.47 (m, 1H), 3.41 (app. q, *J* = 8.3 Hz, 1H), 1.99 – 1.81 (m, 2H), 1.76 – 1.65 (m, 1H), 1.41 (s, 3H), 1.34 (s, 3H), 0.97 (d, *J* = 6.7 Hz, 3H). **$^{13}$C-NMR** (101 MHz, $CDCl_3$) δ 108.54, 78.82, 66.97, 35.79, 34.55, 31.36, 26.30, 25.14, 14.17.

**Compound 22b:**

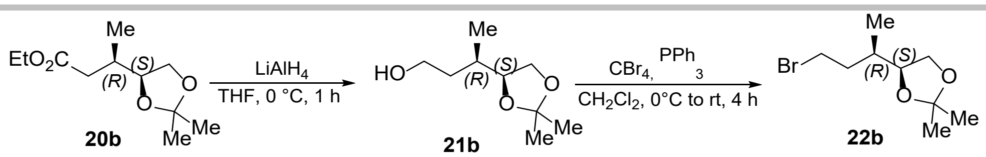


(93%, two steps)

Compound **22b** (8.19 g, 36.5 mmol) could be obtained in 93% yield over two steps by subjecting **20b** (8.48 g, 39.2 mmol) to the aforementioned procedure. During all evaporations at the bromide stage a minimum pressure of 100 mbar (water bath of 40 °C) was applied to remove solvent.

**$^{1}$H-NMR** (400 MHz, $CDCl_3$) δ 4.03 – 3.91 (m, 2H), 3.63 (t, *J* = 6.3 Hz, 1H), 3.54 – 3.47 (m, 1H), 3.44 – 3.36 (m, 1H), 1.97 – 1.79 (m, 2H), 1.74 – 1.64 (m, 1H), 1.39 (s, 3H), 1.33 (s, 3H), 0.95 (d, *J* = 6.6 Hz, 3H). **$^{13}$C-NMR** (101 MHz, $CDCl_3$) δ 108.94, 79.15, 77.48, 77.16, 76.84, 67.27, 36.01, 34.80, 31.68, 26.56, 25.40, 14.45.

**Compound 23a:**

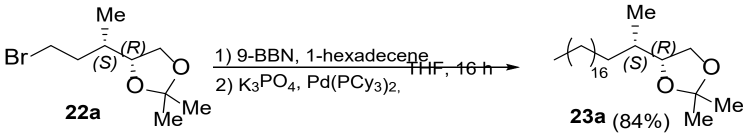


Hydroboration:
An oven dried Schlenk flask was charged with 1-hexadecene (8.89 g, 11.3 ml, 39.6 mmol, 1.4 equiv.) to which 9-BBN (0.5 M in THF, 79.1 ml, 39.6 mmol, 1.4 equiv.) was added at rt, and the reaction mixture was stirred at rt overnight. Next day, $^{1}$H-NMR analysis of the reaction mixture indicated that the conversion of the alkene was around 90%. Therefore, additional 9-BBN solution (12 ml, 6.0 mmol, 0.15 equiv.) was added to the reaction mixture. After 5 h the conversion of the starting alkene was >98% according to $^{1}$H-NMR. This solution was used without further manipulation in the next step.

Cross-coupling:
An oven dried three necked roundbottom flask was charged with $Pd(PCy_3)_2$ (0.42 g, 0.63 mmol, 2.2 mol%) and $K_3PO_4 \cdot H_2O$ (9.11 g, 39.6 mmol, 1.4 equiv.). Any possibly leached oxygen was removed by applying 3 nitrogen/vacuum cycles. To the flask was added the complete previously formed alkyl borane solution. The suspension turned dark orange/yellow within few seconds and was allowed to stir for 20 min at rt. To this suspension bromide **22a** (6.7 g, 28.3 mmol, 1.0 equiv.) was added and the reaction mixture was vigorously stirred for 6 days at rt, after which TLC (2% $Et_2O$ in pentane) indicated that the bromide was completely consumed. Celite was added (ca 25 g) to the crude reaction mixture and volatiles were removed *in vacuo*. The residue was purified by flash column chromatography using a 0-3% ether in pentane gradient, yielding the product (9.06 g, 23.7 mmol, 84% yield) as a colorless oil that solidified upon standing.

**$^{1}$H-NMR** (400 MHz, $CDCl_3$) δ 4.00 (app. t, *J* = 7.1 Hz, 1H), 3.86 (q, *J* = 6.8 Hz, 1H), 3.60 (t, *J* = 7.6 Hz, 1H), 1.60 – 1.50 (m, 1H), 1.43 – 1.01 (m, 40H), 0.96 (d, *J* = 6.6 Hz, 3H), 0.88 (t, *J* = 6.6 Hz, 3H). **$^{13}$C-NMR** (101 MHz, $CDCl_3$) δ 108.59, 80.52, 67.95, 36.67, 32.91, 32.09, 30.03, 29.87, 29.85, 29.83, 29.81, 29.78, 29.53, 27.15, 26.76, 25.66, 22.84, 15.74, 14.24.

**Compound 23b:**

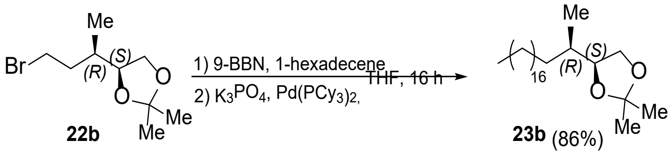


Compound **23b** (9.74 g, 25.4 mmol) could be obtained in 86% yield by subjecting **22b** (7.01 g, 29.6 mmol) to the aforementioned procedure. In the cross-coupling reaction a 5 mol% palladium loading was used, which resulted in complete consumption of the bromide in 2 days.

**$^{1}$H-NMR** (400 MHz, $CDCl_3$) δ 4.00 (dd, *J* = 7.8, 6.2 Hz, 1H), 3.86 (q, *J* = 7.1 Hz, 1H), 3.59 (t, *J* = 7.7 Hz, 1H), 1.59 – 1.50 (m, 1H), 1.44 – 1.01 (m, 40H), 0.96 (d, *J* = 6.7 Hz, 3H), 0.88 (t, *J* = 6.7 Hz, 3H). **$^{13}$C-NMR** (101 MHz, $CDCl_3$) δ 108.63, 80.55, 67.97, 36.66, 32.87, 32.09, 30.03, 29.86, 29.85, 29.83, 29.82, 29.81, 29.78, 29.53, 27.14, 26.78, 25.70, 22.85, 15.78, 14.29.

**Compound 25a:**

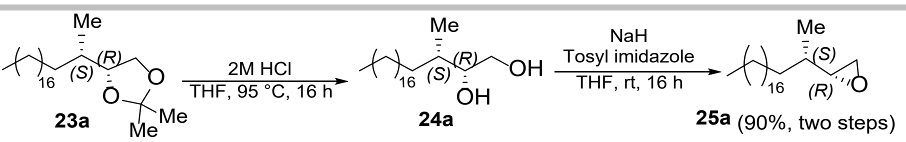


Deprotection:
Acetonide **23a** (5.00 g, 13.1 mmol, 1.0 equiv.) was dissolved in THF (130 ml) and $HCl_{aq}$ (2 M, 65 ml, 130 mmol, 10 equiv.) was added. The resulting two-phase mixture was immersed into a preheated oil bath of 95 °C and stirred vigorously at reflux overnight. Next day, TLC (80% ether in pentane) indicated full conversion of the acetonide, after which the reaction mixture was cooled down to rt and the THF was evaporated. The aqueous residue was extracted with ether (3× 100 ml). The combined organic layer was washed with saturated aqueous $NaHCO_3$ solution (1× 100 ml), brine (1× 100 ml), dried over $MgSO_4$ and concentrated *in vacuo*, yielding the product (5.37 g, 15.7 mmol) as a colorless oil that solidified upon standing. The product was used in the next step without additional purification.

Epoxide formation:
A Schlenk flask was charged with NaH (60% dispersion in mineral oil, 1.51 g, 37.6 mmol, 2.87 equiv.), and the NaH was washed with pentane (3x 25 ml) in order to remove the oil. The NaH was suspended in THF (60 ml) and cooled to 0 °C using an ice-water bath. Diol **24a** from the previous step (5.37 g) was added carefully as a solution in THF (60 ml), and the resulting thick suspension was allowed to stir at 0 °C for an additional 2 h. Tosyl imidazole (4.18 g, 18.8 mmol, 1.44 equiv.) was added and the resulting suspension was stirred at rt for 18 h. The reaction mixture was filtered over a glass filter and the filter cake was washed with $Et_2O$ (3× 100 ml). The combined filtrate was evaporated with 15 g of celite and purified by flash column chromatography using 1-3% ether in pentane, yielding the product (3.80 g, 11.7 mmol, 90% yield over two steps) as a colorless oil that solidified upon standing.

**$^1$H-NMR** (400 MHz, $CDCl_3$) δ 2.74 (t, *J* = 4.5 Hz, 1H), 2.70 – 2.62 (m, 1H), 2.51 (dd, *J* = 5.1, 2.8 Hz, 1H), 1.40 – 1.19 (m, 35H), 1.01 (d, *J* = 6.0 Hz, 3H), 0.87 (t, *J* = 6.6 Hz, 3H). **$^{13}$C-NMR** (101 MHz, $CDCl_3$) δ 57.24, 47.09, 36.38, 33.74, 32.08, 30.04, 29.86, 29.83, 29.82, 29.80, 29.75, 29.52, 27.28, 22.84, 17.26, 14.24. **HRMS (ESI)** Calcd. for $C_{22}H_{45}O$ ($[M + H]^+$): 325.3465, found: 325.3461.

**Compound 25b:**

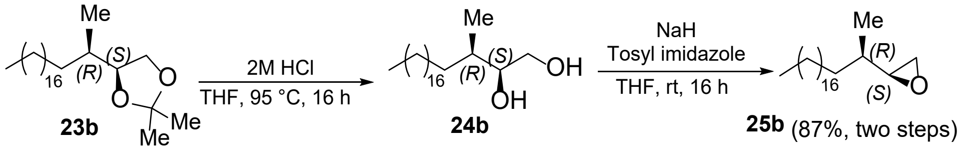


Compound **24b** could be obtained by subjecting acetonide **23b** (9.74 g, 28.5 mmol) to the aforementioned deprotection procedure. Unlike **24a**, crude **24b** was purified by recrystallization in 1:1 pentane/ether, affording analytically pure material (6.75 g, 19.7 mmol, 69% yield). The mother liquor was purified by flash column chromatography using a 50-100% ether in pentane gradient to afford additional pure **25b** (2.01 g, 5.87 mmol, 21% yield.), resulting in a total yield of 90%. Compound **24b** (6.75 g, 19.7 mmol) was converted into the epoxide **25b** (6.18g, 19.0 mmol, 97%, 87% over two steps) using the aforementioned procedure.
**$^1$H-NMR** (400 MHz, $CDCl_3$) δ 2.75 (dd, *J* = 5.0, 3.9 Hz, 1H), 2.70 – 2.65 (m, 1H), 2.52 (dd, *J* = 5.0, 2.8 Hz, 1H), 1.42 – 1.18 (m, 35H), 1.02 (d, *J* = 6.2 Hz, 3H), 0.88 (t, *J* = 6.9 Hz, 3H). **$^{13}$C-NMR** (101 MHz, $CDCl_3$) δ 57.30, 47.15, 36.40, 33.74, 32.09, 30.04, 29.86, 29.83, 29.82, 29.80, 29.75, 29.52, 27.29, 22.85, 17.28, 14.27. **HRMS (ESI)** Calcd. for $C_{22}H_{45}O$ ($[M + H]^+$): 325.3465; found: 325.3461

**Compound 26:**

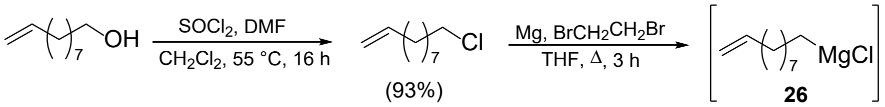


Chloride formation:
The alcohol (12.7 g, 15.0 ml, 81.4 mmol, 1.0 equiv.) was dissolved in $CH_2Cl_2$ (81 ml), followed by the addition of DMF (0.94 ml, 12.2 mmol, 0.15 equiv.) and thionyl chloride (19.4 g, 163 mmol, 11.8 ml, 2.0 equiv.). The resulting solution was heated at 55°C. The outlet of the condenser was directed to a base trap in order to quench the formed HCl. After 4 h, TLC indicated incomplete conversion of the alcohol. Therefore, an additional amount of thionyl chloride (6.36 g, 7.5 ml, 40.7 mmol, 1.0 equiv.) and DMF (0.94 ml, 12.2 mmol, 0.15 equiv.) were added and stirring was continued at 55 °C overnight. Excess thionyl chloride was quenched very carefully with water (200 ml) while cooling with an ice-water bath to 0 °C. The mixture was transferred to a separatory funnel and extracted with $CH_2Cl_2$ (3× 100 ml). The combined organic layer was washed with 2 M $NaOH_{aq}$ (1× 150 ml), water (1× 150 ml), brine (1× 150 ml), dried over $MgSO_4$ and concentrated *in vacuo*. The residue was purified by flash column chromatography in 100% pentane, yielding the product (13.3 g, 75.9 mmol, 93% yield) as a colorless oil.

**$^1$H-NMR** (400 MHz, $CDCl_3$) δ 5.81 (ddt, *J* = 16.9, 10.2, 6.7 Hz, 1H), 5.04 – 4.89 (m, 2H), 3.53 (t, *J* = 6.8 Hz, 2H), 2.08 – 2.00 (m, 2H), 1.82 – 1.71 (m, 2H), 1.48 – 1.25 (m, 10H). **$^{13}$C-NMR** (101 MHz, $CDCl_3$) δ 139.19, 114.29, 45.22, 33.90, 32.78, 29.43, 29.14, 29.01, 28.97, 27.00.

Formation of Grignard reagent **26**:

Magnesium (1.39 g, 57.2 mmol, 1.9 equiv.) was ground in a mortar, in an attempt to get most of the magnesium as shiny as possible, and transferred to a dry flask under a nitrogen atmosphere. A small crystal of iodine was added, and the flask was heated while stirring the magnesium using a stirring bar. After the purple vapor reduced significantly in intensity, the magnesium was allowed to cool to rt and was subsequently suspended in THF (15 ml). Dibromoethane (50 µl) was added, and after gas evolution ceased, the reaction mixture was brought to a gentle reflux. The alkyl chloride (5.21 g, 28.6 mmol, 1.0 equiv.) was added dropwise via syringe, and after complete addition, heating at reflux was continued for an additional 2 h. The dark turbid reaction mixture was allowed to cool down to rt, and diluted with additional THF (35 ml). A small sample of the Grignard reagent was titrated against a 1 M menthol solution (in THF) in the presence of a trace amount of phenanthroline as indicator, which confirmed a ± 0.6 M concentration of the Grignard solution. The formed Grignard solution was used within 24 h in the CuCl catalyzed epoxide opening reaction without further manipulation.
Note: in a later experiment the Grignard reagent was diluted with THF after metalation to result in 0.1 M solutions of the Grignard reagent. There was no difference in yield (in epoxide opening) between applying 0.6 M or 0.1 M solutions.

**Compound 27a:**

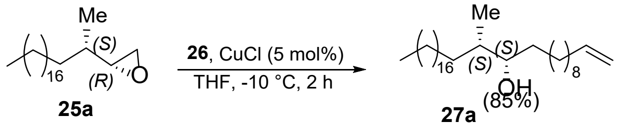


Freshly prepared grey solution of **26** in THF (1 M, 50 ml, 50 mmol, 1.9 equiv.) was cooled to -10 °C using an ice/brine bath. To this solution, anhydrous CuCl (174 mg, 1.76 mmol, 6.8 mol%) was added. The color of the solution changed from grey to brown. Then, a solution of epoxide **25a** (8.34 g, 25.7 mmol, 1.0 equiv.) in THF (70 ml) was added over 2.5 h. After complete addition, the brown reaction mixture was stirred for 30 additional min at -10 °C, before TLC (3% ether in pentane) showed full conversion of the epoxide. The reaction was quenched by addition of MeOH (12 ml) followed by $HCl_{aq}$ (2 M, 22 ml) at -10 °C. The mixture was stirred for 30 min at rt and $HCl_{aq}$ (2 M) was added until the mixture became completely homogeneous. The mixture was extracted with $Et_2O$ (3× 200 ml), and the combined organic layer was washed with brine (1× 200 ml), dried over $MgSO_4$ and concentrated *in vacuo*. The crude product was purified by flash column chromatography in a 1-2% ether in pentane gradient, yielding the product (10.1 g, 21.8 mmol, 85% yield) as a colorless oil that solidified upon standing.

**$^1$H-NMR** (400 MHz, $CDCl_3$) δ 5.81 (ddt, *J* = 16.9, 10.2, 6.7 Hz, 1H), 4.99 (app. d, *J* = 17.2 Hz, 1H), 4.93 (app. d, *J* = 10.2 Hz, 1H), 3.54 – 3.45 (m, 1H), 2.04 (q, *J* = 7.0 Hz, 2H), 1.27 (d, *J* = 9.8 Hz, 52H), 0.92 – 0.82 (m, 6H). **$^{13}$C-NMR** (101 MHz, $CDCl_3$) δ 139.40, 114.24, 75.36, 38.33, 34.65, 33.97, 33.53, 32.09, 30.12, 29.90, 29.86, 29.84, 29.82, 29.79, 29.72, 29.64, 29.52, 29.30, 29.10, 27.58, 26.44, 22.85, 14.28, 13.74. **HRMS (ESI)** Calcd. for $C_{32}H_{63}O$ ($[M - H]^-$): 463.4873; found: 463.4870.

**Compound 27b:**

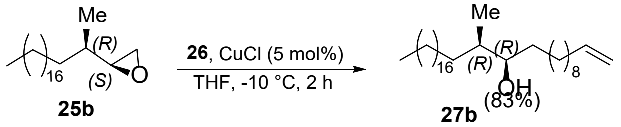


Compound **27b** (7.33 g, 15.78 mmol) could be obtained in 83% yield after column purification by subjecting epoxide **25b** (5.67 g, 28.5 mmol) to the aforementioned procedure.

**$^1$H-NMR** (400 MHz, $CDCl_3$) 5.81 (ddt, *J* = 16.9, 10.2, 6.7 Hz, 1H), 4.99 (dq, *J* = 17.1, 1.7 Hz, 1H), 4.93 (ddt, *J* = 10.2, 2.3, 1.2 Hz, 1H), 3.54 – 3.44 (m, 1H), 2.09 – 1.99 (m, 2H), 1.52 – 1.11 (m, 52H), 0.91 – 0.83 (m, 6H). **$^{13}$C-NMR** (101 MHz, $CDCl_3$) δ 139.28, 114.22, 75.29, 38.34, 34.66, 33.97, 33.52, 32.09, 30.13, 29.91, 29.87, 29.85, 29.83, 29.80, 29.73, 29.65, 29.53, 29.30, 29.10, 27.58, 26.45, 22.85, 14.25, 13.72. **HRMS (ESI)** Calcd. for $C_{32}H_{64}NaO$ ($[M + Na]^+$): 487.4849; found: 463.4842.

***S,S*-fragment C:**

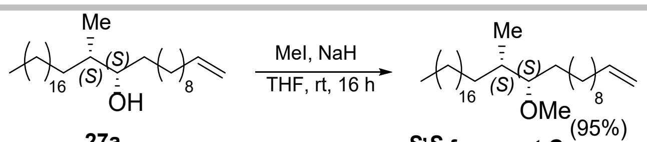


**27a** **S'S-fragment C** (95%)

A dry Schlenk flask was charged with NaH (60% in mineral oil, 1.64 g, 41.1 mmol, 2.5 equiv.). Washing turned out essential since the product and the mineral oil are not separable by column chromatography. The mineral oil was removed by 3 successive washings with pentane (25 ml). The grey residue was suspended in THF (10 ml) and a solution of alcohol **27a** (7.64 g, 16.5 mmol, 1.0 equiv.) in THF (50 ml) was added dropwise over 30 min. When the gas evolution ceased, MeI (7.00 g, 3.10 ml, 49.4 mmol 3.0 equiv.) was added in one portion. The reaction mixture was stirred at rt overnight, after which TLC indicated complete conversion of the starting material. The reaction mixture was diluted with $Et_2O$ (200 ml) and quenched by addition of MeOH (20 ml). As soon as all NaH was quenched, the reaction mixture turned into a clear colorless solution. The resulting solution was poured into $HCl_{aq}$ (1 M, 60 ml). The organic layer was separated and the aqueous layer was extracted with $Et_2O$ (3× 100 ml). The combined organic layer was washed with aqueous $Na_2SO_3$ (0.1 M, 1× 250 ml), brine (1× 200 ml), dried over $MgSO_4$ and concentrated *in vacuo*. The crude product was purified with flash column chromatography applying a 1-1.5% ether in pentane gradient, yielding the product (7.48 g, 15.6 mmol, 95% yield) as a colorless oil that solidified upon standing.

**$^1$H-NMR** (400 MHz, $CDCl_3$) δ 5.81 (ddt, *J* = 16.9, 10.2, 6.6 Hz, 1H), 4.99 (app. d, *J* = 17.0 Hz, 1H), 4.93 (app. d, *J* = 10.2 Hz, 1H), 3.34 (s, 3H), 3.00 – 2.91 (m, 1H), 2.04 (q, *J* = 7.1 Hz, 2H), 1.68 – 1.56 (m, 1H), 1.50 – 0.99 (m, 50H), 0.88 (t, *J* = 6.7 Hz, 3H), 0.85 (d, *J* = 6.8 Hz, 3H). **$^{13}$C-NMR** (101 MHz, $CDCl_3$) δ 139.25, 114.23, 114.23, 85.57, 77.48, 77.16, 76.84, 57.81, 35.51, 33.99, 32.54, 32.12, 30.65, 30.17, 30.11, 29.90, 29.88, 29.86, 29.84, 29.77, 29.68, 29.56, 29.33, 29.13, 27.76, 26.33, 22.87, 15.03, 14.26. **HRMS (ESI)** Calcd. for $C_{33}H_{67}O$ ($[M + H]^+$): 479.5186; found: 479.5181.

***R*,*R*-fragment C:**

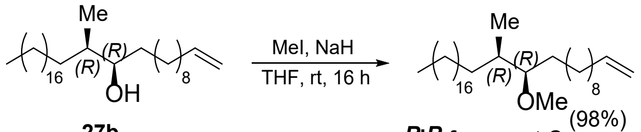


**27b** **R'R-fragment C** (98%)

***R*,*R*-fragment C** (0.72 g, 1.50 g) could be obtained in 98% yield after column purification by subjecting alcohol **27b** (717 mg, 1.54 mmol) to the aforementioned procedure.

**$^1$H-NMR** (400 MHz, $CDCl_3$) δ 5.81 (ddt, *J* = 16.9, 10.2, 6.7 Hz, 1H), 4.99 (dq, *J* = 17.1, 1.7 Hz, 1H), 4.92 (ddt, *J* = 10.2, 2.3, 1.3 Hz, 1H), 3.34 (s, 3H), 3.00 – 2.91 (m, 1H), 2.09 – 1.98 (m, 2H), 1.69 – 1.56 (m, 1H), 1.49 – 0.99 (m, 50H), 0.88 (t, *J* = 6.6 Hz, 3H), 0.85 (d, *J* = 6.8 Hz, 4H). **$^{13}$C-NMR** (101 MHz, $CDCl_3$) δ 139.25, 114.23, 85.57, 57.81, 35.51, 34.00, 32.54, 32.12, 30.65, 30.17, 30.11, 29.90, 29.88, 29.86, 29.84, 29.77, 29.68, 29.56, 29.33, 29.13, 27.77, 26.33, 22.87, 15.04, 14.26. **HRMS (APCI)** Calcd. for $C_{33}H_{67}O$ ($[M + H]^+$): 479.5187; found: 479.5180.

**Endgame:**

**Compound 28a:**

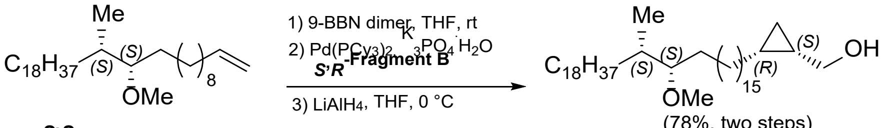


**S'S-Fragment C** **28a** (78%, two steps)

Hydroboration:

***S*,*S*-Fragment C** (2.82 g, 5.90 mmol, 1.2 equiv.) was dissolved in anhydrous THF (9 ml) and solid 9-BBN dimer (780 mg, 3.19 mmol, 0.65 equiv.) was added in one portion. The resulting solution was stirred at rt for 3.5 h, after which $^1$H-NMR showed complete disappearance of the olefinic signals.

Cross-coupling:

To the hydroboration solution was added $Pd(PCy_3)_2$ (225 mg, 0.34 mmol 6.9 mol%), and $K_3PO_4·H_2O$ (1.69 g, 7.37 mmol, 1.5 equiv.) and stirring was continued for 15 min. Then, ***S*,*R*-fragment B** (1.50 g, 4.91 mmol, 1.0 equiv.) was added, and THF (3 ml) was used to rinse the syringe. The reaction mixture was vigorously stirred at rt overnight. $^1$H-NMR indicated full consumption of the bromide and the reaction mixture was coated on celite and purified by flash column chromatography

using a 1-4% ether in pentane gradient, yielding a dark black oil. The obtained (impure) product was used as such in the next step.

*Note: The bromide and the product coelute on TLC, and therefore $^{1}$H-NMR is essential to determine the conversion. Although TLC showed easy separation of the product, upon purification a less polar side-product coeluted. All fractions containing the product were combined and used as such in the next step.*

LiAlH$_4$ reduction:
To a chilled solution at 0 °C of the pivaloyl ester in THF (25 ml) was added dropwise a solution of LiAlH$_4$ (1 M in THF, 4.4 ml, 4.4 mmol, 1.1 equiv.). After 15 min TLC (5% ether in pentane) showed complete consumption of the ester. The reaction mixture was diluted with ether (30 ml), and water (0.18 ml), NaOH$_{aq}$ (15%, 0.18 ml) and water (0.50 ml) were carefully added. The reaction mixture was then stirred for 15 min at rt, followed by the addition of enough MgSO$_4$ in order to remove the excess water. After stirring for 15 min, the suspension was filtered and the filtrate was concentrated *in vacuo*, yielding the crude as a brown oil. Flash column chromatography using a 10-15% EtOAc in pentane gradient yielded the product (2.38 g, 3.84 mmol, 78% over two steps) as a yellow oil.

**$^{1}$H-NMR** (400 MHz, CDCl$_3$) δ 3.65 (dd, *J* = 11.3, 7.1 Hz, 1H), 3.58 (dd, *J* = 11.3, 8.1 Hz, 1H), 3.34 (s, 3H), 3.00 – 2.91 (m, 1H), 1.67 – 1.58 (m, 1H), 1.57 – 1.03 (m, 67H), 0.94 – 0.79 (m, 8H), 0.70 (td, *J* = 8.3, 4.4 Hz, 1H), -0.04 (q, *J* = 5.3 Hz, 1H). **$^{13}$C-NMR** (101 MHz, CDCl$_3$) δ 85.56, 63.28, 57.76, 35.46, 32.49, 32.06, 30.60, 30.30, 30.11, 30.07, 29.84, 29.82, 29.80, 29.71, 29.50, 28.70, 27.70, 26.27, 22.81, 18.24, 16.26, 14.98, 14.22, 9.59. **HRMS (ESI)** Calcd. for $C_{42}H_{84}NaO_2$ ([M + Na]$^+$): 643.6364, found: 643.6343.

**Compound 28b:**

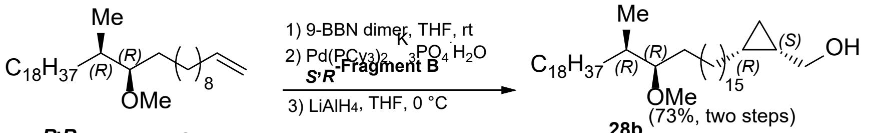


***R,R*-Fragment C** **28b** (73%, two steps)

Compound **28b** (2.23 g, 3.60 mmol) was obtained in a 73% isolated yield by subjecting ***R,R*-Fragment C** (2.82 g, 5.90 mmol, 1.2 equiv.) and ***S,R*-Fragment B** (1.50 g, 4.91 mmol, 1.0 equiv.) to the aforementioned procedures.

**$^{1}$H-NMR** (400 MHz, CDCl$_3$) δ 3.65 (dd, *J* = 11.3, 7.1 Hz, 1H), 3.58 (dd, *J* = 11.3, 8.0 Hz, 1H), 3.34 (s, 3H), 3.01 – 2.90 (m, 1H), 1.68 – 1.57 (m, 1H), 1.57 – 1.01 (m, 67H), 0.95 – 0.78 (m, 8H), 0.70 (td, *J* = 8.3, 4.5 Hz, 1H), -0.04 (q, *J* = 5.2 Hz, 1H). **$^{13}$C-NMR** (101 MHz, CDCl$_3$) δ 85.59, 63.43, 57.82, 35.48, 32.51, 32.08, 30.63, 30.33, 30.13, 30.09, 29.85, 29.81, 29.73, 29.52, 28.72, 27.72, 26.31, 22.84, 18.30, 16.30, 15.02, 14.25, 9.62. **HRMS (ESI)** Calcd. for $C_{42}H_{84}NaO_2$ ([M + Na]$^+$): 643.6364, found: 643.6340.

**Compound 28c:**

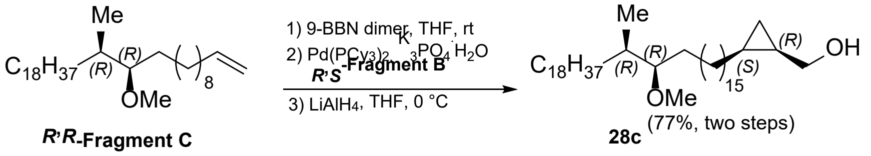


***R,R*-Fragment C** **28c** (77%, two steps)

Compound **28c** (2.23 g, 3.60 mmol) was obtained in a 77% isolated yield by subjecting ***R,R*-Fragment C** (2.59 g, 5.41 mmol, 1.2 equiv.) and ***R,S*-Fragment B** (1.42 g, 4.65 mmol, 1.0 equiv.) to the aforementioned procedures.

**$^{1}$H-NMR** (400 MHz, CDCl$_3$) δ 3.69 – 3.61 (m, 1H), 3.61 – 3.54 (m, 1H), 3.34 (s, 3H), 3.00 – 2.91 (m, 1H), 1.68 – 1.56 (m, 1H), 1.50 – 1.01 (m, 67H), 0.94 – 0.79 (m, 8H), 0.70 (td, *J* = 8.3, 4.5 Hz, 1H), -0.04 (q, *J* = 5.3 Hz, 1H). **$^{13}$C-NMR** (101 MHz, CDCl$_3$) δ 85.58, 63.36, 57.79, 35.48, 32.51, 32.07, 30.63, 30.32, 30.12, 30.08, 29.85, 29.80, 29.72, 29.51, 28.71, 27.71, 26.29, 22.82, 18.28, 16.29, 15.01, 14.23, 9.61. **HRMS (ESI)** Calcd. for $C_{42}H_{84}NaO_2$ ([M + Na]$^+$): 643.6364, found: 643.6342.

**Compound 28d:**

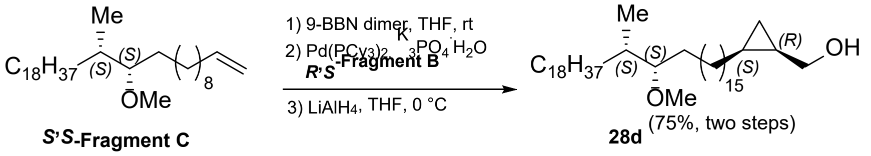


***S,S*-Fragment C** **28d** (75%, two steps)

Compound **28d** (2.19 g, 3.52 mmol) was obtained in a 75% isolated yield by subjecting ***S,S*-Fragment C** (2.70 g, 5.65 mmol, 1.2 equiv.) and ***R,S*-Fragment B** (1.44 g, 4.71 mmol, 1.0 equiv.) to the aforementioned procedures.

**$^1$H-NMR** (400 MHz, $CDCl_3$) δ 3.70 – 3.62 (m, 1H), 3.61 – 3.54 (m, 1H), 3.34 (s, 3H), 3.02 – 2.89 (m, 1H), 1.69 – 1.57 (m, 1H), 1.51 – 1.00 (m, 67H), 0.94 – 0.78 (m, 8H), 0.70 (td, *J* = 8.3, 4.5 Hz, 1H), -0.04 (q, *J* = 5.2 Hz, 1H). **$^{13}$C-NMR** (101 MHz, $CDCl_3$) δ 85.58, 63.40, 57.81, 35.47, 32.51, 32.08, 30.62, 30.32, 30.13, 30.09, 29.85, 29.81, 29.73, 29.52, 28.71, 27.72, 26.30, 22.83, 18.29, 16.30, 15.02, 14.25, 9.61. **HRMS (ESI)** Calcd. for $C_{42}H_{84}NaO_2$ ([M + Na]$^+$): 643.6364, found: 643.6345.

**Compound 35a:**

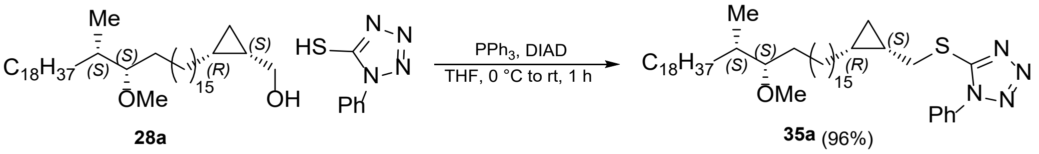


**28a** **35a** (96%)

A solution of alcohol **28a** (2.35 g, 3.79 mmol, 1.0 equiv.) in THF (10 ml) was cooled to 0 °C, followed by the addition of $PPh_3$ (1.49 g, 5.69 mmol, 1.5 equiv.) and 1-phenyl-1*H*-tetrazole-5-thiol (878 mg, 4.93 mmol, 1.3 equiv.). Then, DIAD (970 µl, 4.93 mmol, 1.3 equiv.) was added dropwise and the resulting orange mixture was stirred for 45 min at rt. After TLC (10% EtOAc in pentane) indicated full conversion, the reaction mixture was poured in water (50 ml) and extracted with ether (3× 50 ml). The combined organic layer was washed with brine (1× 50 ml), dried over $MgSO_4$ and evaporated *in vacuo*. The crude residue was purified by flash column chromatography using a 5-10% ether in pentane gradient, yielding the product (2.86 g, 3.66 mmol, 96%) as a colorless oil that solidified upon standing.

**$^1$H-NMR** (400 MHz, $CDCl_3$) δ 7.67 – 7.48 (m, 5H), 3.48 (d, *J* = 7.9 Hz, 2H), 3.34 (s, 3H), 2.99 – 2.91 (m, 1H), 1.68 – 1.58 (m, 1H), 1.53 – 1.01 (m, 66H), 1.00 – 0.78 (m, 9H), 0.07 (q, *J* = 5.3 Hz, 1H). **$^{13}$C-NMR** (101 MHz, $CDCl_3$) δ 154.55, 133.90, 129.97, 129.75, 123.76, 85.43, 57.71, 35.38, 35.06, 32.43, 32.00, 30.54, 30.09, 30.05, 30.02, 29.78, 29.76, 29.74, 29.61, 29.44, 28.52, 27.64, 26.22, 22.75, 18.02, 14.95, 14.71, 14.18, 12.51. **HRMS (ESI)** Calcd. for $C_{49}H_{89}N_4OS$ ([M + H]$^+$): 781.6752, found: 781.6732.

**Compound 35b:**

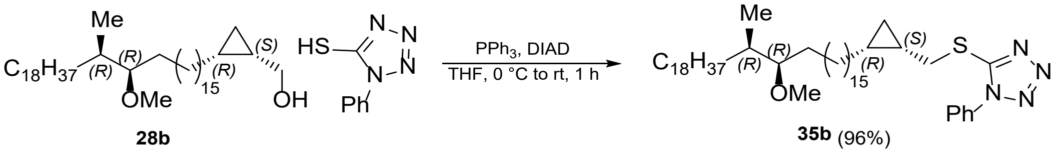


**28b** **35b** (96%)

Compound **35b** (2.68 g, 3.43 mmol) was obtained in a 96% isolated yield by subjecting alcohol **28b** (2.21 g, 3.56 mmol) to the aforementioned procedure.

**$^1$H-NMR** (400 MHz, $CDCl_3$) δ 7.65 – 7.49 (m, 5H), 3.48 (d, *J* = 7.9 Hz, 2H), 3.34 (s, 3H), 2.99 – 2.91 (m, 1H), 1.67 – 1.58 (m, 1H), 1.52 – 1.03 (m, 66H), 1.00 – 0.78 (m, 9H), 0.07 (q, *J* = 5.3 Hz, 1H). **$^{13}$C-NMR** (101 MHz, $CDCl_3$) δ 154.66, 133.94, 130.05, 129.82, 123.85, 85.50, 57.78, 35.43, 35.12, 32.47, 32.03, 30.59, 30.13, 30.09, 30.05, 29.82, 29.80, 29.77, 29.65, 29.48, 28.57, 27.68, 26.27, 22.80, 18.07, 14.99, 14.74, 14.22, 12.57. **HRMS (ESI)** Calcd. for $C_{49}H_{89}N_4OS$ ([M + H]$^+$): 781.6752, found: 781.6737.

**Compound 35c:**

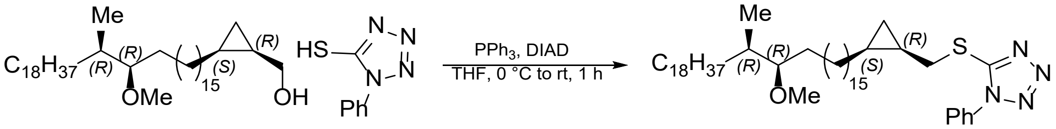


**28c** **35c** (93%)

Compound **35c** (2.56 g, 3.28 mmol) was obtained in a 93% isolated yield by subjecting alcohol **28c** (2.20 g, 3.54 mmol) to the aforementioned procedure.

**$^1$H-NMR** (400 MHz, $CDCl_3$) δ 7.64 – 7.50 (m, 5H), 3.48 (d, *J* = 7.9 Hz, 2H), 3.33 (s, 3H), 3.00 – 2.91 (m, 1H), 1.67 – 1.58 (m, 1H), 1.54 – 1.01 (m, 66H), 0.98 – 0.78 (m, 9H), 0.07 (q, *J* = 5.3 Hz, 1H). **$^{13}$C-NMR** (101 MHz, $CDCl_3$) δ 154.68, 133.96, 130.07, 129.83, 123.88, 85.53, 57.80, 35.46, 35.14, 32.49, 32.04, 30.61, 30.14, 30.10, 30.06, 29.82, 29.80, 29.78, 29.66, 29.48, 28.58, 27.69, 26.28, 22.81, 15.01, 14.77, 14.23, 12.58. **HRMS (ESI)** Calcd. for $C_{49}H_{89}N_4OS$ ([M + H]$^+$): 781.6752, found: 781.6737.

**Compound 35d:**

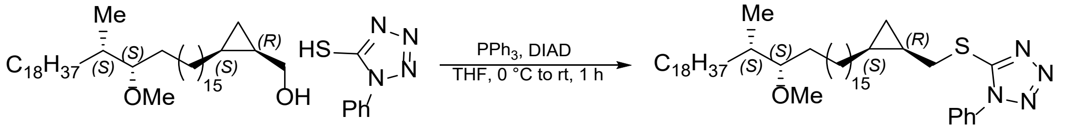

**28d** **35d** (96%)

Compound **35d** (2.62 g, 3.35 mmol) was obtained in a 96% isolated yield by subjecting alcohol **28d** (2.18 g, 3.51 mmol) to the aforementioned procedure.
**$^{1}$H-NMR** (400 MHz, $CDCl_3$) δ 7.70 – 7.45 (m, 5H), 3.48 (d, *J* = 7.9 Hz, 2H), 3.34 (s, 3H), 3.00 – 2.88 (m, 1H), 1.68 – 1.56 (m, 1H), 1.52 – 1.02 (m, 66H), 0.99 – 0.78 (m, 9H), 0.07 (q, *J* = 5.3 Hz, 1H). **$^{13}$C-NMR** (101 MHz, $CDCl_3$) δ 154.49, 133.88, 129.92, 129.72, 123.71, 85.38, 57.67, 35.36, 35.02, 35.02, 32.41, 31.97, 30.51, 30.07, 30.03, 29.99, 29.76, 29.74, 29.72, 29.59, 28.50, 27.62, 26.19, 22.73, 17.99, 14.92, 14.69, 14.15, 12.48. **HRMS (ESI)** Calcd. for $C_{49}H_{89}N_4OS$ ($[M + H]^+$): 781.6752, found: 781.6737.

**Compound 29a:**

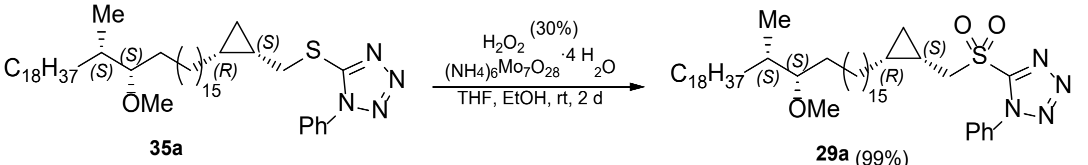

**35a** **29a** (99%)

To a solution of **35a** (2.70 g, 3.57 mmol, 1.0 equiv.) in EtOH (40 ml) and THF (30 ml) was added ammonium molybdate (1.28 g, 1.04 mmol, 30 mol%) followed by $H_2O_2$ (30% aqueous solution, 8.2 ml, 86.39 mmol, 25 equiv). The resulting mixture was stirred overnight at rt. $^{1}$H-NMR showed complete consumption of the sulfide, resulting in a 5:1 sulfone/sulfoxide mixture. More catalyst (650 mg, 0.53 mmol, 15 mol%) and peroxide (4.5 ml, 47.51 mmol, 14 equiv.) were added and the reaction mixture was allowed to stir for another day. Then, the volatiles were evaporated and the residue was diluted with water (100 ml) and extracted with ether (3× 75 ml). The combined organic layer was washed with a saturated aqueous solution of $Na_2SO_3$ (1× 100 ml), water (1× 100 ml) brine (1× 100 ml), dried over $MgSO_4$ and evaporated *in vacuo*. The crude residue was purified by flash column chromatograph using 10% ether in pentane, yielding the product (2.77 g, 3.41 mmol, 99%) as a colorless oil that solidified upon standing.

**$^{1}$H-NMR** (400 MHz, $CDCl_3$) δ 7.72 – 7.67 (m, 2H), 7.64 – 7.57 (m, 3H), 3.96 (dd, *J* = 14.7, 5.6 Hz, 1H), 3.56 (dd, *J* = 14.7, 9.3 Hz, 1H), 3.34 (s, 3H), 3.01 – 2.90 (m, 1H), 1.67 – 1.57 (m, 1H), 1.50 – 0.93 (m, 68H), 0.92 – 0.81 (m, 7H), 0.24 (q, *J* = 5.5 Hz, 1H). **$^{13}$C-NMR** (101 MHz, $CDCl_3$) δ 153.84, 133.24, 131.51, 129.75, 125.29, 85.54, 57.82, 57.21, 35.45, 32.49, 32.06, 30.61, 30.11, 30.08, 29.83, 29.80, 29.77, 29.74, 29.57, 29.50, 29.20, 27.70, 26.29, 22.82, 16.00, 15.02, 14.25, 11.48, 8.12. **HRMS (ESI)** Calcd. for $C_{49}H_{92}N_5O_3S$ ($[M + NH_4]^+$): 830.6915, found: 830.6901.

**Compound 29b:**

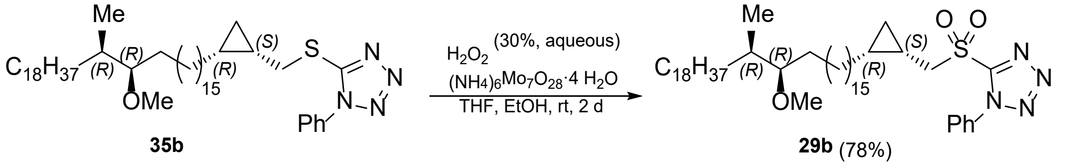

**35b** **29b** (78%)
(21% as sulfoxide)

Compound **29b** (2.17 g, 2.67 mmol) was obtained in a 78% isolated yield by subjecting sulfide **35b** (2.66 g, 3.41 mmol) to the aforementioned procedure. After complete elution of **29b** from the column, eluting with 25% ether in pentane resulted in the recovery of the sulfoxide intermediate (575 mg, 0.72 mmol, 21%). The sulfoxide was used for conditions screening, but by resubmission according to the aforementioned procedure extra sulfone could have been acquired.

**$^{1}$H-NMR** (400 MHz, $CDCl_3$) δ 7.73 – 7.66 (m, 2H), 7.67 – 7.56 (m, 3H), 3.96 (dd, *J* = 14.7, 5.6 Hz, 1H), 3.56 (dd, *J* = 14.7, 9.3 Hz, 1H), 3.34 (s, 3H), 3.00 – 2.91 (m, 1H), 1.68 – 1.59 (m, 1H), 1.50 – 0.93 (m, 68H), 0.93 – 0.79 (m, 7H), 0.24 (q, *J* = 5.6 Hz, 1H). **$^{13}$C-NMR** (101 MHz, $CDCl_3$) δ 153.69, 133.17, 131.21, 129.51, 125.16, 85.31, 57.59, 56.97, 35.33, 32.39, 31.95, 30.48, 30.01, 29.97, 29.75, 29.71, 29.68, 29.66, 29.64, 29.46, 29.42, 29.40, 29.03, 27.59, 26.15, 22.70, 15.85, 14.88, 14.12, 11.28, 7.99. **HRMS (ESI)** Calcd. for $C_{49}H_{92}N_5O_3S$ ($[M + NH_4]^+$): 830.6915, found: 830.6899.

**Compound 29c:**

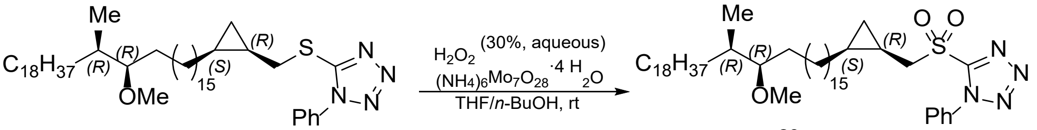

**35c** **29c** (98%)

To a solution of **35c** (2.56 g, 3.28 mmol, 1.0 equiv.) in *n*-BuOH (35 ml) and THF (30 ml) was added ammonium molybdate (1.24 g, 1.00 mmol, 31 mol%) followed by $H_2O_2$ (30% aqueous solution, 5.0 ml, 52.8 mmol, 16 equiv.). The resulting mixture was stirred at rt overnight. $^1$H-NMR showed complete consumption of the sulfide, resulting in a 5:1 sulfone/sulfoxide mixture. The volatiles were evaporated and the residue was diluted with water (100 ml) and extracted with ether (3× 75 ml). The combined organic layer was washed with a saturated aqueous solution of $Na_2SO_3$ (1× 100 ml), water (1× 100 ml), brine (1× 100 ml), dried over $MgSO_4$ and evaporated *in vacuo*. The crude residue was purified by flash column chromatograph using 10% ether in pentane to elute the sulfone (2.16 g) and 30% ether in pentane to elute the sulfoxide (0.47 g). The sulfoxide was resubmitted under the same conditions, but the reaction was stirred for 3 d. The obtained sulfone was combined with the previously obtained batch, resulting in the product (2.60 g, 3.20 mmol, 98% yield) as a colorless oil that solidified upon standing).

*Note: It is not essential for the reaction to be homogeneous, but no visible micelles should be present in order for the reaction to proceed to completion. Unlike EtOH, n-BuOH does not result in micelle formation giving a more reproducible reaction. This method should be used for all diastereomers in future synthesis. A small amount of the formed precipitate was isolated, but this material did not dissolve in ether. Therefore, it was concluded that the formed precipitate is not organic material and must therefore originate from the molybdate catalyst.*

**$^1$H-NMR** (400 MHz, $CDCl_3$) δ 7.72 – 7.66 (m, 2H), 7.65 – 7.56 (m, 3H), 3.96 (dd, *J* = 14.7, 5.6 Hz, 1H), 3.56 (dd, *J* = 14.7, 9.3 Hz, 1H), 3.34 (s, 3H), 3.00 – 2.90 (m, 1H), 1.67 – 1.57 (m, 1H), 1.51 – 0.94 (m, 68H), 0.93 – 0.80 (m, 7H), 0.24 (q, *J* = 5.5 Hz, 1H). **$^{13}$C-NMR** (101 MHz, $CDCl_3$) δ 153.85, 133.24, 131.51, 129.75, 125.29, 85.54, 57.82, 57.21, 35.45, 32.49, 32.06, 30.61, 30.11, 30.08, 29.84, 29.80, 29.77, 29.74, 29.57, 29.50, 29.21, 27.71, 26.29, 22.82, 16.01, 15.02, 14.25, 11.48, 8.12. **HRMS (ESI)** Calcd. for $C_{49}H_{92}N_5O_3S$ ($[M + NH_4]^+$): 830.6915, found: 830.6898.

**Compound 29d:**

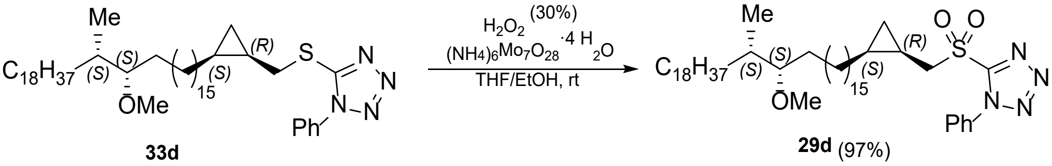

**33d** **29d** (97%)

Compound **29d** (2.65 g, 3.25 mmol) was obtained in 97% yield, by using the same procedure as for **29a**, but with two workup/resubmission sequences.

**$^1$H-NMR** (400 MHz, $CDCl_3$) δ 7.73 – 7.65 (m, 2H), 7.65 – 7.55 (m, 3H), 3.96 (dd, *J* = 14.7, 5.6 Hz, 1H), 3.56 (dd, *J* = 14.6, 9.3 Hz, 1H), 3.34 (s, 3H), 3.00 – 2.90 (m, 1H), 1.68 – 1.57 (m, 1H), 1.51 – 0.93 (m, 68H), 0.96 – 0.79 (m, 7H), 0.24 (q, *J* = 5.6 Hz, 1H). **$^{13}$C-NMR** (101 MHz, $CDCl_3$) δ 153.84, 133.23, 131.50, 129.75, 125.29, 85.54, 77.48, 77.16, 76.84, 57.81, 57.20, 35.45, 32.49, 32.05, 30.61, 30.11, 30.07, 29.83, 29.79, 29.76, 29.74, 29.57, 29.49, 29.20, 27.70, 26.29, 22.82, 16.00, 15.02, 14.24, 11.47, 8.12. **HRMS (ESI)** Calcd. for $C_{49}H_{92}N_5O_3S$ ($[M + NH_4]^+$): 830.6915, found: 830.6899.

**Compound 30a:**

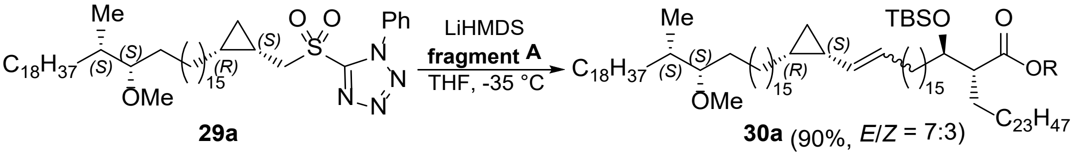

**29a** **30a** (90%, *E*/*Z* = 7:3)

A solution of sulfone **29a** (1.57 g, 1.93 mmol, 1.20 equiv.) in anhydrous THF (40 ml) was cooled to -40 °C. LiHMDS (solid, 0.31 g, 1.85 mmol, 1.15 equiv.) in THF (7 ml) was added while keeping the temperature between -40 °C and -30 °C. The reaction mixture turned bright yellow and after stirring for 15 min, **fragment A** (1.91 g, 1.61 mmol, 1.0 equiv.) in THF (8 ml) was added over 5 min and the reaction mixture was stirred for an additional 45 min. Then, the reaction was quenched by the addition of $HCl_{aq}$ (1 M, 50 ml) and water (100 ml) and the mixture was allowed to reach rt. The mixture was extracted with ether (3× 50 ml), and the combined organic layer was washed with brine (1× 50 ml), dried over $MgSO_4$ and evaporated *in vacuo*. The crude residue was purified by flash column chromatograph using 5% ether in pentane, yielding the product (2.57 g, 1.45 mmol, 90%, *E*/*Z* = 7:3) as a colorless oil.

**$^{1}$H-NMR** (400 MHz, $CDCl_3$) δ 7.33 (d, *J* = 6.6 Hz, 2H), 7.28 – 7.20 (m, 3H), 7.17 (t, *J* = 7.4 Hz, 1H), 7.08 (t, *J* = 7.5 Hz, 2H), 6.87 – 6.76 (m, 4H), 5.70 (d, *J* = 6.1 Hz, 1H), 5.51 (dt, *J* = 14.3, 6.8 Hz, 0.7H), 5.40 (dd, *J* = 11.0, 7.6 Hz, 0.3H), 5.17 (dd, *J* = 15.2, 8.5 Hz, 0.7H), 5.03 (t, *J* = 10.1 Hz, 0.3H), 4.81 (d, *J* = 16.2 Hz, 1H), 4.42 (d, *J* = 16.2 Hz, 1H), 4.14 (p, *J* = 6.6 Hz, 1H), 3.86 (br s, 1H), 3.34 (s, 3H), 2.99 – 2.92 (m, 1H), 2.52 – 2.44 (m, 1H), 2.39 (s, 6H), 2.28 (s, 3H), 2.14 (q, 1H), 1.99 (q, *J* = 6.9 Hz, 1H), 1.68 – 0.73 (m, 165H), 0.11 (q, *J* = 5.3 Hz, 1H), 0.04 (s, 3H), 0.03 (s, 3H).**$^{13}$C-NMR** (101 MHz, $CDCl_3$) δ 172.76, 142.40, 140.50, 138.39, 138.34, 133.26, 132.17, 130.53, 130.29, 129.59, 129.54, 128.43, 128.34, 128.19, 127.92, 127.40, 126.93, 85.50, 77.87, 77.36, 72.83, 57.77, 56.53, 51.68, 48.28, 35.46, 33.64, 32.89, 32.51, 32.08, 30.61, 30.13, 30.09, 29.94, 29.87, 29.82, 29.77, 29.75, 29.73, 29.71, 29.66, 29.59, 29.53, 29.48, 29.33, 29.29, 27.75, 27.72, 27.69, 27.10, 26.29, 26.00, 25.02, 22.97, 22.84, 20.98, 18.57, 18.38, 18.18, 15.07, 15.02, 14.31, 14.26, 14.03, 12.44, -4.27, -4.51. **HRMS (ESI)** Calcd. for $C_{116}H_{211}N_2O_6SSi$ ($[M + NH_4]^+$): 1788.5752, found: 1788.5762.

**Compound 30b:**

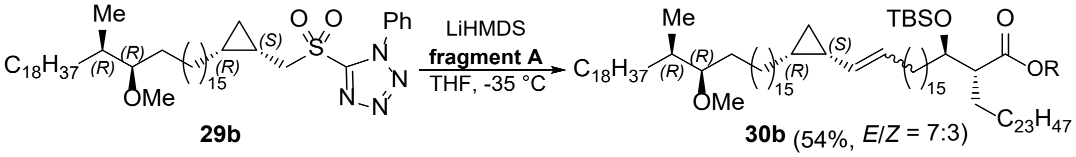


**29b** **30b** (54%, *E*/*Z* = 7:3)

Compound **30b** (2.02 g, 1.14 mmol) was obtained in a 54% yield by submitting sulfone **29b** (2.16, 2.66 mmol, 1.26 equiv.), **fragment A** (2.50 g, 2.11 mmol, 1.0 equiv.) and LiHMDS (crystallized, 0.42g, 2.49 mmol, 1.2 equiv.) to the aforementioned procedure.
A sulfone and aldehyde mixture could be recovered after the product eluted from the column by eluting with 30% ether in pentane. The crude mixture was dissolved in EtOH/THF (8:1, 9 ml) and treated with $NaBH_4$ (100 mg) at 0 °C. After 30 min the reaction mixture was quenched by the addition of $HCl_{aq}$ (1 M, 50 ml) and the mixture was extracted with ether (3× 25 ml). The combined organic layer was concentrated and the sulfone (450 mg, 0.55 mmol) could be recovered after flash column chromatography using 20% ether in pentane.

**$^{1}$H-NMR** (400 MHz, $CDCl_3$) δ 7.33 (d, *J* = 6.5 Hz, 2H), 7.28 – 7.20 (m, 3H), 7.17 (t, *J* = 7.4 Hz, 1H), 7.08 (t, *J* = 7.5 Hz, 2H), 6.87 – 6.76 (m, 4H), 5.70 (d, *J* = 6.1 Hz, 1H), 5.51 (dt, *J* = 14.3, 6.8 Hz, 0.7H), 5.40 (dt, *J* = 10.7, 7.3 Hz, 0.3H), 5.17 (dd, *J* = 15.2, 8.5 Hz, 0.7H), 5.03 (t, *J* = 10.1 Hz, 0.3H), 4.81 (d, *J* = 16.2 Hz, 1H), 4.42 (d, *J* = 16.2 Hz, 1H), 4.14 (p, *J* = 6.7 Hz, 1H), 3.86 (br s, *J* = 6.1, 4.9 Hz, 1H), 3.34 (s, 3H), 3.02 – 2.90 (m, 1H), 2.48 (dt, *J* = 10.6, 5.6 Hz, 1H), 2.39 (s, 6H), 2.28 (s, 3H), 2.14 (q, *J* = 7.6, 7.1 Hz, 0.6H), 1.99 (q, *J* = 6.9 Hz, 1.4H), 1.70 – 0.73 (m, 165H), 0.11 (q, *J* = 5.3 Hz, 1H), 0.04 (s, 3H), 0.04 (s, 3H). **$^{13}$C-NMR** (101 MHz, $CDCl_3$) δ 172.79, 142.44, 140.51, 138.39, 138.34, 133.26, 132.19, 130.55, 130.31, 129.60, 129.56, 128.44, 128.34, 128.21, 127.94, 127.41, 126.94, 85.53, 77.90, 77.36, 72.85, 57.80, 56.54, 51.71, 48.29, 35.47, 33.65, 32.90, 32.51, 32.08, 30.62, 30.45, 30.13, 30.10, 29.94, 29.87, 29.82, 29.78, 29.76, 29.73, 29.72, 29.67, 29.59, 29.53, 29.50, 29.49, 29.33, 29.30, 27.77, 27.73, 27.70, 27.09, 26.30, 26.01, 25.05, 22.99, 22.84, 21.00, 18.59, 18.40, 18.19, 15.07, 15.03, 14.32, 14.26, 14.04, 12.45, -4.26, -4.49. **HRMS (ESI)** Calcd. for $C_{116}H_{211}N_2O_6SSi$ ($[M + NH_4]^+$): 1788.5752, found: 1788.5740.

**Compound 30c:**

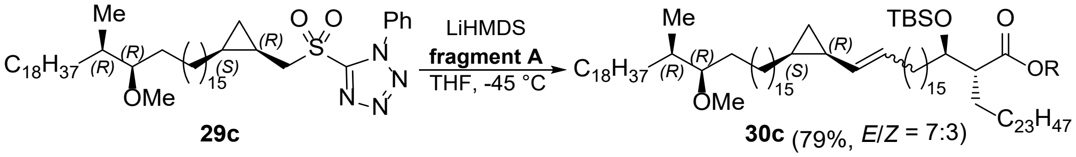


**29c** **30c** (79%, *E*/*Z* = 7:3)

Compound **29c** (1.12 g, 0.63 mmol) was obtained in a 79% yield by the reaction of sulfone **5c** (1.90 g, 2.33 mmol, 2.9 equiv.), **fragment A** (0.95 g, 0.80 mmol, 1.0 equiv.) and LiHMDS (crystallized, 0.38 g, 0.63 mmol, 2.8 equiv.) at -45 °C in an otherwise similar way to **7a**. Eluting with 20% ether in pentane after the product completely eluted from the column resulted in the recovery of sulfone (1.10 g, 1.68 mmol).

**$^{1}$H-NMR** (400 MHz, $CDCl_3$) δ 7.35 – 7.31 (m, 2H), 7.26 – 7.14 (m, 4H), 7.08 (t, 2H), 6.86 – 6.77 (m, 4H), 5.70 (d, *J* = 6.1 Hz, 1H), 5.51 (dt, *J* = 15.3, 6.8 Hz, 0.7H), 5.40 (dd, *J* = 15.2, 8.5 Hz, 0.3H), 5.17 (dd, *J* = 15.2, 8.5 Hz, 0.7H), 5.03 (t, *J* = 9.7 Hz, 0.3H), 4.80 (d, *J* = 16.2 Hz, 1H), 4.42 (d, *J* = 16.2 Hz, 1H), 4.15 (p, *J* = 6.8 Hz, 1H), 3.86 (br s, 1H), 3.34 (s, 3H), 2.99 – 2.92 (m, 1H), 2.51 – 2.44 (m, 1H), 2.39 (s, 6H), 2.28 (s, 3H), 2.14 (q, *J* = 6.8 Hz, 0.6H), 1.99 (q, *J* = 6.8 Hz, 1.4H), 1.66 – 0.75 (m, 165H), 0.10 (q, *J* = 5.4 Hz, 1H), 0.04 (s, 3H), 0.03 (s, 3H). **$^{13}$C-NMR** (101 MHz, $CDCl_3$) δ 172.79, 142.45, 140.52, 138.40, 138.36, 133.29, 132.20, 130.56, 130.32, 129.61, 129.56, 128.45, 128.35, 128.22, 127.95, 127.42, 126.95, 85.55, 77.91, 72.86, 57.80, 56.56, 51.73, 48.30, 35.49, 33.66, 32.90, 32.53, 32.11, 32.09, 30.64, 30.14, 30.10, 29.95, 29.91, 29.87, 29.82, 29.78, 29.76, 29.74, 29.72, 29.67, 29.59, 29.53, 29.50, 29.34, 29.30, 27.79, 27.73, 27.71, 27.08, 26.31, 26.02, 25.08, 22.99, 22.84, 21.00, 18.59, 18.41, 18.20, 15.07, 15.03, 14.33, 14.26, 14.05, 12.46, -4.26, -4.48. **HRMS (ESI)** Calcd. for $C_{116}H_{211}N_2O_6SSi$ ($[M + NH_4]^+$): 1788.5752, found: 1788.5737.

**Compound 30d:**

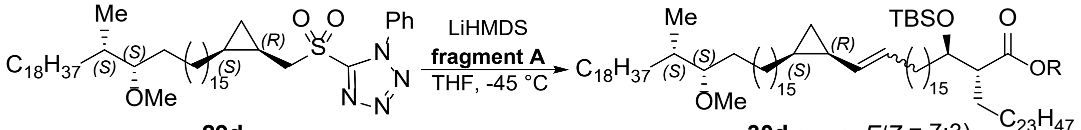

**29d** **30d** (81%, *E*/*Z* = 7:3)

Compound **29d** (3.02 g, 1.70 mmol, 81% yield) was obtained in a similar way to **29a** by reacting sulfone **5d** (2.09, 2.57 mmol, 1.25 equiv.), **fragment A** (2.50 g, 2.11 mmol, 1.00 equiv.) and LiHMDS (solid, 0.41 g, 2.43 mmol, 1.15 equiv.).

**$^1$H-NMR** (400 MHz, $CDCl_3$) δ 7.33 (d, *J* = 7.3 Hz, 2H), 7.24 (d, *J* = 13.5 Hz, 3H), 7.17 (t, *J* = 7.5 Hz, 1H), 7.08 (t, *J* = 7.5 Hz, 2H), 6.87 – 6.76 (m, 4H), 5.70 (d, *J* = 6.1 Hz, 1H), 5.51 (dt, *J* = 14.2, 6.8 Hz, 0.7H), 5.39 (q, *J* = 8.8 Hz, 0.3H), 5.17 (dd, *J* = 15.3, 8.5 Hz, 0.7H), 5.03 (t, *J* = 10.1 Hz, 0.3H), 4.81 (d, *J* = 16.2 Hz, 1H), 4.42 (d, *J* = 16.2 Hz, 1H), 4.14 (p, *J* = 6.7 Hz, 1H), 3.86 (br s, 1H), 3.34 (s, 3H), 2.96 (br s, 1H), 2.47 (dd, *J* = 9.9, 5.3 Hz, 1H), 2.39 (s, 6H), 2.28 (s, 3H), 2.14 (q, *J* = 7.8, 7.3 Hz, 0.6H), 1.99 (q, *J* = 7.0 Hz, 1.4H), 1.81 – 0.74 (m, 165H), 0.14 – 0.06 (m, 1H), 0.04 (s, 6H). **$^{13}$C-NMR** (101 MHz, $CDCl_3$) δ 172.67, 142.28, 140.46, 138.37, 138.32, 133.28, 132.12, 130.47, 130.23, 129.53, 129.48, 128.37, 128.34, 128.14, 127.86, 127.35, 126.92, 85.42, 77.80, 77.36, 72.80, 57.69, 56.47, 51.61, 48.23, 35.44, 33.61, 32.87, 32.50, 32.07, 30.58, 30.12, 30.08, 29.86, 29.76, 29.72, 29.63, 29.57, 29.52, 29.48, 29.45, 29.31, 29.28, 27.71, 27.67, 27.13, 26.26, 25.98, 24.93, 22.93, 22.82, 20.93, 18.54, 18.34, 18.13, 15.07, 14.98, 14.28, 14.23, 12.38, -4.31, -4.55. **HRMS (ESI)** Calcd. for $C_{116}H_{211}N_2O_6SSi$ ($[M + NH_4]^+$): 1788.5752, found: 1788.5740.

**Compound 36a:**

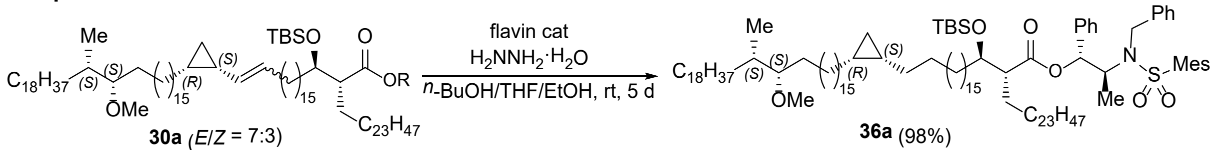

**30a** (*E*/*Z* = 7:3) **36a** (98%)

Compound **30a** (3.20 g, 1.81 mmol, 1.0 equiv.) was dissolved in *n*-BuOH/THF (5:1 v/v, 24 ml) and hydrazine monohydrate (2.2 ml, 25 equiv.) followed by the flavin catalyst[12] (0.34 g, 0.84 mmol, 0.46 equiv.) were added. EtOH (5 ml) was added followed by enough THF so that the liquids formed a single phase (parts of the catalyst did not dissolve). The reaction mixture was stirred at rt, and the volatiles were removed *in vacuo* after full conversion according to $^1$H-NMR (around 5 d). To the crude residue was added water (75 ml), and the aqueous phase was extracted with pentane (3× 75 ml). The combined organic layer was washed with brine (1× 50 ml), dried over $MgSO_4$ and concentrated *in vacuo*. The crude product was filtered over a plug of silica using 10% ether in pentane, yielding the product (3.14 g, 1.77 mmol, 98%) as a colorless oil.

**$^1$H-NMR** (400 MHz, $CDCl_3$) δ 7.33 (d, *J* = 7.2 Hz, 2H), 7.28 – 7.20 (m, 3H), 7.17 (t, *J* = 7.4 Hz, 1H), 7.09 (t, *J* = 7.5 Hz, 2H), 6.87 – 6.76 (m, 4H), 5.71 (d, *J* = 6.1 Hz, 1H), 4.81 (d, *J* = 16.2 Hz, 1H), 4.43 (d, *J* = 16.2 Hz, 1H), 4.15 (p, *J* = 6.7 Hz, 1H), 3.91 – 3.83 (m, 1H), 3.34 (s, 3H), 3.00 – 2.92 (m, 1H), 2.52 – 2.44 (m, 1H), 2.40 (s, 6H), 2.29 (s, 3H), 1.69 – 1.57 (m, 1H), 1.57 – 1.02 (m, 147H), 1.02 – 0.92 (m, 2H), 0.92 – 0.80 (m, 18H), 0.70 – 0.61 (m, 2H), 0.60 – 0.52 (m, 1H), 0.04 (s, 6H), -0.33 (q, *J* = 4.9 Hz, 1H). **$^{13}$C-NMR** (101 MHz, $CDCl_3$) δ 172.66, 142.27, 140.46, 138.38, 138.32, 133.28, 132.12, 128.37, 128.35, 128.13, 127.86, 127.35, 126.93, 85.42, 77.79, 77.36, 72.80, 57.69, 56.47, 51.60, 48.23, 35.45, 33.62, 32.51, 32.08, 30.59, 30.40, 30.37, 30.13, 30.09, 29.88, 29.84, 29.81, 29.78, 29.76, 29.72, 29.68, 29.64, 29.53, 29.46, 28.86, 27.71, 27.67, 27.15, 26.26, 25.98, 24.91, 22.93, 22.83, 20.92, 18.13, 15.89, 15.08, 14.98, 14.23, 11.07, -4.31, -4.55. **HRMS (ESI)** Calcd. for $C_{116}H_{213}N_2O_6SSi$ ($[M + NH_4]^+$): 1790.5908, found: 1790.5884.

**Compound 36b:**

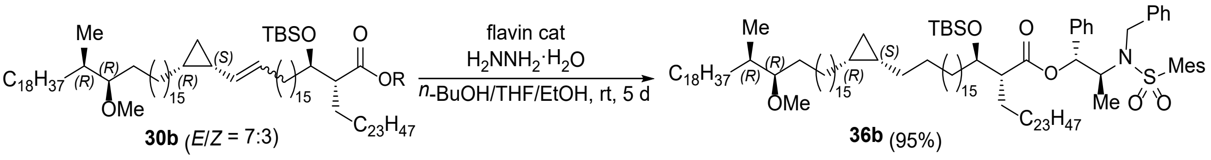

**30b** (*E*/*Z* = 7:3) **36b** (95%)

Compound **36b** (2.63 g, 1.48 mmol) was obtained in 95% isolated yield by subjecting **30b** (2.77 g, 1.56 mmol) to the aforementioned procedure for 5 days.

**$^1$H-NMR** (400 MHz, $CDCl_3$) δ 7.33 (d, *J* = 7.2 Hz, 2H), 7.29 – 7.13 (m, 4H), 7.08 (t, *J* = 7.5 Hz, 2H), 6.88 – 6.76 (m, 4H), 5.70 (d, *J* = 6.1 Hz, 1H), 4.81 (d, *J* = 16.2 Hz, 1H), 4.42 (d, *J* = 16.2 Hz, 1H), 4.14 (p, *J* = 6.7 Hz, 1H), 3.86 (br s, 1H), 3.34 (s, 3H), 3.01 – 2.88 (m, 1H), 2.52 – 2.44 (m, 1H), 2.39 (s, 6H), 2.28 (s, 3H), 1.69 – 1.57 (m, 1H), 1.57 – 1.03 (m, 147H), 1.03 – 0.91 (m, 2H), 0.91 – 0.81 (m, 18H), 0.69 – 0.60 (m, 2H), 0.60 – 0.52 (m, 1H), 0.04 (s, 3H), 0.03 (s, 3H), -0.33 (q, *J* = 5.0 Hz, 1H). **$^{13}$C-NMR** (101 MHz, $CDCl_3$) δ 172.69, 142.30, 140.47, 138.38, 138.33, 133.28, 132.14, 128.39, 128.35, 128.15, 127.88, 127.36, 126.93, 85.44, 77.81, 77.36, 72.81, 57.71, 56.49, 51.63, 48.24, 35.46, 33.62, 32.51, 32.08, 30.59,

30.37, 30.13, 30.09, 29.88, 29.83, 29.79, 29.77, 29.72, 29.69, 29.64, 29.53, 29.46, 28.87, 27.72, 27.69, 27.14, 26.27, 25.98, 24.94, 22.94, 22.83, 20.94, 18.14, 15.89, 15.08, 14.99, 14.24, 11.07, -4.30, -4.54. **HRMS (ESI)** Calcd. for $C_{116}H_{213}N_2O_6SSi$ ([M + $NH_4$]$^+$): 1790.5908, found: 1790.5887.

**Compound 36c:**

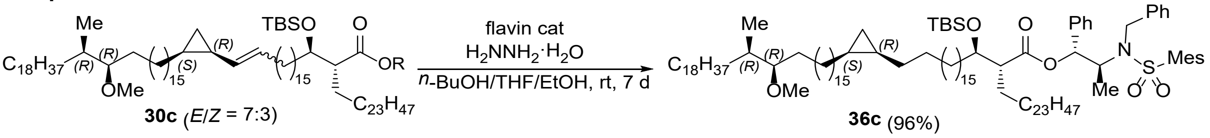


Compound **36c** (1.40 g, 0.79 mmol) was obtained in 96% isolated yield by subjecting **30c** (1.45 g, 0.82 mmol) to the aforementioned procedure for 7 days.

**$^1$H-NMR** (400 MHz, $CDCl_3$) δ 7.35 – 7.30 (m, 2H), 7.25 – 7.14 (m, 4H), 7.08 (t, *J* = 7.8 Hz, 2H), 6.85 – 6.77 (m, 4H), 5.70 (d, *J* = 6.2 Hz, 1H), 4.81 (d, *J* = 16.2 Hz, 1H), 4.42 (d, *J* = 16.2 Hz, 1H), 4.14 (p, *J* = 6.7 Hz, 1H), 3.90 – 3.80 (m, 1H), 3.34 (s, 3H), 3.00 – 2.90 (m, 1H), 2.52 – 2.43 (m, 1H), 2.39 (s, 6H), 2.28 (s, 3H), 1.68 – 1.57 (m, 1H), 1.53 – 1.03 (m, 147H), 1.00 – 0.92 (m, 2H), 0.92 – 0.81 (m, 18H), 0.69 – 0.61 (m, 2H), 0.59 – 0.52 (m, 1H), 0.04 (s, 3H), 0.03 (s, 3H), -0.33 (q, *J* = 5.1 Hz, 1H). **$^{13}$C-NMR** (101 MHz, $CDCl_3$) δ 172.75, 142.37, 140.50, 138.39, 138.34, 133.28, 132.17, 128.42, 128.35, 128.18, 127.91, 127.39, 126.94, 125.58, 85.49, 77.86, 77.36, 72.84, 57.75, 56.52, 51.67, 48.27, 35.47, 33.64, 32.52, 32.09, 30.61, 30.43, 30.39, 30.14, 30.10, 29.88, 29.84, 29.81, 29.78, 29.66, 29.54, 29.48, 28.88, 27.73, 27.12, 26.29, 26.00, 24.99, 22.97, 22.84, 20.97, 18.17, 15.91, 15.08, 15.01, 14.25, 11.08, -4.28, -4.51. **HRMS (ESI)** Calcd. for $C_{116}H_{213}N_2O_6SSi$ ([M + $NH_4$]$^+$): 1790.5908, found: 1790.5887.

**Compound 36d:**

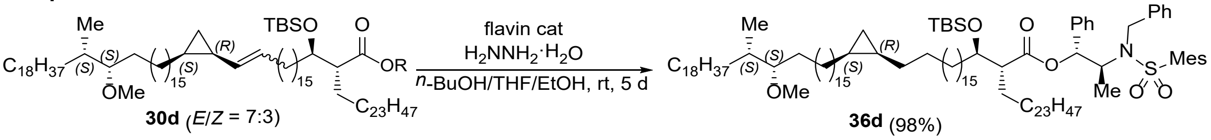


Compound **36d** (2.96 g, 1.67 mmol) was obtained in 98% isolated yield by subjecting **30d** (3.00 g, 1.69 mmol) to the aforementioned procedure for 5 days.

**$^1$H-NMR** (400 MHz, $CDCl_3$) δ 7.33 (d, *J* = 7.1 Hz, 2H), 7.28 – 7.20 (m, 3H), 7.17 (t, *J* = 7.4 Hz, 1H), 7.08 (t, *J* = 7.5 Hz, 2H), 6.85 – 6.77 (m, 4H), 5.70 (d, *J* = 6.1 Hz, 1H), 4.81 (d, *J* = 16.1 Hz, 1H), 4.42 (d, *J* = 16.2 Hz, 1H), 4.14 (p, *J* = 6.8 Hz, 1H), 3.86 (br s, 1H), 3.34 (s, 3H), 3.00 – 2.91 (m, 1H), 2.53 – 2.43 (m, 1H), 2.39 (s, 6H), 2.28 (s, 3H), 1.69 – 1.56 (m, 1H), 1.56 – 1.01 (m, 147H), 1.01 – 0.92 (m, 2H), 0.92 – 0.81 (m, 18H), 0.69 – 0.60 (m, 2H), 0.60 – 0.52 (m, 1H), 0.04 (s, 6H), -0.33 (q, *J* = 4.9 Hz, 1H). **$^{13}$C-NMR** (101 MHz, $CDCl_3$) δ 172.76, 142.39, 140.50, 138.39, 138.34, 133.28, 132.18, 128.43, 128.35, 128.19, 127.92, 127.40, 126.94, 85.50, 77.87, 77.36, 72.84, 57.77, 56.53, 51.68, 48.28, 35.47, 33.65, 32.52, 32.09, 30.61, 30.39, 30.14, 30.10, 29.88, 29.83, 29.80, 29.78, 29.75, 29.71, 29.66, 29.54, 29.49, 28.88, 27.75, 27.73, 27.11, 26.29, 26.01, 25.01, 22.98, 22.84, 20.98, 18.18, 15.92, 15.08, 15.02, 14.26, 11.08, -4.27, -4.51. **HRMS (ESI)** Calcd. for $C_{116}H_{213}N_2O_6SSi$ ([M + $NH_4$]$^+$): 1790.5908, found: 1790.5881.

**Compound 31a:**

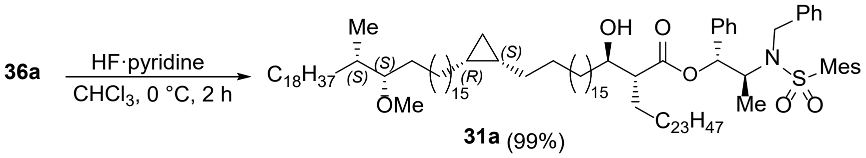


To a 0 °C solution of TBS ether **36a** (3.08 g, 1.74 mmol, 1.0 equiv.) in chloroform (10 ml), hydrogen fluoride pyridine (70% HF, 2.5 ml, 19.4 mmol, 11 equiv.) was slowly added. The reaction mixture was stirred at 0 °C for 2 h, and carefully poured in aqueous saturated $NaHCO_3$ (200 ml). The mixture was extracted with 1:1 ether/pentane (3× 75 ml), and the combined organic layer was washed with brine (1× 100 ml), dried over $MgSO_4$ and concentrated *in vacuo*. The crude residue was purified by flash column chromatography using 10% ether in pentane, yielding the product (2.87 g, 1.73 mmol, 99%).

**$^1$H-NMR** (400 MHz, $CDCl_3$) δ 7.32 – 7.17 (m, 6H), 7.13 (app. t, *J* = 7.5 Hz, 2H), 6.88 – 6.79 (m, 4H), 5.81 (d, *J* = 5.6 Hz, 1H), 4.77 (d, *J* = 16.3 Hz, 1H), 4.51 (d, *J* = 16.4 Hz, 1H), 4.15 (p, *J* = 6.8 Hz, 1H), 3.64 (br s, 1H), 3.34 (s, 3H), 3.00 – 2.90 (m, 1H), 2.47 – 2.36 (m, 7H), 2.28 (s, 3H), 1.69 – 0.94 (m, 150H), 0.94 – 0.79 (m, 9H), 0.70 – 0.60 (m, 2H), 0.59 – 0.52 (m, 1H), -0.33 (q, *J* = 4.9 Hz, 1H). **$^{13}$C-NMR** (101 MHz, $CDCl_3$) δ 174.68, 142.47, 140.45, 138.54, 138.08, 133.37, 132.16, 128.38, 128.29, 128.14, 128.08, 127.30, 126.76, 85.49, 78.08, 77.36, 72.36, 57.75, 56.49, 51.36, 48.21, 35.45, 32.50, 32.07, 30.60, 30.36, 30.11, 30.07, 29.85, 29.81, 29.76, 29.71, 29.61, 29.51, 29.47, 28.86, 27.70, 27.25, 26.27, 25.70,

22.98, 22.82, 20.96, 15.89, 14.99, 14.48, 14.24, 11.05. **HRMS (ESI)** Calcd. for $C_{110}H_{199}N_2O_6S$ ($[M + NH_4]^+$): 1676.5043, found: 1676.5026.

*Note: the reaction mixture should be poured in a large beaker (1L) in order to prevent spilling during the $NaHCO_3$ quench, which results in violent gas formation. Moreover, it was noticed that washing the white slimy residue in the flask with the pentane/ether used for extractions (without transferring the white residue) was sufficient for complete recovery of the product. Dissolving this white substance in water and transferring it to the separatory funnel led to a severe emulsion, which only could be removed by filtration over Celite.*

*Note: if necessary, partial separation of diastereomers in the anti-aldol segment (2R,3R) is possible at this stage by careful flash column chromatography using 6% ether in pentane. Fractions should be checked by $^1$H-NMR. Once the product starts eluting in an unacceptable diastereomeric ratio, increasing the mobile phase to 10-15% ether resulted in the complete elution of the diastereomeric mixture, which can be resubmitted to the same column purification if required. Furthermore, this slow elution resulted in the separation of very minor traces of material that was not hydrogenated (<5% on $^1$H-NMR) during the diimide reduction. Although some separation is possible it is best to push the hydrogenation as much as possible in order to prevent loss of material in this hard separation. A single column of **31a** (2.87 g, 1.73 mmol, dr 94:6) using a 6% ether in pentane mobile phase resulted in pure product (2.13 g, 74%, dr > 97:3) and slightly impure product (0.74g, 25%) containing traces of olefin.*

**Compound 31b:**

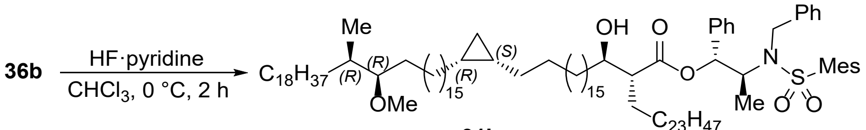


**31b** (98%)

Compound **31b** (2.40 g, 1.44 mmol) was obtained in 98% isolated yield by subjecting **36b** (2.61 g, 1.47 mmol) to the aforementioned procedure, but using only 6.4 equiv. of hydrogen fluoride pyridine solution.

**$^1$H-NMR** (400 MHz, $CDCl_3$) δ 7.32 – 7.17 (m, 6H), 7.16 – 7.10 (m, 2H), 6.87 – 6.79 (m, 4H), 5.81 (d, *J* = 5.6 Hz, 1H), 4.77 (d, *J* = 16.4 Hz, 1H), 4.51 (d, *J* = 16.4 Hz, 1H), 4.20 – 4.10 (m, 1H), 3.64 (br s, 1H), 3.34 (s, 3H), 2.99 – 2.89 (m, 1H), 2.45 – 2.38 (m, 7H), 2.28 (s, 3H), 1.67 – 0.94 (m, 150H), 0.92 – 0.81 (m, 9H), 0.69 – 0.60 (m, 2H), 0.59 – 0.52 (m, 1H), -0.34 (q, *J* = 5.0 Hz, 1H). **$^{13}$C-NMR** (101 MHz, $CDCl_3$) δ 174.62, 142.39, 140.40, 138.53, 138.09, 133.38, 132.12, 128.32, 128.24, 128.12, 128.02, 127.24, 126.73, 85.44, 78.02, 77.36, 72.34, 57.69, 56.47, 51.37, 48.17, 35.43, 35.39, 32.48, 32.05, 30.57, 30.34, 30.10, 30.05, 29.84, 29.80, 29.75, 29.73, 29.71, 29.59, 29.50, 29.45, 28.84, 27.68, 27.24, 26.24, 25.66, 22.94, 22.80, 20.91, 15.87, 14.96, 14.45, 14.21, 11.04. **HRMS (ESI)** Calcd. for $C_{110}H_{199}N_2O_6S$ ($[M + NH_4]^+$): 1676. 5043, found: 1676.5027.

**Compound 31c:**

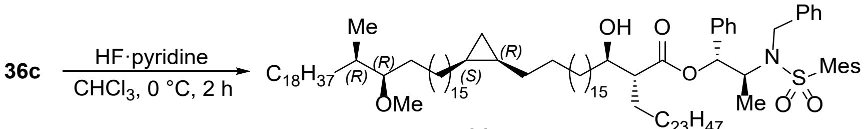


**31c** (97%)

Compound **31c** (1.32 g, 0.74 mmol) was obtained in 97% isolated yield by subjecting **36c** (1.36 g, 1.37 mmol) to the aforementioned procedure, but using only 6.4 equiv. of pyridine hydrogen fluoride solution.

**$^1$H-NMR** (400 MHz, $CDCl_3$) δ 7.31 – 7.17 (m, 6H), 7.16 – 7.10 (m, 2H), 6.87 – 6.80 (m, 4H), 5.81 (d, J = 5.6 Hz, 1H), 4.77 (d, J = 16.3 Hz, 1H), 4.51 (d, J = 16.4 Hz, 1H), 4.20 – 4.11 (m, 1H), 3.69 – 3.60 (m, 1H), 3.34 (s, 3H), 2.99 – 2.92 (m, 1H), 2.46 – 2.37 (m, 7H), 2.32 (d, J = 7.5 Hz, 1H), 2.29 (s, 3H), 1.68 – 1.59 (m, 1H), 1.56 – 0.96 (m, 150H), 0.93 – 0.78 (m, 9H), 0.70 – 0.60 (m, 2H), 0.59 – 0.51 (m, 1H), -0.33 (q, J = 5.0 Hz, 1H). **$^{13}$C-NMR** (101 MHz, $CDCl_3$) δ 174.72, 142.52, 140.46, 138.53, 138.08, 133.36, 132.18, 128.40, 128.32, 128.15, 128.12, 127.32, 126.77, 85.52, 78.12, 72.37, 57.78, 56.50, 51.36, 48.23, 35.46, 32.51, 32.07, 30.61, 30.37, 30.12, 30.08, 29.85, 29.81, 29.77, 29.75, 29.72, 29.62, 29.51, 29.48, 28.86, 27.71, 27.26, 26.28, 25.72, 23.00, 22.83, 20.98, 15.90, 15.01, 14.50, 14.25, 11.06. **HRMS (ESI)** Calcd. for $C_{110}H_{199}N_2O_6S$ ($[M + NH_4]^+$): 1676. 5043, found: 1676.5030.

**Compound 31d:**

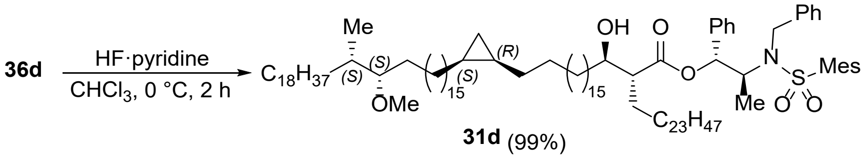

Compound **31d** (2.70 g, 1.63 mmol) was obtained in 99% isolated yield by subjecting **36d** (2.92 g, 1.65 mmol) to the aforementioned procedure, but using only 6.4 equiv. of pyridine hydrogen fluoride solution.

**$^1$H-NMR** (400 MHz, $CDCl_3$) δ 7.32 – 7.17 (m, 6H), 7.16 – 7.10 (m, 2H), 6.87 – 6.79 (m, 4H), 5.81 (d, *J* = 5.6 Hz, 1H), 4.77 (d, *J* = 16.3 Hz, 1H), 4.51 (d, *J* = 16.4 Hz, 1H), 4.21 – 4.10 (m, 1H), 3.63 (br s, 1H), 3.34 (s, 3H), 3.00 – 2.90 (m, 1H), 2.50 – 2.35 (m, 7H), 2.28 (s, 3H), 1.71 – 0.94 (m, 150H), 0.93 – 0.80 (m, 9H), 0.69 – 0.60 (m, 2H), 0.60 – 0.51 (m, 1H), -0.34 (q, *J* = 5.0 Hz, 1H). **$^{13}$C-NMR** (101 MHz, $CDCl_3$) δ 174.62, 142.39, 140.41, 138.53, 138.09, 133.38, 132.12, 128.33, 128.24, 128.12, 128.03, 127.24, 126.73, 85.44, 78.02, 77.36, 72.34, 57.70, 56.47, 51.37, 48.17, 35.43, 35.40, 32.49, 32.05, 30.57, 30.34, 30.10, 30.06, 29.84, 29.80, 29.75, 29.73, 29.71, 29.59, 29.50, 29.45, 28.84, 27.68, 27.23, 26.24, 25.66, 22.95, 22.80, 20.91, 15.87, 14.96, 14.45, 14.21, 11.04. **HRMS (ESI)** Calcd. for $C_{110}H_{199}N_2O_6S$ ($[M + NH_4]^+$): 1676. 5043, found: 1676.5027.

**Compound 1a:**

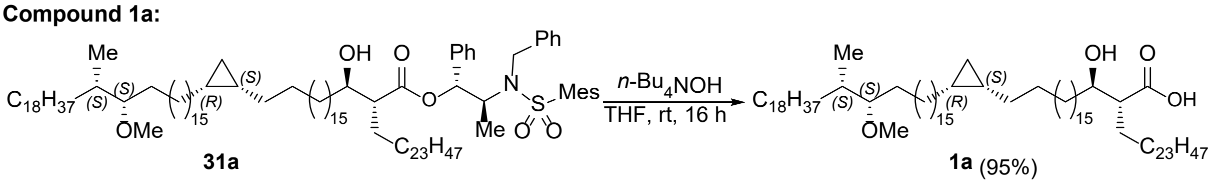

To a stirred solution of ester **31a** (550 mg, 0.33 mmol) in THF (3 ml) a solution of aqueous tetrabutylammonium hydroxide (1.5 M, 0.55 ml, 0.83 mmol, 2.5 equiv.) was added. The mixture was stirred at rt overnight. The reaction mixture was poured in an aqueous $KHSO_4$ solution (5%, 100 ml) and extracted with ether (3× 20 ml). The combined organic layer was washed with brine (1× 20 ml), dried over $MgSO_4$ and concentrated *in vacuo*. The crude residue was redissolved in pentane (200 ml) and washed with acetonitrile (5× 30 ml). The pentane was concentrated *in vacuo*, yielding the product (394 mg, 0.31 mmol, 95%) in sufficient purity as a white solid.

*Note: if necessary, the product can be purified further by flash column chromatography using a MeOH/$CHCl_3$ mobile phase on normal silica gel. A diol silica stationary phase or ether/pentane and ether/ $CH_2Cl_2$ mobile phases resulted in inferior product recovery.*

**$^1$H-NMR** (400 MHz, $CDCl_3$) δ 3.76 – 3.66 (m, 1H), 3.34 (s, 3H), 3.00 – 2.93 (m, 1H), 2.46 (dt, *J* = 9.0, 5.3 Hz, 1H), 1.83 – 0.95 (m, 147H), 0.94 – 0.78 (m, 9H), 0.69 – 0.60 (m, 2H), 0.59 – 0.50 (m, 1H), -0.34 (q, *J* = 5.0 Hz, 1H). **$^{13}$C-NMR** (151 MHz, $CDCl_3$) δ 180.08, 85.76, 72.29, 57.82, 51.05, 35.67, 35.52, 32.54, 32.10, 30.65, 30.39, 30.15, 30.10, 29.88, 29.84, 29.78, 29.70, 29.66, 29.61, 29.54, 28.89, 27.73, 27.51, 26.32, 25.90, 22.86, 15.94, 15.05, 14.27, 11.08. **HRMS (ESI)** Calcd. for $C_{85}H_{169}O_4$ ($[M + H]^+$): 1254.3015, found: 1254.3076.

**Compound 1b:**

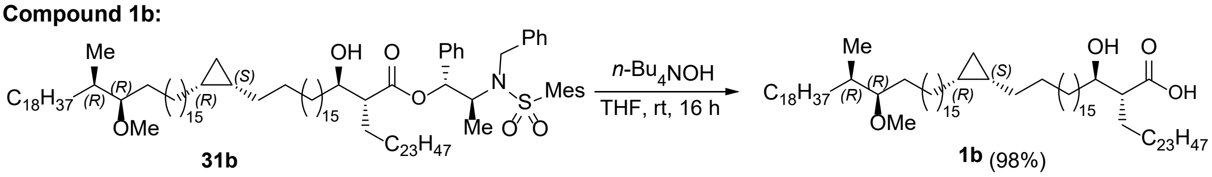

Compound **1b** (420 mg, 0.33 mmol) was obtained in 98% isolated yield by subjecting **31b** (570 mg, 0.34 mmol) to the aforementioned procedure.

**$^1$H-NMR** (400 MHz, $CDCl_3$) δ 3.76 – 3.66 (m, 1H), 3.34 (s, 3H), 3.01 – 2.92 (m, 1H), 2.46 (dt, *J* = 9.0, 5.3 Hz, 1H), 1.81 – 0.98 (m, 147H), 0.95 – 0.79 (m, 9H), 0.69 – 0.59 (m, 2H), 0.59 – 0.51 (m, 1H), -0.34 (q, *J* = 4.9 Hz, 1H). **$^{13}$C-NMR** (151 MHz, $CDCl_3$) δ 180.21, 85.77, 72.29, 57.81, 51.09, 35.66, 35.53, 32.54, 32.10, 30.65, 30.40, 30.15, 30.10, 29.88, 29.84, 29.81, 29.79, 29.71, 29.65, 29.61, 29.54, 28.90, 27.73, 27.52, 26.32, 25.90, 22.86, 15.95, 15.05, 14.27, 11.08. **HRMS (ESI)** Calcd. for $C_{85}H_{169}O_4$ ($[M + H]^+$): 1254.3015, found: 1254.3069.

**Compound 1c:**

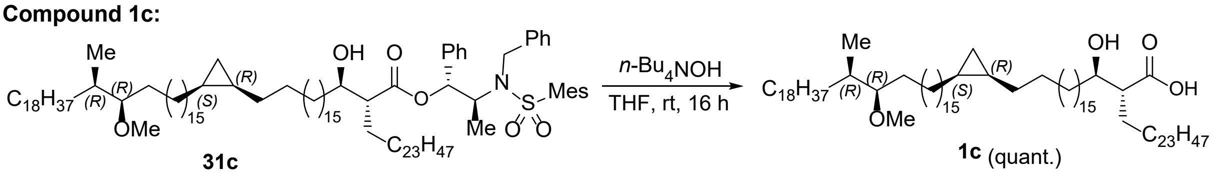

Compound **1c** (649 mg, 0.52 mmol) was obtained in quantitative isolated yield by subjecting **31c** (860 mg, 0.52 mmol) to the aforementioned procedure.

**$^1$H-NMR** (400 MHz, $CDCl_3$) δ 3.77 – 3.65 (m, 1H), 3.34 (s, 3H), 2.99 – 2.93 (m, 1H), 2.45 (dt, *J* = 9.0, 5.4 Hz, 1H), 1.78 – 1.02 (m, 147H), 0.95 – 0.80 (m, 9H), 0.69 – 0.60 (m, 2H), 0.59 – 0.52 (m, 1H), -0.34 (q, *J* = 5.0 Hz, 1H). **$^{13}$C-NMR** (151 MHz, $CDCl_3$) δ 179.99, 85.74, 72.29, 57.83, 51.03, 35.68, 35.49, 32.52, 32.09, 30.63, 30.39, 30.15, 30.10, 29.87, 29.83, 29.80, 29.77, 29.70, 29.67, 29.60, 29.53, 28.89, 27.73, 27.50, 26.31, 25.89, 22.86, 15.94, 15.05, 14.28, 11.07. **HRMS (ESI)** Calcd. for $C_{85}H_{169}O_4$ ([M + H]$^+$): 1254.3015, found: 1254.3034.

**Compound 1d:**

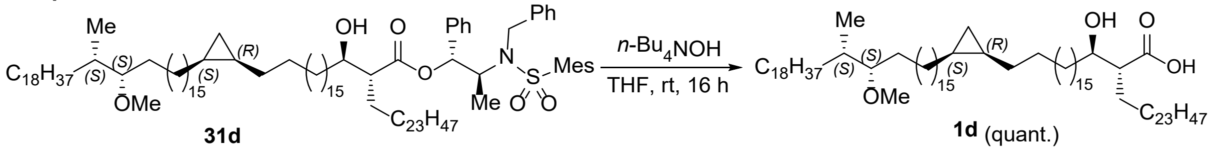

Compound **1d** (234 mg, 0.19 mmol) was obtained in quantitative isolated yield by subjecting **31d** (310 mg, 0.19 mmol) to the aforementioned procedure.

**$^1$H-NMR** (400 MHz, $CDCl_3$) δ 3.77 – 3.67 (m, 1H), 3.34 (s, 3H), 3.01 – 2.92 (m, 1H), 2.46 (dt, *J* = 9.8, 5.4 Hz, 1H), 1.80 – 0.99 (m, 147H), 0.94 – 0.78 (m, 9H), 0.71 – 0.60 (m, 2H), 0.60 – 0.49 (m, 1H), -0.33 (q, *J* = 4.9 Hz, 1H). **$^{13}$C-NMR** (151 MHz, $CDCl_3$) δ 180.22, 85.77, 77.37, 77.16, 76.95, 72.29, 57.81, 51.07, 35.67, 35.52, 32.54, 32.10, 30.65, 30.40, 30.15, 30.10, 29.88, 29.84, 29.81, 29.79, 29.71, 29.66, 29.61, 29.54, 28.90, 27.73, 27.51, 26.32, 25.90, 22.86, 15.95, 15.05, 14.27, 11.08. **HRMS (ESI)** Calcd. for $C_{85}H_{169}O_4$ ([M + H]$^+$): 1254.3015, found: 1254.3071.

All Glucose monomycolates were obtained following a procedure reported by Prandi.[13]

**Compound 32a:**

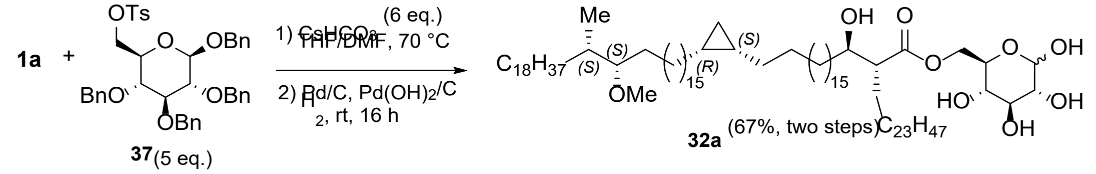

Esterification:
Compound **1a** (75 mg, 60 µmol, 1.0 equiv.), **37** (208 mg, 0.30 mmol, 5 equiv.) and anhydrous $CsHCO_3$ (70 mg, 0.36 mmol, 6.0 equiv.) were charged in a pressure tube, and dissolved in THF/DMF (5:1 v/v, 0.6 ml). The reaction mixture was stirred at 70 °C for 10 h, after which it was cooled to rt, poured in water (20 ml) and extracted with ether (3× 15 ml). The combined organic layer was washed with brine (1× 15 ml), dried over $MgSO_4$ and concentrated *in vacuo*. The crude residue was redissolved in pentane (40 ml) and washed with acetonitrile (3× 15 ml). The pentane layer was concentrated *in vacuo* and the residue was purified by flash column chromatography using 5-10% EtOAc in pentane, yielding the product (74 mg, 42 µmol, 70%) as a colorless oil that solidified upon standing.
**$^1$H-NMR** (400 MHz, $CDCl_3$) δ 7.37 – 7.20 (m, 20H), 4.96 – 4.82 (m, 4H), 4.76 (d, *J* = 10.9 Hz, 1H), 4.68 (d, *J* = 10.9 Hz, 1H), 4.64 – 4.54 (m, 2H), 4.54 – 4.46 (m, 2H), 4.19 (dd, *J* = 11.5, 4.6 Hz, 1H), 3.70 – 3.57 (m, 2H), 3.55 – 3.41 (m, 3H), 3.32 (s, 3H), 2.99 – 2.89 (m, 1H), 2.50 – 2.38 (m, 2H), 1.77 – 0.94 (m, 147H), 0.94 – 0.72 (m, 9H), 0.69 – 0.58 (m, 2H), 0.58 – 0.48 (m, 1H), -0.36 (q, *J* = 5.0 Hz, 1H). **$^{13}$C-NMR** (151 MHz, $CDCl_3$) δ 175.34, 138.54, 138.43, 137.87, 137.32, 128.65, 128.56, 128.54, 128.49, 128.27, 128.18, 128.12, 128.11, 128.02, 128.00, 127.83, 102.48, 85.59, 84.70, 82.46, 77.97, 75.89, 75.28, 75.08, 73.03, 72.47, 71.27, 63.03, 57.86, 51.48, 35.81, 35.50, 32.54, 32.09, 30.65, 30.39, 30.14, 30.10, 29.90, 29.86, 29.82, 29.77, 29.73, 29.63, 29.52, 28.88, 27.73, 27.69, 26.32, 26.04, 22.85, 15.94, 15.05, 14.27, 11.08. **HRMS (ESI)** Calcd. for $C_{119}H_{206}O_9N$ ([M + $NH_4$]$^+$): 1793.5687, found:1793.5707.

Deprotection:
The protected GMM obtained in the previous reaction was dissolved in deoxygenated pentane/EtOAc/MeOH (4:5:1 v/v/v, 4.5 ml), after which $Pd(OH)_2/C$ (20%, 6 mg, 9 µmol, 20 mol%) and Pd/C (9 mg, 8 µmol, 20 mol%) were added. Then, the system was put under an $H_2$ atmosphere by three successive vacuum and hydrogen cycles, and left to stir overnight under a positive pressure of $H_2$. The reaction mixture was filtered over Celite, and the Celite was washed with chloroform/

methanol (4:1 v/v, 200 ml). The filtrate was concentrated *in vacuo*, and purified by flash column chromatography using 5-10% MeOH in $CH_2Cl_2$, yielding the product (57 mg, 40 µmol, 96%, α:β = 3:2) as a colorless oil that solidified upon standing.

**$^{1}$H-NMR** (400 MHz, $CDCl_3$/MeOD 4:1) δ 5.02 (d, *J* = 3.7 Hz, 0.6H), 4.38 (d, *J* = 7.8 Hz, 0.4H), 4.36 – 4.30 (m, 1H), 4.19 – 4.10 (m, 1H), 3.90 – 3.84 (m, 0.6H), 3.60 – 3.50 (m, 1.6H), 3.48 – 3.44 (m, 0.4H), 3.42 – 3.35 (m, 0.4H), 3.32 – 3.17 (m, 4.6H), 3.11 – 3.03 (m, 0.4H), 2.92 – 2.83 (m, 1H), 2.36 – 2.26 (m, 1H), 1.57 – 0.88 (m, 147H), 0.81 – 0.69 (m, 9H), 0.57 – 0.48 (m, 2H), 0.47 – 0.39 (m, 1H), -0.45 (q, *J* = 5.2 Hz, 1H). **$^{13}$C-NMR** (151 MHz, $CDCl_3$/MeOD 4:1) δ 175.24, 175.19, 96.57, 92.33, 85.63, 76.21, 74.50, 73.71, 73.43, 72.42, 72.39, 72.19, 70.49, 70.35, 69.23, 63.54, 57.49, 52.80, 52.68, 35.27, 34.84, 32.30, 31.82, 30.39, 30.11, 30.10, 29.83, 29.76, 29.63, 29.61, 29.59, 29.55, 29.53, 29.43, 29.33, 29.31, 29.25, 29.05, 29.02, 28.62, 27.37, 27.31, 27.28, 25.94, 25.32, 25.29, 22.56, 15.66, 14.63, 13.88, 10.75. **HRMS (ESI)** Calcd. for $C_{91}H_{182}O_9N$ ($[M + NH_4]^+$): 1433.3809, found: 1433.3843.

**Compound 32b:**

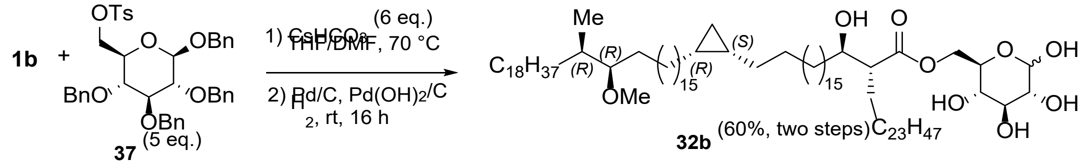


Esterification:
The reaction was performed in 67% isolated yield by subjecting **1b** (75 mg, 60 µmol), **37** (208 mg, 0.30 mmol, 5.0 equiv.) and anhydrous $CsHCO_3$ (70 mg, 0.36 mmol, 6.0 equiv.) to the aforementioned procedure.

**$^{1}$H-NMR** (400 MHz, $CDCl_3$) δ 7.39 – 7.23 (m, 20H), 4.98 – 4.85 (m, 4H), 4.78 (d, *J* = 10.9 Hz, 1H), 4.70 (d, *J* = 10.9 Hz, 1H), 4.65 – 4.56 (m, 2H), 4.57 – 4.49 (m, 2H), 4.22 (dd, *J* = 11.6, 4.7 Hz, 1H), 3.70 – 3.60 (m, 2H), 3.57 – 3.45 (m, 3H), 3.34 (s, 3H), 2.99 – 2.91 (m, 1H), 2.51 – 2.40 (m, 2H), 1.78 – 1.00 (m, 147H), 0.93 – 0.80 (m, 9H), 0.69 – 0.60 (m, 2H), 0.60 – 0.52 (m, 1H), -0.33 (q, *J* = 4.9 Hz, 1H).**$^{13}$C-NMR** (151 MHz, $CDCl_3$) δ 175.31, 138.54, 138.43, 137.87, 137.31, 128.63, 128.55, 128.53, 128.48, 128.27, 128.17, 128.11, 128.09, 128.01, 127.99, 127.81, 102.47, 85.58, 84.69, 82.45, 77.96, 75.88, 75.27, 75.07, 73.03, 72.47, 71.26, 63.01, 57.84, 51.48, 35.81, 35.50, 32.53, 32.08, 30.64, 30.38, 30.13, 30.09, 29.89, 29.86, 29.82, 29.76, 29.73, 29.63, 29.52, 28.88, 27.73, 27.68, 26.31, 26.03, 22.84, 15.93, 15.04, 14.27, 11.08. **HRMS (ESI)** Calcd. for $C_{119}H_{206}O_9N$ ($[M + NH_4]^+$): 1793.5687, found: 1793.5704.

Deprotection:
Compound **32b** (50 mg, 35 µmol, α:β = 3:2) was obtained in 90% isolated yield by subjecting the intermediate from the esterification reaction to the aforementioned deprotection conditions.

**$^{1}$H-NMR** (400 MHz $CDCl_3$/MeOD 4:1) δ 5.00 (d, *J* = 3.8 Hz, 0.6H), 4.39 – 4.26 (m, 1.4H), 4.17 – 4.08 (m, 1H), 3.88 – 3.80 (m, 0.6H), 3.59 – 3.45 (m, 2.4H), 3.40 – 3.31 (m, 0.4H), 3.30 – 3.16 (m, 4.6H), 3.09 – 3.00 (m, 0.4H), 2.90 – 2.81 (m, 1H), 2.34 – 2.24 (m, 1H), 1.55 – 0.81 (m, 147H), 0.80 – 0.65 (m, 9H), 0.56 – 0.45 (m, 2H), 0.46 – 0.37 (m, 1H), -0.48 (q, *J* = 5.0 Hz, 1H). **$^{13}$C-NMR** (151 MHz, $CDCl_3$/MeOD 4:1) δ 175.26, 175.22, 96.61, 92.35, 85.65, 76.24, 74.53, 73.73, 73.49, 72.47, 72.44, 72.22, 70.53, 70.38, 69.27, 63.58, 57.55, 52.83, 52.70, 35.31, 34.90, 32.33, 31.85, 30.43, 30.15, 30.14, 29.87, 29.80, 29.67, 29.65, 29.62, 29.59, 29.58, 29.47, 29.37, 29.28, 29.11, 29.08, 28.65, 27.42, 27.35, 27.32, 25.99, 25.36, 25.33, 22.60, 15.70, 14.68, 13.93, 10.80. **HRMS (ESI)** Calcd. for $C_{91}H_{182}O_9N$ ($[M + NH_4]^+$): 1433.3809, found: 1433.3837.

**Compound 32c:**

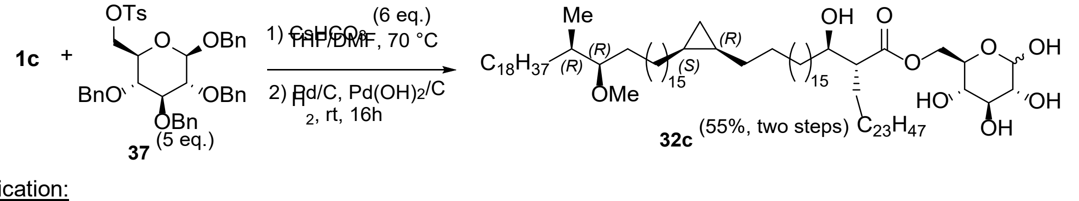


Esterification:
The reaction was performed in 58% isolated yield by subjecting **1c** (85 mg, 68 µmol), **37** (235 mg, 0.34 mmol, 5.0 equiv.) and anhydrous $CsHCO_3$ (79 mg, 0.41 mmol, 6.0 equiv.) to the aforementioned procedure.

**$^{1}$H-NMR** (400 MHz $CDCl_3$) δ 7.38 – 7.24 (m, 20H), 4.98 – 4.86 (m, 4H), 4.78 (d, *J* = 10.9 Hz, 1H), 4.70 (d, *J* = 10.9 Hz, 1H), 4.66 – 4.57 (m, 2H), 4.57 – 4.49 (m, 2H), 4.21 (dd, *J* = 11.7, 4.6 Hz, 1H), 3.70 – 3.61 (m, 2H), 3.57 – 3.45 (m, 3H), 3.34 (s, 3H), 2.99 – 2.92 (m, 1H), 2.51 – 2.43 (m, 2H), 1.78 – 1.03 (m, 147H), 0.93 – 0.82 (m, 9H), 0.69 – 0.60 (m, 2H), 0.59 – 0.52 (m, 1H), -0.33 (q, *J* = 4.9 Hz, 1H).**$^{13}$C-NMR** (151 MHz, $CDCl_3$) δ 175.34, 138.52, 138.41, 137.85, 137.30, 128.64, 128.56, 128.54, 128.49, 128.30, 128.28, 128.17, 128.15, 128.12, 128.11, 128.02, 127.99, 127.83, 102.47, 85.58,

84.69, 82.44, 77.94, 75.90, 75.28, 75.08, 75.04, 73.01, 72.47, 71.27, 63.01, 57.85, 51.47, 35.80, 35.47, 32.52, 32.08, 30.63, 30.39, 30.13, 30.09, 29.89, 29.86, 29.82, 29.76, 29.73, 29.63, 29.52, 28.88, 27.73, 27.68, 26.31, 26.03, 22.85, 15.93, 15.04, 14.28, 11.07. **HRMS (ESI)** Calcd. for $C_{119}H_{206}O_9N$ ($[M + NH_4]^+$): 1793.5687, found: 1793.5702.

Deprotection:
Compound **32c** (50 mg, 35 µmol, α:β = 1:1) was obtained in 94% isolated yield by subjecting the intermediate from the esterification reaction to the aforementioned deprotection conditions.

**$^1$H-NMR** (400 MHz CDCl$_3$/MeOD 4:1) δ 4.99 (d, *J* = 3.8 Hz, 0.5H), 4.39 – 4.25 (m, 1.5H), 4.11 (dt, *J* = 11.8, 5.9 Hz, 1H), 3.88 – 3.80 (m, 0.5H), 3.63 – 3.46 (m, 2H), 3.40 – 3.32 (m, 0.5H), 3.30 – 3.14 (m, 4.5H), 3.05 (t, *J* = 8.3 Hz, 0.5H), 2.89 – 2.80 (m, 1H), 2.33 – 2.22 (m, 1H), 1.54 – 0.85 (m, 147H), 0.79 – 0.63 (m, 9H), 0.56 – 0.44 (m, 2H), 0.45 – 0.37 (m, 1H), -0.48 (q, *J* = 4.9 Hz, 1H). **$^{13}$C-NMR** (151 MHz, CDCl$_3$/MeOD 4:1) δ 175.27, 175.22, 96.59, 92.34, 85.65, 76.22, 74.51, 73.71, 73.47, 72.46, 72.43, 72.20, 70.50, 70.35, 69.25, 63.56, 57.54, 52.83, 52.70, 35.28, 34.89, 32.31, 31.85, 30.40, 30.14, 29.86, 29.79, 29.66, 29.62, 29.58, 29.47, 29.35, 29.28, 29.10, 29.07, 28.64, 27.41, 27.35, 27.32, 25.98, 25.35, 25.32, 22.59, 15.69, 14.68, 13.93, 10.78. **HRMS (ESI)** Calcd. for $C_{91}H_{182}O_9N$ ($[M + NH_4]^+$): 1433.3809, found: 1433.3830.

**Compound 32d:**

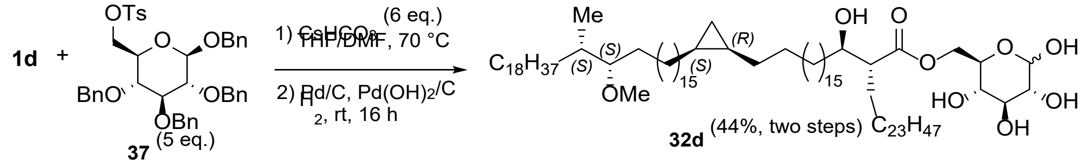


Esterification:
The reaction was performed in 51% isolated yield by subjecting **1d** (75 mg, 60 µmol), **37** (208 mg, 0.30 mmol, 5.0 equiv.) and anhydrous $CsHCO_3$ (70 mg, 0.36 mmol, 6.0 equiv.) to the aforementioned procedure.

**$^1$H-NMR** (400 MHz, CDCl$_3$) δ 7.40 – 7.23 (m, 20H), 5.00 – 4.84 (m, 4H), 4.78 (d, *J* = 10.9 Hz, 1H), 4.70 (d, *J* = 10.9 Hz, 1H), 4.66 – 4.56 (m, 2H), 4.56 – 4.48 (m, 2H), 4.22 (dd, *J* = 11.7, 4.6 Hz, 1H), 3.72 – 3.60 (m, 2H), 3.57 – 3.44 (m, 3H), 3.34 (s, 3H), 3.01 – 2.91 (m, 1H), 2.52 – 2.41 (m, 2H), 1.77 – 1.00 (m, 147H), 0.95 – 0.82 (m, 9H), 0.69 – 0.60 (m, 2H), 0.60 – 0.52 (m, 1H), -0.33 (q, *J* = 4.9 Hz, 1H). **HRMS (ESI)** Calcd. for $C_{119}H_{206}O_9N$ ($[M + NH_4]^+$): 1793.5687, found: 1793.5722.

Deprotection:
Compound **32d** (37 mg, 26 µmol, α:β = 3:2) was obtained in 87% isolated yield by subjecting the intermediate from the esterification reaction to the aforementioned deprotection conditions.

**$^1$H-NMR** (400 MHz, CDCl$_3$/MeOD 4:1) δ 5.01 (d, *J* = 3.7 Hz, 0.6H), 4.39 – 4.27 (m, 1.4H), 4.18 – 4.08 (m, 1H), 3.85 – 3.82 (m, 0.6H), 3.61 – 3.46 (m, 2H), 3.41 – 3.33 (m, 0.6H), 3.32 – 3.15 (m, 4.6H), 3.07 (t, *J* = 8.3 Hz, 0.4H), 2.91 – 2.81 (m, 1H), 2.35 – 2.25 (m, 1H), 1.57 – 0.87 (m, 147H), 0.80 – 0.67 (m, 9H), 0.57 – 0.47 (m, 2H), 0.47 – 0.39 (m, 1H), -0.47 (q, *J* = 5.0 Hz, 1H). **$^{13}$C-NMR** (151 MHz, CDCl$_3$/MeOD 4:1) δ 175.23, 175.19, 96.58, 92.32, 85.63, 76.23, 74.51, 73.70, 73.45, 72.40, 72.38, 72.20, 70.47, 70.34, 69.21, 63.54, 63.51, 57.49, 52.79, 52.67, 35.26, 34.84, 32.29, 31.81, 30.38, 30.09, 29.81, 29.74, 29.61, 29.59, 29.57, 29.53, 29.51, 29.42, 29.31, 29.30, 29.24, 29.04, 29.01, 28.61, 27.36, 27.30, 27.27, 25.93, 25.32, 25.29, 22.55, 15.66, 14.62, 13.86, 10.74. **HRMS (ESI)** Calcd. for $C_{91}H_{182}O_9N$ ($[M + NH_4]^+$): 1433.3809, found: 1433.3840.

**Compound 33a:**

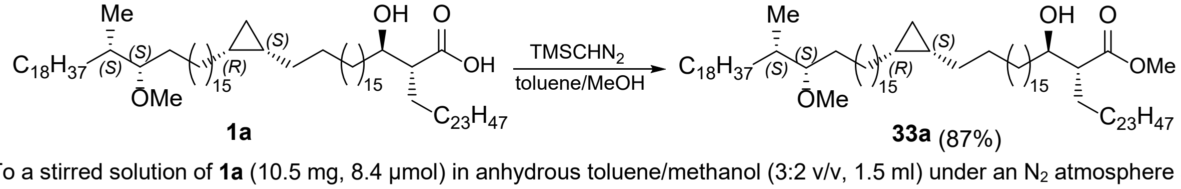


To a stirred solution of **1a** (10.5 mg, 8.4 µmol) in anhydrous toluene/methanol (3:2 v/v, 1.5 ml) under an $N_2$ atmosphere at rt, was added trimethylsilyldiazomethane (2 M in ether) until the yellow color persisted (around 4 drops). After 5 min, TLC (2% MeOH in $CH_2Cl_2$) indicated full conversion of the acid, after which AcOH was added until the reaction mixture became colorless. The reaction mixture was concentrated *in vacuo*, and purified by flash column chromatography using 2.5-5% EtOAc in pentane, yielding the product (9.2 mg, 7.3 µmol, 87%) as a colorless oil that solidified upon standing.

**$[\alpha]_D^{20}$** = 0.0° (CHCl$_3$, c = 0.46), $[\Phi]_D^{20}$ = 0.0° (CHCl$_3$, c = 0.46). **$^1$H-NMR** (400 MHz CDCl$_3$) δ 3.71 (s, 3H), 3.68 – 3.60 (m, 1H), 3.34 (s, 3H), 3.00 – 2.90 (m, 1H), 2.48 – 2.35 (m, 2H), 1.76 – 1.58 (m, 3H), 1.52 – 1.01 (m, 144H), 0.96 – 0.80 (m, 9H), 0.69 – 0.59 (m, 2H), 0.60 – 0.50 (m, 1H), -0.34 (q, *J* = 4.9 Hz, 1H). **$^{13}$C-NMR** (151 MHz, CDCl$_3$) δ 176.39, 85.60,

72.45, 57.87, 51.67, 51.08, 35.87, 35.48, 32.52, 32.09, 30.64, 30.39, 30.14, 30.10, 29.91, 29.87, 29.83, 29.83, 29.80, 29.77, 29.74, 29.73, 29.71, 29.66, 29.59, 29.53, 28.88, 27.74, 27.58, 26.32, 25.89, 22.85, 15.94, 15.05, 14.28, 11.07. **HRMS (ESI)** Calcd. for $C_{86}H_{170}O_4Na$ ([M + Na]$^+$): 1290.2991, found: 1290.3031.

**Compound 33b:**

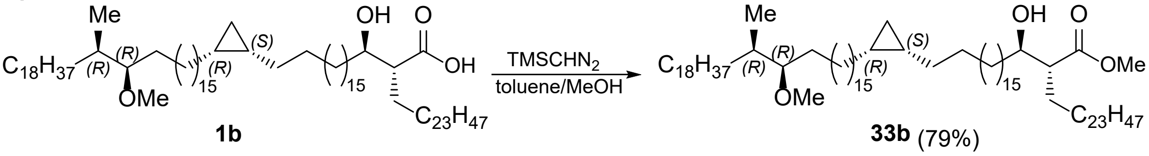

Compound **33b** (7.7 mg, 6.1 μmol) was obtained in 79% isolated yield by subjecting **1b** (9.7 mg, 7.7 μmol) to the aforementioned procedure.

**[α]$_D$$^{20}$** = +7.8° ($CHCl_3$, c = 0.39), [Φ]$_D$$^{20}$ = +99° ($CHCl_3$, c = 0.39). **$^1$H-NMR** (400 MHz $CDCl_3$) δ 3.71 (s, 3H), 3.69 – 3.60 (m, 1H), 3.34 (s, 3H), 3.00 – 2.90 (m, 1H), 2.52 – 2.26 (m, 2H), 1.76 – 1.57 (m, 3H), 1.51 – 1.00 (m, 144H), 0.93 – 0.76 (m, 9H), 0.69 – 0.59 (m, 2H), 0.60 – 0.50 (m, 1H), -0.34 (q, *J* = 5.0 Hz, 1H). **$^{13}$C-NMR** (151 MHz, $CDCl_3$) δ176.39, 85.60, 72.46, 57.87, 51.67, 51.09, 35.86, 35.48, 32.52, 32.09, 30.64, 30.39, 30.14, 30.10, 29.91, 29.86, 29.83, 29.82, 29.80, 29.79, 29.77, 29.74, 29.73, 29.71, 29.66, 29.59, 29.52, 28.88, 27.74, 27.58, 26.32, 25.89, 22.85, 11.07. **HRMS (ESI)** Calcd. for $C_{86}H_{170}O_4Na$ ([M + Na]$^+$): 1290.2991, found: 1290.3050.

**Compound 33c:**

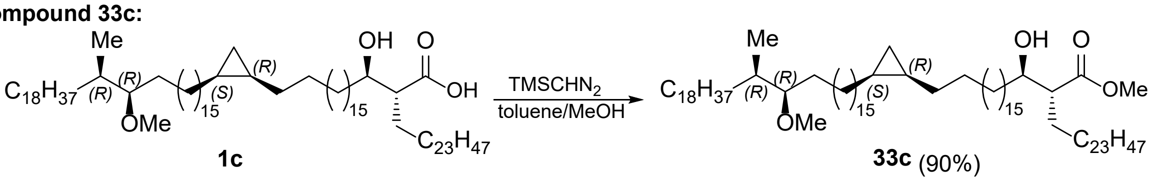

Compound **33c** (8.6 mg, 6.7 μmol) was obtained in 90% isolated yield by subjecting **1c** (9.5 mg, 7.6 μmol) to the aforementioned procedure.

**[α]$_D$$^{20}$** = +7.9° ($CHCl_3$, c = 0.43), [Φ]$_D$$^{20}$ = +100° ($CHCl_3$, c = 0.43). **$^1$H-NMR** (400 MHz $CDCl_3$) δ 3.71 (s, 3H), 3.69 – 3.62 (m, 1H), 3.34 (s, 3H), 2.99 – 2.91 (m, 1H), 2.48 – 2.36 (m, 2H), 1.77 – 1.58 (m, 3H), 1.50 – 1.00 (m, 144H), 0.94 – 0.80 (m, 9H), 0.69 – 0.60 (m, 2H), 0.59 – 0.50 (m, 1H), -0.34 (q, J = 5.0 Hz, 1H). **$^{13}$C-NMR** (151 MHz, $CDCl_3$) δ 176.40, 85.60, 72.46, 57.87, 51.67, 51.09, 35.87, 35.48, 32.52, 32.09, 30.64, 30.39, 30.14, 30.10, 29.91, 29.86, 29.83, 29.82, 29.79, 29.77, 29.74, 29.73, 29.71, 29.66, 29.59, 29.52, 28.88, 27.74, 27.58, 26.32, 25.89, 22.85, 15.94, 15.05, 14.28, 11.07. **HRMS (ESI)** Calcd. for $C_{86}H_{170}O_4Na$ ([M + Na]$^+$): 1290.2991, found: 1290.3016.

**Compound 33d:**

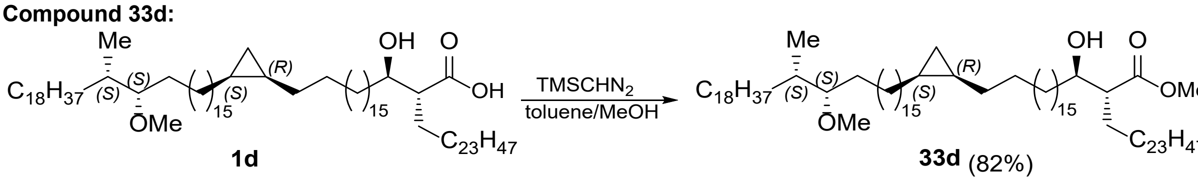

Compound **33d** (9.1 mg, 7.2 μmol) was obtained in 82% isolated yield by subjecting **1c** (11 mg, 8.8 μmol) to the aforementioned procedure.

**[α]$_D$$^{20}$** = 0.0° ($CHCl_3$, c = 0.46), [Φ]$_D$$^{20}$ = 0.0° ($CHCl_3$, c = 0.46). **$^1$H-NMR** (400 MHz $CDCl_3$) δ 3.71 (s, 3H), 3.68 – 3.62 (m, 1H), 3.34 (s, 3H), 2.98 – 2.92 (m, 1H), 2.47 – 2.37 (m, 2H), 1.76 – 1.57 (m, 3H), 1.51 – 1.03 (m, 144H), 0.91 – 0.81 (m, 9H), 0.68 – 0.60 (m, 2H), 0.59 – 0.52 (m, 1H), -0.34 (q, J = 5.0 Hz, 1H). **$^{13}$C-NMR** (151 MHz, $CDCl_3$) δ 176.39, 85.60, 72.46, 57.87, 51.67, 51.09, 35.86, 35.48, 32.52, 32.09, 30.64, 30.39, 30.14, 30.10, 29.91, 29.86, 29.83, 29.82, 29.81, 29.79, 29.77, 29.74, 29.73, 29.71, 29.66, 29.59, 29.52, 28.88, 27.74, 27.58, 26.32, 25.89, 22.85, 15.94, 15.05, 14.28, 11.07. **HRMS (ESI)** Calcd. for $C_{86}H_{170}O_4Na$ ([M + Na]$^+$): 1290.2991, found: 1290.3010.

## References

[1] D. Ly, A. G. Kasmar, T.-Y. Cheng, A. de Jong, S. Huang, S. Roy, A. Bhatt, R. P. van Summeren, J. D. Altman, W. R. Jacobs Jr., E. J. Adams, A. J. Minnaard, S. A. Porcelli, D. B. Moody, *J. Exp. Med.* **2013**, *210*, 729–741.

[2] I. Van Rhijn, S. K. Iwany, P. Fodran, T.-Y. Cheng, L. Gapin, A. J. Minnaard, D. B. Moody, *Eur. J. Immunol.* **2017**, *47*, 1525–1534.

[3] D. B. Moody, M. R. Guy, E. Grant, T.-Y. Cheng, M. B. Brenner, G. S. Besra, S. A. Porcelli, *J. Exp. Med.* **2000**, *192*, 965–976.

[4] A. G. Kasmar, I. Van Rhijn, K. G. Magalhaes, D. C. Young, T.-Y. Cheng, M. T. Turner, A. Schiefner, R. C. Kalathur, I. A. Wilson, M. Bhati, S. Gras, R. W. Birkinshaw, L. L. Tan, J. Rossjohn, J. Shires, S. Jakobsen, J. D. Altman, D. B. Moody, *J. Immunol.* **2013**, *191*, 4499–4503.

[5] G. E. Keck, E. P. Boden, S. A. Mabury, *J. Org. Chem.* **1985**, *50*, 709–710.

[6] A. B. Charette, H. Lebel, *Org. Synth.* **1999**, *76*, 86.

[7] J. R. Al Dulayymi, M. S. Baird, E. Roberts, M. Deysel, J. Verschoor, *Tetrahedron* **2007**, *63*, 2571–2592.

[8] P. J. L. M. Quaedflieg, B. R. Kesteleyn, P. B. T. P. Wigerinck, N. M. F. Goyvaerts, R. J. Vijn, C. S. M. Liebregts, J. H. M. H. Kooistra, C. Cusan, *Org. Lett.* **2005**, *7*, 5917–5920.

[9] I. Loke, G. Bentzinger, J. Holz, A. Raja, A. Bhasin, F. Sasse, A. Köhn, R. Schobert, S. Laschat, *Org. Biomol. Chem.* **2016**, *14*, 884–894.

[10] N. P. S. Hassan, B. J. Naysmith, J. Sperry, M. A. Brimble, *Tetrahedron* **2015**, *71*, 7137–7143.

[11] J. Zhao, K. Burgess, *Org. Lett.* **2009**, *11*, 2053–2056.

[12] C. Smit, M. W. Fraaije, A. J. Minnaard, *J. Org. Chem.* **2008**, *73*, 9482–9485.

[13] J. Prandi, *Carbohydr. Res.* **2012**, *347*, 151–154.

## NMR spectra

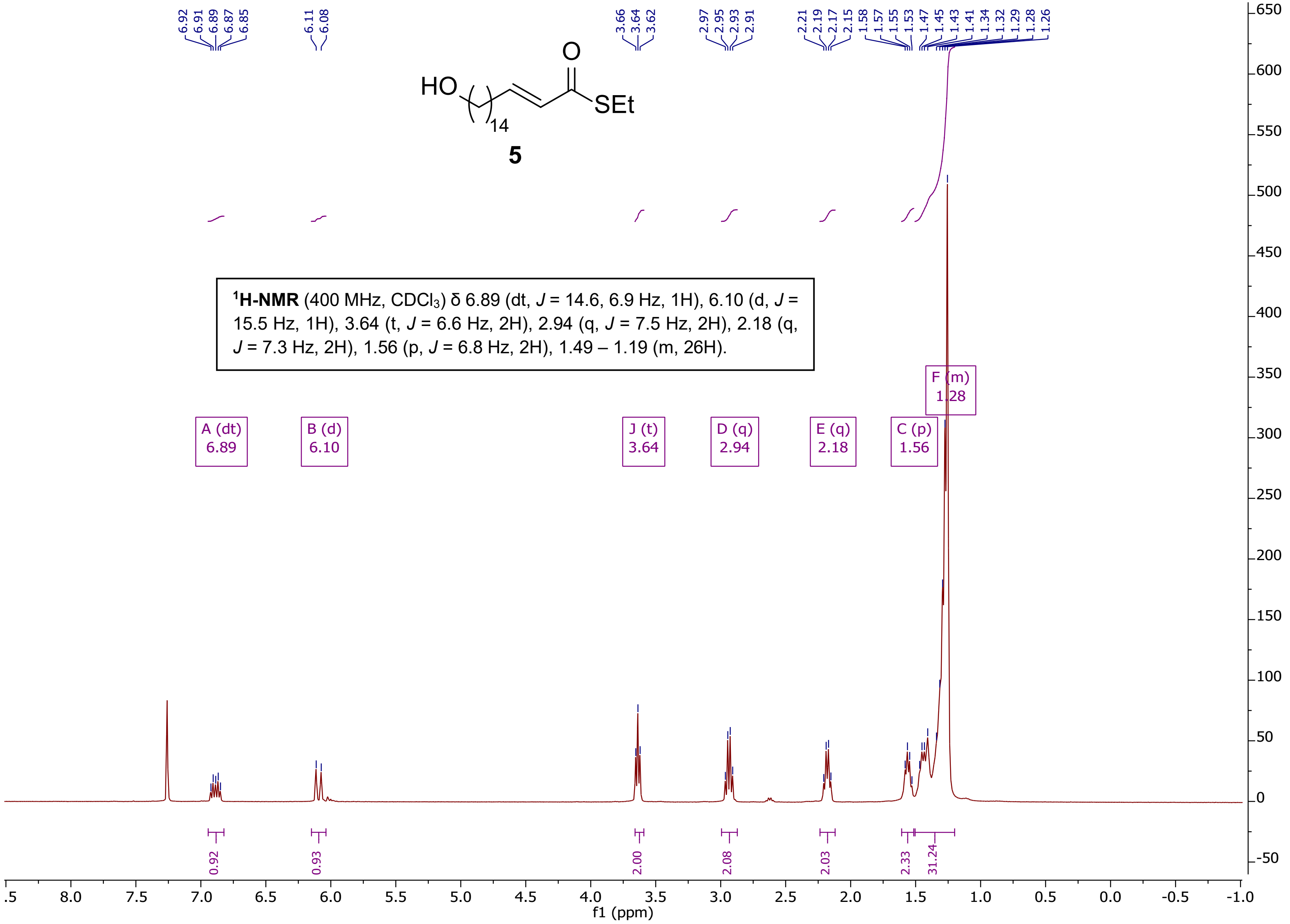


**$^1$H-NMR** (400 MHz, $CDCl_3$) δ 6.89 (dt, *J* = 14.6, 6.9 Hz, 1H), 6.10 (d, *J* = 15.5 Hz, 1H), 3.64 (t, *J* = 6.6 Hz, 2H), 2.94 (q, *J* = 7.5 Hz, 2H), 2.18 (q, *J* = 7.3 Hz, 2H), 1.56 (p, *J* = 6.8 Hz, 2H), 1.49 – 1.19 (m, 26H).

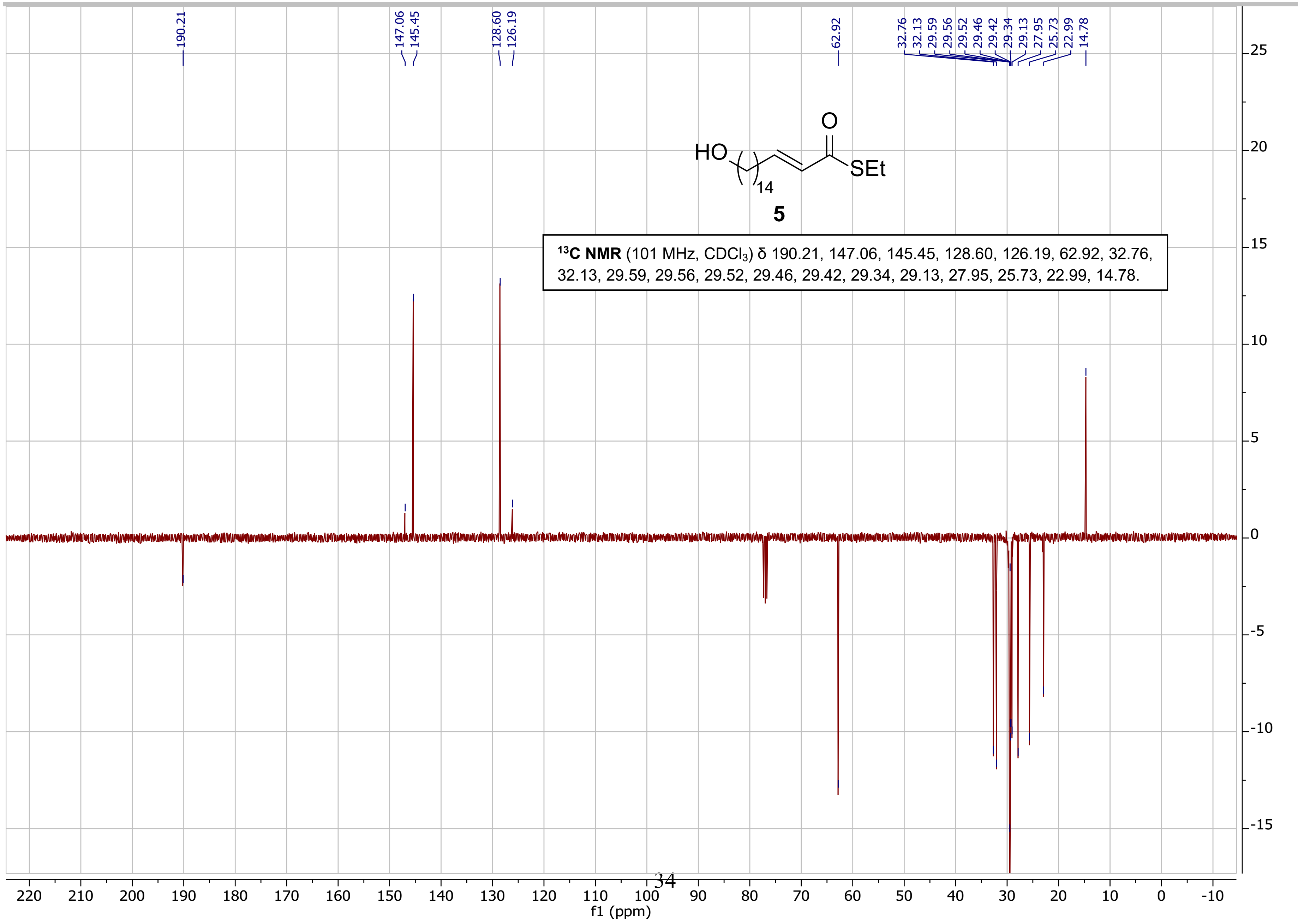


**$^{13}$C NMR** (101 MHz, $CDCl_3$) δ 190.21, 147.06, 145.45, 128.60, 126.19, 62.92, 32.76, 32.13, 29.59, 29.56, 29.52, 29.46, 29.42, 29.34, 29.13, 27.95, 25.73, 22.99, 14.78.

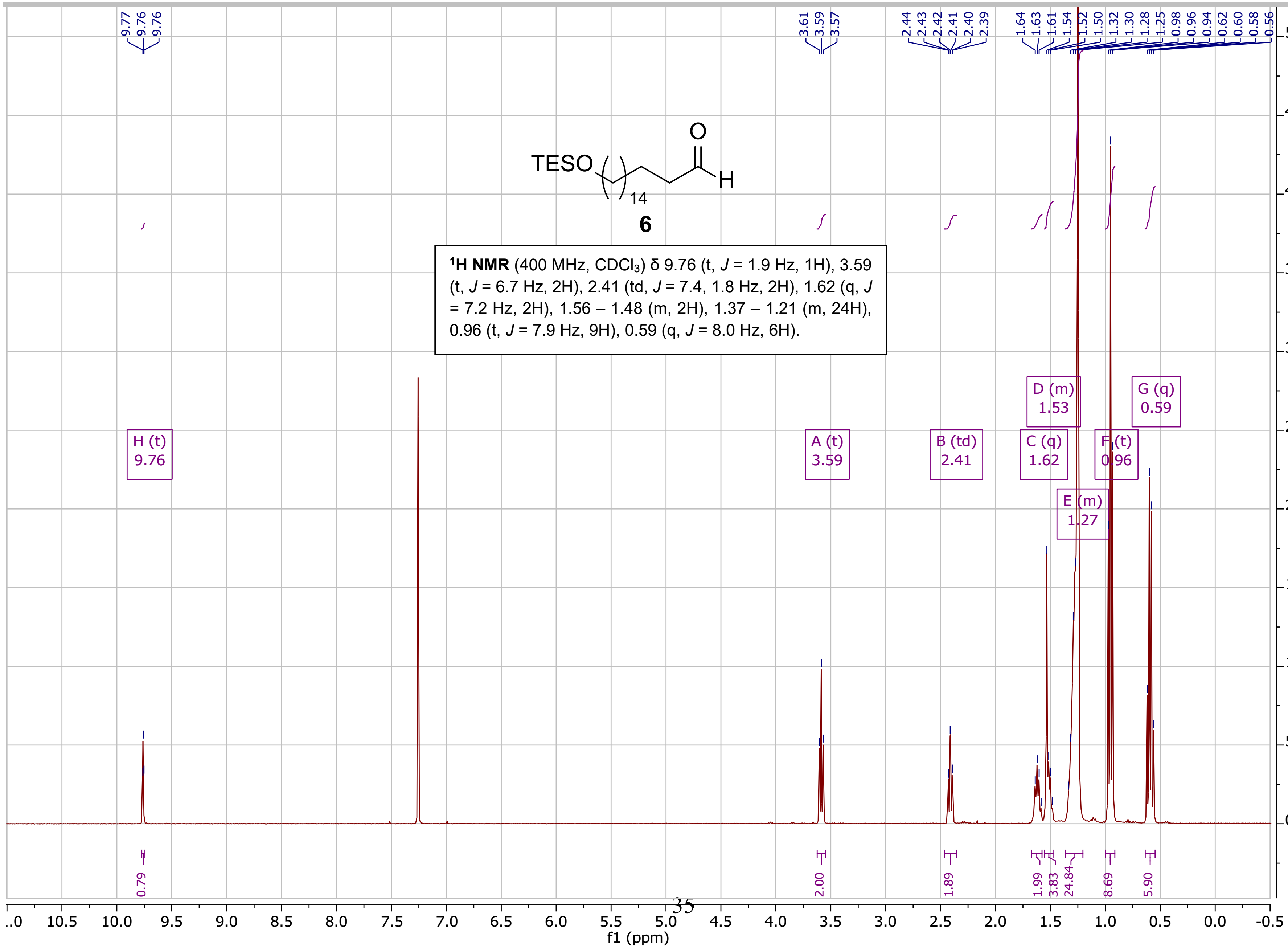


**$^1$H NMR** (400 MHz, $CDCl_3$) δ 9.76 (t, *J* = 1.9 Hz, 1H), 3.59 (t, *J* = 6.7 Hz, 2H), 2.41 (td, *J* = 7.4, 1.8 Hz, 2H), 1.62 (q, *J* = 7.2 Hz, 2H), 1.56 – 1.48 (m, 2H), 1.37 – 1.21 (m, 24H), 0.96 (t, *J* = 7.9 Hz, 9H), 0.59 (q, *J* = 8.0 Hz, 6H).

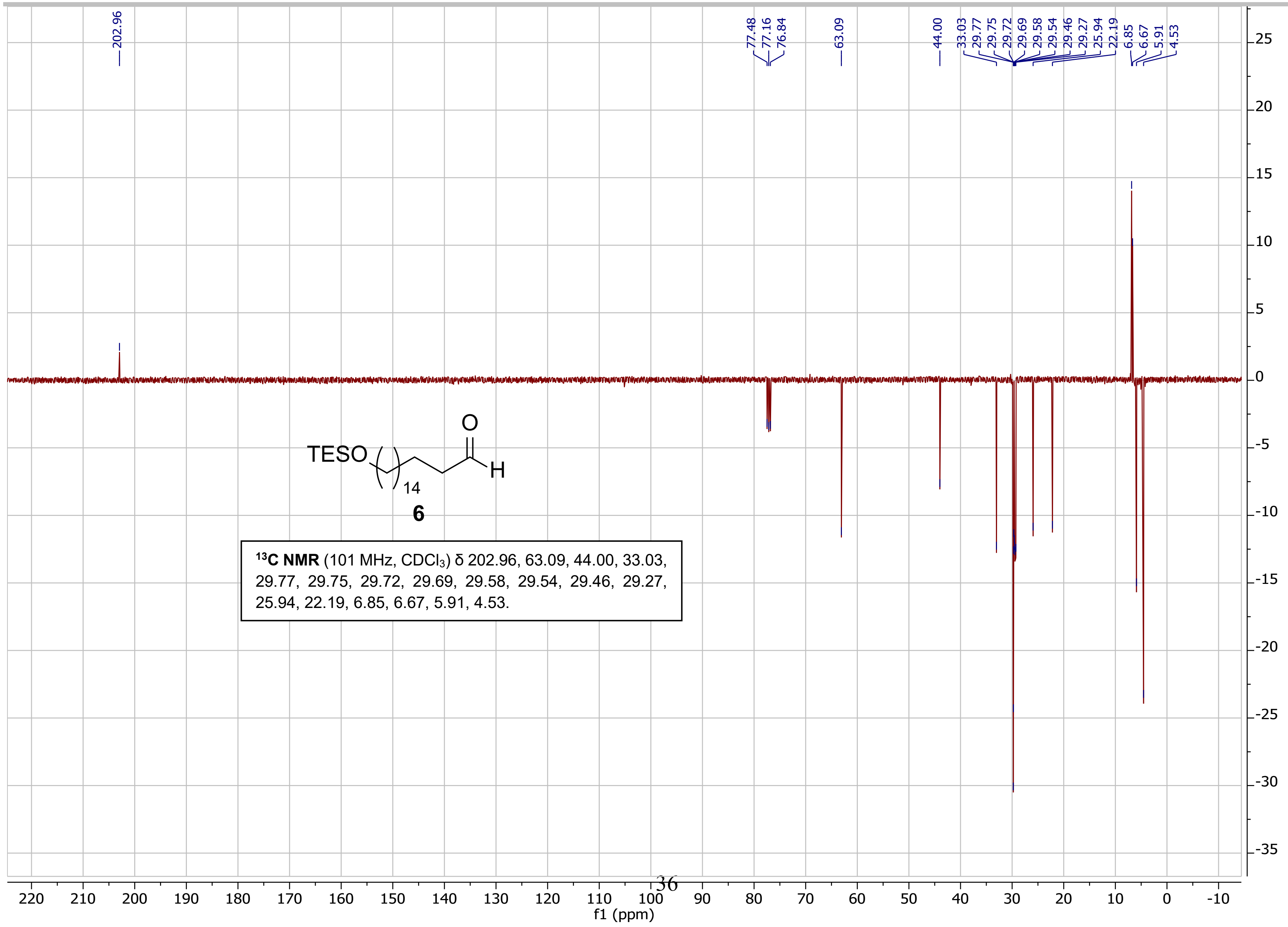


**$^{13}$C NMR** (101 MHz, $CDCl_3$) δ 202.96, 63.09, 44.00, 33.03, 29.77, 29.75, 29.72, 29.69, 29.58, 29.54, 29.46, 29.27, 25.94, 22.19, 6.85, 6.67, 5.91, 4.53.

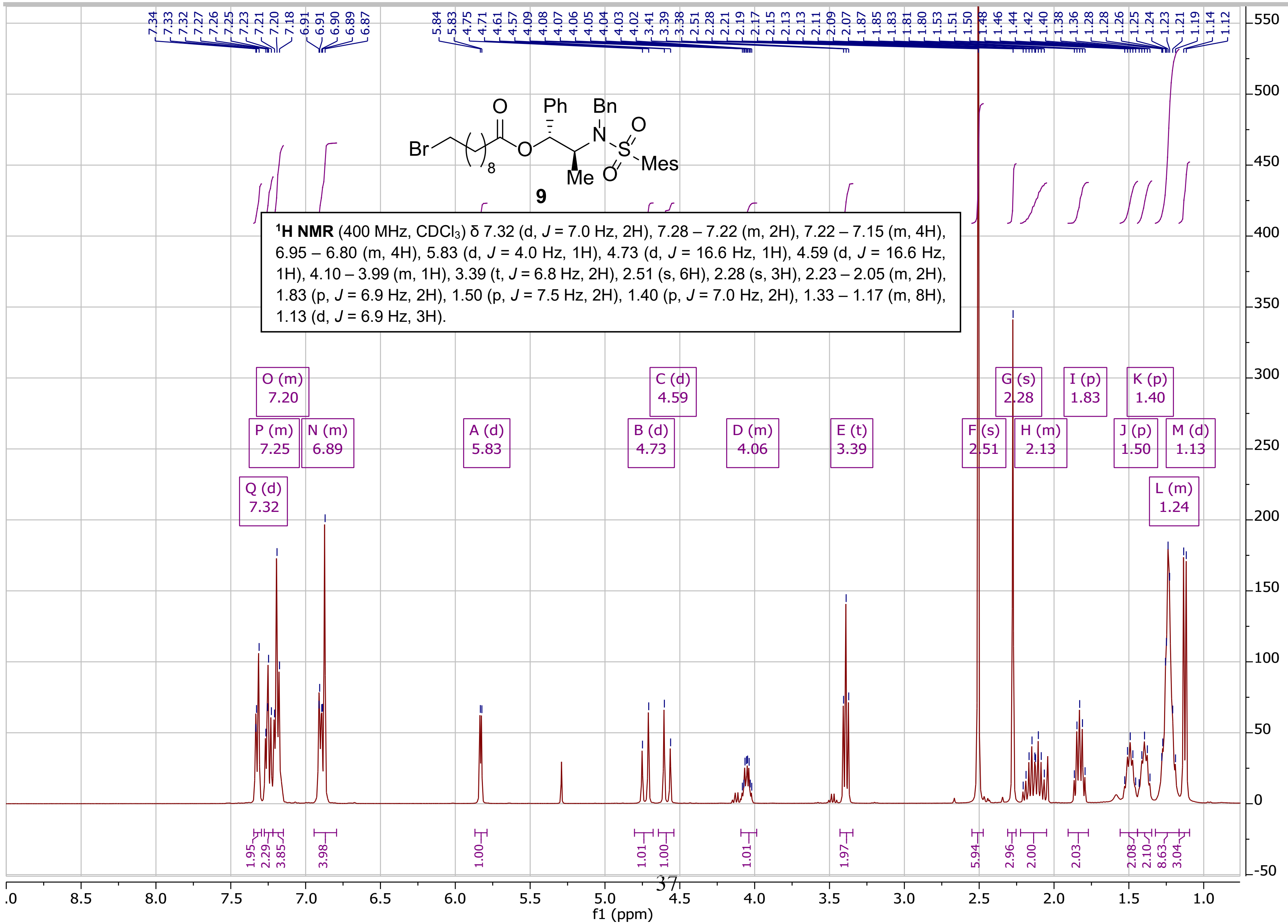

$^{1}$**H NMR** (400 MHz, $CDCl_3$) δ 7.32 (d, *J* = 7.0 Hz, 2H), 7.28 – 7.22 (m, 2H), 7.22 – 7.15 (m, 4H), 6.95 – 6.80 (m, 4H), 5.83 (d, *J* = 4.0 Hz, 1H), 4.73 (d, *J* = 16.6 Hz, 1H), 4.59 (d, *J* = 16.6 Hz, 1H), 4.10 – 3.99 (m, 1H), 3.39 (t, *J* = 6.8 Hz, 2H), 2.51 (s, 6H), 2.28 (s, 3H), 2.23 – 2.05 (m, 2H), 1.83 (p, *J* = 6.9 Hz, 2H), 1.50 (p, *J* = 7.5 Hz, 2H), 1.40 (p, *J* = 7.0 Hz, 2H), 1.33 – 1.17 (m, 8H), 1.13 (d, *J* = 6.9 Hz, 3H).

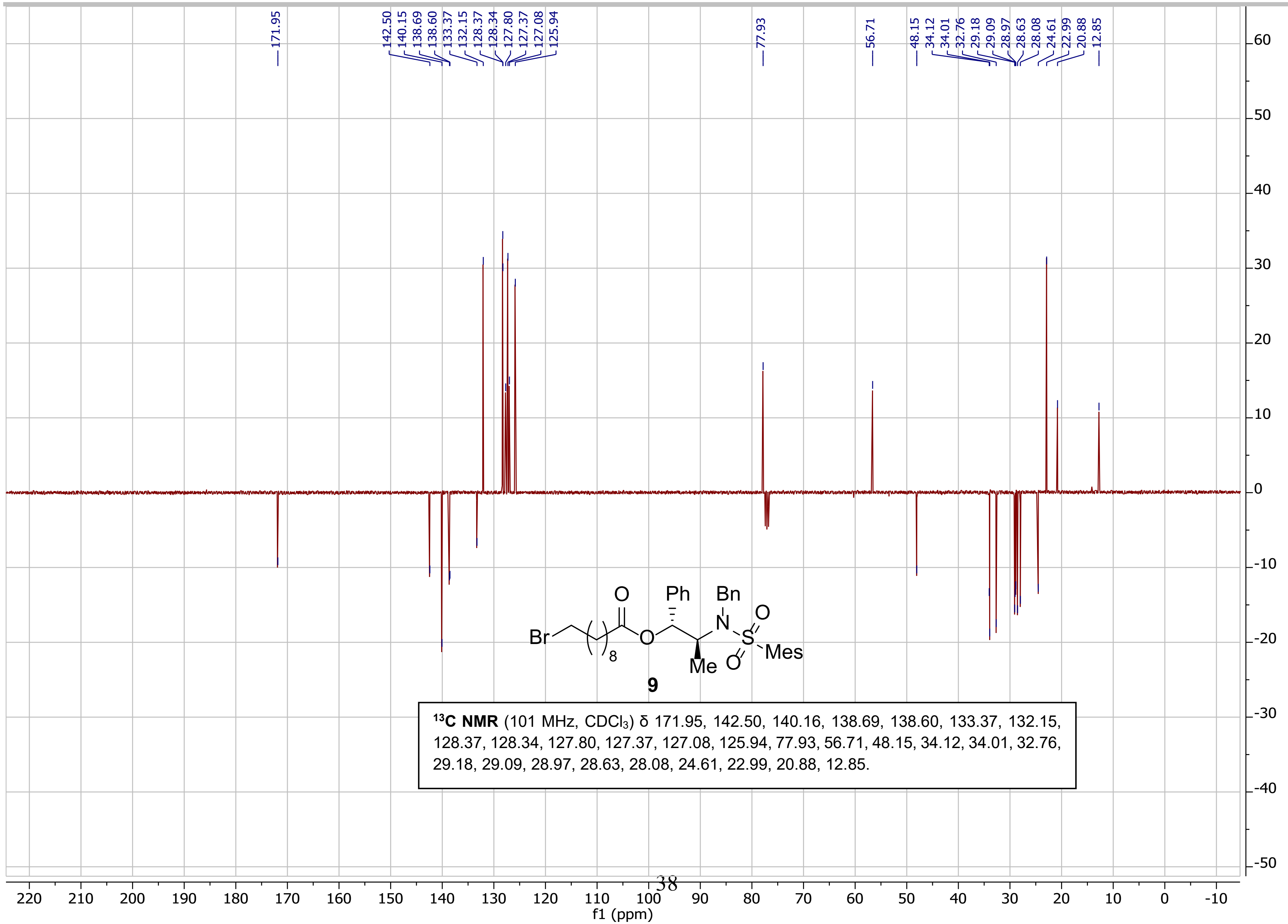

**$^{13}$C NMR** (101 MHz, $CDCl_3$) δ 171.95, 142.50, 140.16, 138.69, 138.60, 133.37, 132.15, 128.37, 128.34, 127.80, 127.37, 127.08, 125.94, 77.93, 56.71, 48.15, 34.12, 34.01, 32.76, 29.18, 29.09, 28.97, 28.63, 28.08, 24.61, 22.99, 20.88, 12.85.

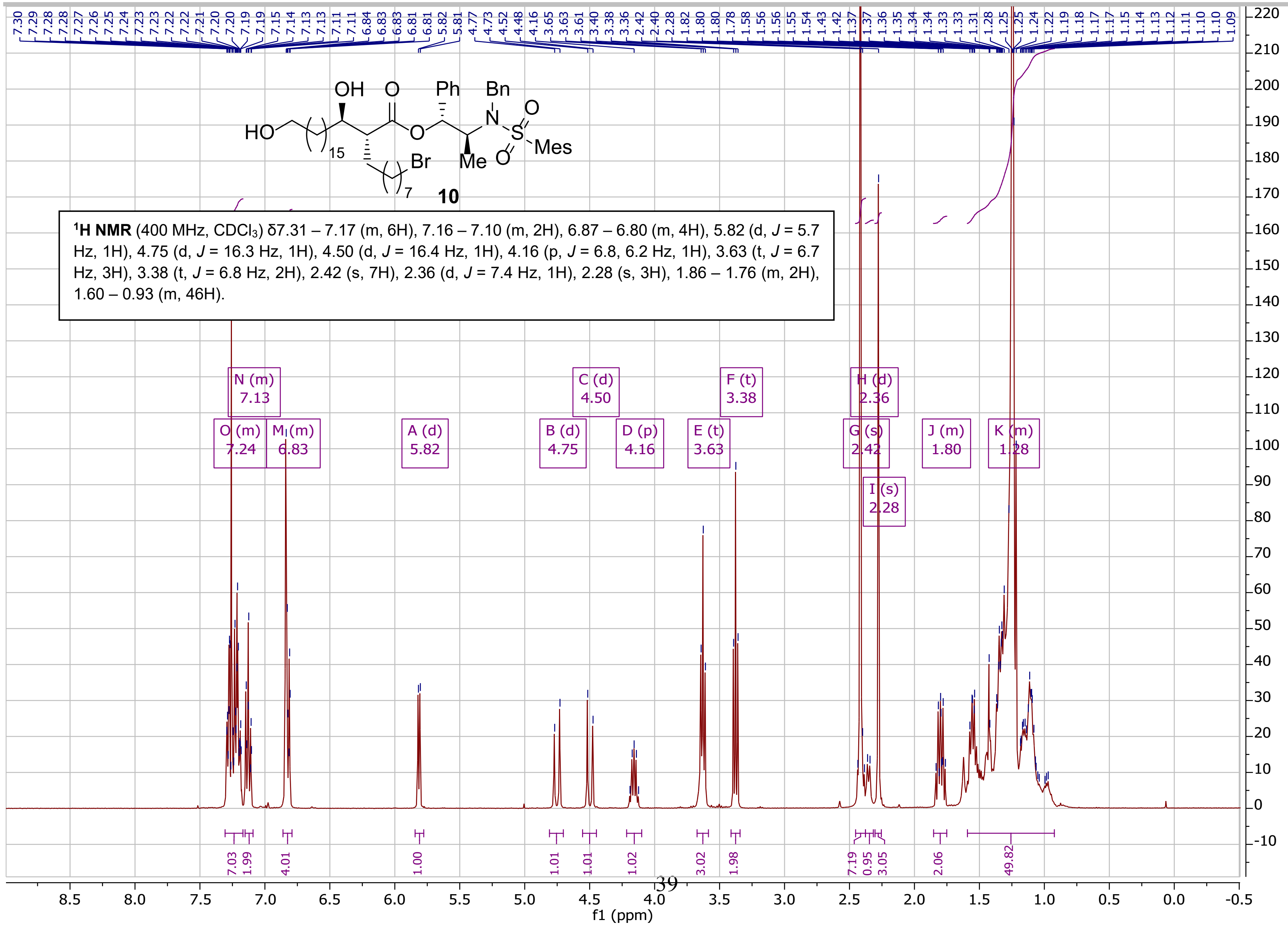


**$^1$H NMR** (400 MHz, $CDCl_3$) δ7.31 – 7.17 (m, 6H), 7.16 – 7.10 (m, 2H), 6.87 – 6.80 (m, 4H), 5.82 (d, $J$ = 5.7 Hz, 1H), 4.75 (d, $J$ = 16.3 Hz, 1H), 4.50 (d, $J$ = 16.4 Hz, 1H), 4.16 (p, $J$ = 6.8, 6.2 Hz, 1H), 3.63 (t, $J$ = 6.7 Hz, 3H), 3.38 (t, $J$ = 6.8 Hz, 2H), 2.42 (s, 7H), 2.36 (d, $J$ = 7.4 Hz, 1H), 2.28 (s, 3H), 1.86 – 1.76 (m, 2H), 1.60 – 0.93 (m, 46H).

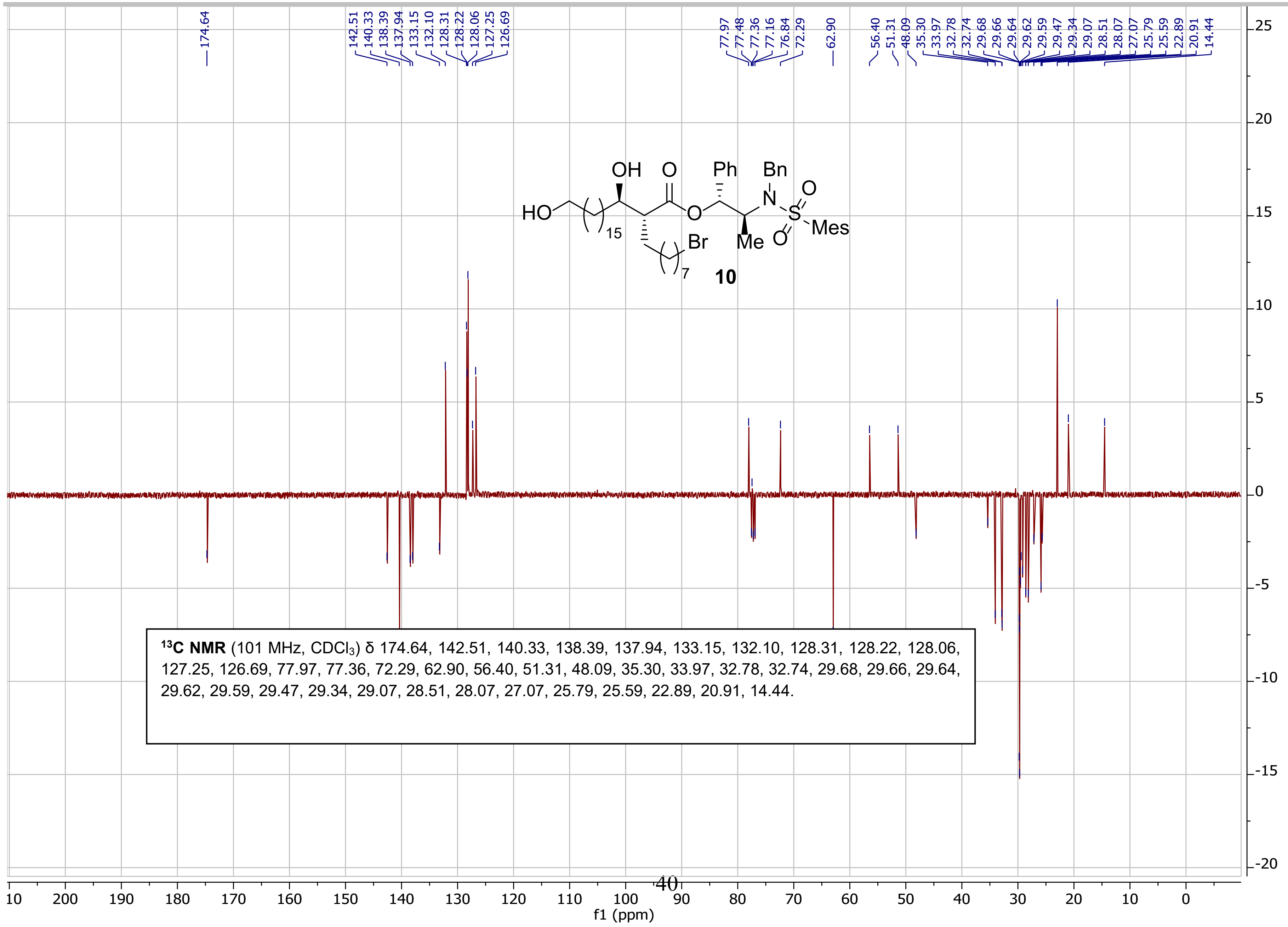

**$^{13}$C NMR** (101 MHz, $CDCl_3$) δ 174.64, 142.51, 140.33, 138.39, 137.94, 133.15, 132.10, 128.31, 128.22, 128.06, 127.25, 126.69, 77.97, 77.36, 72.29, 62.90, 56.40, 51.31, 48.09, 35.30, 33.97, 32.78, 32.74, 29.68, 29.66, 29.64, 29.62, 29.59, 29.47, 29.34, 29.07, 28.51, 28.07, 27.07, 25.79, 25.59, 22.89, 20.91, 14.44.

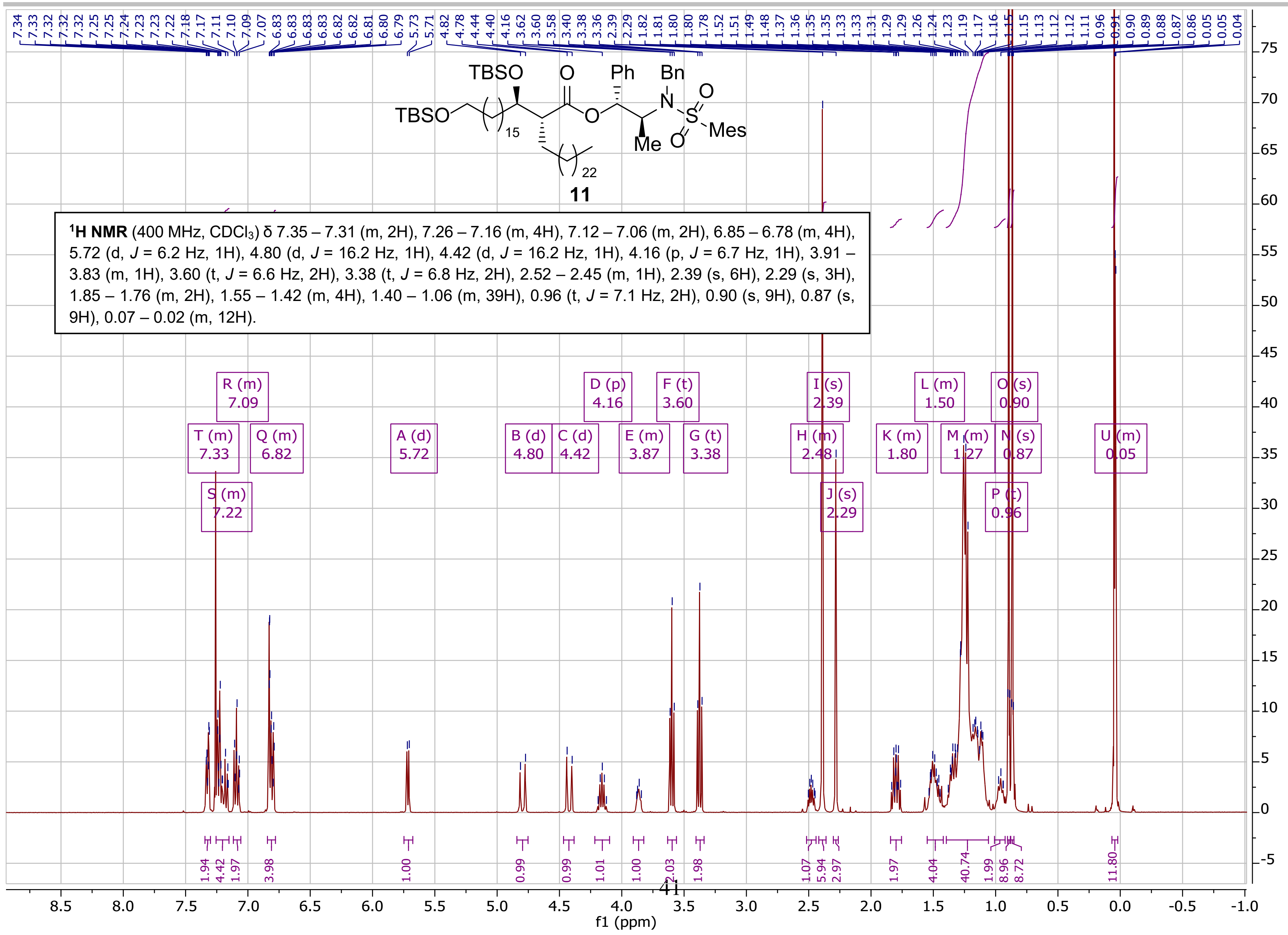

**$^{1}$H NMR** (400 MHz, $CDCl_3$) δ 7.35 – 7.31 (m, 2H), 7.26 – 7.16 (m, 4H), 7.12 – 7.06 (m, 2H), 6.85 – 6.78 (m, 4H), 5.72 (d, *J* = 6.2 Hz, 1H), 4.80 (d, *J* = 16.2 Hz, 1H), 4.42 (d, *J* = 16.2 Hz, 1H), 4.16 (p, *J* = 6.7 Hz, 1H), 3.91 – 3.83 (m, 1H), 3.60 (t, *J* = 6.6 Hz, 2H), 3.38 (t, *J* = 6.8 Hz, 2H), 2.52 – 2.45 (m, 1H), 2.39 (s, 6H), 2.29 (s, 3H), 1.85 – 1.76 (m, 2H), 1.55 – 1.42 (m, 4H), 1.40 – 1.06 (m, 39H), 0.96 (t, *J* = 7.1 Hz, 2H), 0.90 (s, 9H), 0.87 (s, 9H), 0.07 – 0.02 (m, 12H).

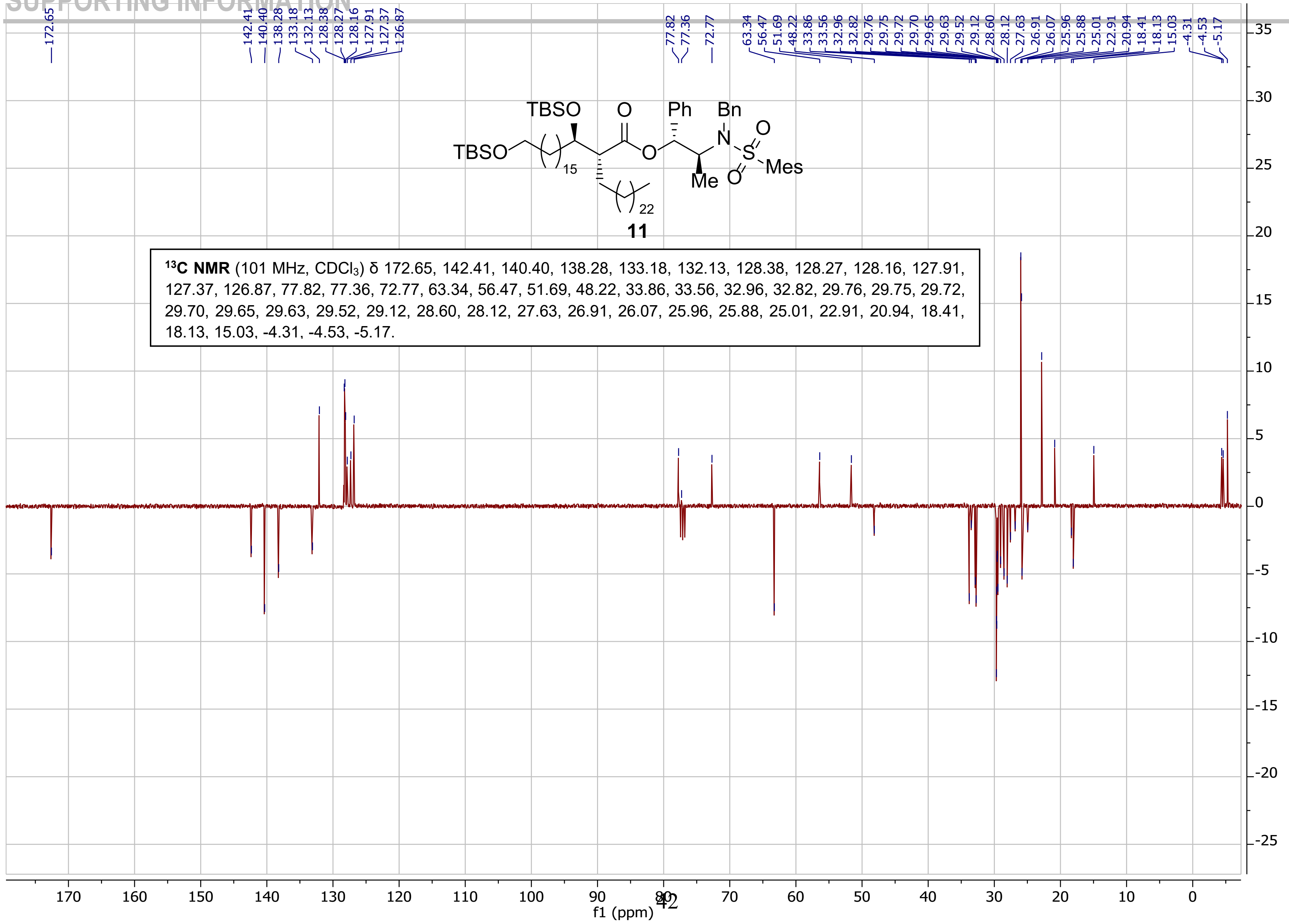

**11**

[13]**C NMR** (101 MHz, $CDCl_3$) δ 172.65, 142.41, 140.40, 138.28, 133.18, 132.13, 128.38, 128.27, 128.16, 127.91, 127.37, 126.87, 77.82, 77.36, 72.77, 63.34, 56.47, 51.69, 48.22, 33.86, 33.56, 32.96, 32.82, 29.76, 29.75, 29.72, 29.70, 29.65, 29.63, 29.52, 29.12, 28.60, 28.12, 27.63, 26.91, 26.07, 25.96, 25.88, 25.01, 22.91, 20.94, 18.41, 18.13, 15.03, -4.31, -4.53, -5.17.

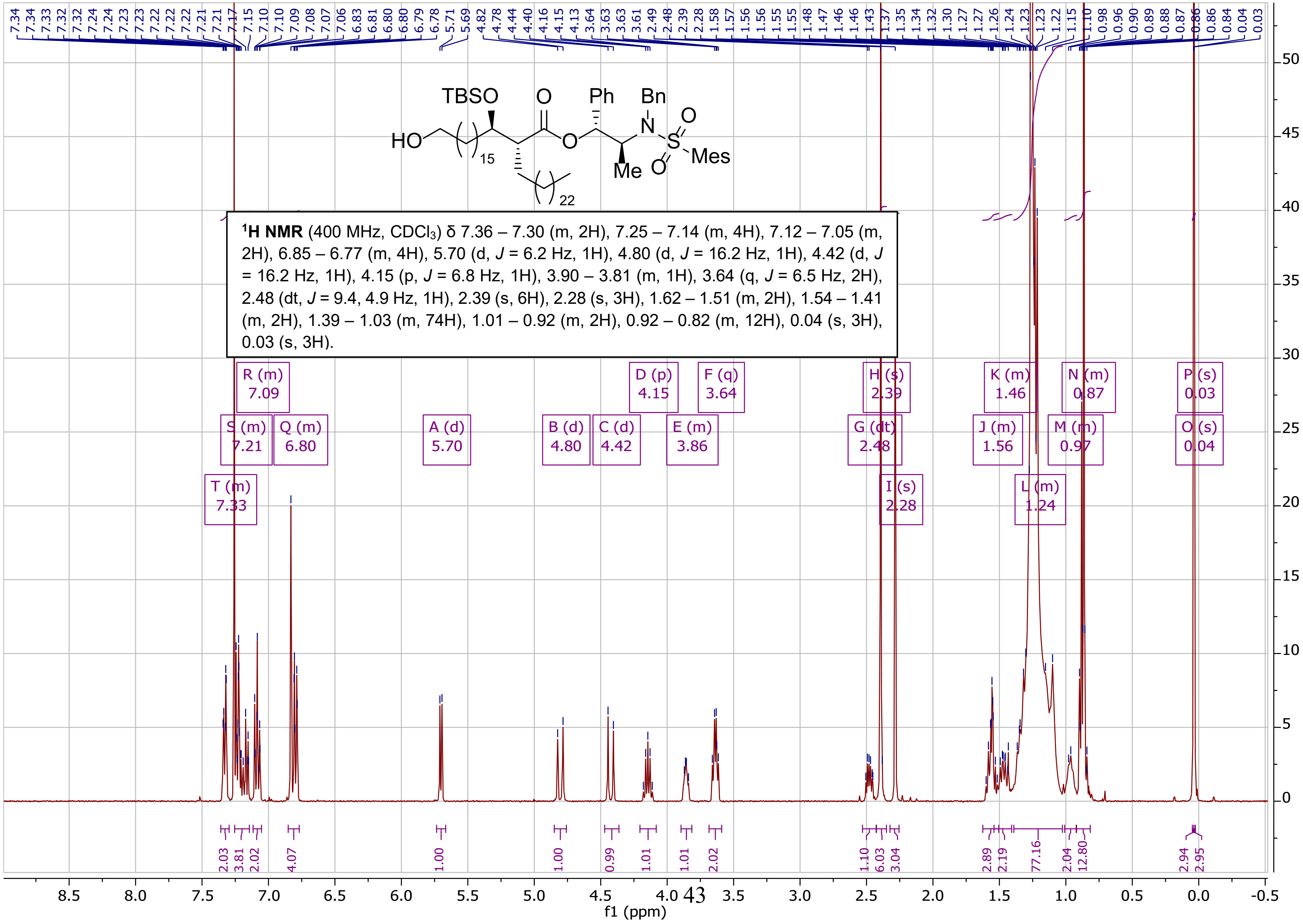
TBSO
O
Ph
Bn
HO
15
22
N
S
O
Mes
Me
$^{1}$H NMR (400 MHz, $CDCl_3$) δ 7.36 – 7.30 (m, 2H), 7.25 – 7.14 (m, 4H), 7.12 – 7.05 (m, 2H), 6.85 – 6.77 (m, 4H), 5.70 (d, J = 6.2 Hz, 1H), 4.80 (d, J = 16.2 Hz, 1H), 4.42 (d, J = 16.2 Hz, 1H), 4.15 (p, J = 6.8 Hz, 1H), 3.90 – 3.81 (m, 1H), 3.64 (q, J = 6.5 Hz, 2H), 2.48 (dt, J = 9.4, 4.9 Hz, 1H), 2.39 (s, 6H), 2.28 (s, 3H), 1.62 – 1.51 (m, 2H), 1.54 – 1.41 (m, 2H), 1.39 – 1.03 (m, 74H), 1.01 – 0.92 (m, 2H), 0.92 – 0.82 (m, 12H), 0.04 (s, 3H), 0.03 (s, 3H).
f1 (ppm)

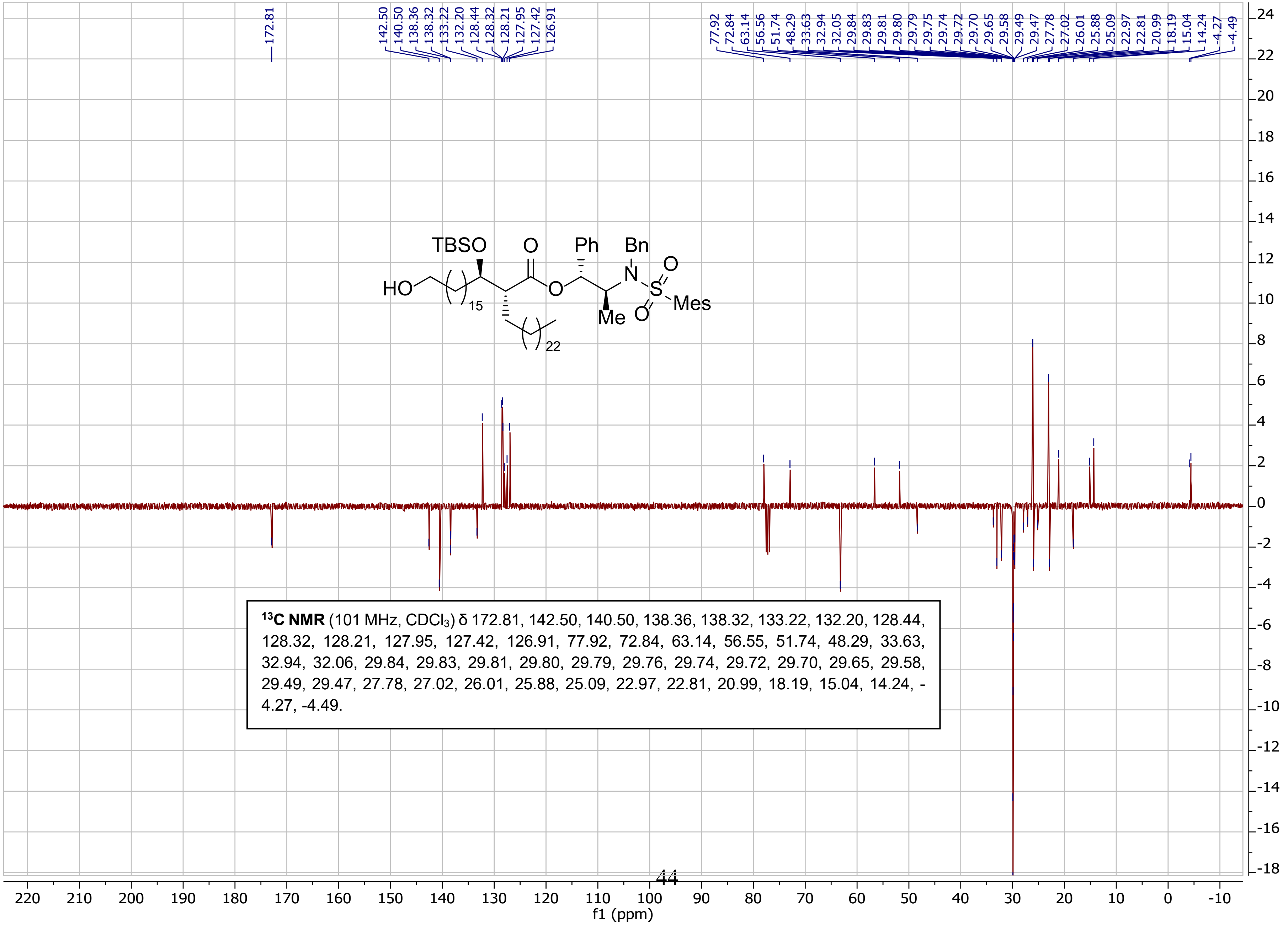

**$^{13}$C NMR** (101 MHz, $CDCl_3$) δ 172.81, 142.50, 140.50, 138.36, 138.32, 133.22, 132.20, 128.44, 128.32, 128.21, 127.95, 127.42, 126.91, 77.92, 72.84, 63.14, 56.55, 51.74, 48.29, 33.63, 32.94, 32.06, 29.84, 29.83, 29.81, 29.80, 29.79, 29.76, 29.74, 29.72, 29.70, 29.65, 29.58, 29.49, 29.47, 27.78, 27.02, 26.01, 25.88, 25.09, 22.97, 22.81, 20.99, 18.19, 15.04, 14.24, -4.27, -4.49.

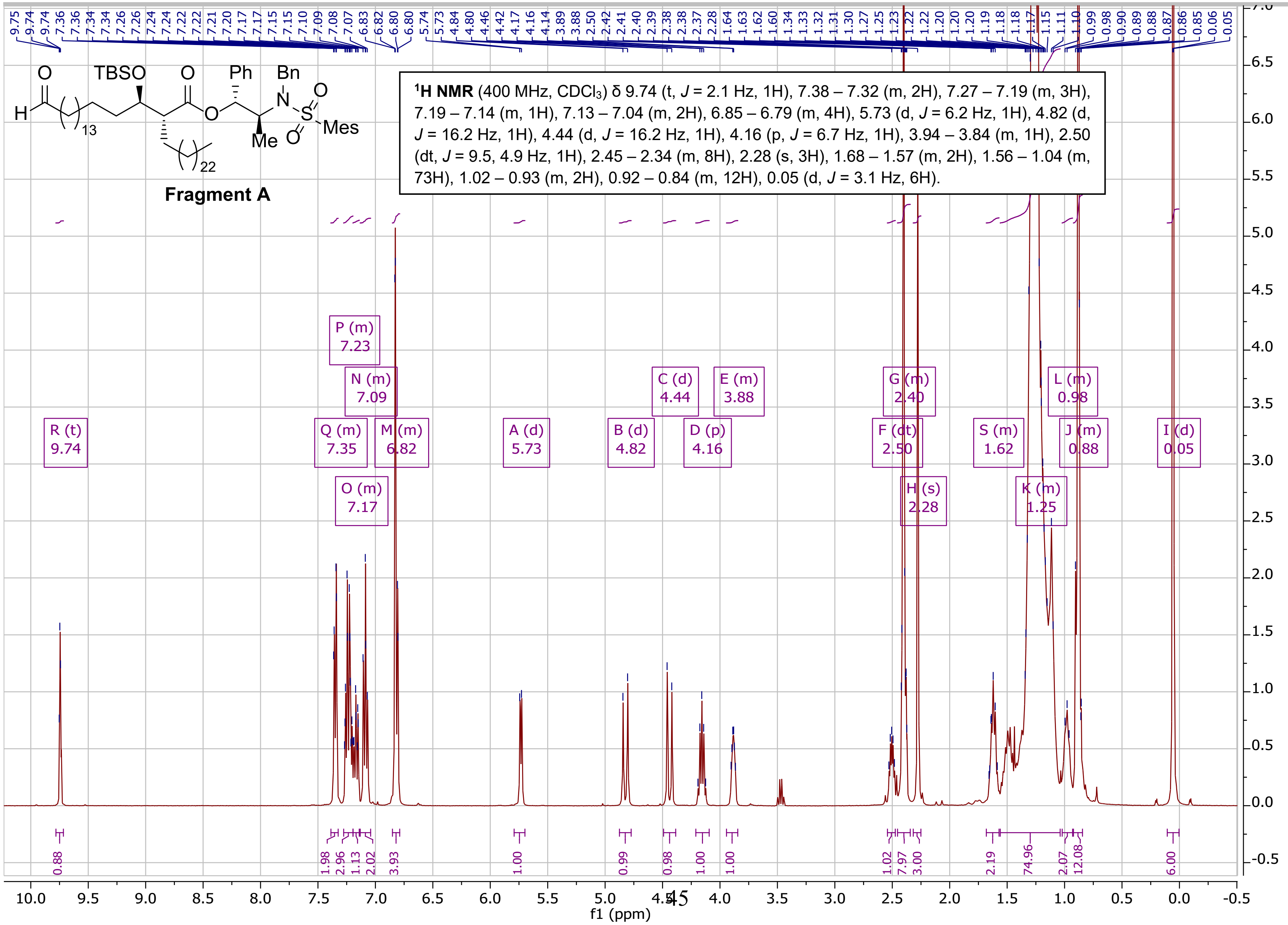


**$^1$H NMR** (400 MHz, $CDCl_3$) δ 9.74 (t, *J* = 2.1 Hz, 1H), 7.38 – 7.32 (m, 2H), 7.27 – 7.19 (m, 3H), 7.19 – 7.14 (m, 1H), 7.13 – 7.04 (m, 2H), 6.85 – 6.79 (m, 4H), 5.73 (d, *J* = 6.2 Hz, 1H), 4.82 (d, *J* = 16.2 Hz, 1H), 4.44 (d, *J* = 16.2 Hz, 1H), 4.16 (p, *J* = 6.7 Hz, 1H), 3.94 – 3.84 (m, 1H), 2.50 (dt, *J* = 9.5, 4.9 Hz, 1H), 2.45 – 2.34 (m, 8H), 2.28 (s, 3H), 1.68 – 1.57 (m, 2H), 1.56 – 1.04 (m, 73H), 1.02 – 0.93 (m, 2H), 0.92 – 0.84 (m, 12H), 0.05 (d, *J* = 3.1 Hz, 6H).

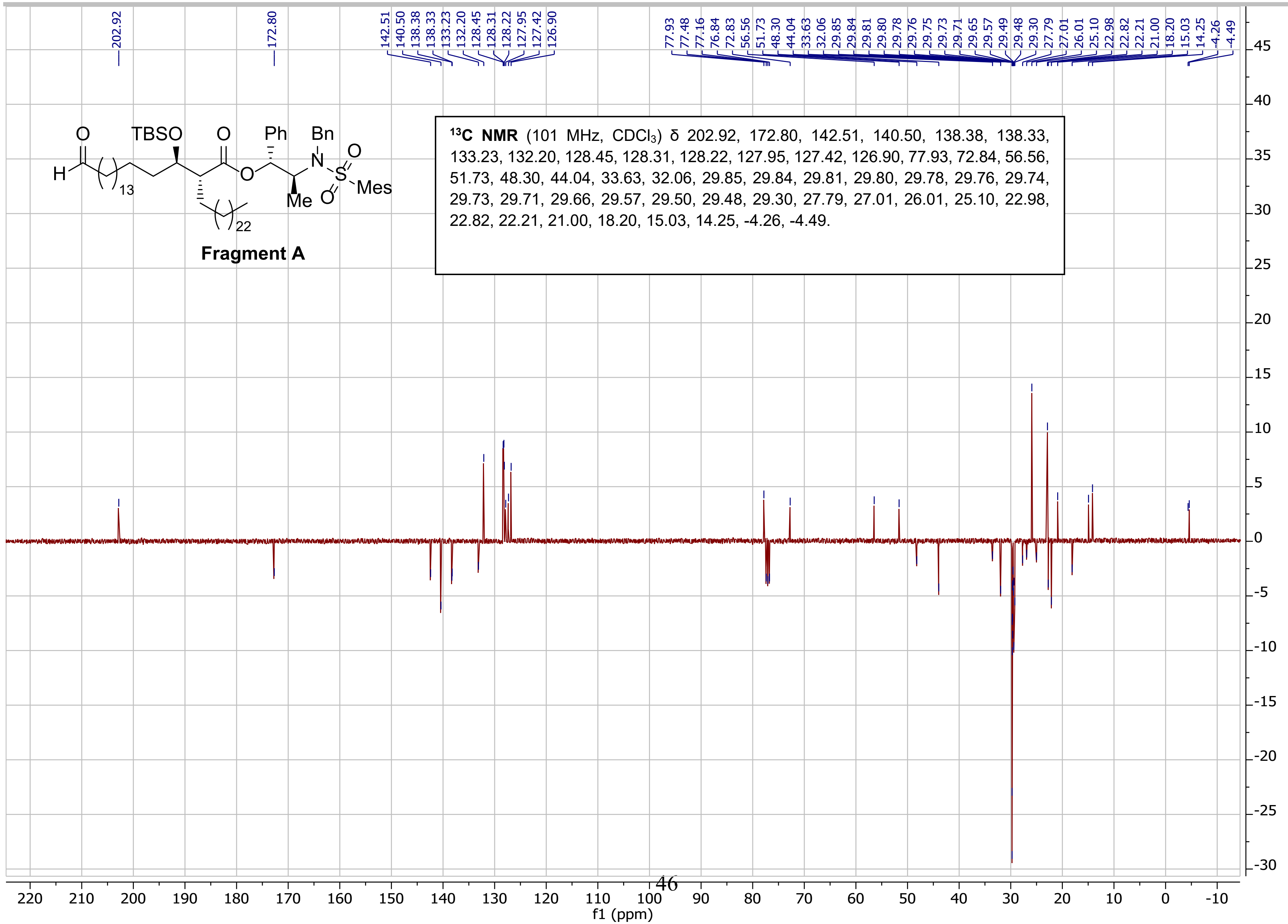
O
TBSO
O
Ph
Bn
H
13
N
O
S
Mes
Me
O
22
Fragment A
¹³C NMR (101 MHz, CDCl₃) δ 202.92, 172.80, 142.51, 140.50, 138.38, 138.33, 133.23, 132.20, 128.45, 128.31, 128.22, 127.95, 127.42, 126.90, 77.93, 72.84, 56.56, 51.73, 48.30, 44.04, 33.63, 32.06, 29.85, 29.84, 29.81, 29.80, 29.78, 29.76, 29.74, 29.73, 29.71, 29.66, 29.57, 29.50, 29.48, 29.30, 27.79, 27.01, 26.01, 25.10, 22.98, 22.82, 22.21, 21.00, 18.20, 15.03, 14.25, -4.26, -4.49.
f1 (ppm)

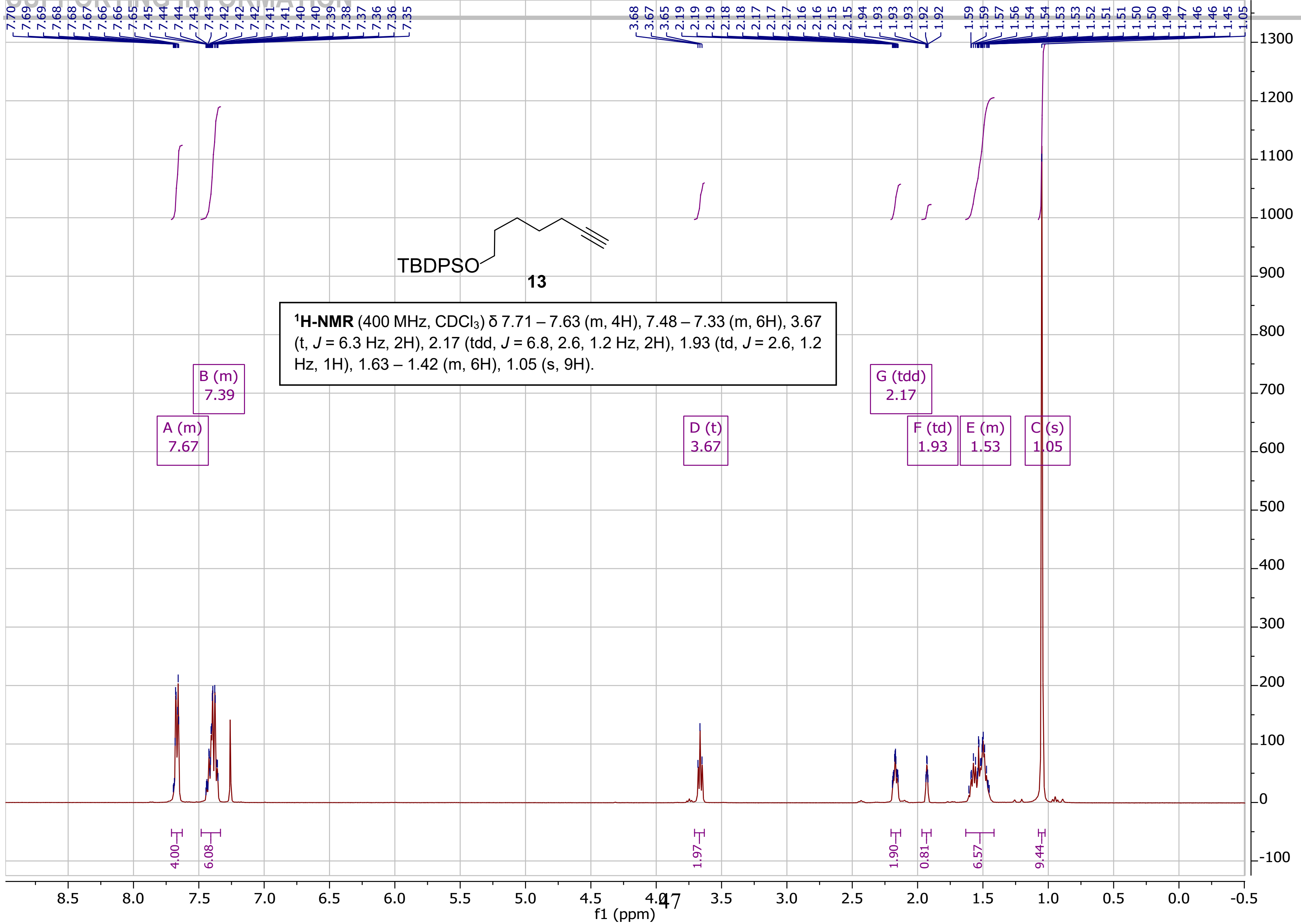
TBDPSO
13
**¹H-NMR** (400 MHz, CDCl3) δ 7.71 – 7.63 (m, 4H), 7.48 – 7.33 (m, 6H), 3.67 (t, *J* = 6.3 Hz, 2H), 2.17 (tdd, *J* = 6.8, 2.6, 1.2 Hz, 2H), 1.93 (td, *J* = 2.6, 1.2 Hz, 1H), 1.63 – 1.42 (m, 6H), 1.05 (s, 9H).
A (m) 7.67
B (m) 7.39
D (t) 3.67
G (tdd) 2.17
F (td) 1.93
E (m) 1.53
C (s) 1.05
4.00
6.08
1.97
1.90
0.81
6.57
9.44
f1 (ppm)

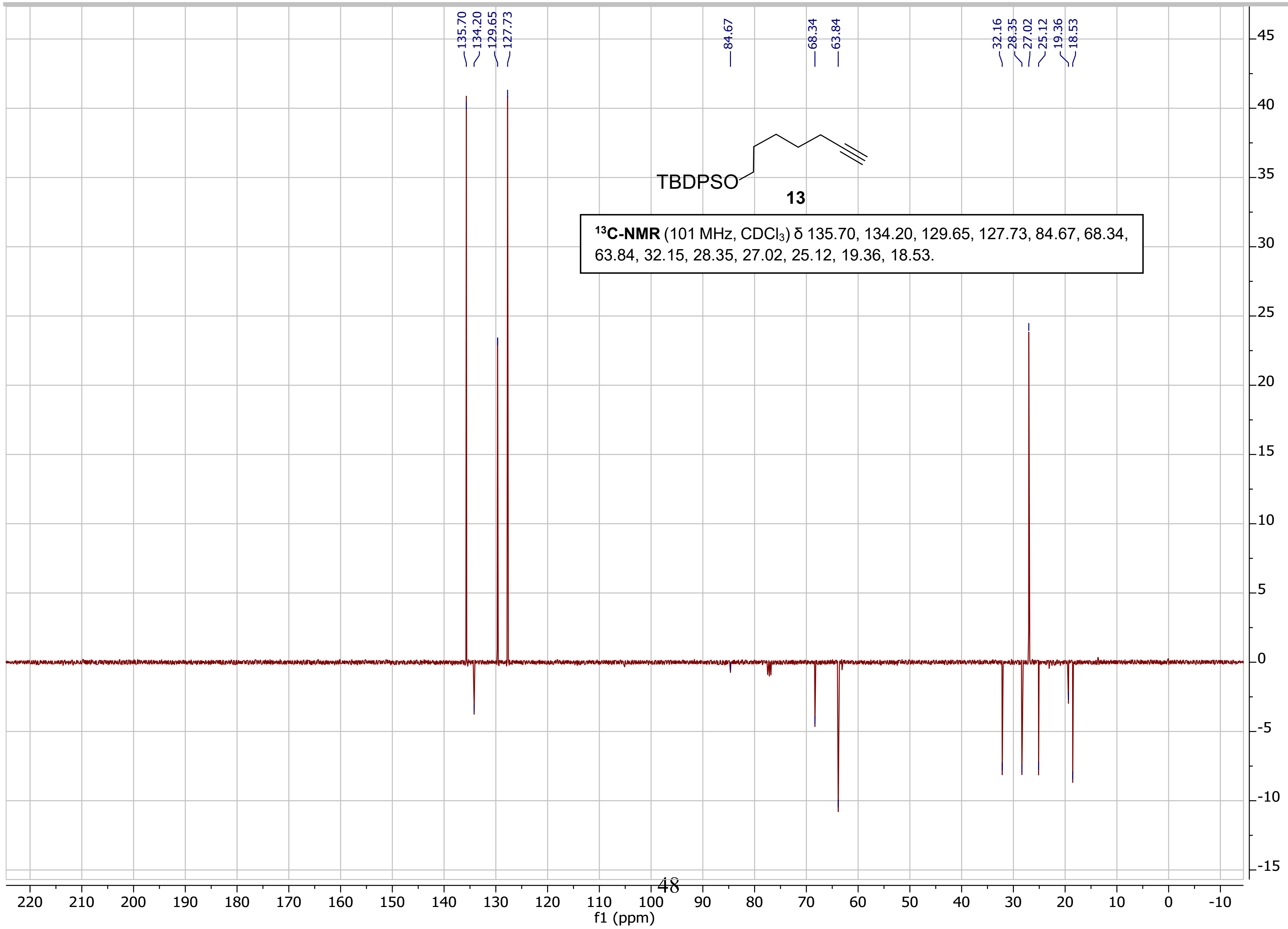


**13**

**$^{13}$C-NMR** (101 MHz, $CDCl_3$) δ 135.70, 134.20, 129.65, 127.73, 84.67, 68.34, 63.84, 32.15, 28.35, 27.02, 25.12, 19.36, 18.53.

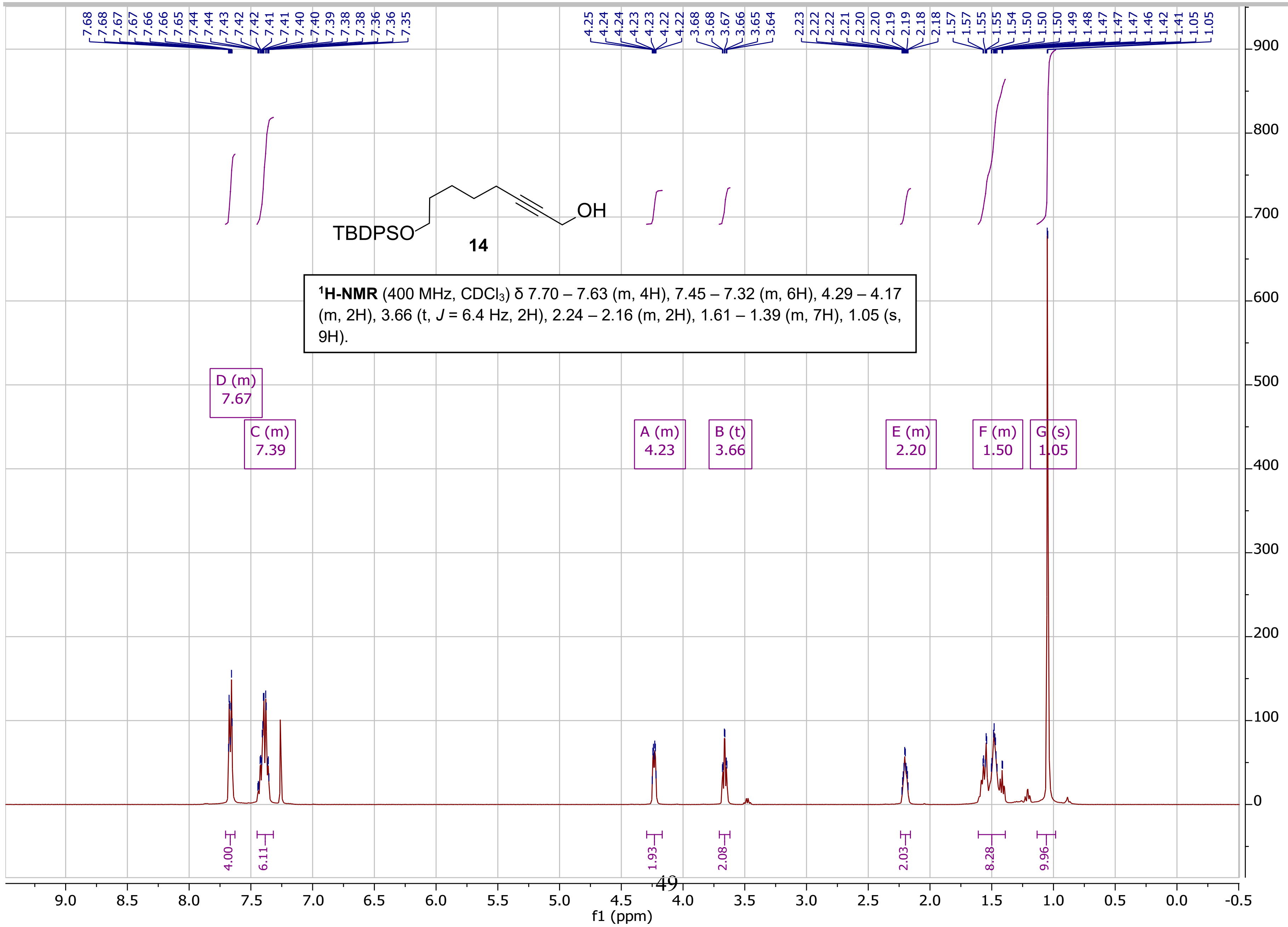
TBDPSO
OH
14
**1H-NMR** (400 MHz, $CDCl_3$) δ 7.70 – 7.63 (m, 4H), 7.45 – 7.32 (m, 6H), 4.29 – 4.17 (m, 2H), 3.66 (t, *J* = 6.4 Hz, 2H), 2.24 – 2.16 (m, 2H), 1.61 – 1.39 (m, 7H), 1.05 (s, 9H).
D (m) 7.67
C (m) 7.39
A (m) 4.23
B (t) 3.66
E (m) 2.20
F (m) 1.50
G (s) 1.05
4.00
6.11
1.93
2.08
2.03
8.28
9.96
f1 (ppm)

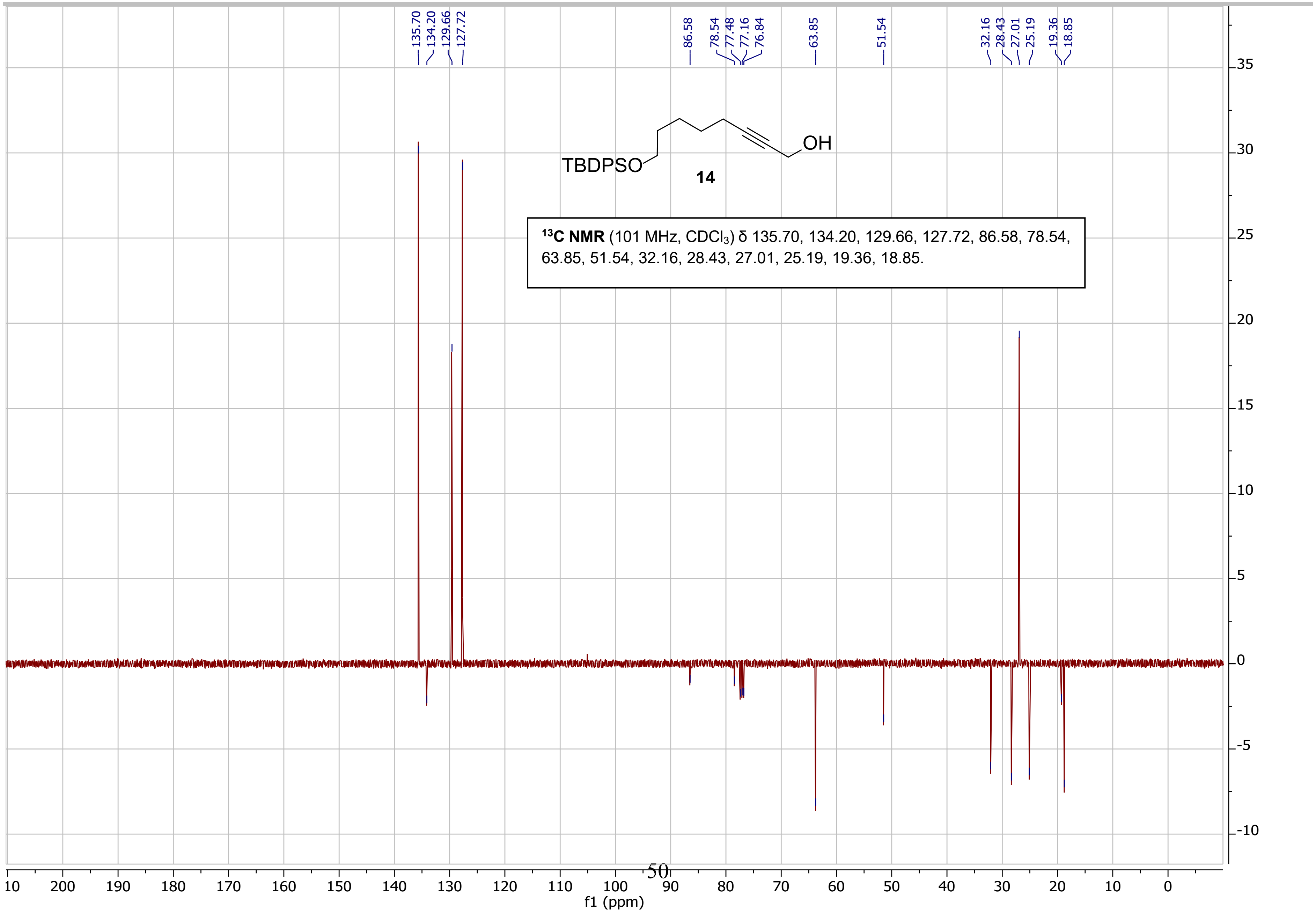


**$^{13}$C NMR** (101 MHz, $CDCl_3$) δ 135.70, 134.20, 129.66, 127.72, 86.58, 78.54, 63.85, 51.54, 32.16, 28.43, 27.01, 25.19, 19.36, 18.85.

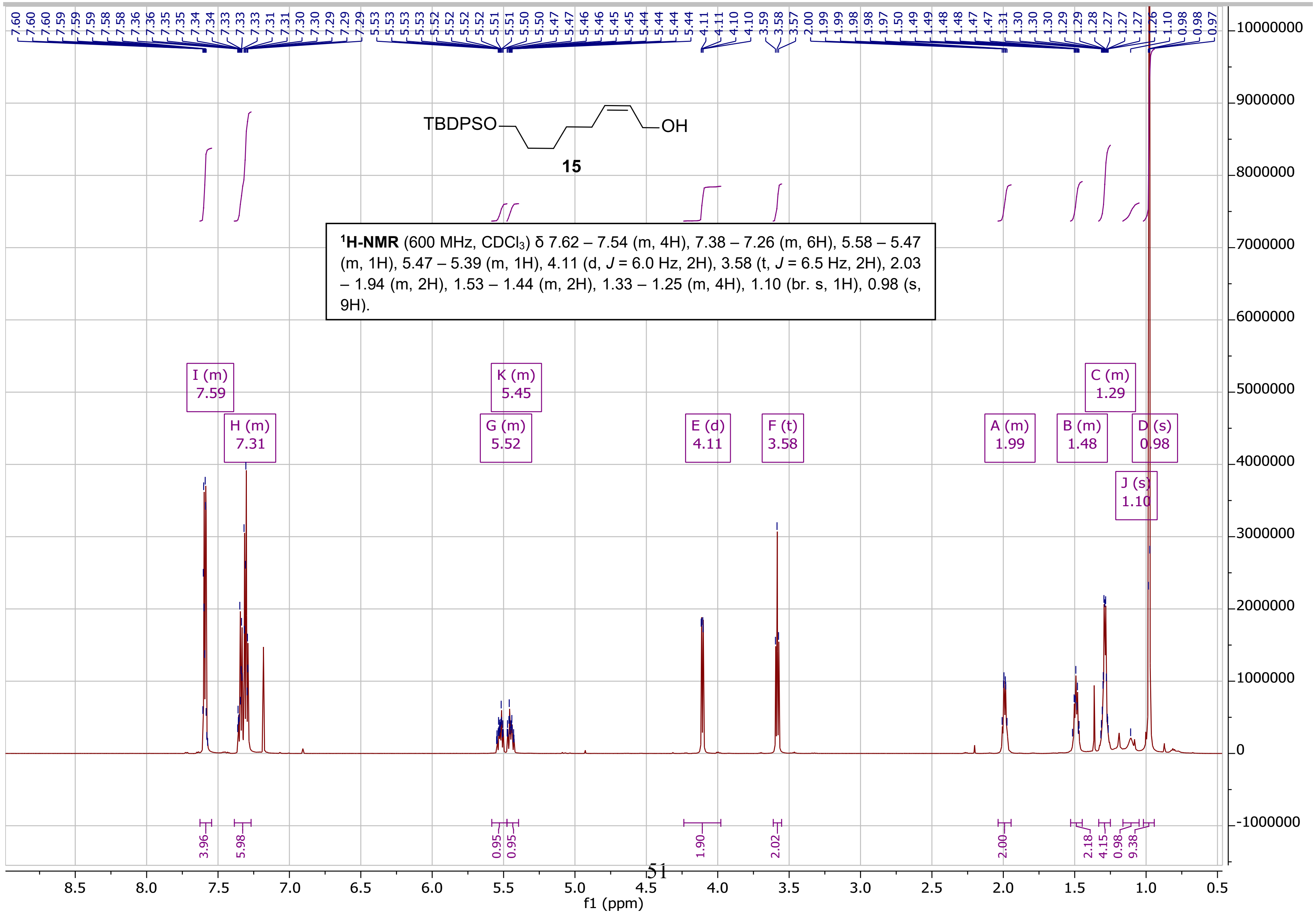
TBDPSO
OH
15
$^{1}$H-NMR (600 MHz, CDCl3) δ 7.62 – 7.54 (m, 4H), 7.38 – 7.26 (m, 6H), 5.58 – 5.47 (m, 1H), 5.47 – 5.39 (m, 1H), 4.11 (d, J = 6.0 Hz, 2H), 3.58 (t, J = 6.5 Hz, 2H), 2.03 – 1.94 (m, 2H), 1.53 – 1.44 (m, 2H), 1.33 – 1.25 (m, 4H), 1.10 (br. s, 1H), 0.98 (s, 9H).
I (m) 7.59
H (m) 7.31
K (m) 5.45
G (m) 5.52
E (d) 4.11
F (t) 3.58
A (m) 1.99
B (m) 1.48
C (m) 1.29
D (s) 0.98
J (s) 1.10
3.96
5.98
0.95
0.95
1.90
2.02
2.00
2.18
4.15
0.98
9.38
f1 (ppm)

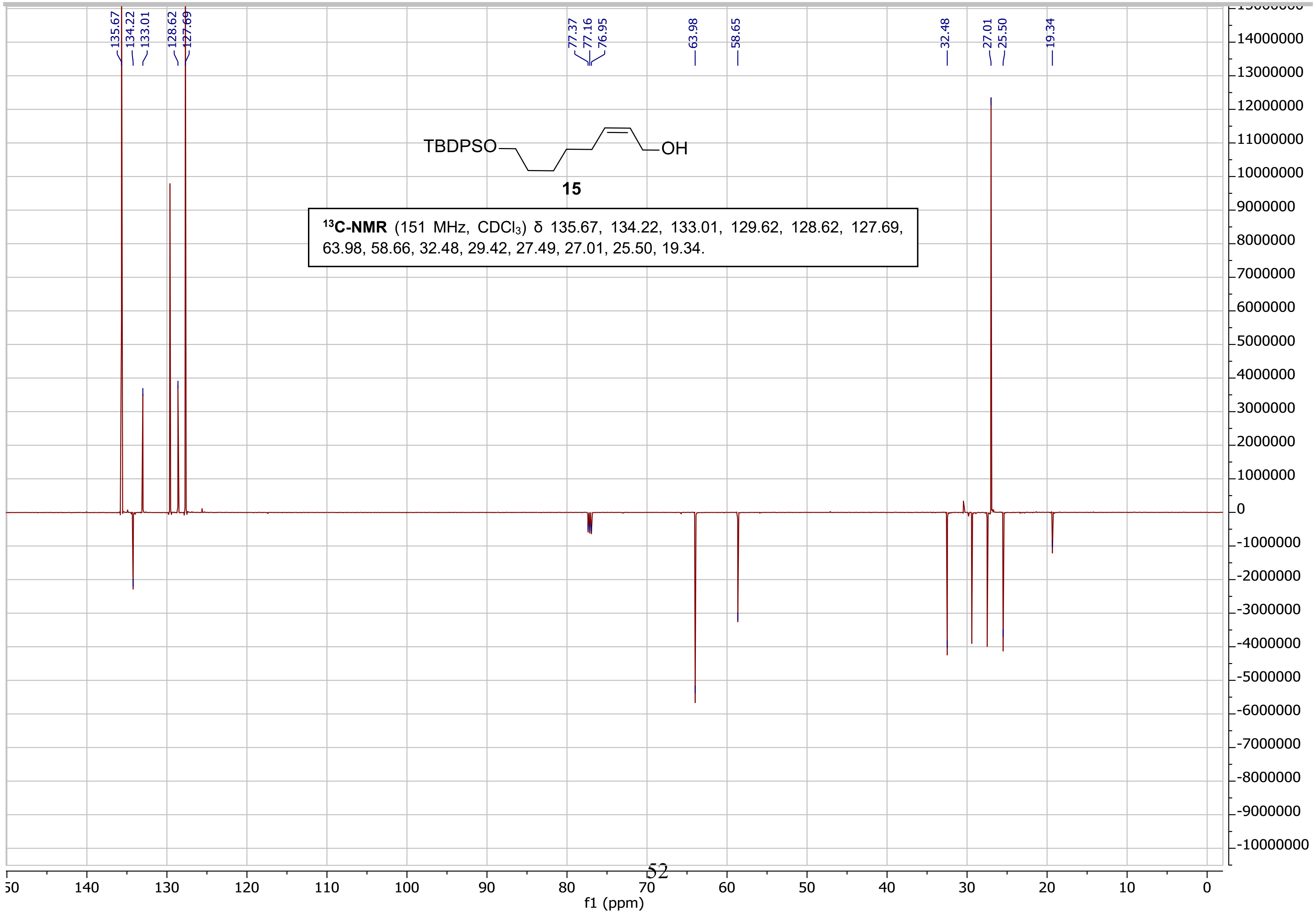


**$^{13}$C-NMR** (151 MHz, $CDCl_3$) δ 135.67, 134.22, 133.01, 129.62, 128.62, 127.69, 63.98, 58.66, 32.48, 29.42, 27.49, 27.01, 25.50, 19.34.

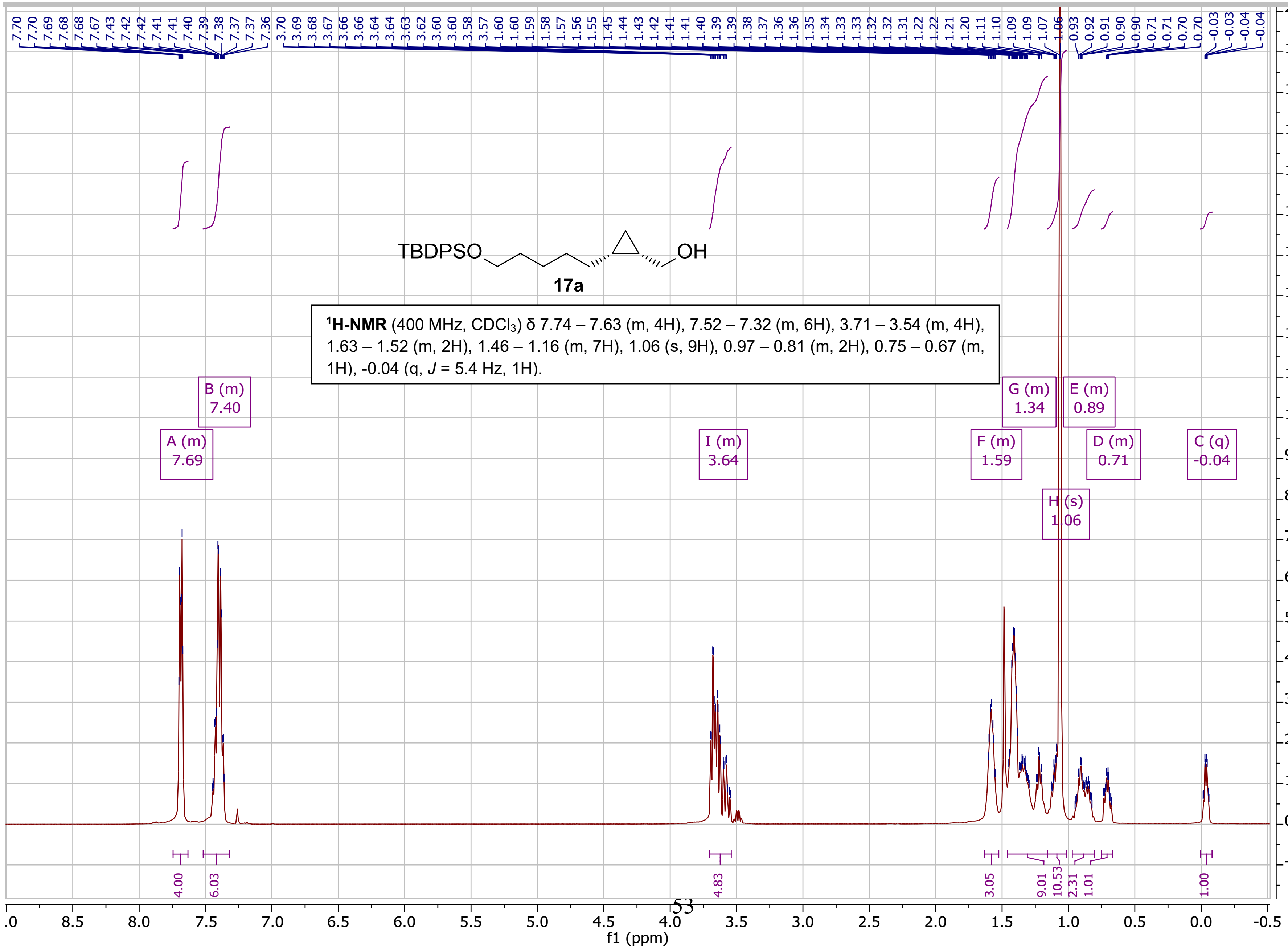
TBDPSO
OH
17a
¹H-NMR (400 MHz, CDCl₃) δ 7.74 – 7.63 (m, 4H), 7.52 – 7.32 (m, 6H), 3.71 – 3.54 (m, 4H), 1.63 – 1.52 (m, 2H), 1.46 – 1.16 (m, 7H), 1.06 (s, 9H), 0.97 – 0.81 (m, 2H), 0.75 – 0.67 (m, 1H), -0.04 (q, J = 5.4 Hz, 1H).
A (m) 7.69
B (m) 7.40
I (m) 3.64
F (m) 1.59
G (m) 1.34
H (s) 1.06
E (m) 0.89
D (m) 0.71
C (q) -0.04
f1 (ppm)

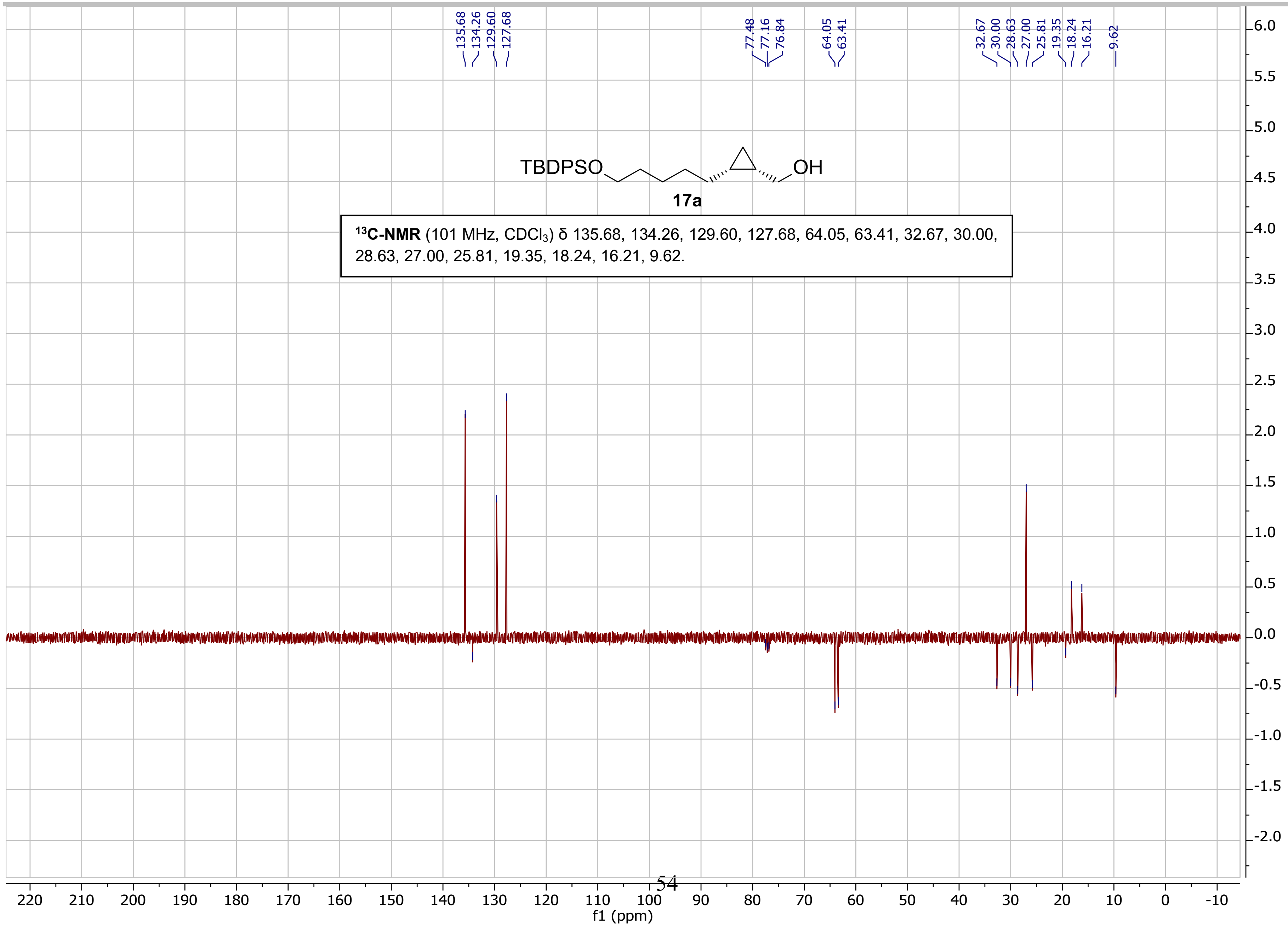


**17a**

[13]**C-NMR** (101 MHz, $CDCl_3$) δ 135.68, 134.26, 129.60, 127.68, 64.05, 63.41, 32.67, 30.00, 28.63, 27.00, 25.81, 19.35, 18.24, 16.21, 9.62.

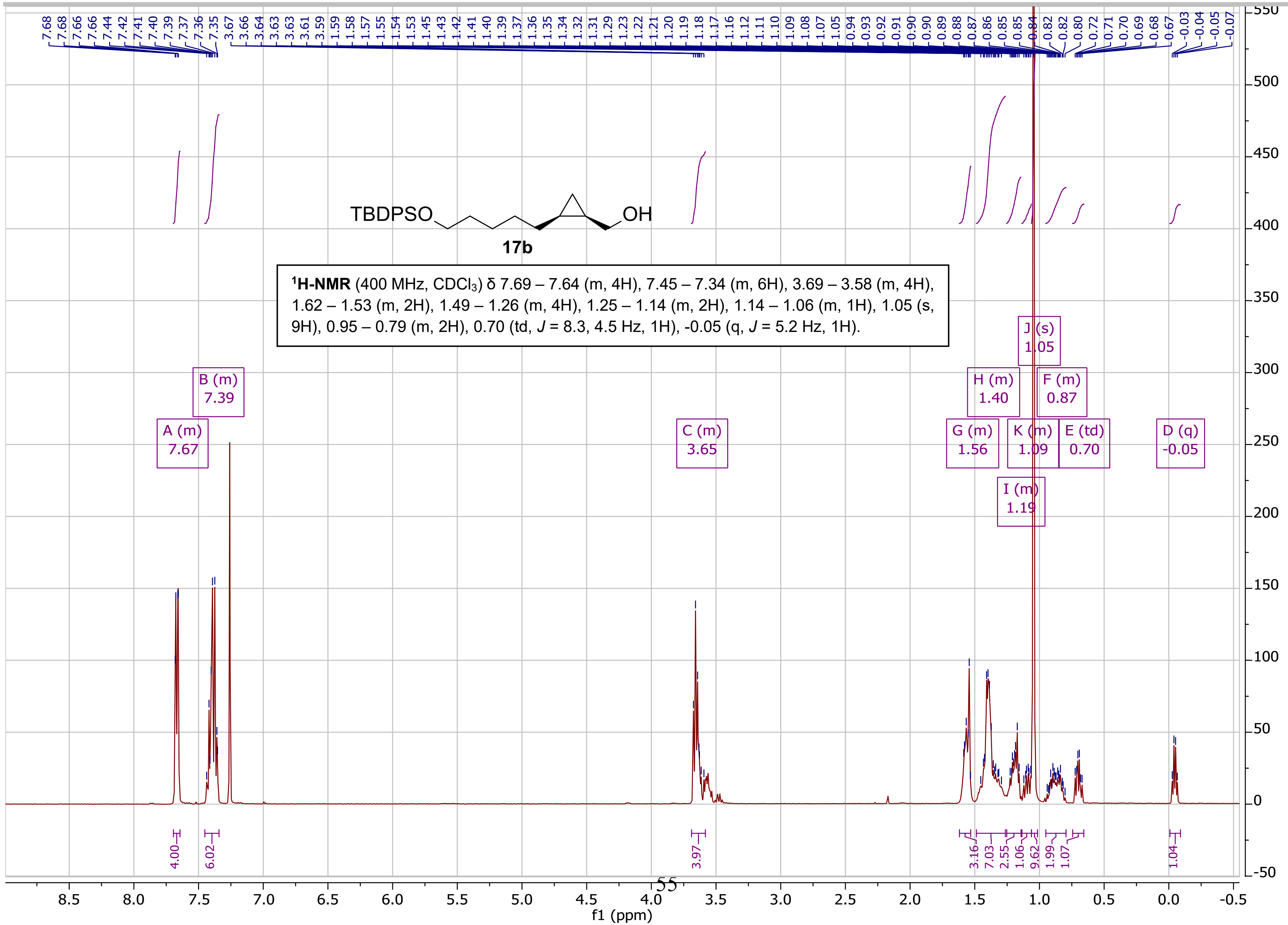


**$^1$H-NMR** (400 MHz, $CDCl_3$) δ 7.69 – 7.64 (m, 4H), 7.45 – 7.34 (m, 6H), 3.69 – 3.58 (m, 4H), 1.62 – 1.53 (m, 2H), 1.49 – 1.26 (m, 4H), 1.25 – 1.14 (m, 2H), 1.14 – 1.06 (m, 1H), 1.05 (s, 9H), 0.95 – 0.79 (m, 2H), 0.70 (td, *J* = 8.3, 4.5 Hz, 1H), -0.05 (q, *J* = 5.2 Hz, 1H).

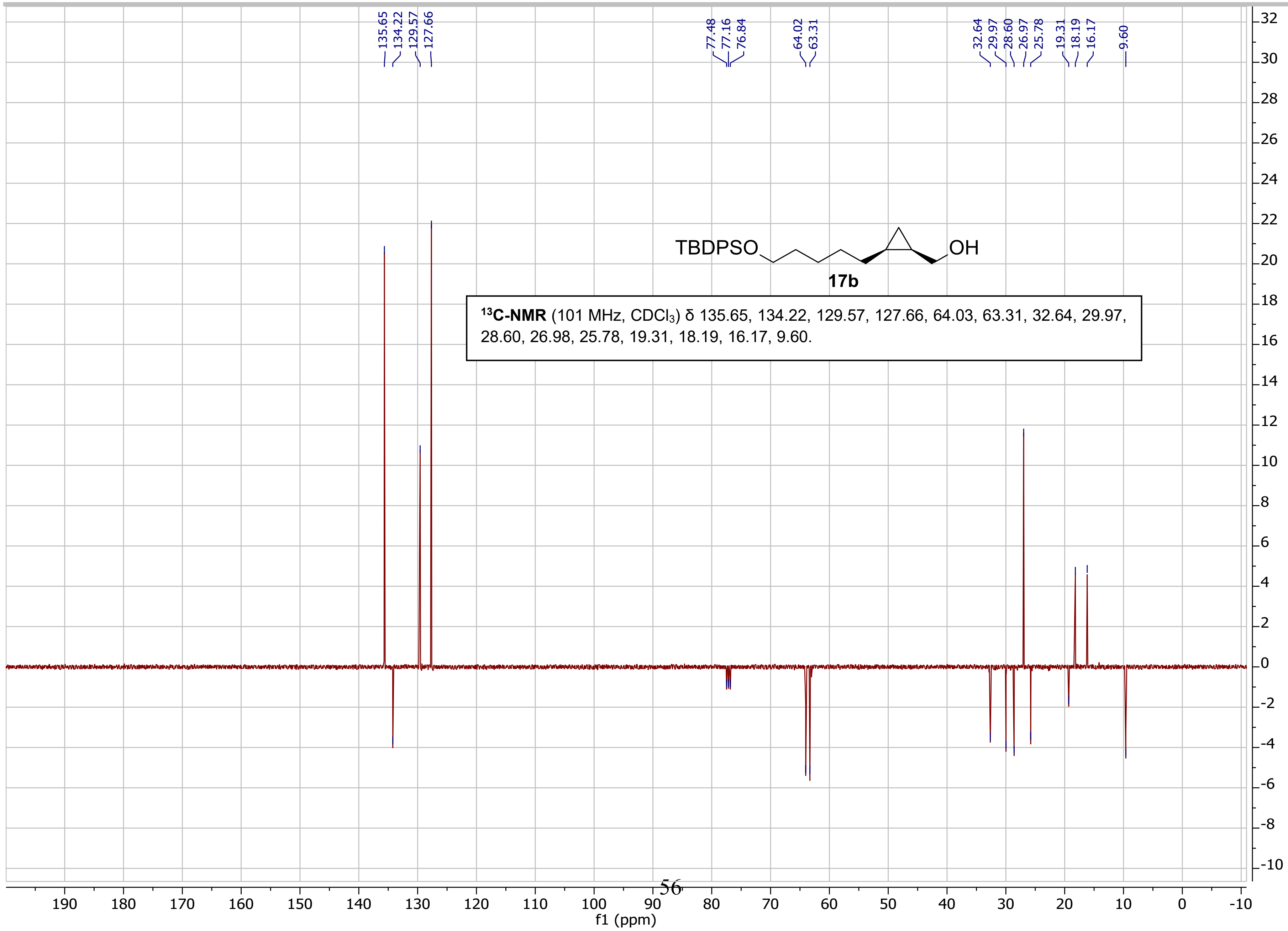


**$^{13}$C-NMR** (101 MHz, $CDCl_3$) δ 135.65, 134.22, 129.57, 127.66, 64.03, 63.31, 32.64, 29.97, 28.60, 26.98, 25.78, 19.31, 18.19, 16.17, 9.60.

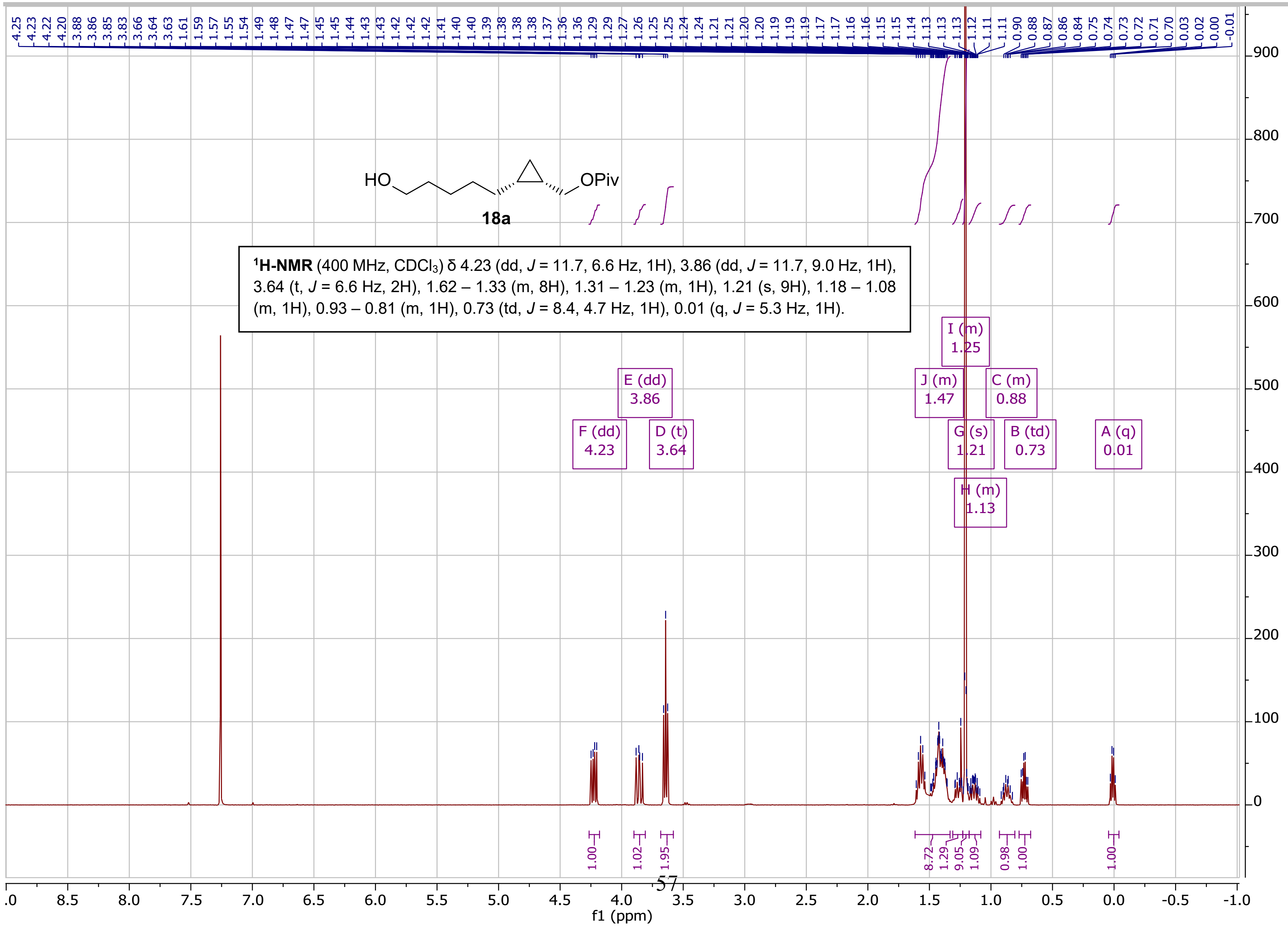
HO
OPiv
18a
$^{1}$H-NMR (400 MHz, $CDCl_3$) δ 4.23 (dd, J = 11.7, 6.6 Hz, 1H), 3.86 (dd, J = 11.7, 9.0 Hz, 1H), 3.64 (t, J = 6.6 Hz, 2H), 1.62 – 1.33 (m, 8H), 1.31 – 1.23 (m, 1H), 1.21 (s, 9H), 1.18 – 1.08 (m, 1H), 0.93 – 0.81 (m, 1H), 0.73 (td, J = 8.4, 4.7 Hz, 1H), 0.01 (q, J = 5.3 Hz, 1H).
F (dd) 4.23
E (dd) 3.86
D (t) 3.64
J (m) 1.47
I (m) 1.25
G (s) 1.21
H (m) 1.13
C (m) 0.88
B (td) 0.73
A (q) 0.01
1.00
1.02
1.95
8.72
1.29
9.05
1.09
0.98
1.00
1.00
f1 (ppm)

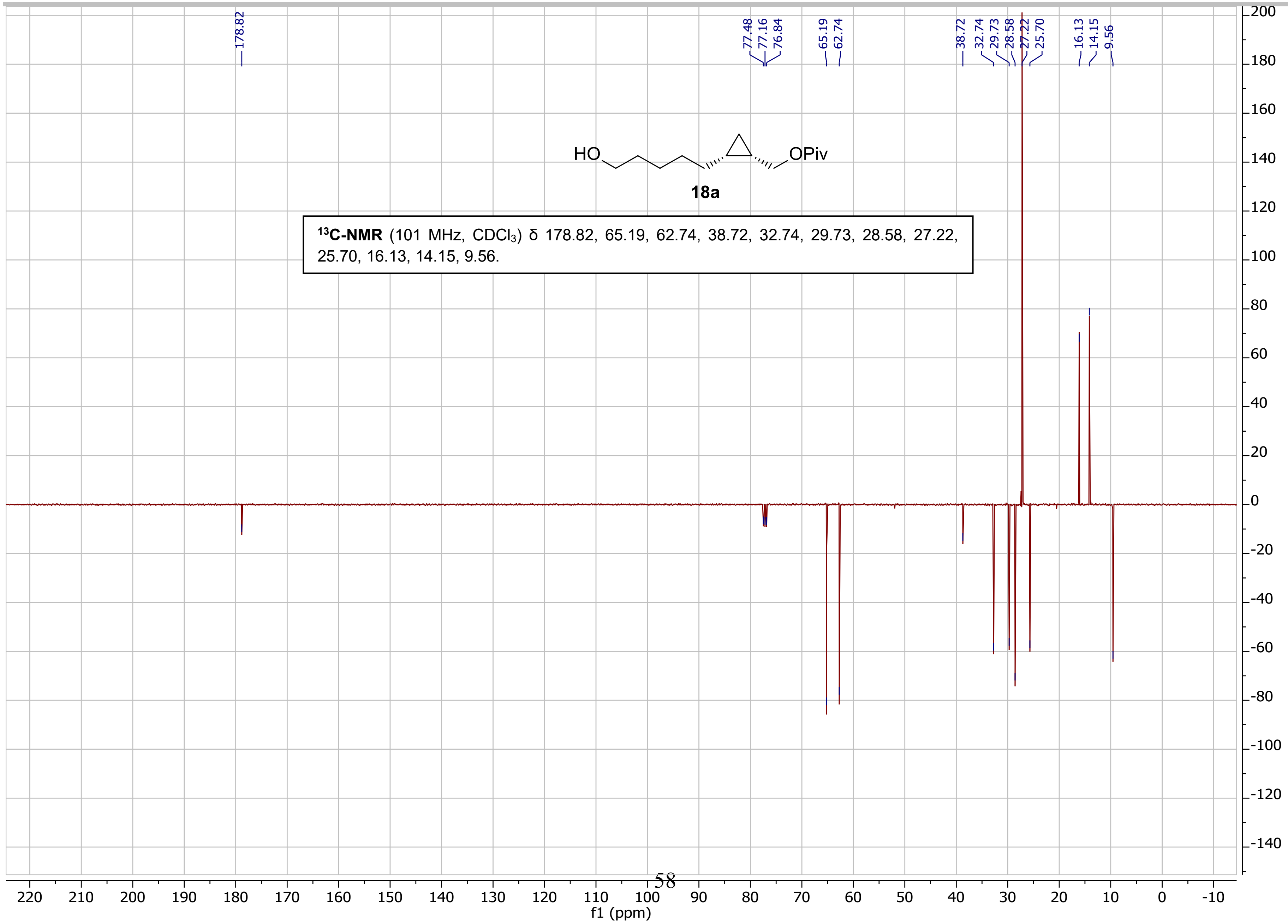


**$^{13}$C-NMR** (101 MHz, $CDCl_3$) δ 178.82, 65.19, 62.74, 38.72, 32.74, 29.73, 28.58, 27.22, 25.70, 16.13, 14.15, 9.56.

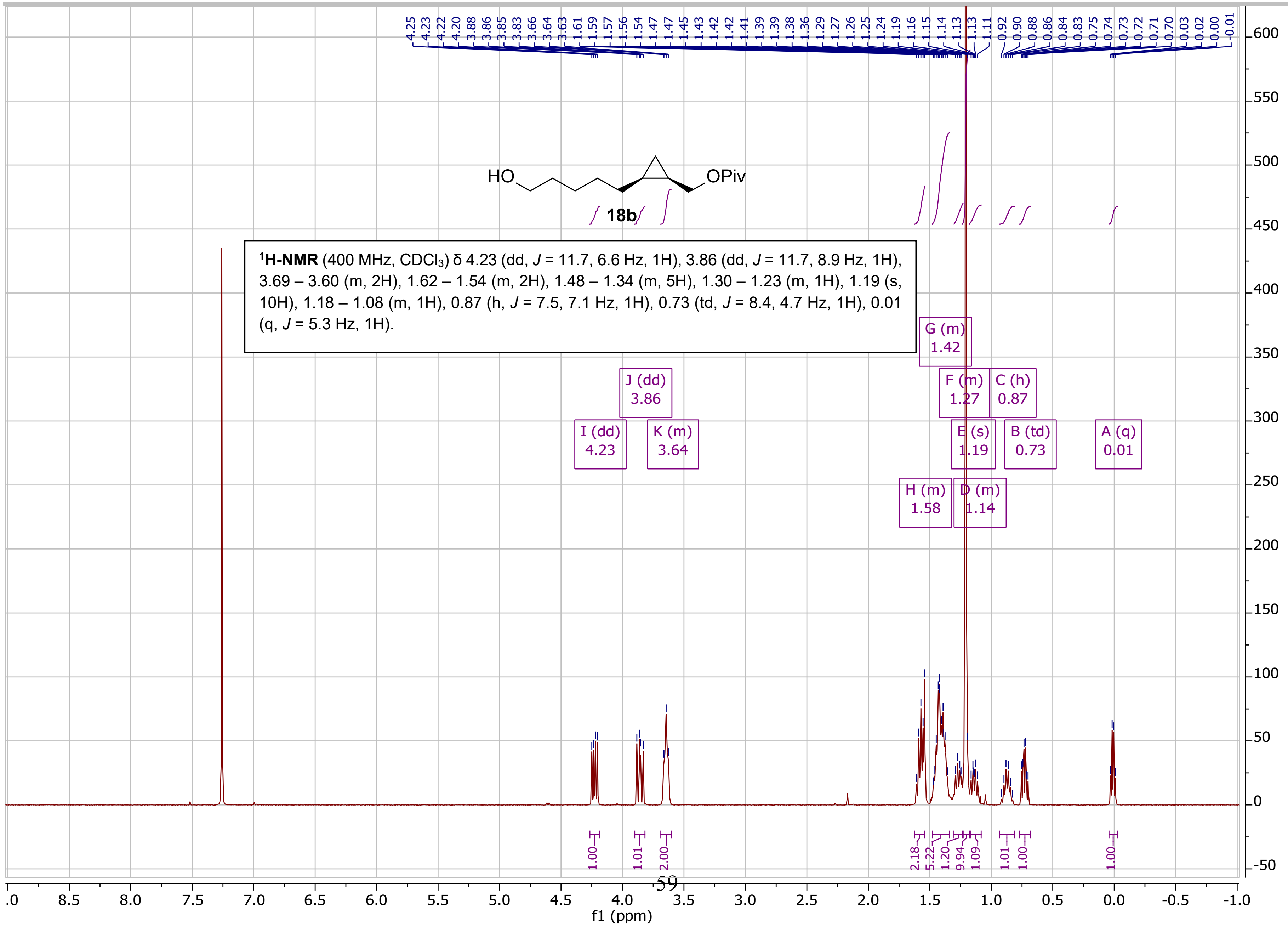


[1]**H-NMR** (400 MHz, $CDCl_3$) δ 4.23 (dd, *J* = 11.7, 6.6 Hz, 1H), 3.86 (dd, *J* = 11.7, 8.9 Hz, 1H), 3.69 – 3.60 (m, 2H), 1.62 – 1.54 (m, 2H), 1.48 – 1.34 (m, 5H), 1.30 – 1.23 (m, 1H), 1.19 (s, 10H), 1.18 – 1.08 (m, 1H), 0.87 (h, *J* = 7.5, 7.1 Hz, 1H), 0.73 (td, *J* = 8.4, 4.7 Hz, 1H), 0.01 (q, *J* = 5.3 Hz, 1H).

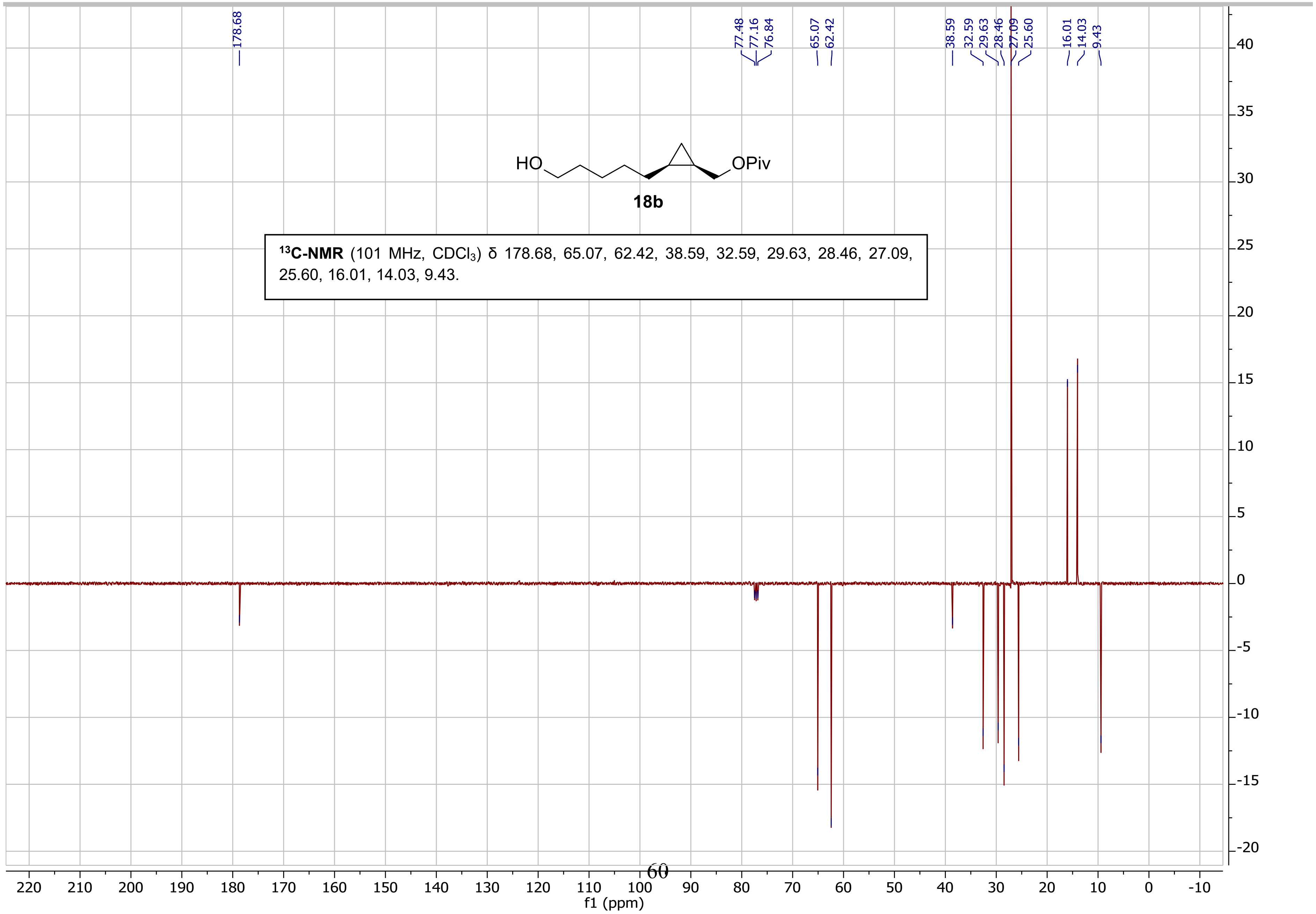


**$^{13}$C-NMR** (101 MHz, $CDCl_3$) δ 178.68, 65.07, 62.42, 38.59, 32.59, 29.63, 28.46, 27.09, 25.60, 16.01, 14.03, 9.43.

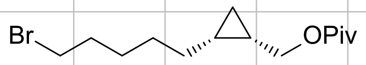

***S,R*-fragment B**

**¹H-NMR** (400 MHz, $CDCl_3$) δ 4.23 (dd, *J* = 11.7, 6.5 Hz, 1H), 3.85 (dd, *J* = 11.7, 9.0 Hz, 1H), 3.41 (t, *J* = 6.8 Hz, 2H), 1.86 (p, *J* = 6.9 Hz, 2H), 1.50 – 1.35 (m, 5H), 1.27 (t, *J* = 7.4 Hz, 1H), 1.21 (s, 9H), 1.18 – 1.09 (m, 1H), 0.93 – 0.79 (m, 1H), 0.74 (td, *J* = 8.4, 4.7 Hz, 1H), 0.02 (q, *J* = 5.3 Hz, 1H).

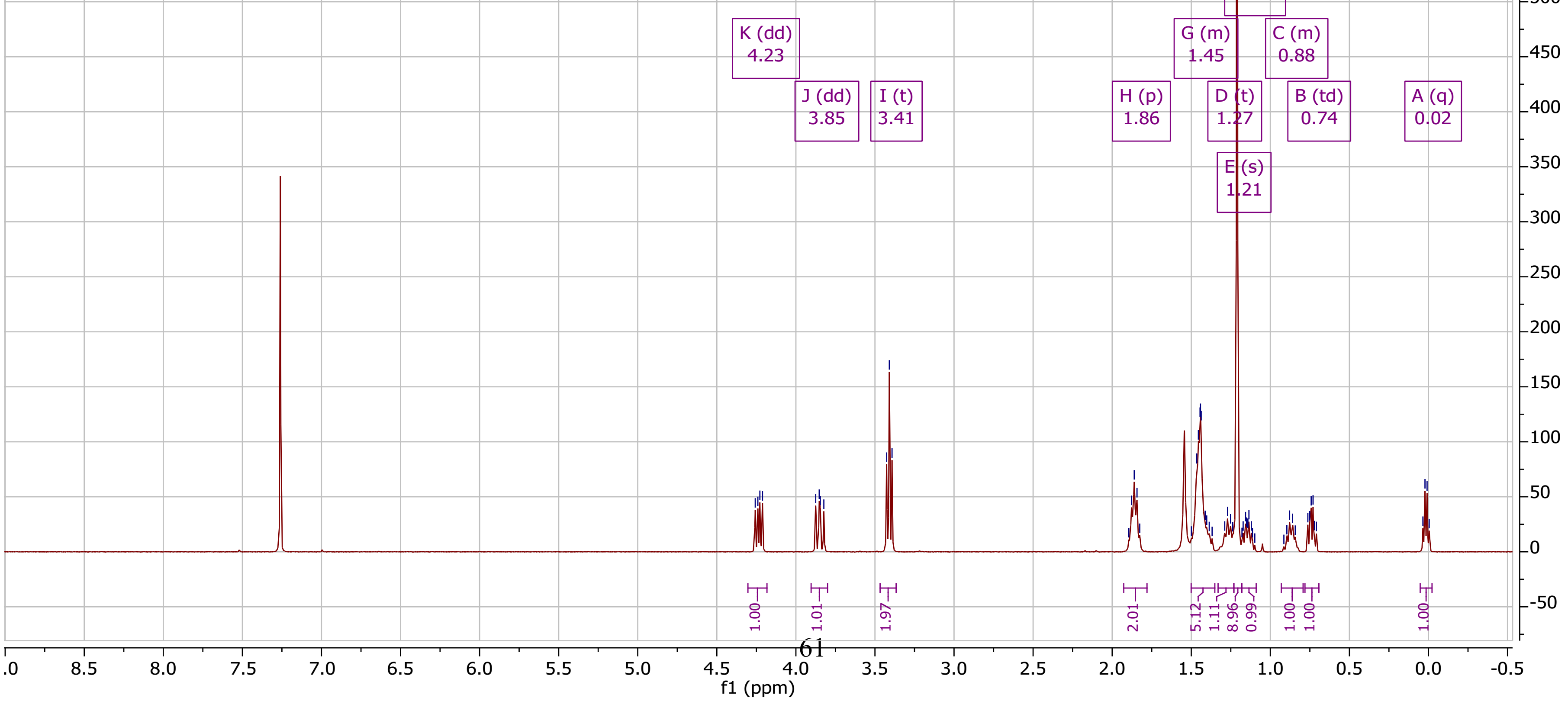

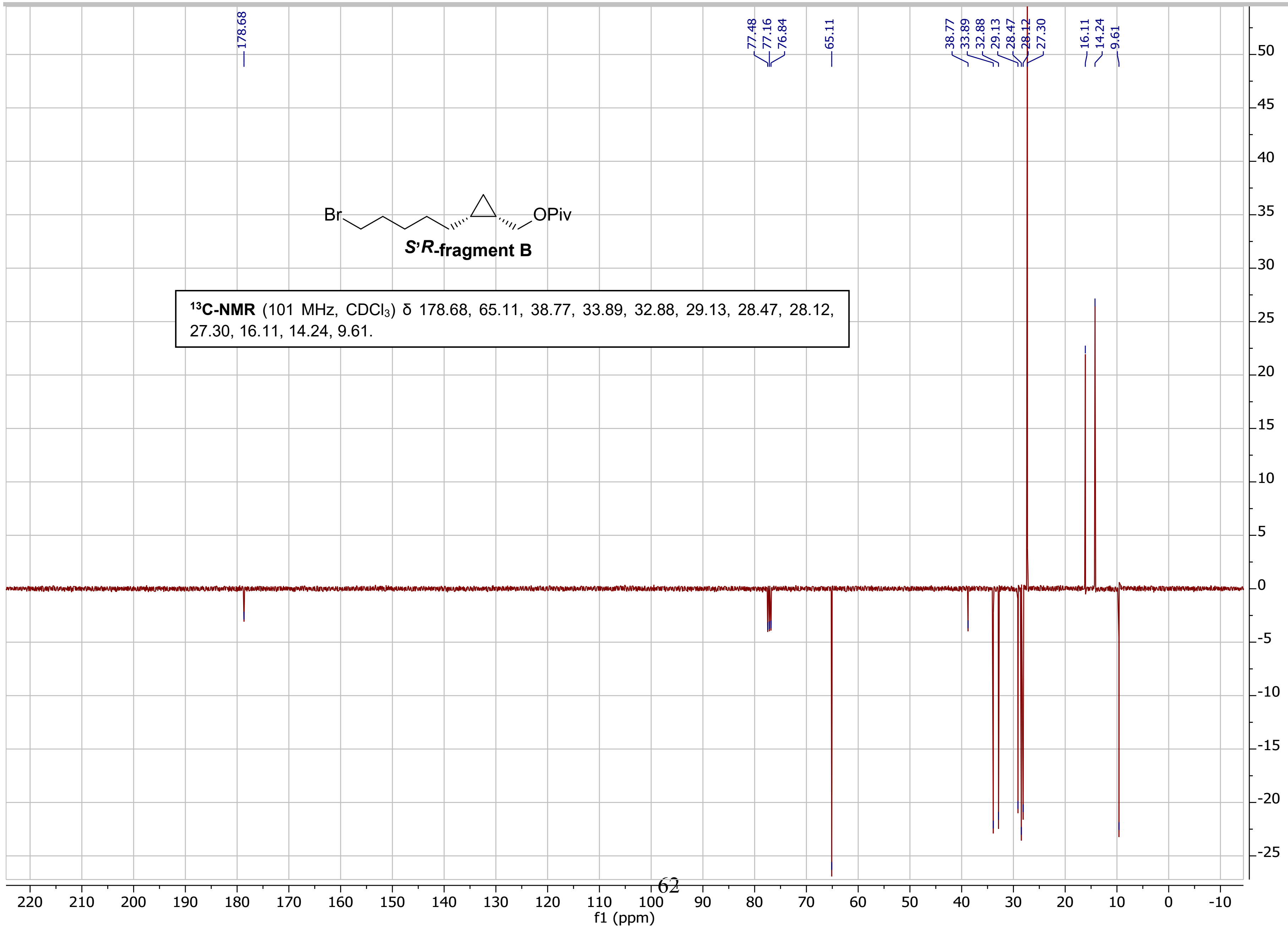


**$^{13}$C-NMR** (101 MHz, $CDCl_3$) δ 178.68, 65.11, 38.77, 33.89, 32.88, 29.13, 28.47, 28.12, 27.30, 16.11, 14.24, 9.61.

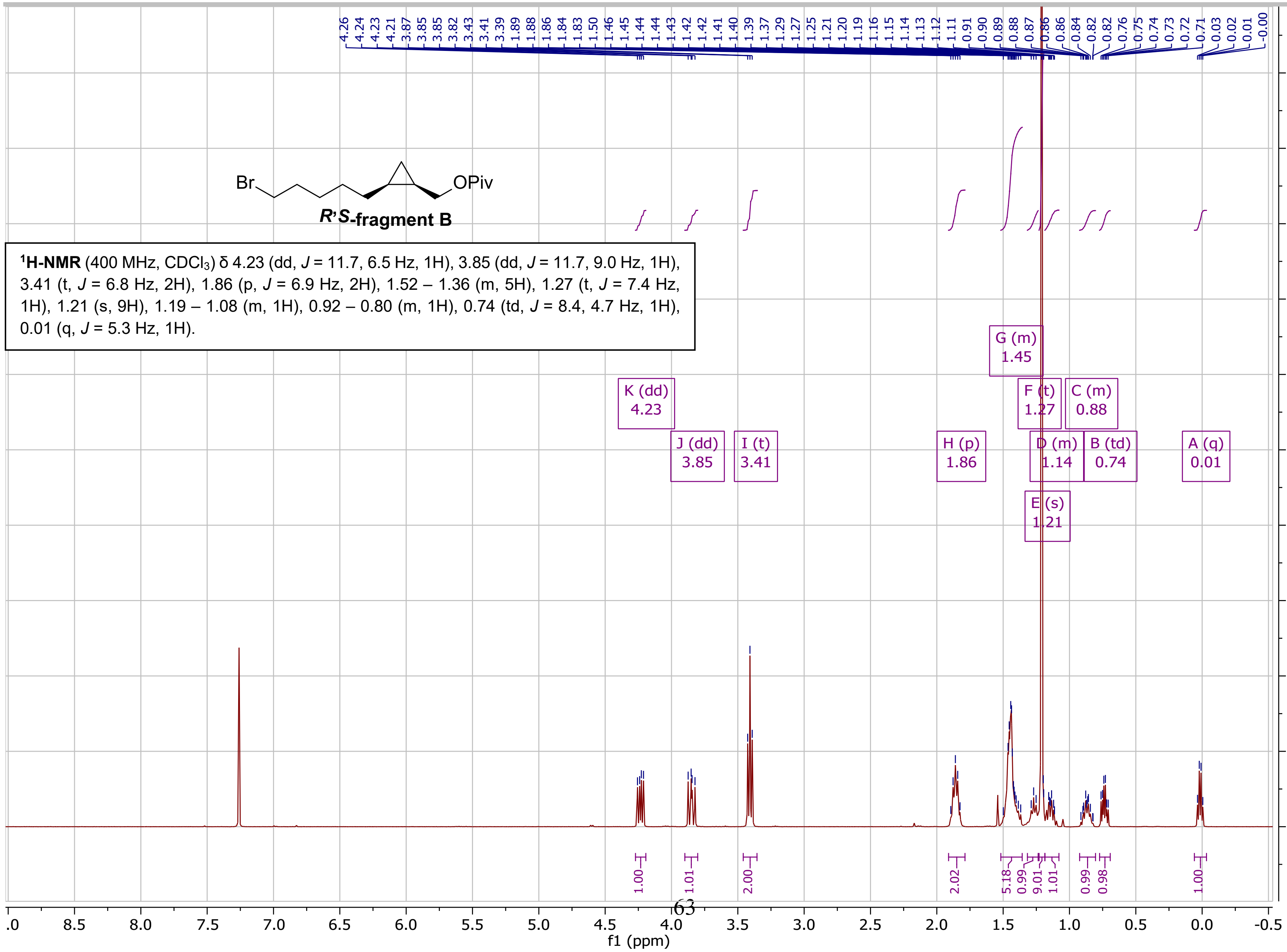


**$^1$H-NMR** (400 MHz, $CDCl_3$) δ 4.23 (dd, *J* = 11.7, 6.5 Hz, 1H), 3.85 (dd, *J* = 11.7, 9.0 Hz, 1H), 3.41 (t, *J* = 6.8 Hz, 2H), 1.86 (p, *J* = 6.9 Hz, 2H), 1.52 – 1.36 (m, 5H), 1.27 (t, *J* = 7.4 Hz, 1H), 1.21 (s, 9H), 1.19 – 1.08 (m, 1H), 0.92 – 0.80 (m, 1H), 0.74 (td, *J* = 8.4, 4.7 Hz, 1H), 0.01 (q, *J* = 5.3 Hz, 1H).

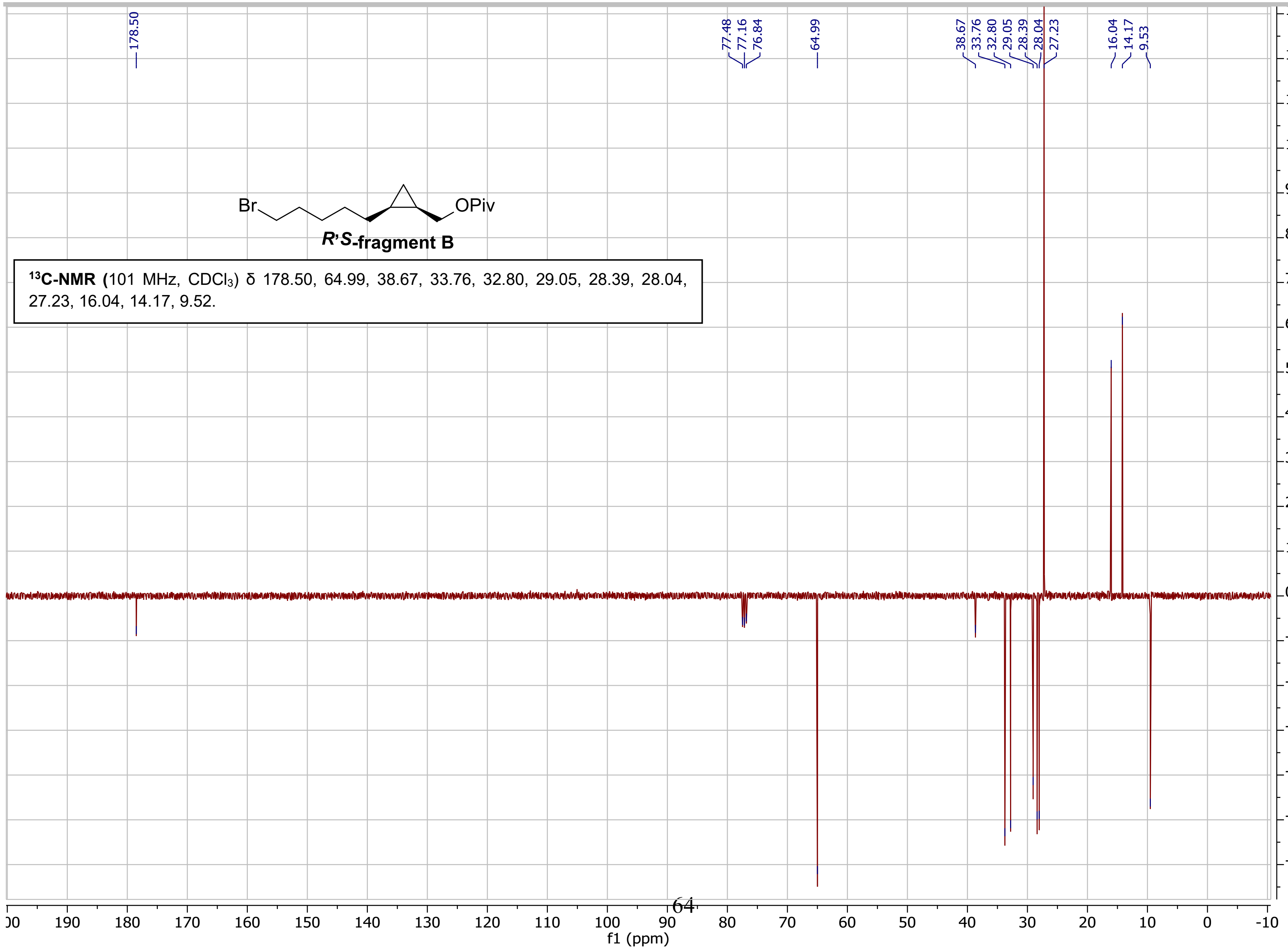


**$^{13}$C-NMR** (101 MHz, $CDCl_3$) δ 178.50, 64.99, 38.67, 33.76, 32.80, 29.05, 28.39, 28.04, 27.23, 16.04, 14.17, 9.52.

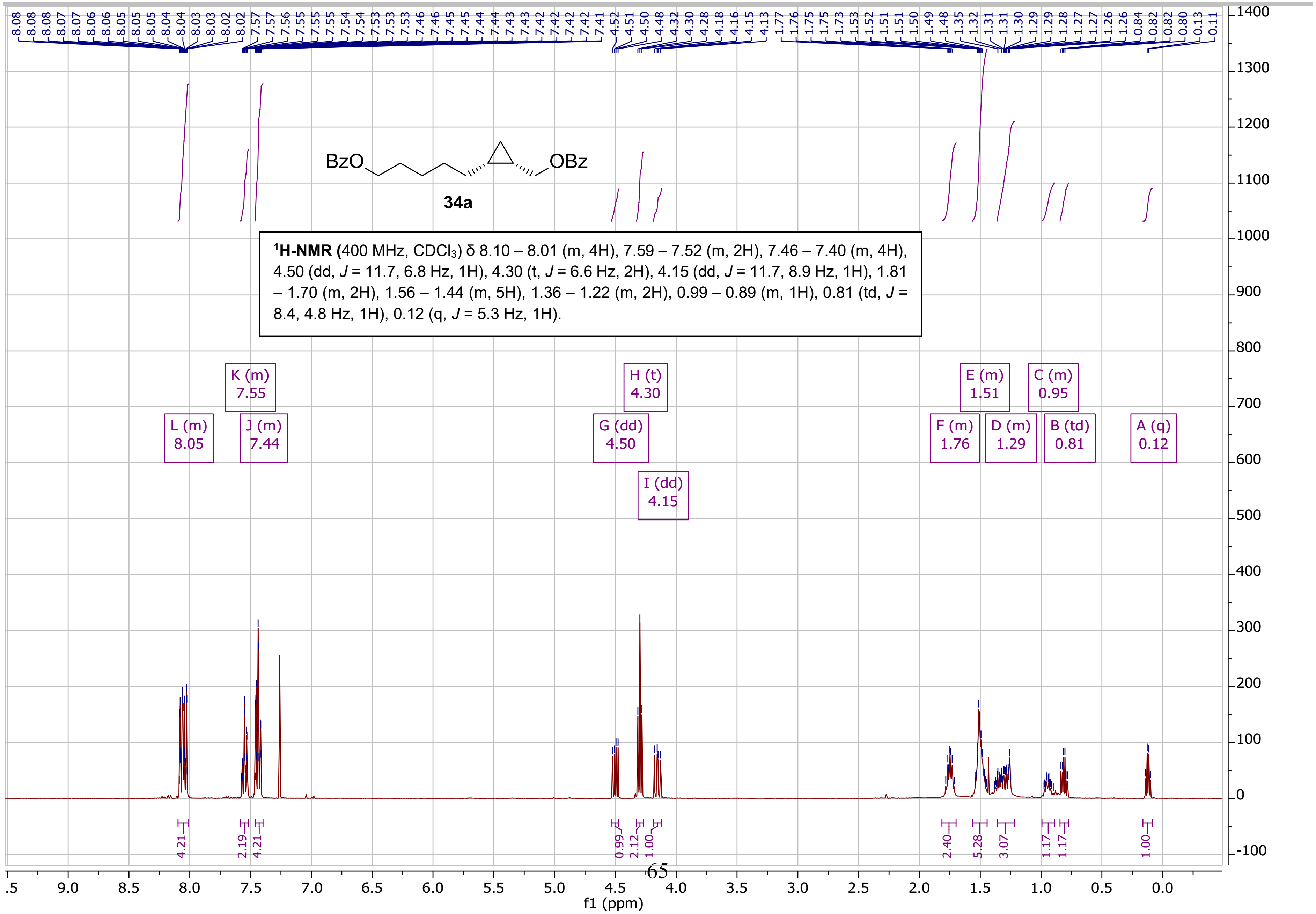


[1]**H-NMR (**400 MHz, $CDCl_3$) δ 8.10 – 8.01 (m, 4H), 7.59 – 7.52 (m, 2H), 7.46 – 7.40 (m, 4H), 4.50 (dd, *J* = 11.7, 6.8 Hz, 1H), 4.30 (t, *J* = 6.6 Hz, 2H), 4.15 (dd, *J* = 11.7, 8.9 Hz, 1H), 1.81 – 1.70 (m, 2H), 1.56 – 1.44 (m, 5H), 1.36 – 1.22 (m, 2H), 0.99 – 0.89 (m, 1H), 0.81 (td, *J* = 8.4, 4.8 Hz, 1H), 0.12 (q, *J* = 5.3 Hz, 1H).

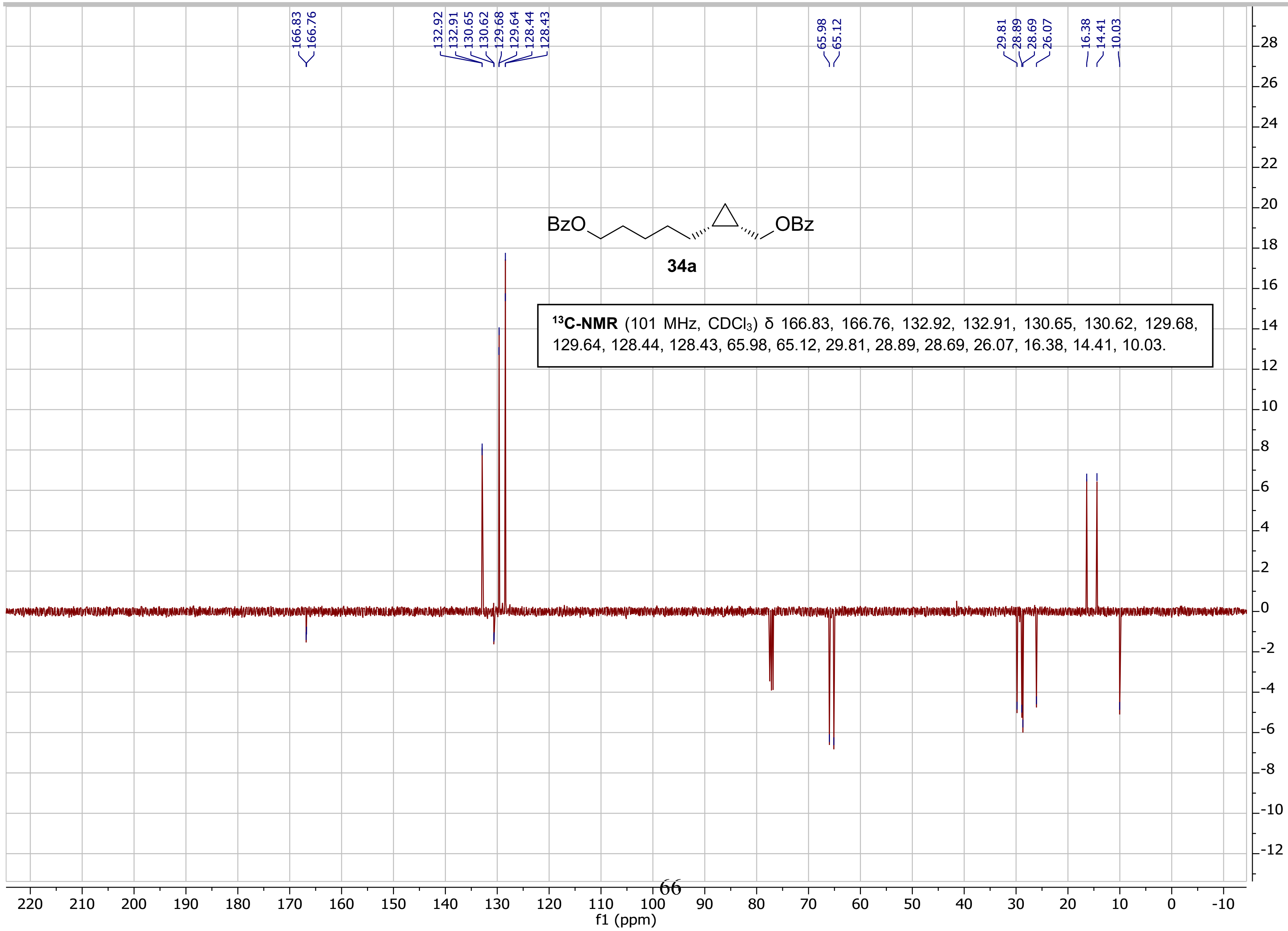


**34a**

**$^{13}$C-NMR** (101 MHz, $CDCl_3$) δ 166.83, 166.76, 132.92, 132.91, 130.65, 130.62, 129.68, 129.64, 128.44, 128.43, 65.98, 65.12, 29.81, 28.89, 28.69, 26.07, 16.38, 14.41, 10.03.

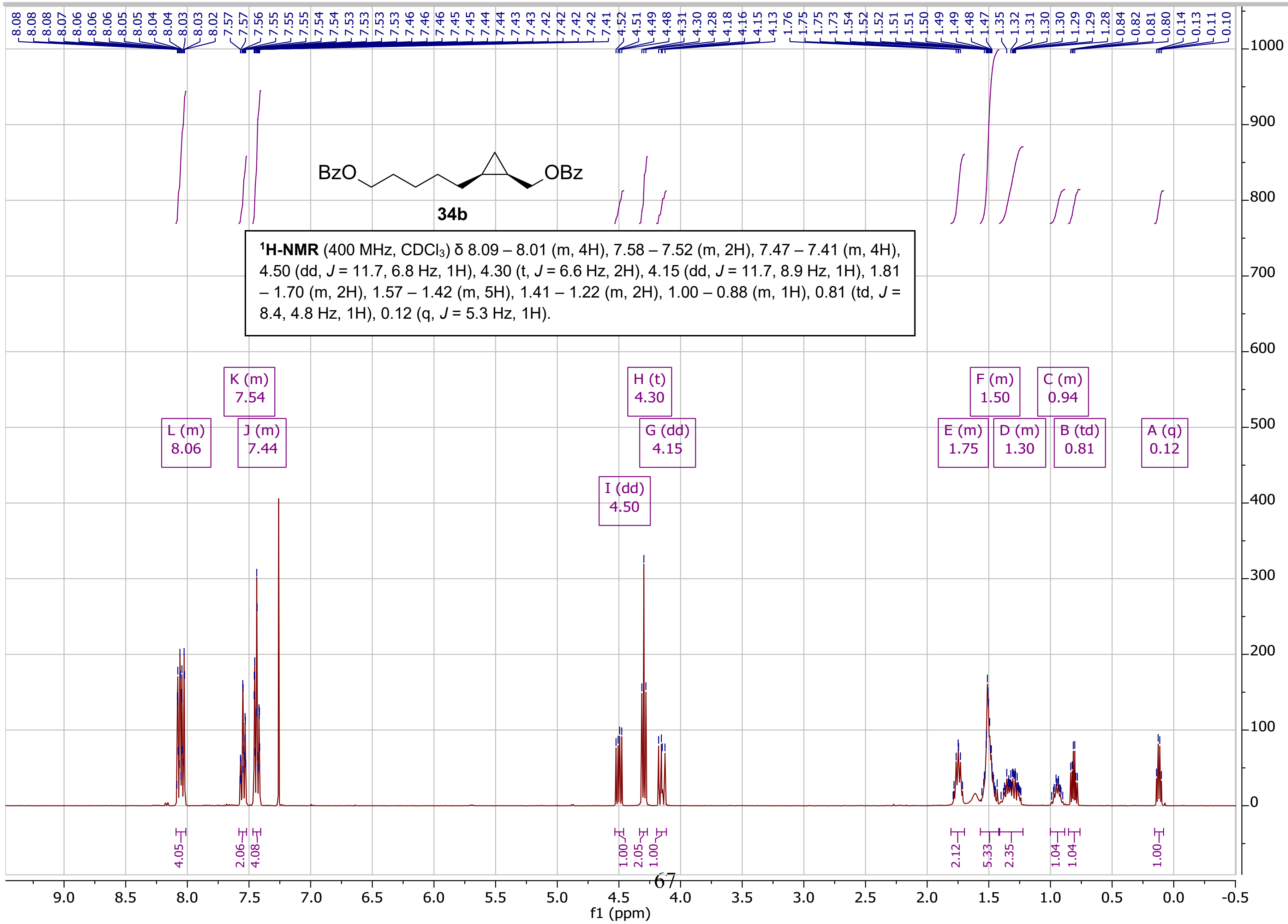

**34b**

[1]**H-NMR** (400 MHz, $CDCl_3$) δ 8.09 – 8.01 (m, 4H), 7.58 – 7.52 (m, 2H), 7.47 – 7.41 (m, 4H), 4.50 (dd, *J* = 11.7, 6.8 Hz, 1H), 4.30 (t, *J* = 6.6 Hz, 2H), 4.15 (dd, *J* = 11.7, 8.9 Hz, 1H), 1.81 – 1.70 (m, 2H), 1.57 – 1.42 (m, 5H), 1.41 – 1.22 (m, 2H), 1.00 – 0.88 (m, 1H), 0.81 (td, *J* = 8.4, 4.8 Hz, 1H), 0.12 (q, *J* = 5.3 Hz, 1H).

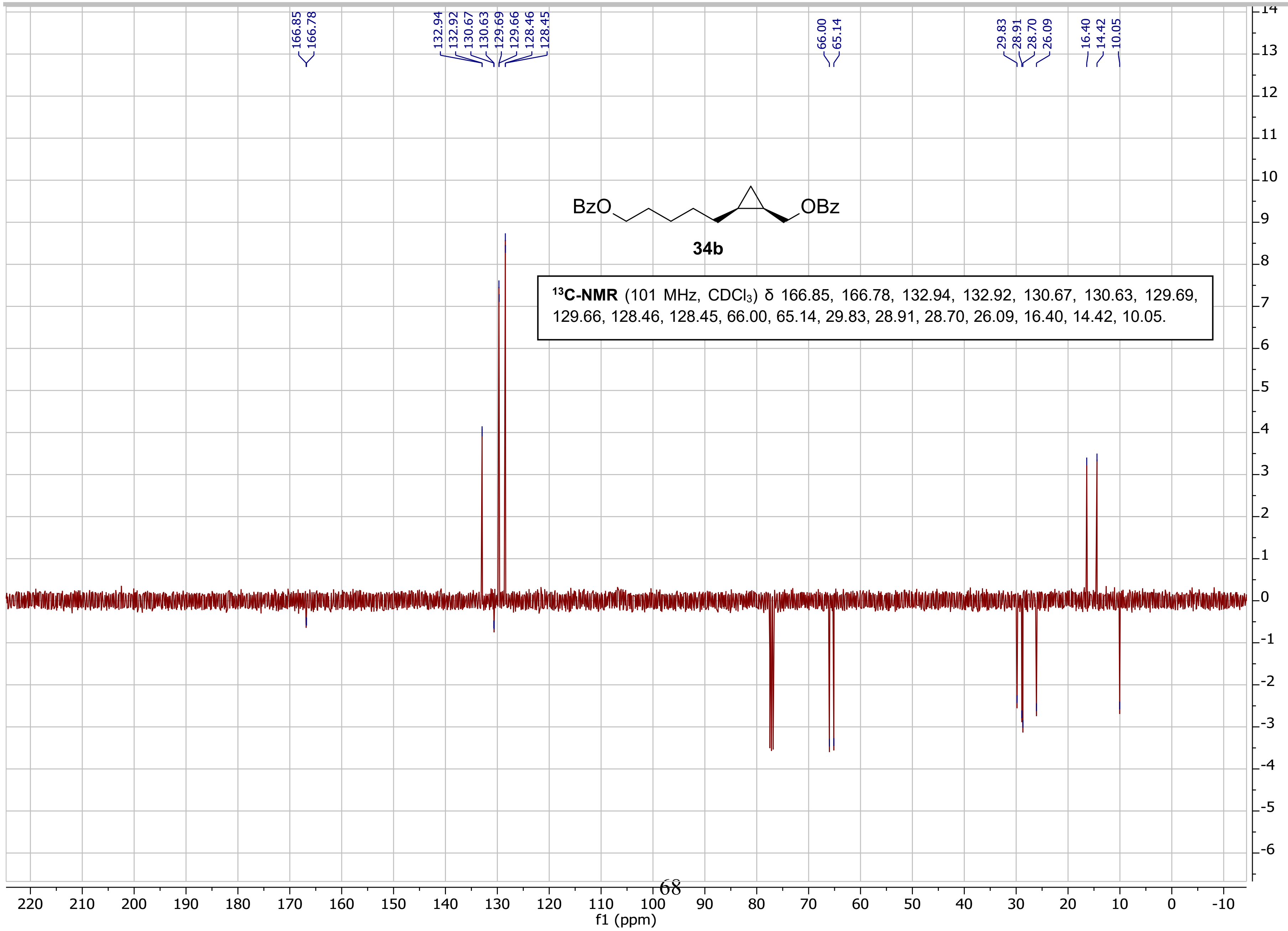


**$^{13}$C-NMR** (101 MHz, $CDCl_3$) δ 166.85, 166.78, 132.94, 132.92, 130.67, 130.63, 129.69, 129.66, 128.46, 128.45, 66.00, 65.14, 29.83, 28.91, 28.70, 26.09, 16.40, 14.42, 10.05.

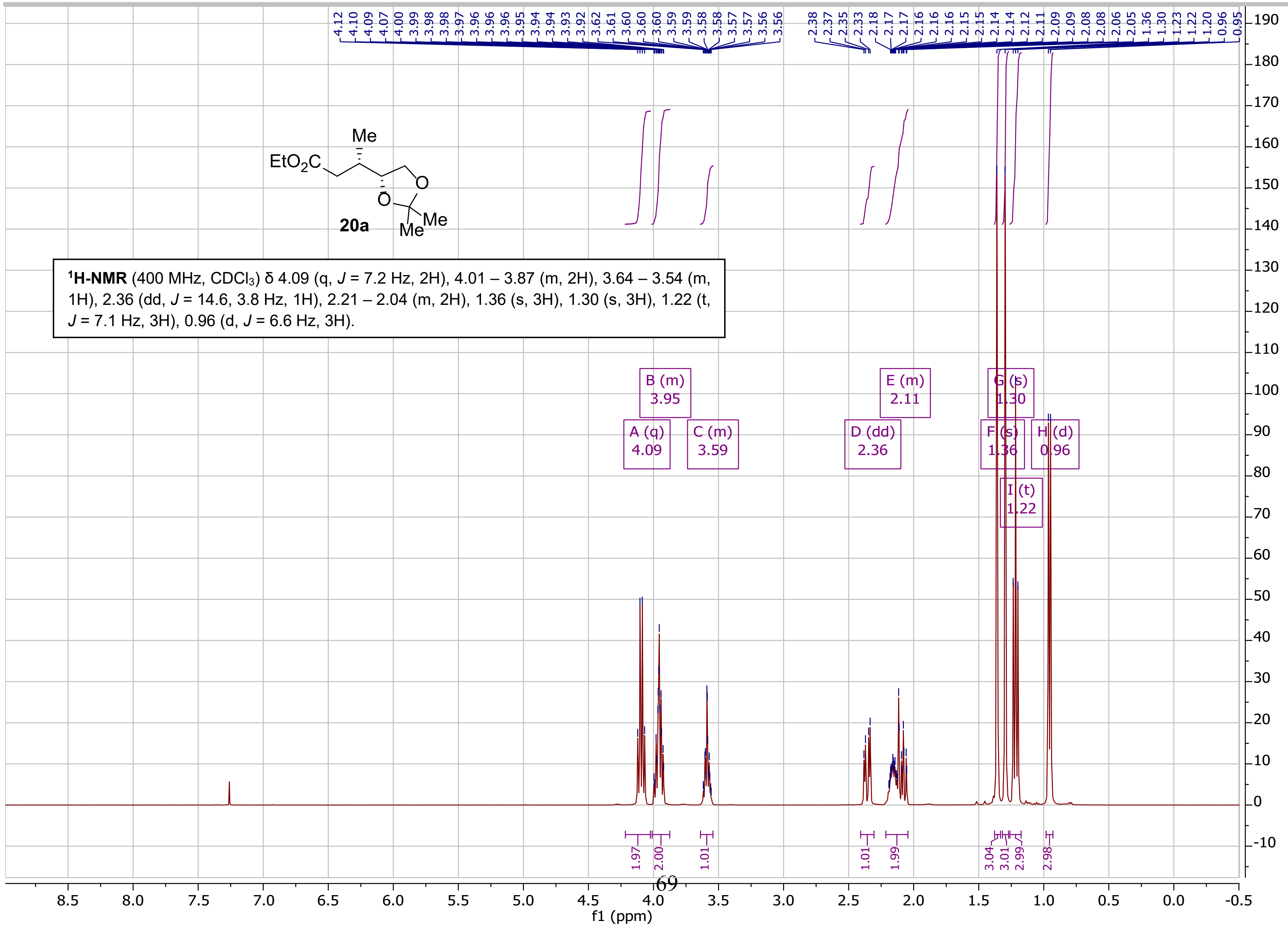


**$^1$H-NMR** (400 MHz, $CDCl_3$) δ 4.09 (q, *J* = 7.2 Hz, 2H), 4.01 – 3.87 (m, 2H), 3.64 – 3.54 (m, 1H), 2.36 (dd, *J* = 14.6, 3.8 Hz, 1H), 2.21 – 2.04 (m, 2H), 1.36 (s, 3H), 1.30 (s, 3H), 1.22 (t, *J* = 7.1 Hz, 3H), 0.96 (d, *J* = 6.6 Hz, 3H).

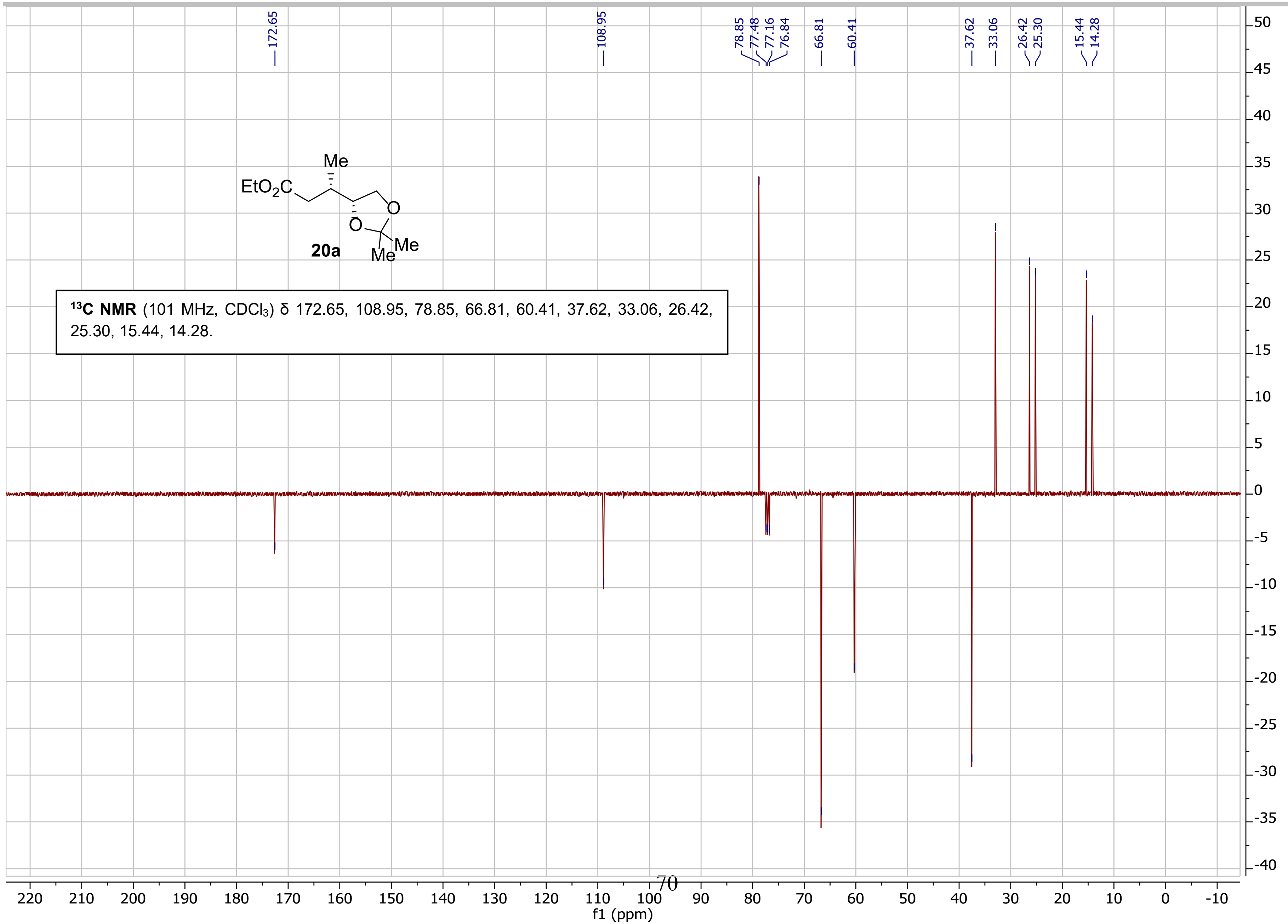


$^{13}$**C NMR** (101 MHz, $CDCl_3$) δ 172.65, 108.95, 78.85, 66.81, 60.41, 37.62, 33.06, 26.42, 25.30, 15.44, 14.28.

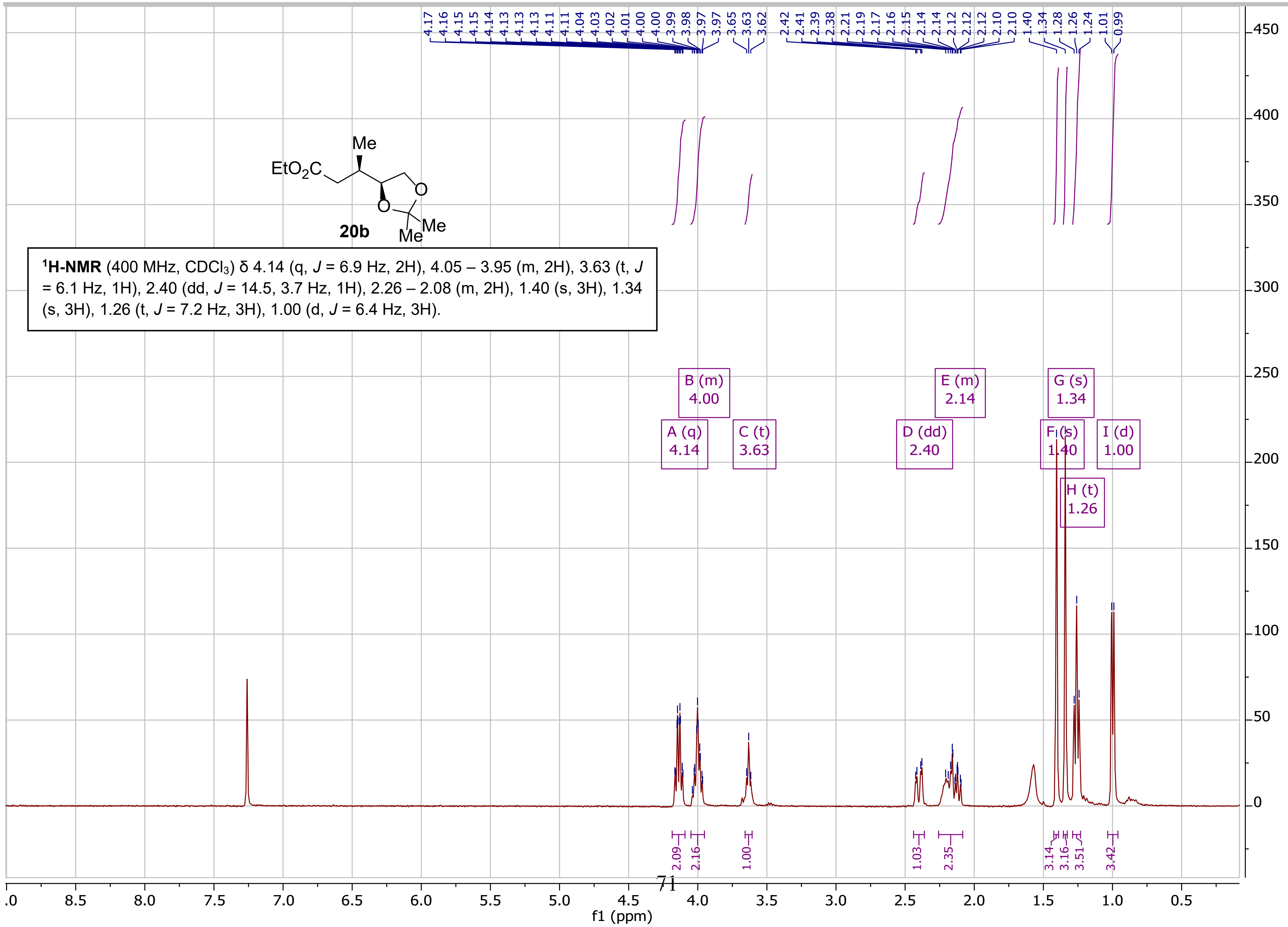


[1]**H-NMR** (400 MHz, $CDCl_3$) δ 4.14 (q, *J* = 6.9 Hz, 2H), 4.05 – 3.95 (m, 2H), 3.63 (t, *J* = 6.1 Hz, 1H), 2.40 (dd, *J* = 14.5, 3.7 Hz, 1H), 2.26 – 2.08 (m, 2H), 1.40 (s, 3H), 1.34 (s, 3H), 1.26 (t, *J* = 7.2 Hz, 3H), 1.00 (d, *J* = 6.4 Hz, 3H).

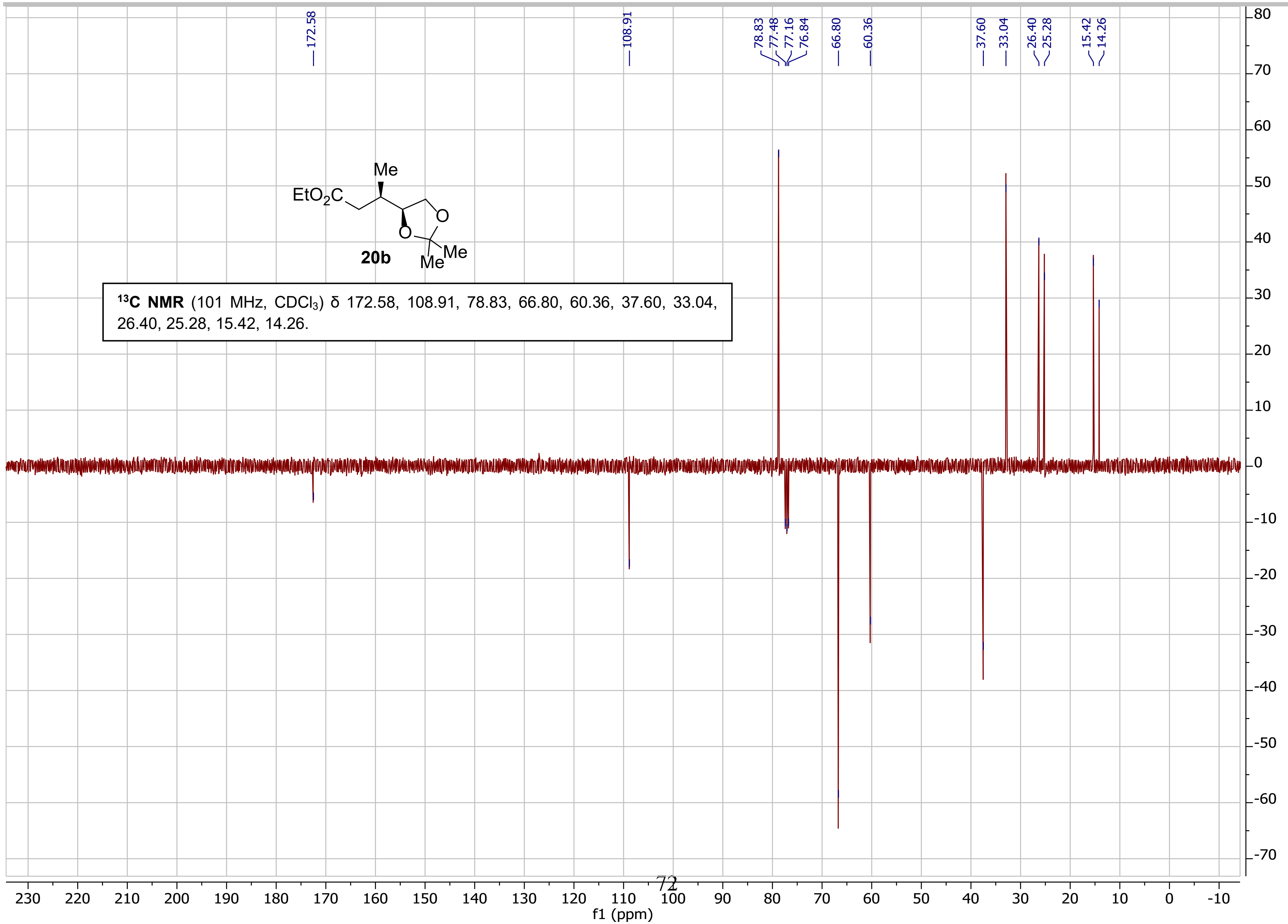


$^{13}$C NMR (101 MHz, $CDCl_3$) δ 172.58, 108.91, 78.83, 66.80, 60.36, 37.60, 33.04, 26.40, 25.28, 15.42, 14.26.

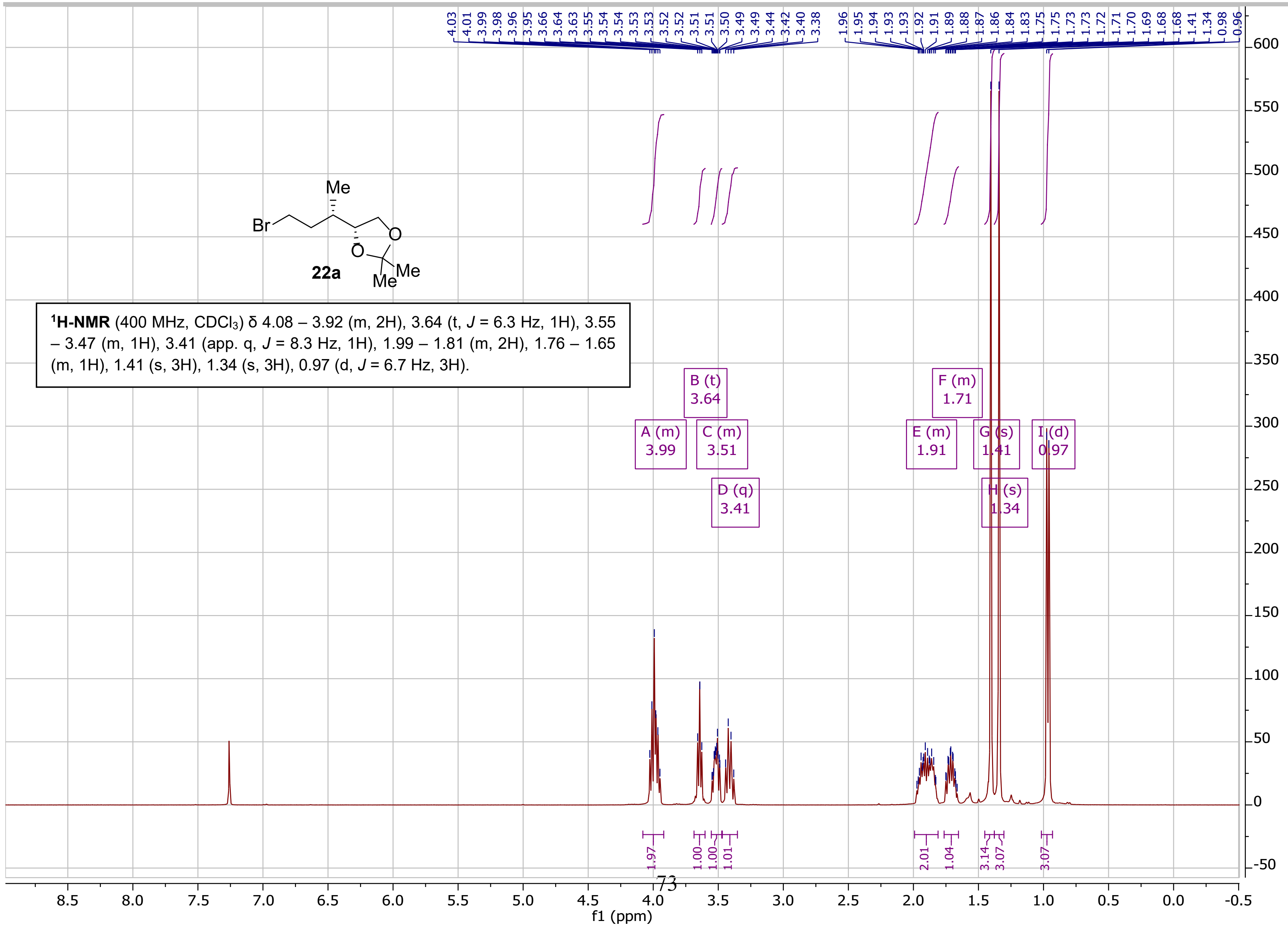


**$^{1}$H-NMR** (400 MHz, $CDCl_3$) δ 4.08 – 3.92 (m, 2H), 3.64 (t, *J* = 6.3 Hz, 1H), 3.55 – 3.47 (m, 1H), 3.41 (app. q, *J* = 8.3 Hz, 1H), 1.99 – 1.81 (m, 2H), 1.76 – 1.65 (m, 1H), 1.41 (s, 3H), 1.34 (s, 3H), 0.97 (d, *J* = 6.7 Hz, 3H).

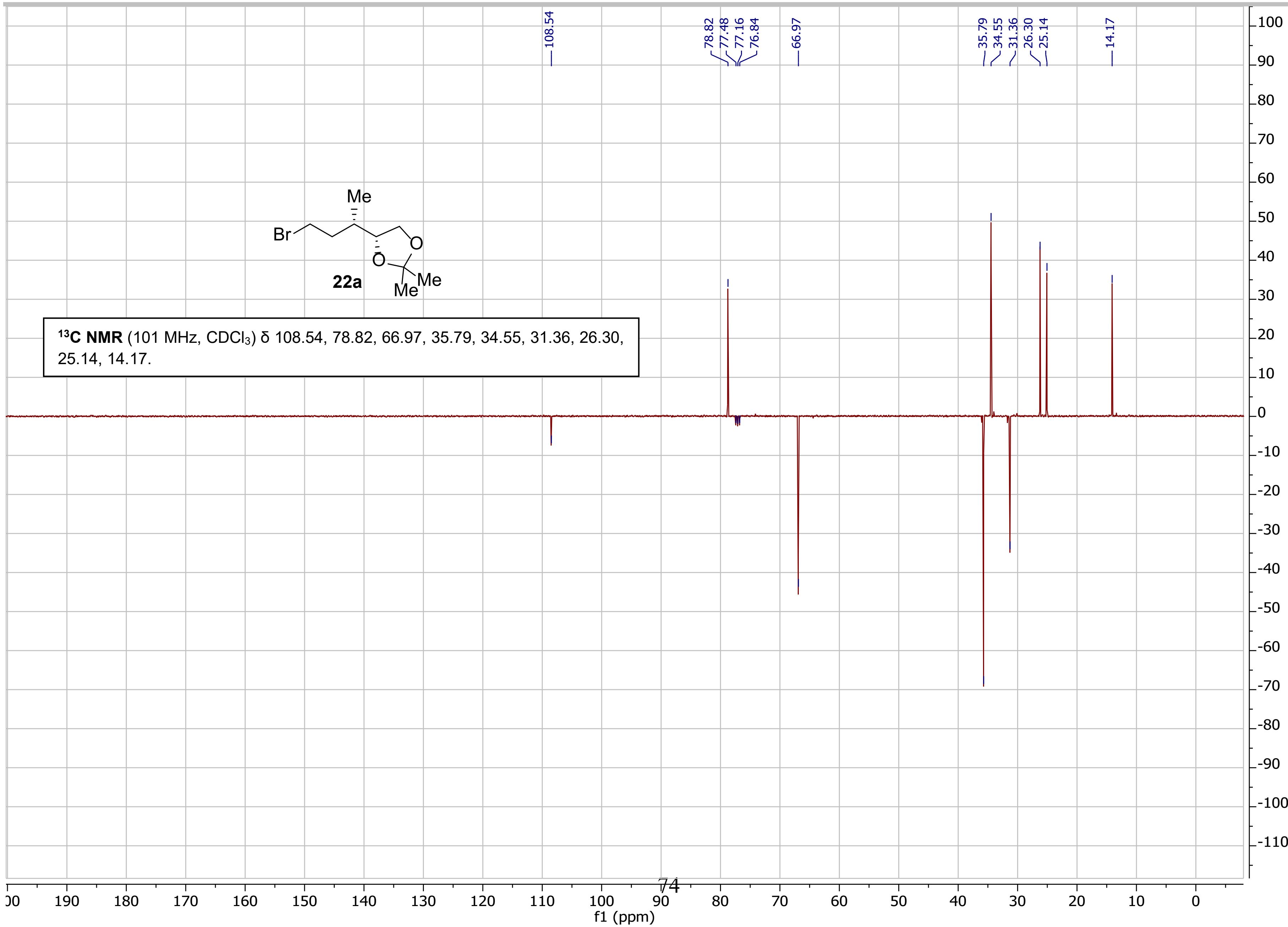


**$^{13}$C NMR** (101 MHz, $CDCl_3$) δ 108.54, 78.82, 66.97, 35.79, 34.55, 31.36, 26.30, 25.14, 14.17.

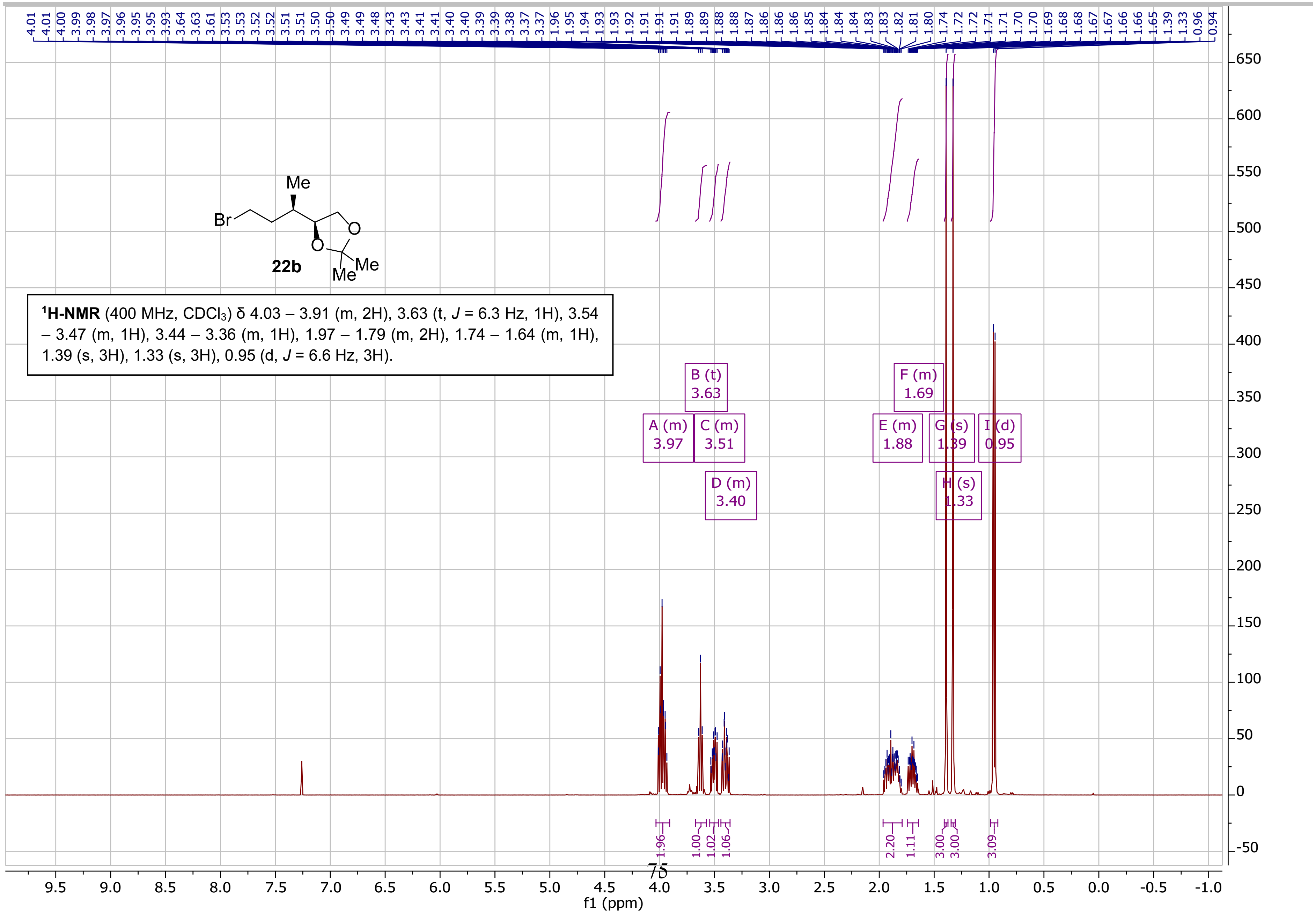

$^1$**H-NMR** (400 MHz, $CDCl_3$) δ 4.03 – 3.91 (m, 2H), 3.63 (t, *J* = 6.3 Hz, 1H), 3.54 – 3.47 (m, 1H), 3.44 – 3.36 (m, 1H), 1.97 – 1.79 (m, 2H), 1.74 – 1.64 (m, 1H), 1.39 (s, 3H), 1.33 (s, 3H), 0.95 (d, *J* = 6.6 Hz, 3H).

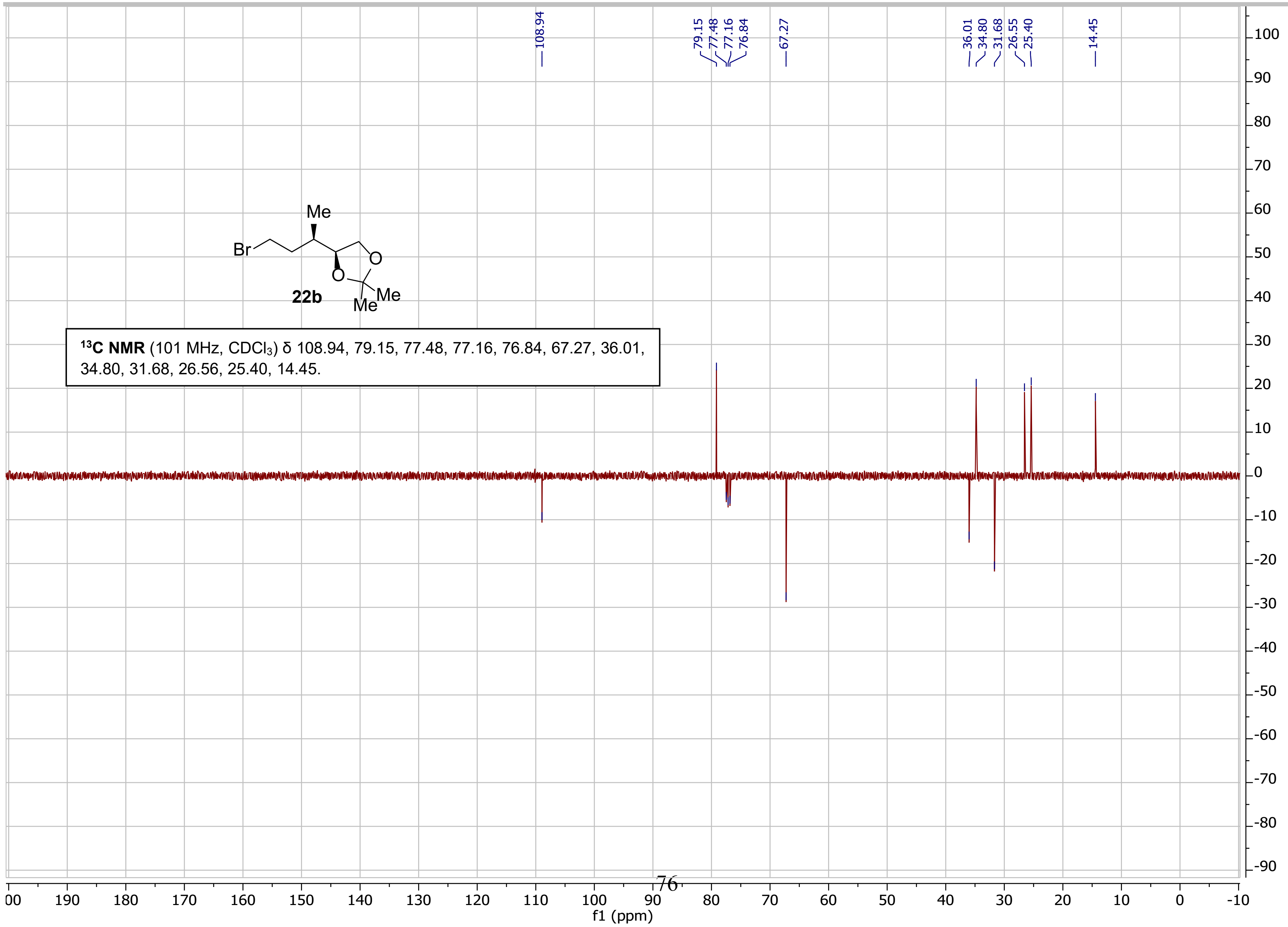


**22b**

**$^{13}$C NMR** (101 MHz, $CDCl_3$) δ 108.94, 79.15, 77.48, 77.16, 76.84, 67.27, 36.01, 34.80, 31.68, 26.56, 25.40, 14.45.

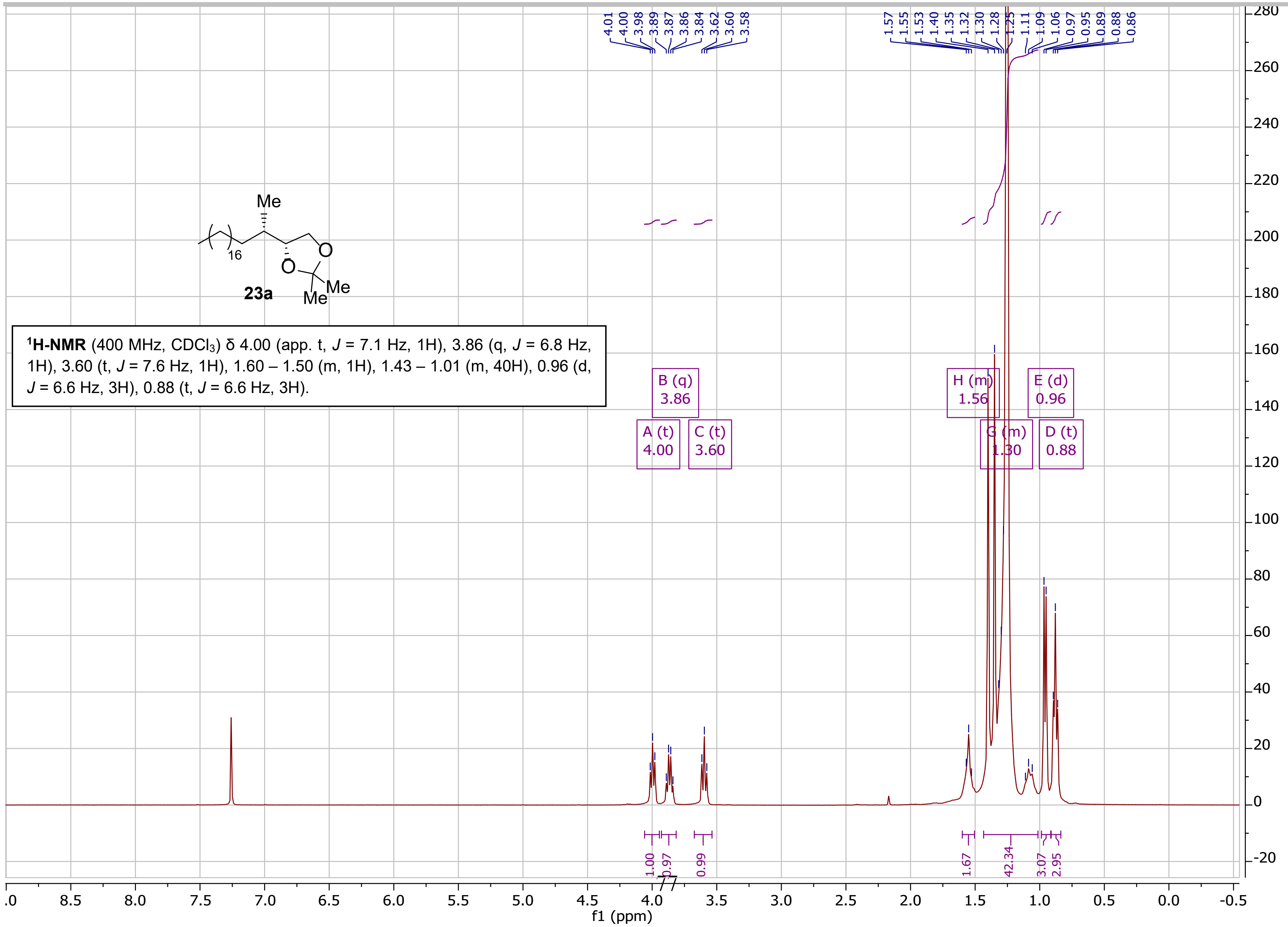


**$^1$H-NMR** (400 MHz, $CDCl_3$) δ 4.00 (app. t, $J$ = 7.1 Hz, 1H), 3.86 (q, $J$ = 6.8 Hz, 1H), 3.60 (t, $J$ = 7.6 Hz, 1H), 1.60 – 1.50 (m, 1H), 1.43 – 1.01 (m, 40H), 0.96 (d, $J$ = 6.6 Hz, 3H), 0.88 (t, $J$ = 6.6 Hz, 3H).

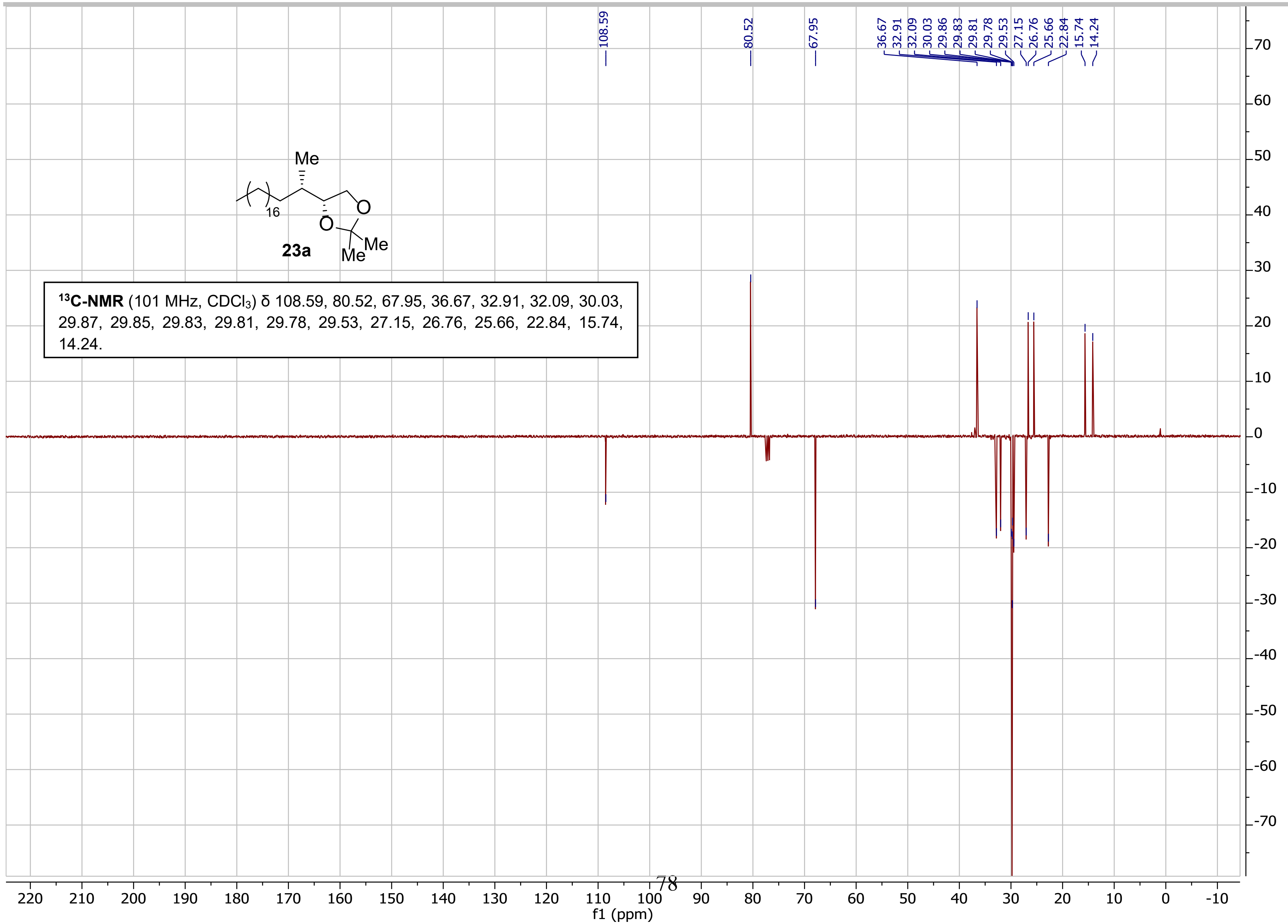


**23a**

**$^{13}$C-NMR** (101 MHz, $CDCl_3$) δ 108.59, 80.52, 67.95, 36.67, 32.91, 32.09, 30.03, 29.87, 29.85, 29.83, 29.81, 29.78, 29.53, 27.15, 26.76, 25.66, 22.84, 15.74, 14.24.

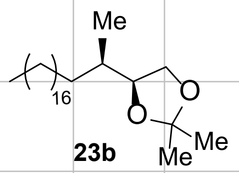


**23b**

**$^{1}$H-NMR** (400 MHz, $CDCl_3$) δ 4.00 (dd, *J* = 7.8, 6.2 Hz, 1H), 3.86 (q, *J* = 7.1 Hz, 1H), 3.59 (t, *J* = 7.7 Hz, 1H), 1.59 – 1.50 (m, 1H), 1.44 – 1.01 (m, 40H), 0.96 (d, *J* = 6.7 Hz, 3H), 0.88 (t, *J* = 6.7 Hz, 3H).

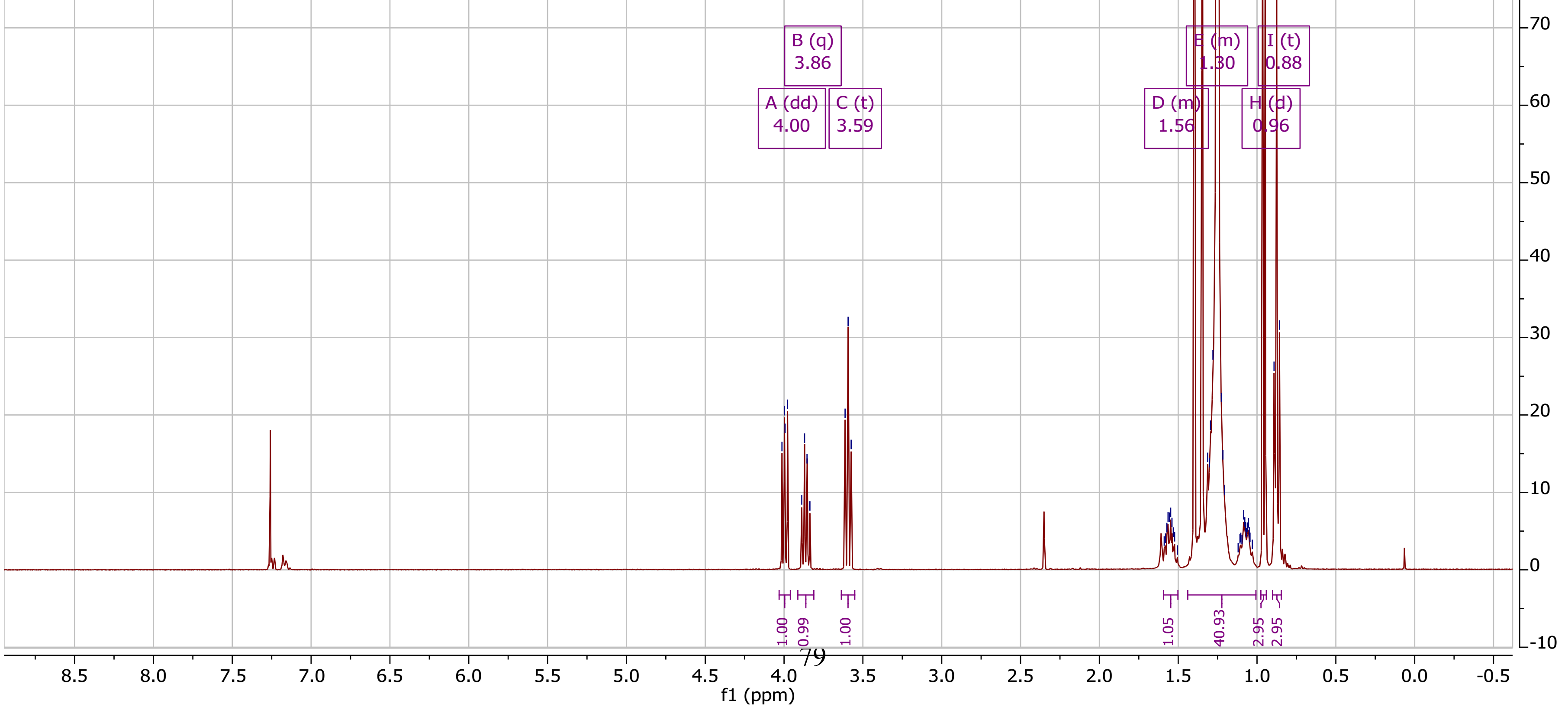

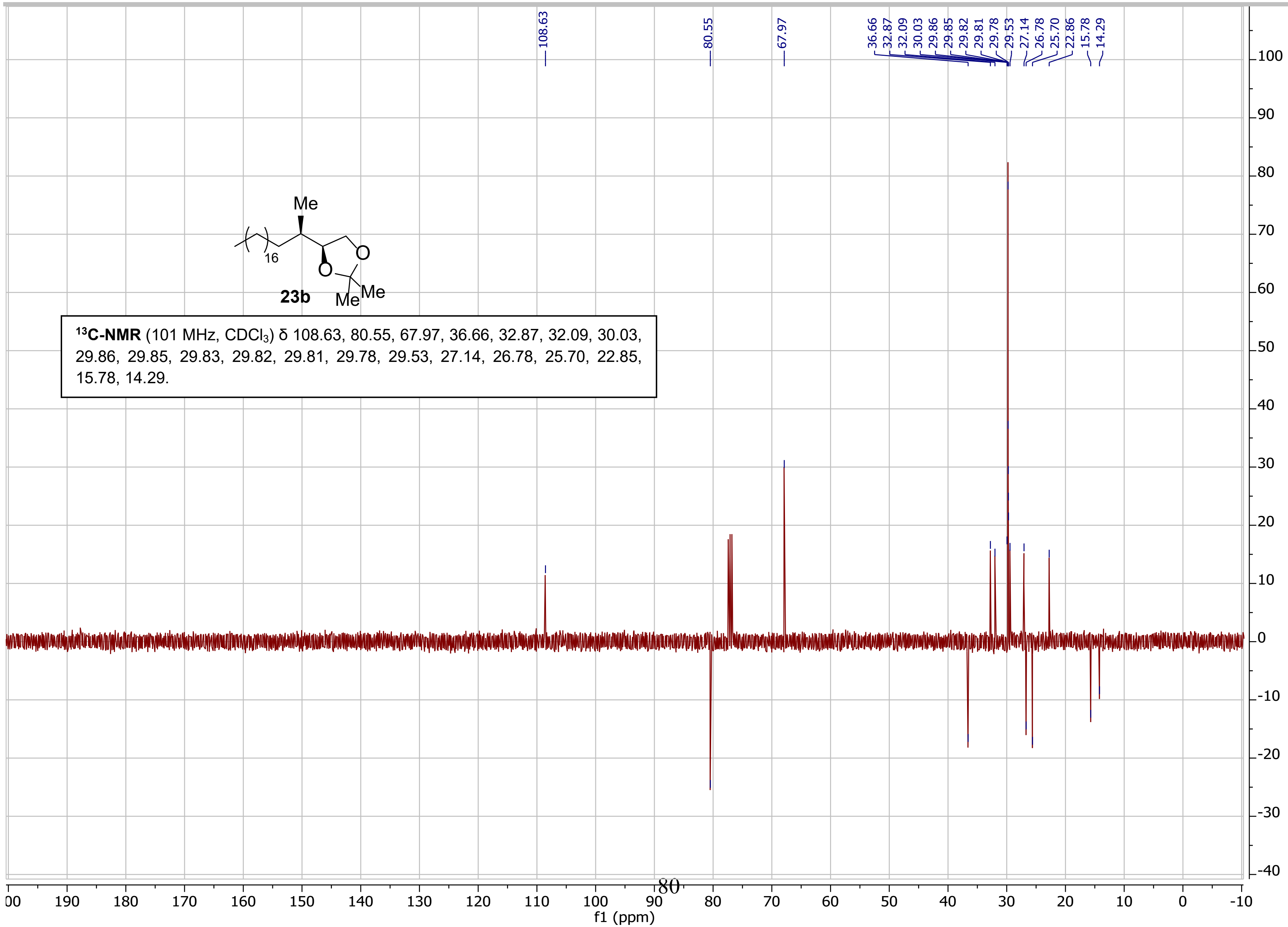


**23b**

**$^{13}$C-NMR** (101 MHz, $CDCl_3$) δ 108.63, 80.55, 67.97, 36.66, 32.87, 32.09, 30.03, 29.86, 29.85, 29.83, 29.82, 29.81, 29.78, 29.53, 27.14, 26.78, 25.70, 22.85, 15.78, 14.29.

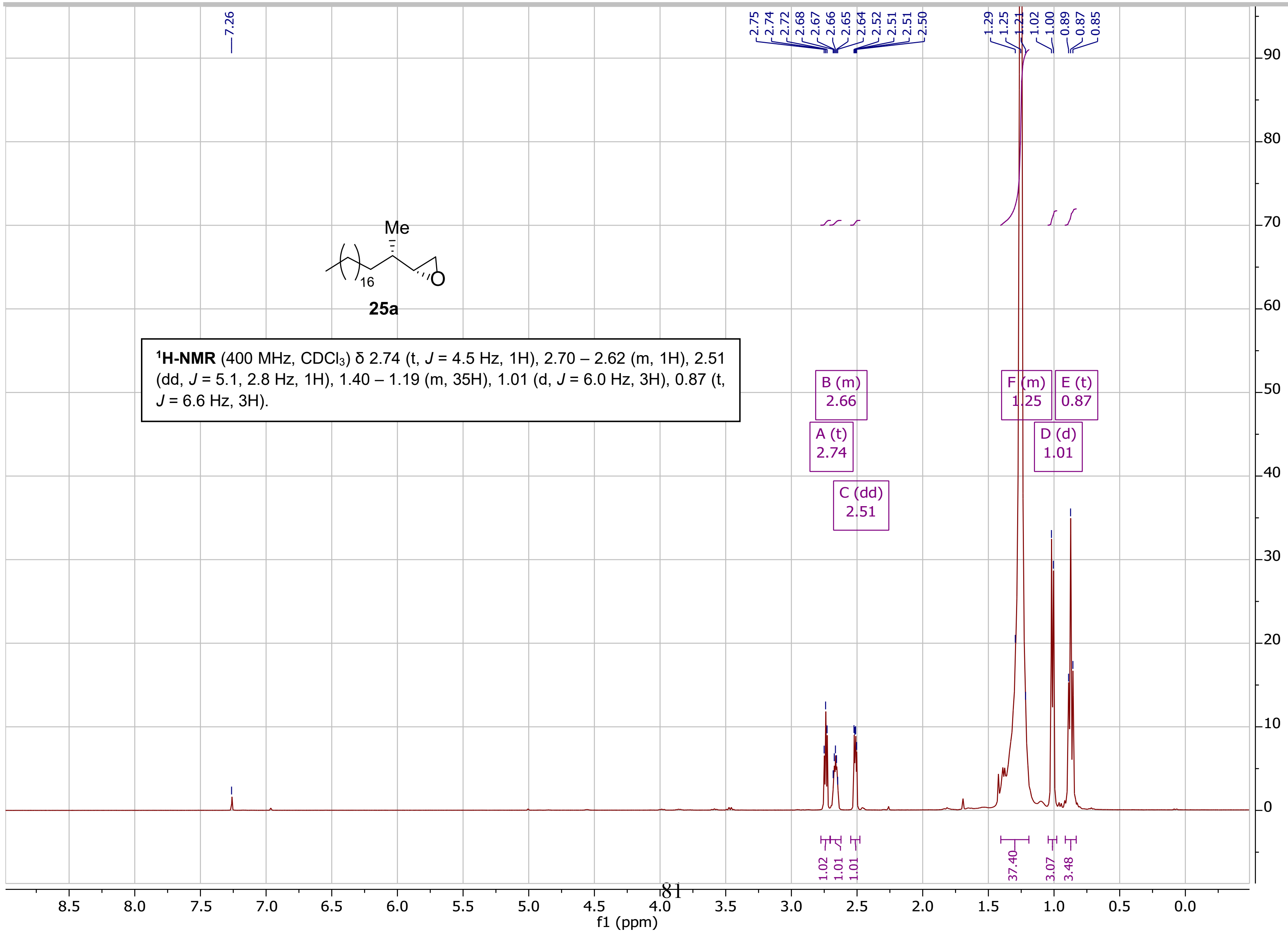


$^{1}$H-NMR (400 MHz, $CDCl_3$) δ 2.74 (t, *J* = 4.5 Hz, 1H), 2.70 – 2.62 (m, 1H), 2.51 (dd, *J* = 5.1, 2.8 Hz, 1H), 1.40 – 1.19 (m, 35H), 1.01 (d, *J* = 6.0 Hz, 3H), 0.87 (t, *J* = 6.6 Hz, 3H).

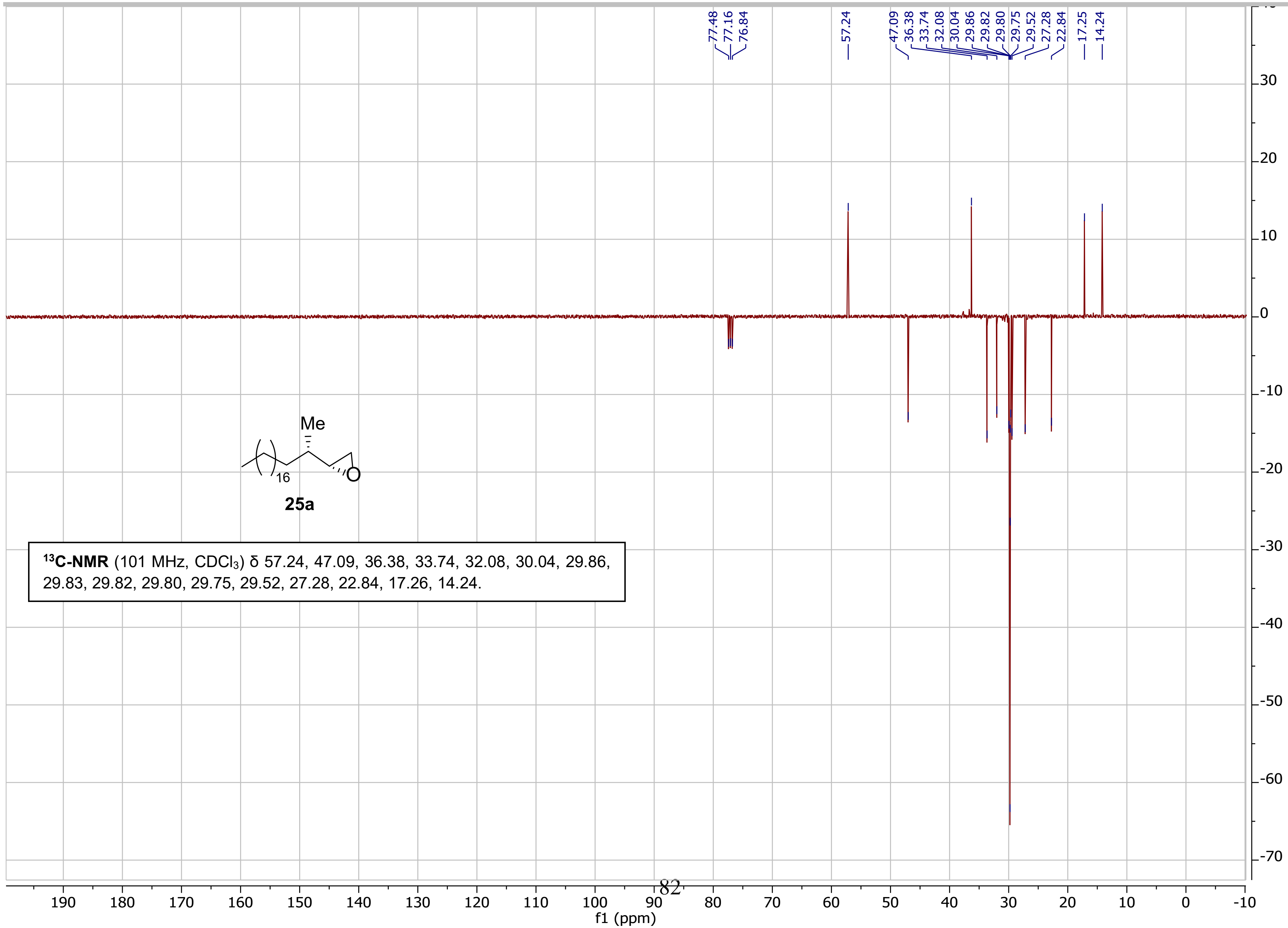


**$^{13}$C-NMR** (101 MHz, $CDCl_3$) δ 57.24, 47.09, 36.38, 33.74, 32.08, 30.04, 29.86, 29.83, 29.82, 29.80, 29.75, 29.52, 27.28, 22.84, 17.26, 14.24.

**25b**

**$^{1}$H-NMR** (400 MHz, $CDCl_3$) δ 2.75 (dd, *J* = 5.0, 3.9 Hz, 1H), 2.70 – 2.65 (m, 1H), 2.52 (dd, *J* = 5.0, 2.8 Hz, 1H), 1.42 – 1.18 (m, 35H), 1.02 (d, *J* = 6.2 Hz, 3H), 0.88 (t, *J* = 6.9 Hz, 3H).

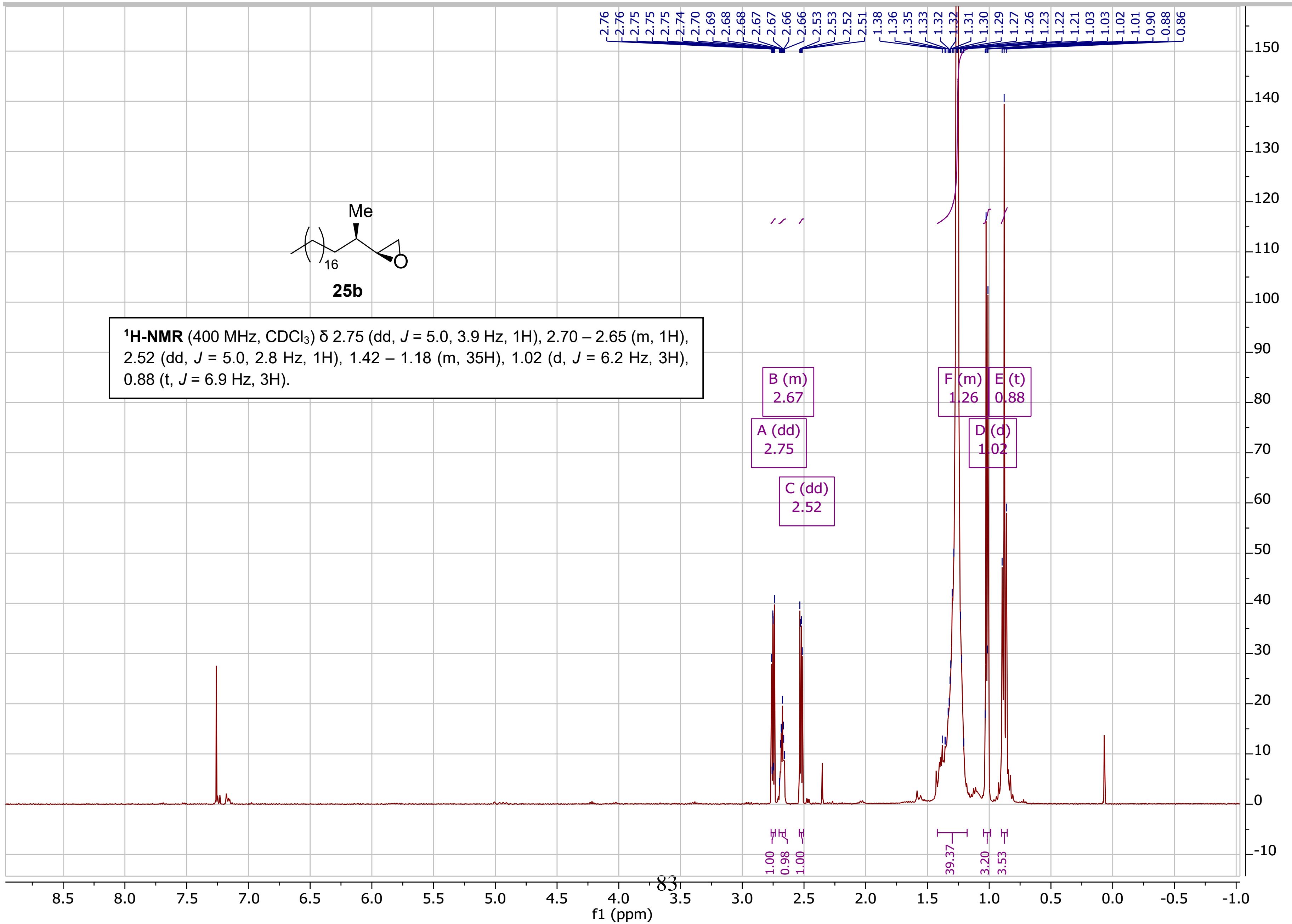

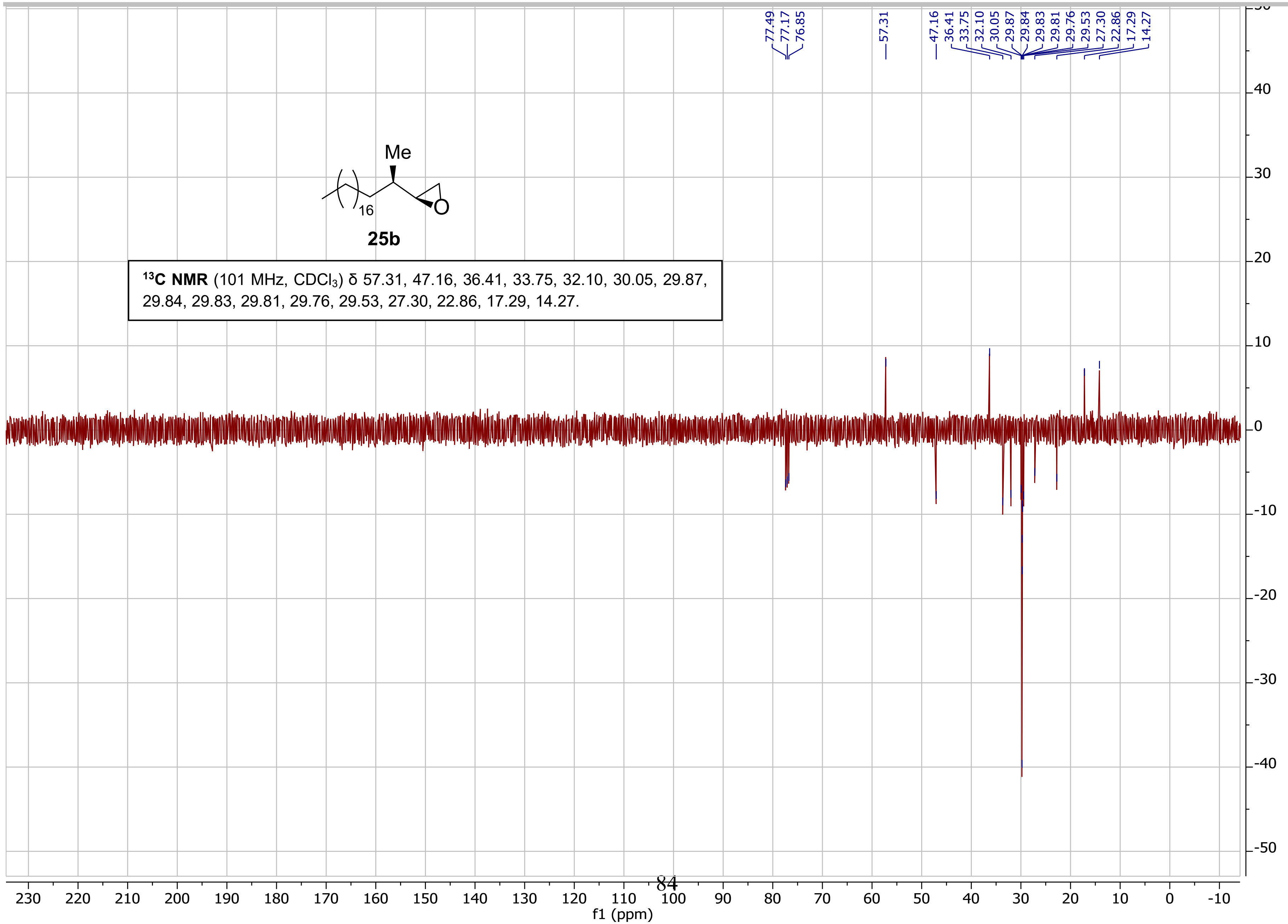


**$^{13}$C NMR** (101 MHz, $CDCl_3$) δ 57.31, 47.16, 36.41, 33.75, 32.10, 30.05, 29.87, 29.84, 29.83, 29.81, 29.76, 29.53, 27.30, 22.86, 17.29, 14.27.

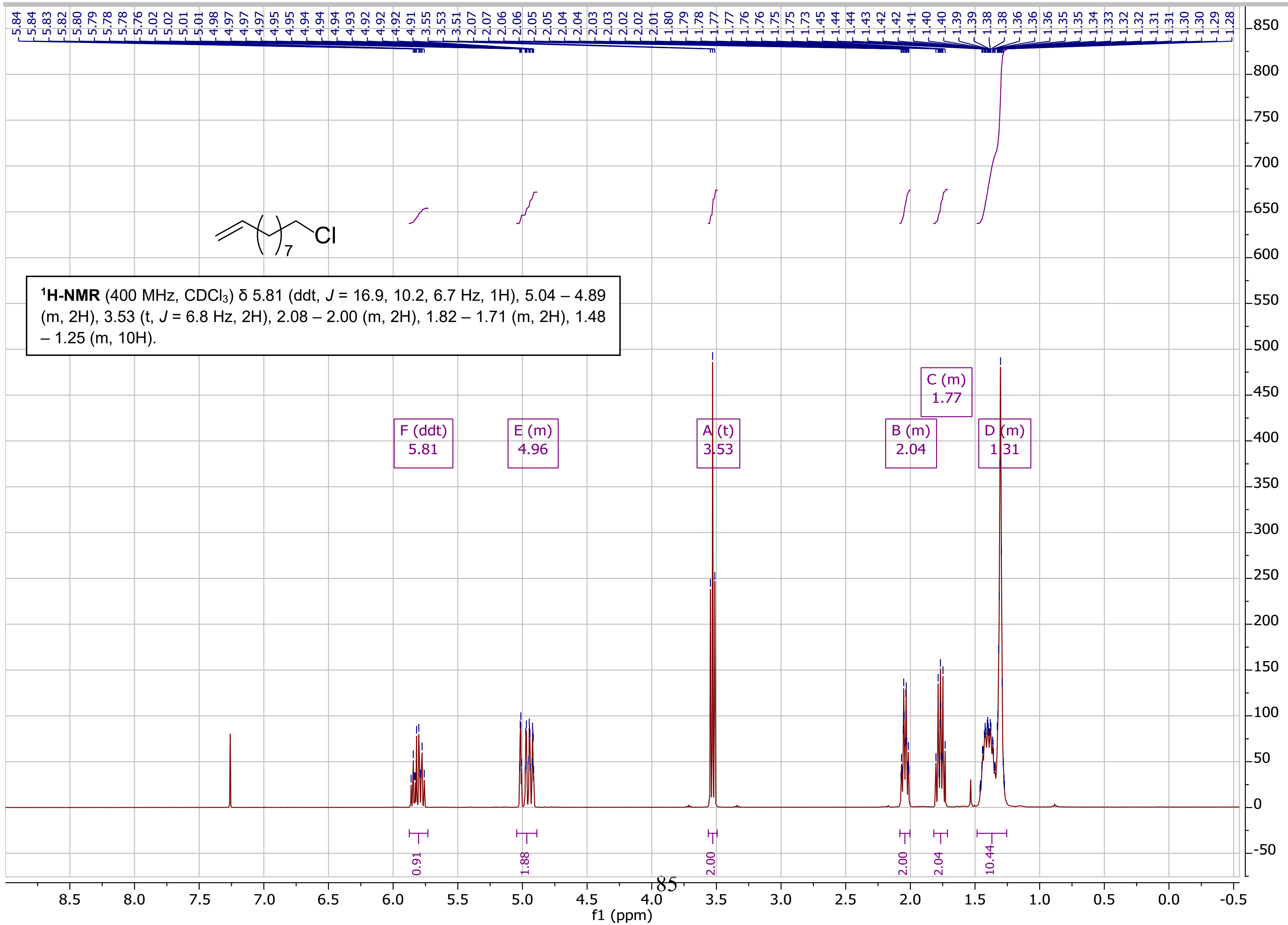
$^{1}$H-NMR (400 MHz, CDCl3) δ 5.81 (ddt, J = 16.9, 10.2, 6.7 Hz, 1H), 5.04 – 4.89 (m, 2H), 3.53 (t, J = 6.8 Hz, 2H), 2.08 – 2.00 (m, 2H), 1.82 – 1.71 (m, 2H), 1.48 – 1.25 (m, 10H).
Cl
7
F (ddt) 5.81
E (m) 4.96
A (t) 3.53
B (m) 2.04
C (m) 1.77
D (m) 1.31
0.91
1.88
2.00
2.00
2.04
10.44
f1 (ppm)

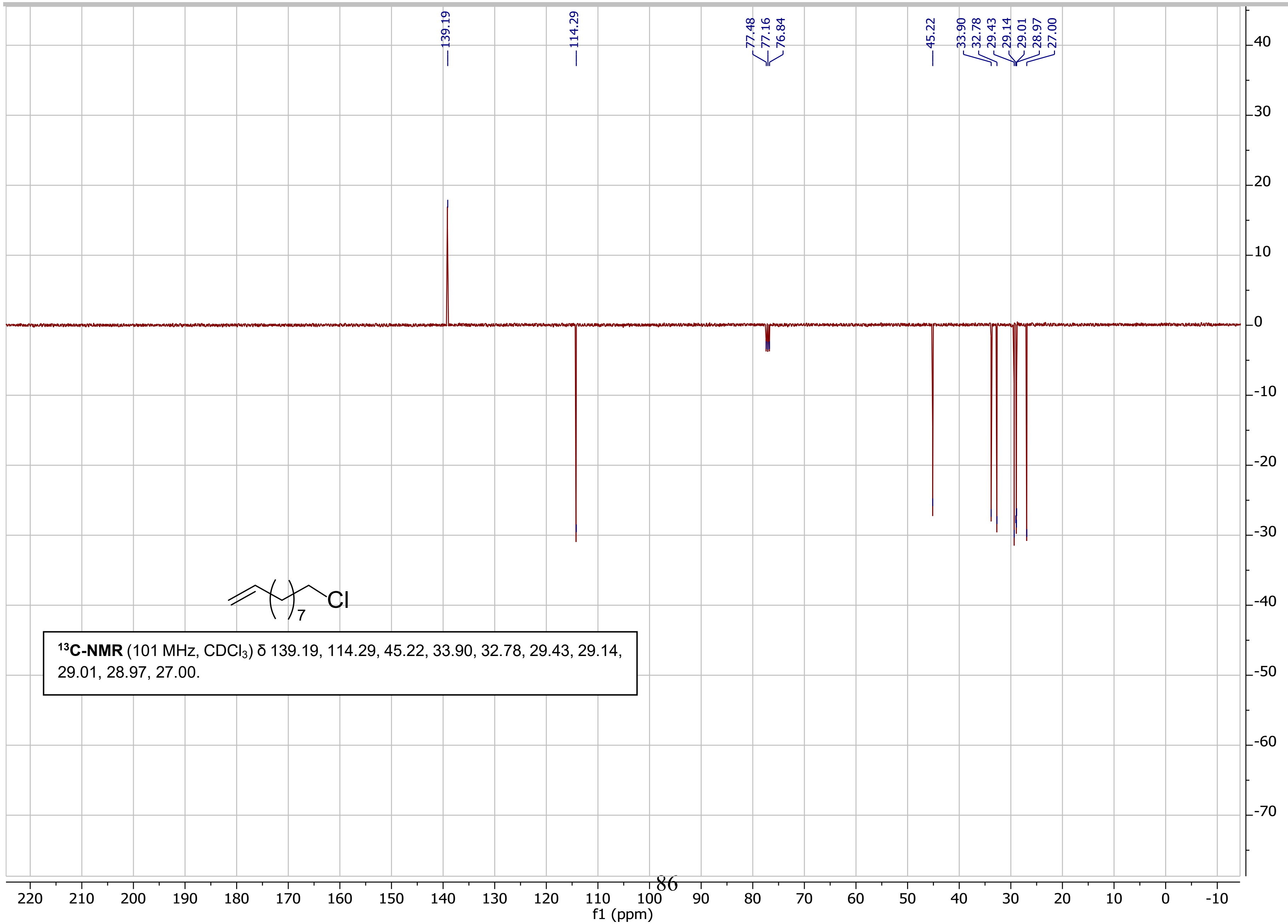


**$^{13}$C-NMR** (101 MHz, $CDCl_3$) δ 139.19, 114.29, 45.22, 33.90, 32.78, 29.43, 29.14, 29.01, 28.97, 27.00.

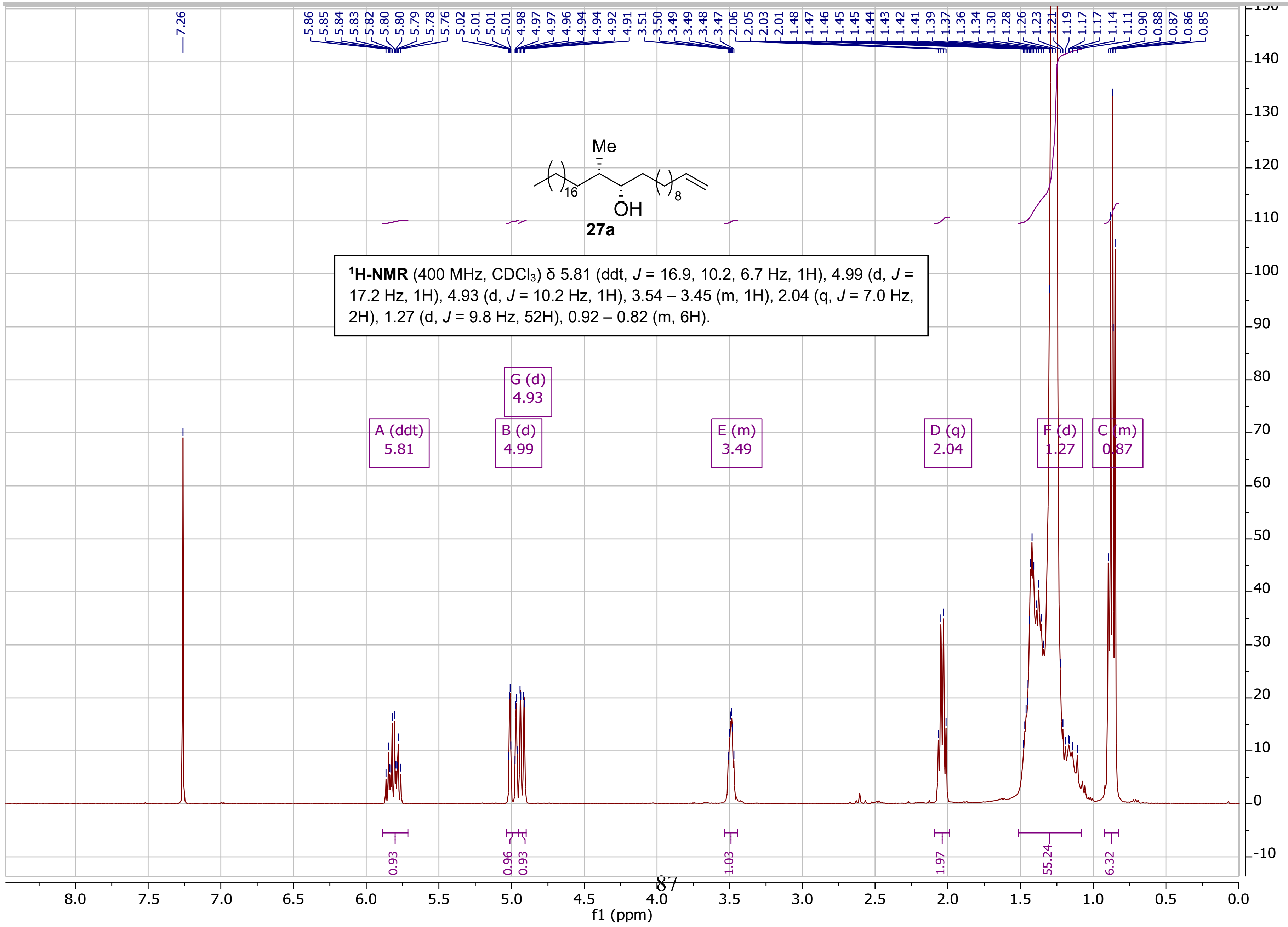

27a

**1H-NMR** (400 MHz, $CDCl_3$) δ 5.81 (ddt, *J* = 16.9, 10.2, 6.7 Hz, 1H), 4.99 (d, *J* = 17.2 Hz, 1H), 4.93 (d, *J* = 10.2 Hz, 1H), 3.54 – 3.45 (m, 1H), 2.04 (q, *J* = 7.0 Hz, 2H), 1.27 (d, *J* = 9.8 Hz, 52H), 0.92 – 0.82 (m, 6H).

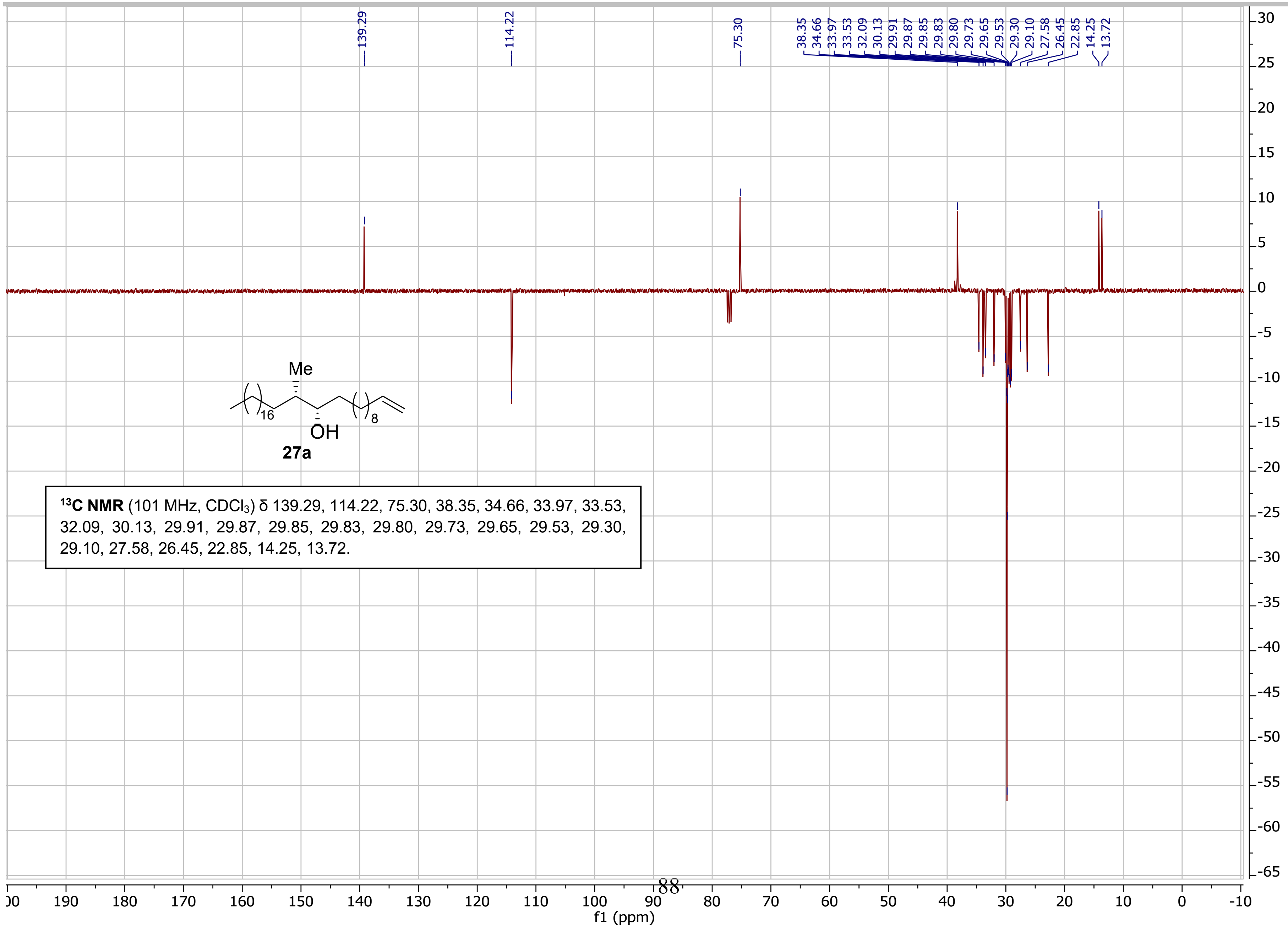


**27a**

**$^{13}$C NMR** (101 MHz, $CDCl_3$) δ 139.29, 114.22, 75.30, 38.35, 34.66, 33.97, 33.53, 32.09, 30.13, 29.91, 29.87, 29.85, 29.83, 29.80, 29.73, 29.65, 29.53, 29.30, 29.10, 27.58, 26.45, 22.85, 14.25, 13.72.

27b

**$^1$H-NMR** (400 MHz, $CDCl_3$) δ 5.81 (ddt, *J* = 16.9, 10.2, 6.7 Hz, 1H), 4.99 (dq, *J* = 17.1, 1.7 Hz, 1H), 4.93 (ddt, *J* = 10.2, 2.3, 1.2 Hz, 1H), 3.54 – 3.44 (m, 1H), 2.09 – 1.99 (m, 2H), 1.52 – 1.11 (m, 52H), 0.91 – 0.83 (m, 6H).

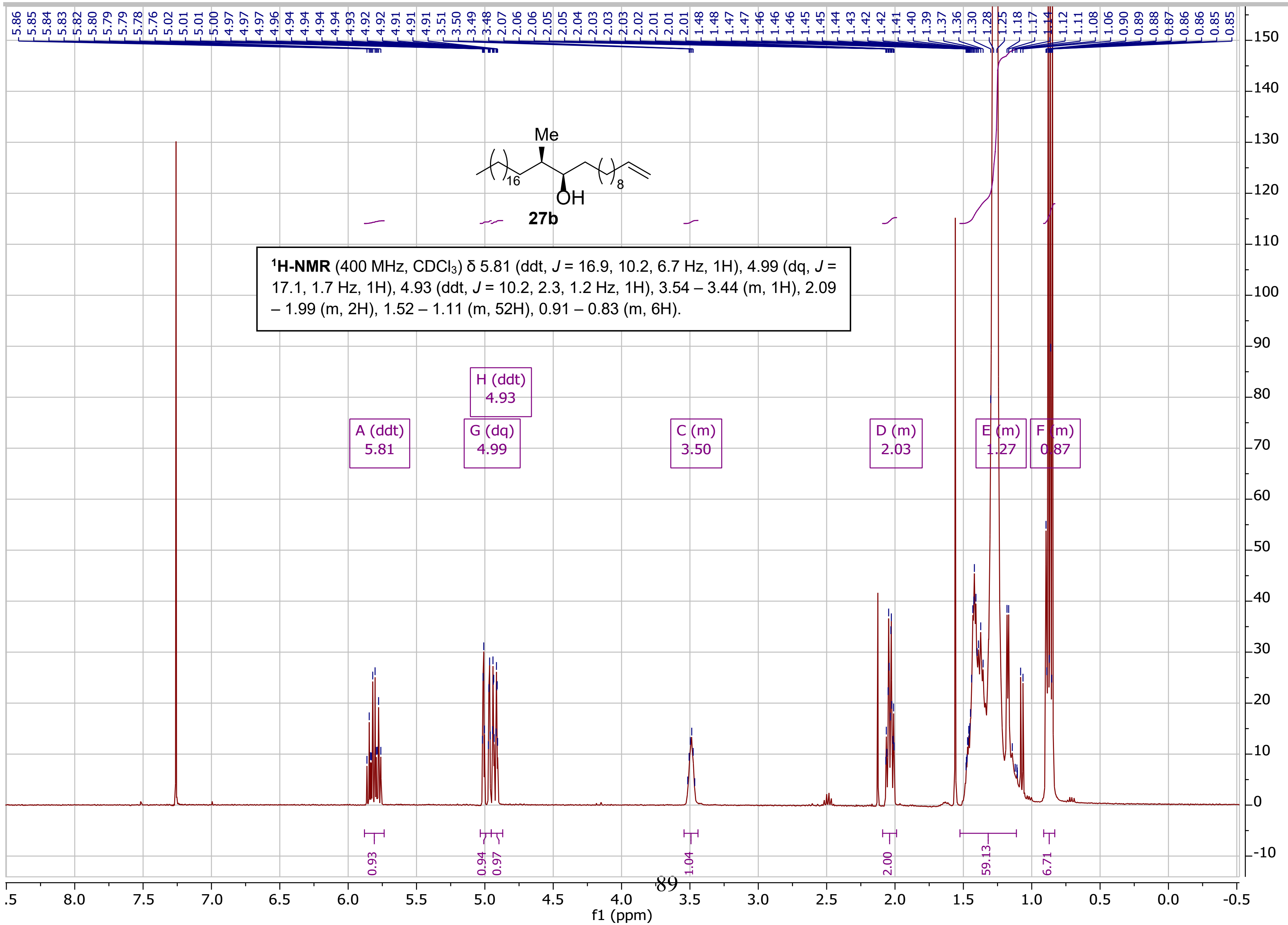

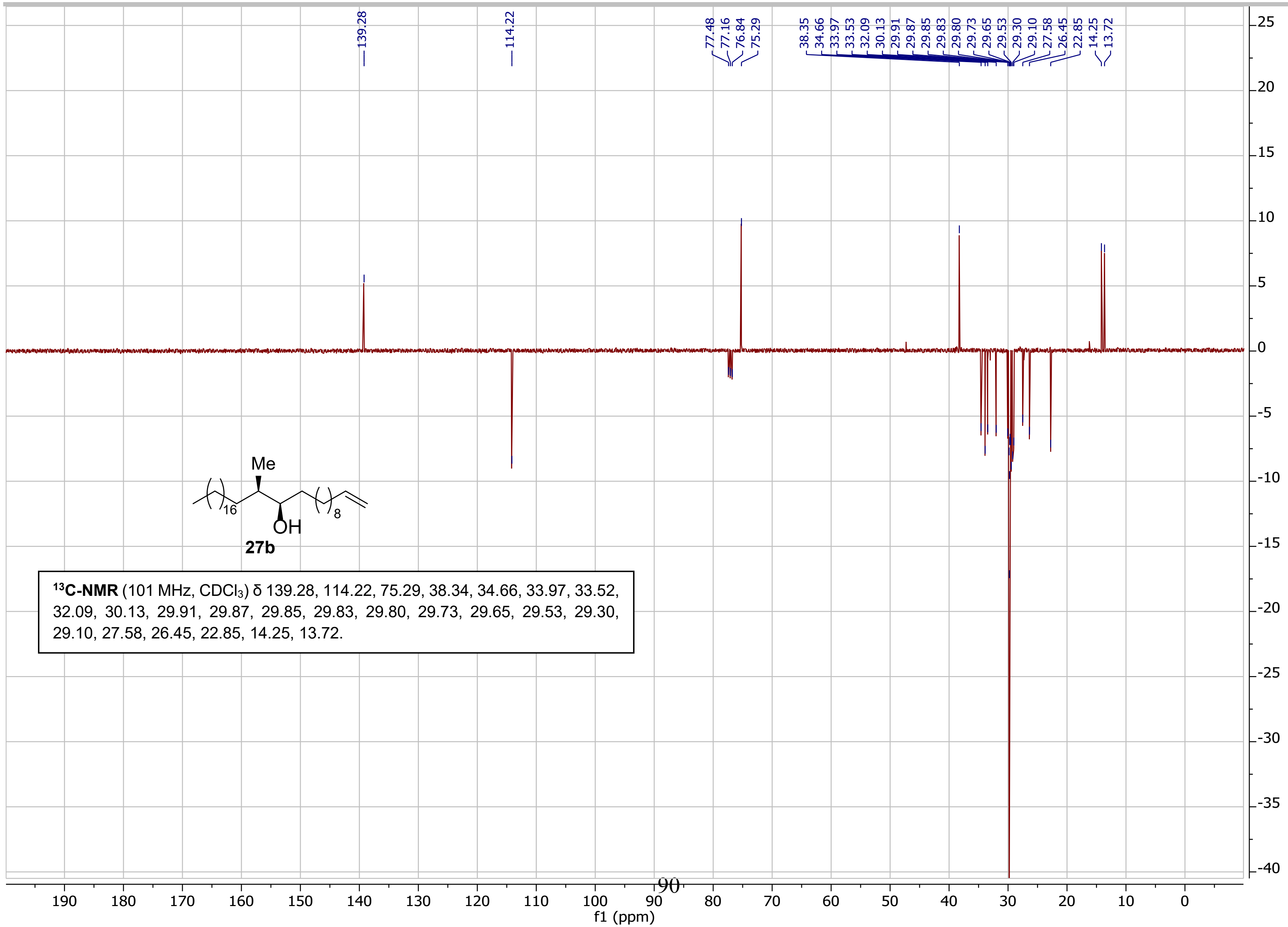


**27b**

**$^{13}$C-NMR** (101 MHz, $CDCl_3$) δ 139.28, 114.22, 75.29, 38.34, 34.66, 33.97, 33.52, 32.09, 30.13, 29.91, 29.87, 29.85, 29.83, 29.80, 29.73, 29.65, 29.53, 29.30, 29.10, 27.58, 26.45, 22.85, 14.25, 13.72.

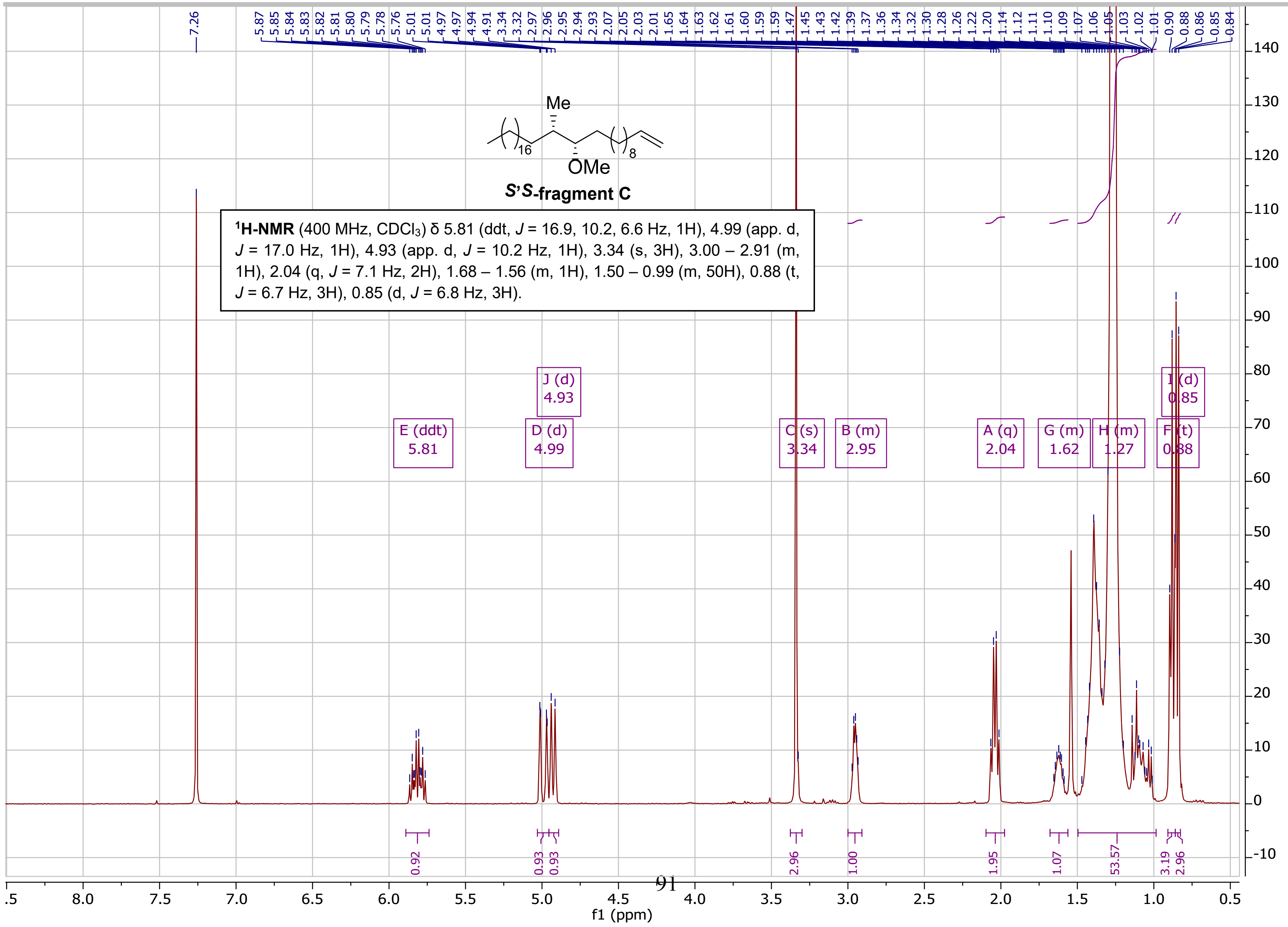


**¹H-NMR** (400 MHz, $CDCl_3$) δ 5.81 (ddt, *J* = 16.9, 10.2, 6.6 Hz, 1H), 4.99 (app. d, *J* = 17.0 Hz, 1H), 4.93 (app. d, *J* = 10.2 Hz, 1H), 3.34 (s, 3H), 3.00 – 2.91 (m, 1H), 2.04 (q, *J* = 7.1 Hz, 2H), 1.68 – 1.56 (m, 1H), 1.50 – 0.99 (m, 50H), 0.88 (t, *J* = 6.7 Hz, 3H), 0.85 (d, *J* = 6.8 Hz, 3H).

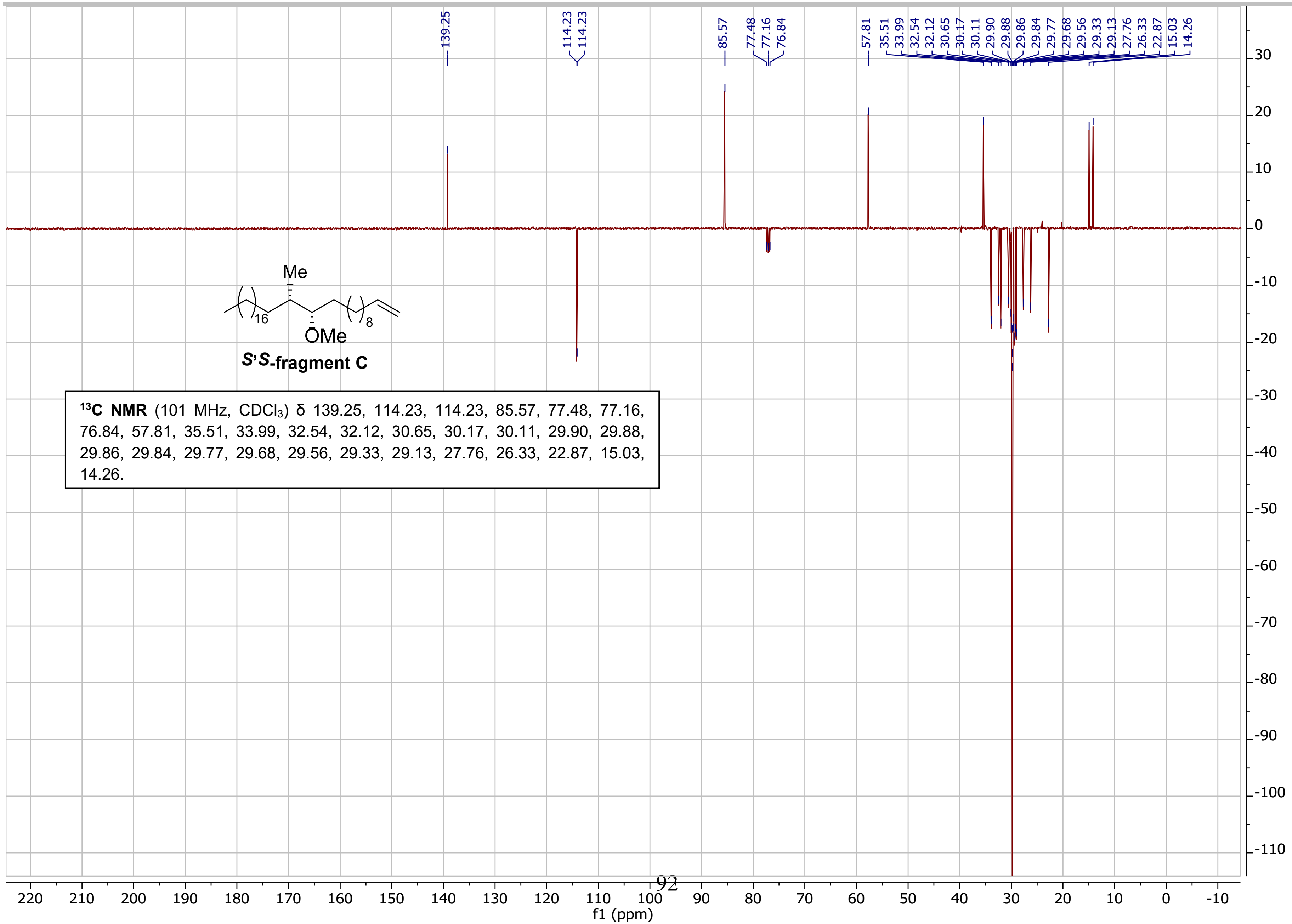


$^{13}$**C NMR** (101 MHz, $CDCl_3$) δ 139.25, 114.23, 114.23, 85.57, 77.48, 77.16, 76.84, 57.81, 35.51, 33.99, 32.54, 32.12, 30.65, 30.17, 30.11, 29.90, 29.88, 29.86, 29.84, 29.77, 29.68, 29.56, 29.33, 29.13, 27.76, 26.33, 22.87, 15.03, 14.26.

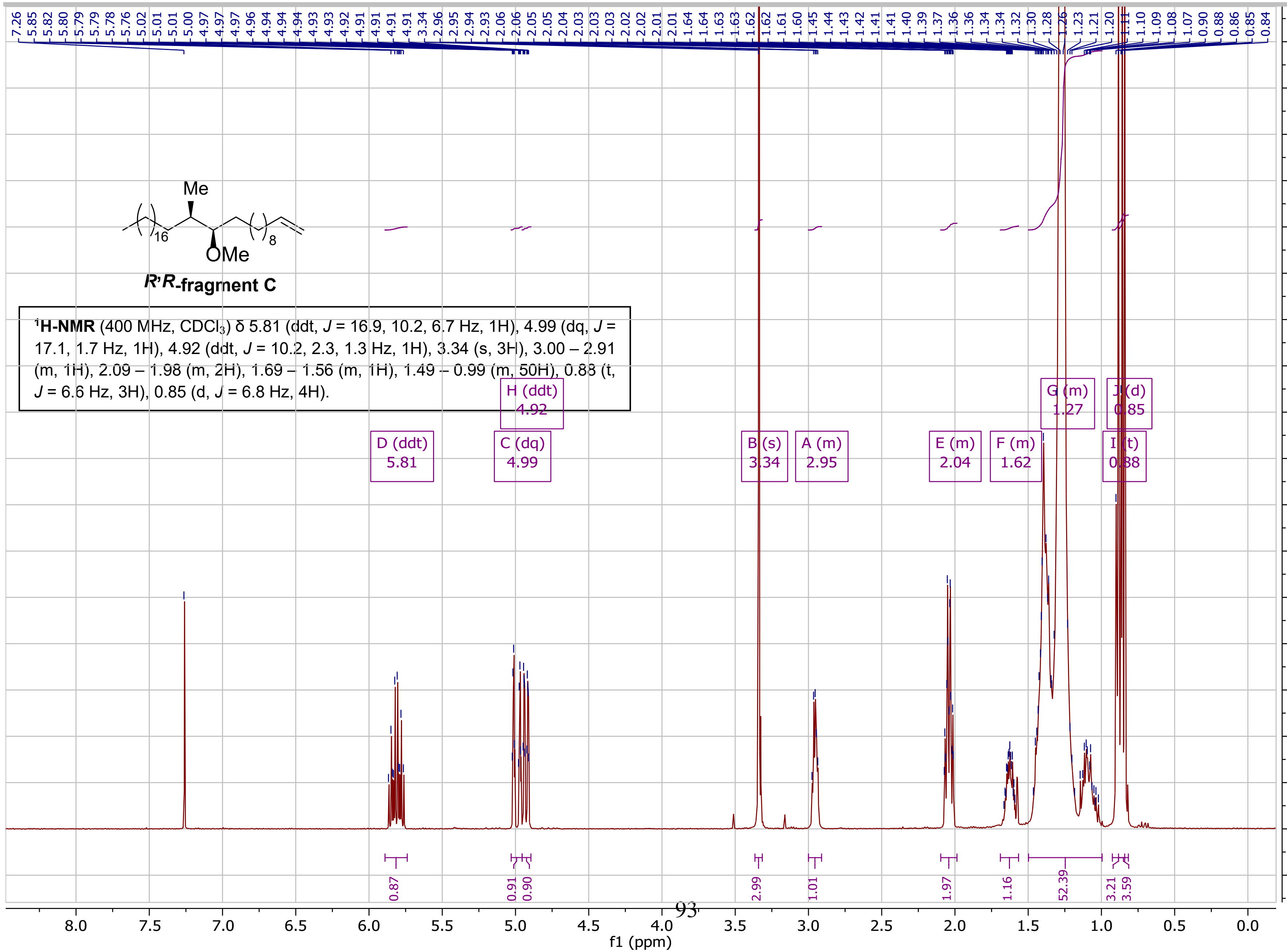


**R,R-fragment C**

**$^{1}$H-NMR** (400 MHz, $CDCl_3$) δ 5.81 (ddt, *J* = 16.9, 10.2, 6.7 Hz, 1H), 4.99 (dq, *J* = 17.1, 1.7 Hz, 1H), 4.92 (ddt, *J* = 10.2, 2.3, 1.3 Hz, 1H), 3.34 (s, 3H), 3.00 – 2.91 (m, 1H), 2.09 – 1.98 (m, 2H), 1.69 – 1.56 (m, 1H), 1.49 – 0.99 (m, 50H), 0.88 (t, *J* = 6.6 Hz, 3H), 0.85 (d, *J* = 6.8 Hz, 4H).

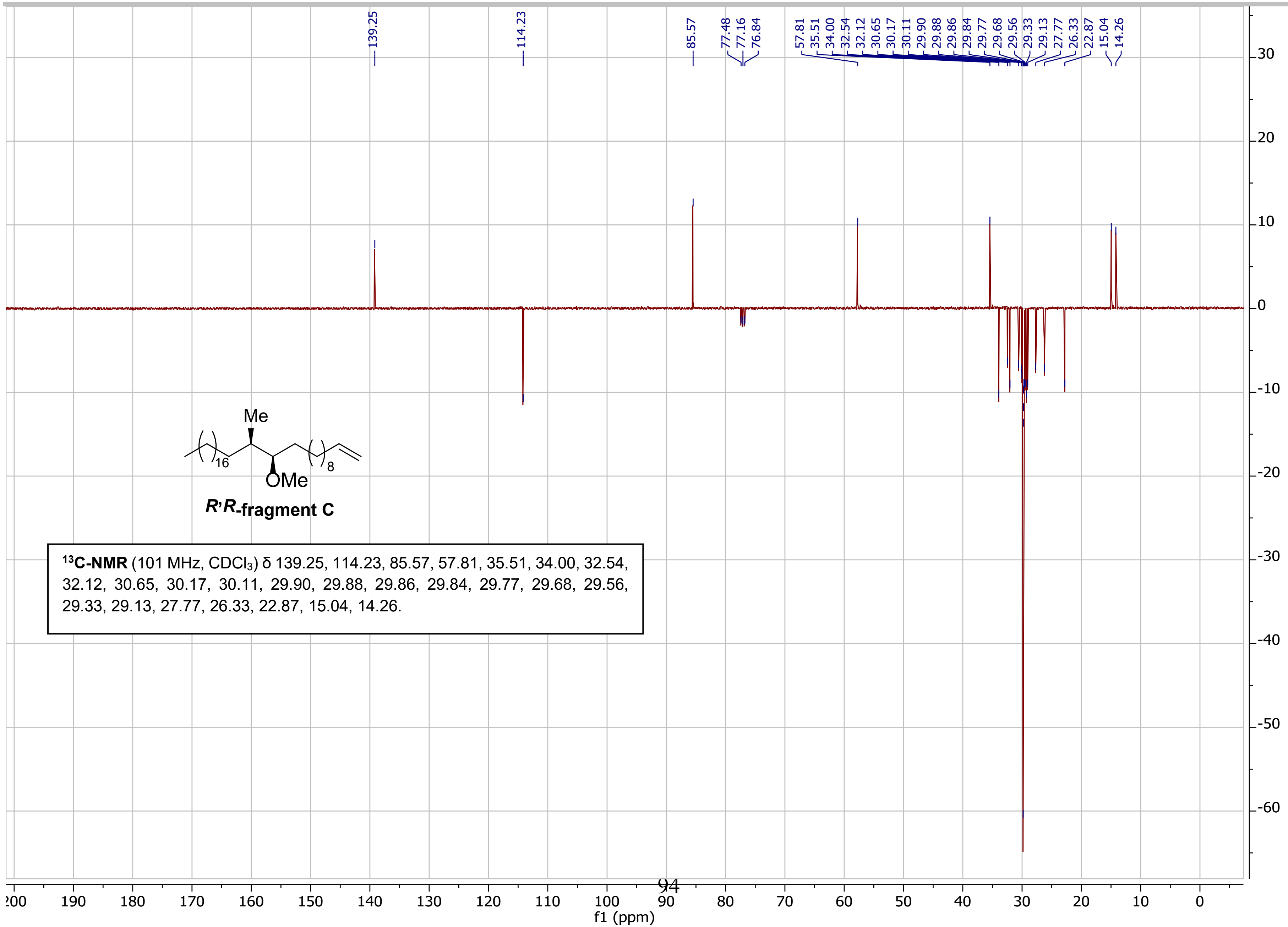


***R*,*R*-fragment C**

**$^{13}$C-NMR** (101 MHz, $CDCl_3$) δ 139.25, 114.23, 85.57, 57.81, 35.51, 34.00, 32.54, 32.12, 30.65, 30.17, 30.11, 29.90, 29.88, 29.86, 29.84, 29.77, 29.68, 29.56, 29.33, 29.13, 27.77, 26.33, 22.87, 15.04, 14.26.

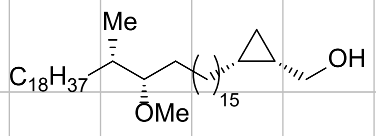


**28a**

**$^{1}$H-NMR** (400 MHz, $CDCl_3$) δ 3.65 (dd, $J$ = 11.3, 7.1 Hz, 1H), 3.58 (dd, $J$ = 11.3, 8.1 Hz, 1H), 3.34 (s, 3H), 3.00 – 2.91 (m, 1H), 1.67 – 1.58 (m, 1H), 1.57 – 1.03 (m, 67H), 0.94 – 0.79 (m, 8H), 0.70 (td, $J$ = 8.3, 4.4 Hz, 1H), -0.04 (q, $J$ = 5.3 Hz, 1H).

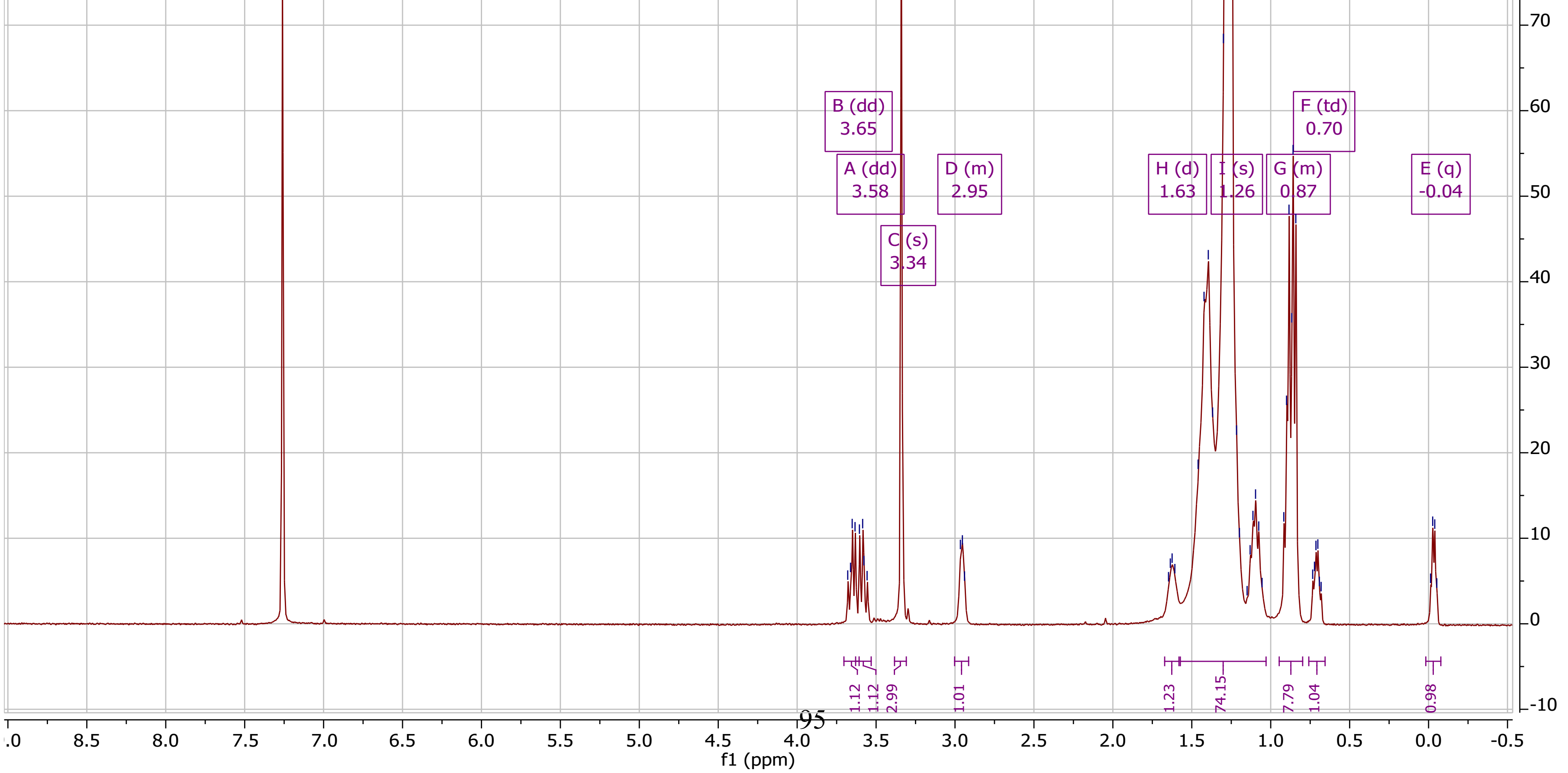

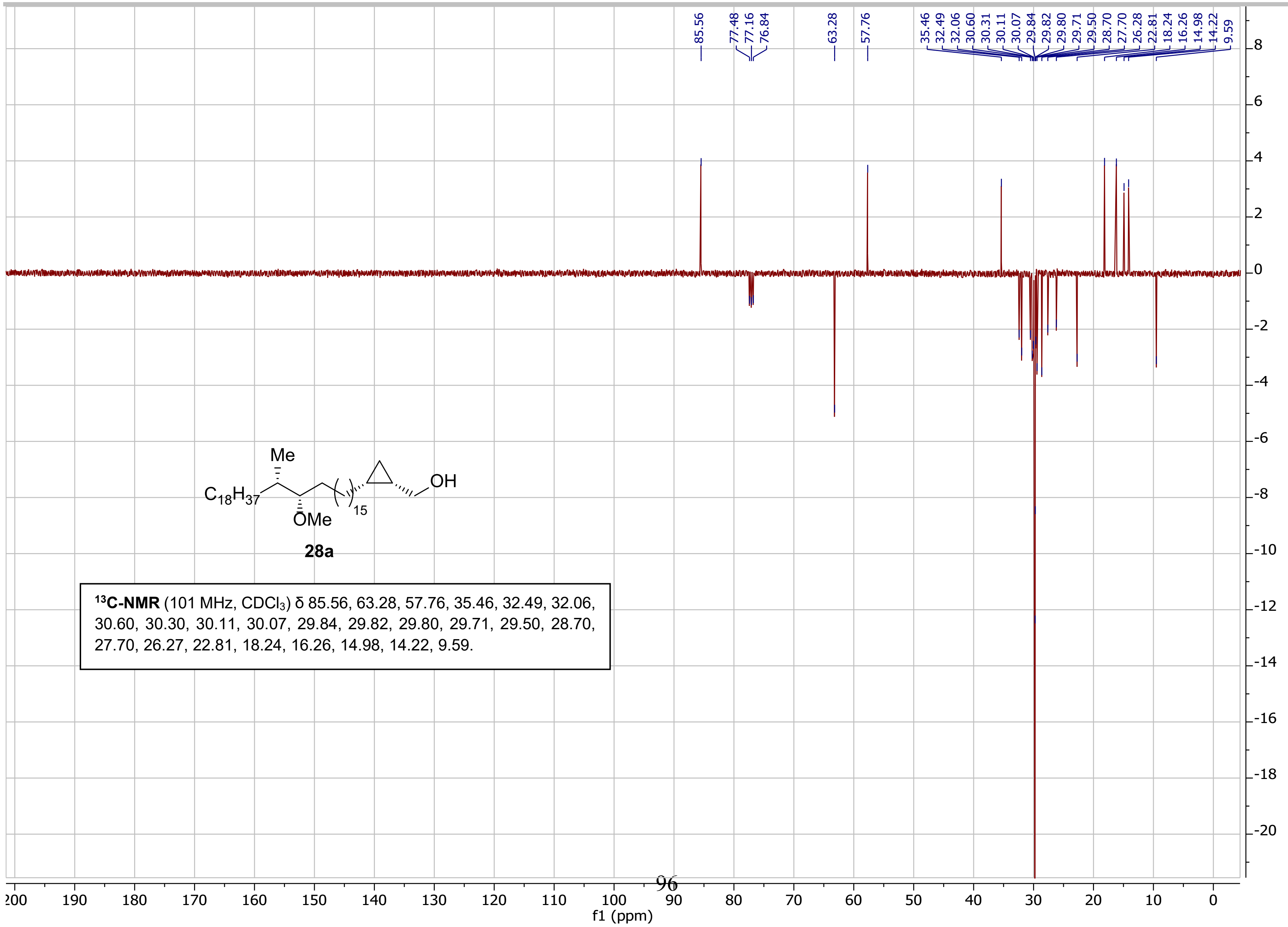


**$^{13}$C-NMR** (101 MHz, $CDCl_3$) δ 85.56, 63.28, 57.76, 35.46, 32.49, 32.06, 30.60, 30.30, 30.11, 30.07, 29.84, 29.82, 29.80, 29.71, 29.50, 28.70, 27.70, 26.27, 22.81, 18.24, 16.26, 14.98, 14.22, 9.59.

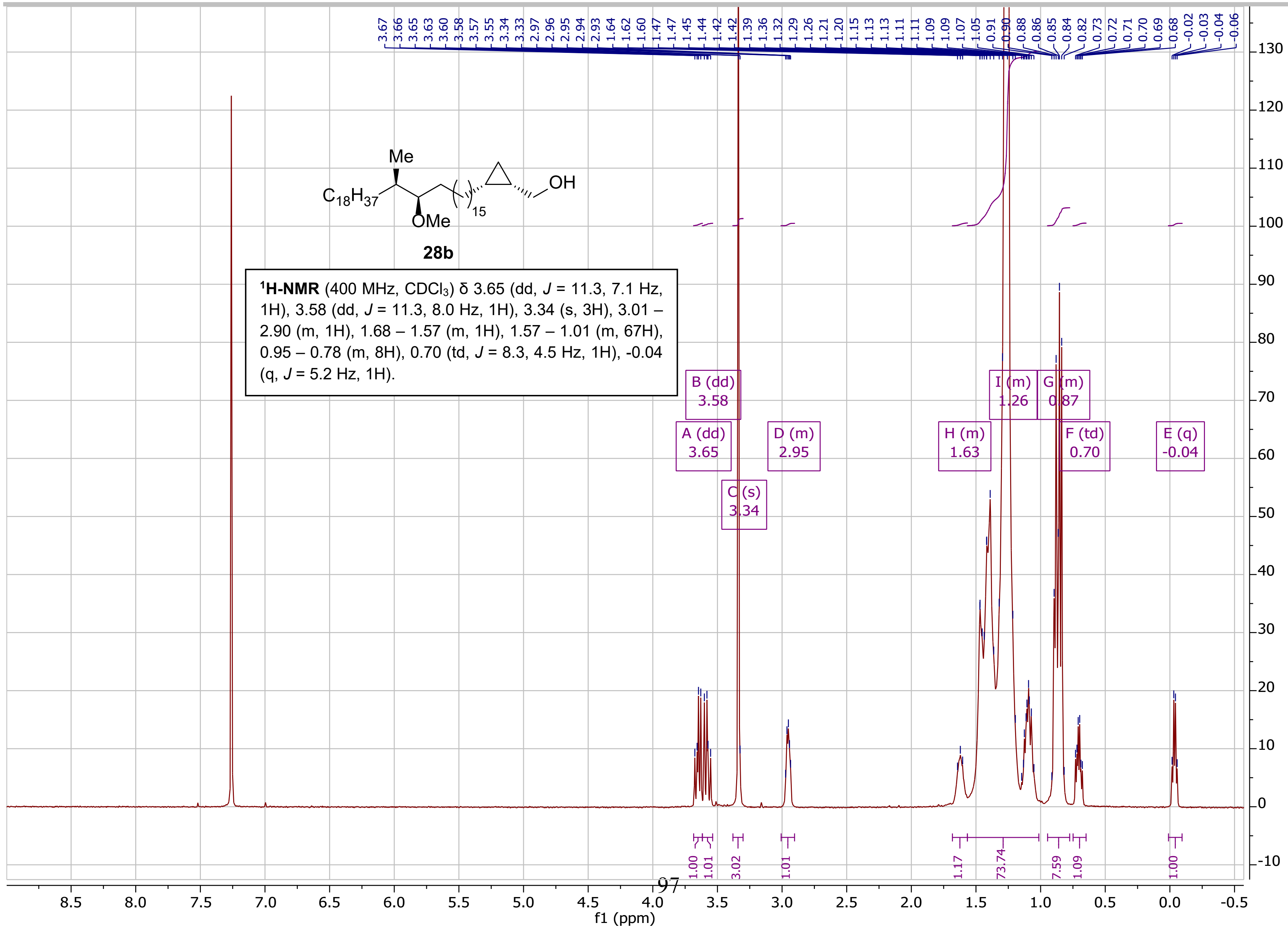


[1]**H-NMR** (400 MHz, $CDCl_3$) δ 3.65 (dd, *J* = 11.3, 7.1 Hz, 1H), 3.58 (dd, *J* = 11.3, 8.0 Hz, 1H), 3.34 (s, 3H), 3.01 – 2.90 (m, 1H), 1.68 – 1.57 (m, 1H), 1.57 – 1.01 (m, 67H), 0.95 – 0.78 (m, 8H), 0.70 (td, *J* = 8.3, 4.5 Hz, 1H), -0.04 (q, *J* = 5.2 Hz, 1H).

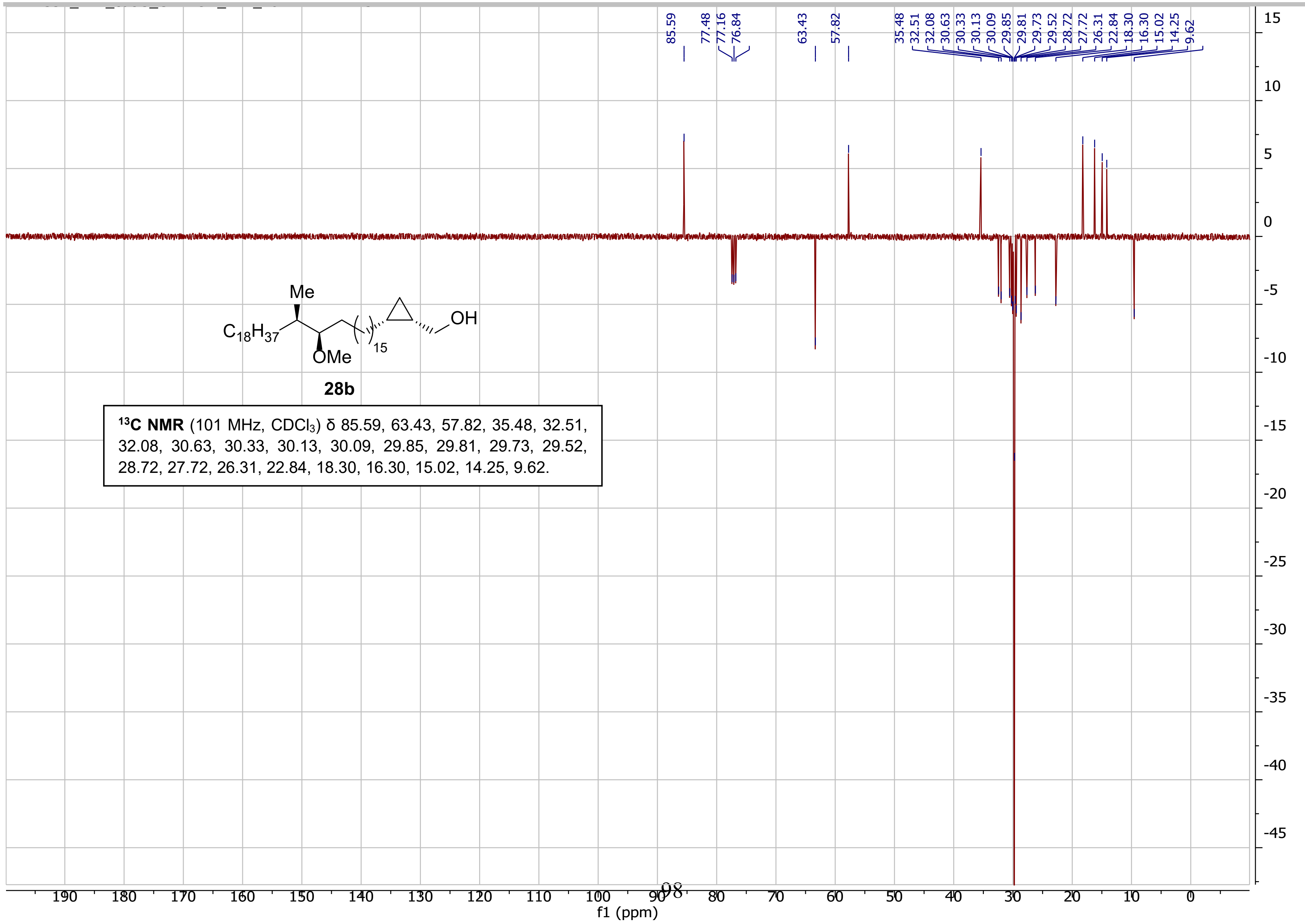


**28b**

**$^{13}$C NMR** (101 MHz, $CDCl_3$) δ 85.59, 63.43, 57.82, 35.48, 32.51, 32.08, 30.63, 30.33, 30.13, 30.09, 29.85, 29.81, 29.73, 29.52, 28.72, 27.72, 26.31, 22.84, 18.30, 16.30, 15.02, 14.25, 9.62.

**$^{1}$H-NMR** (400 MHz, $CDCl_3$) δ 3.69 – 3.61 (m, 1H), 3.61 – 3.54 (m, 1H), 3.34 (s, 3H), 3.00 – 2.91 (m, 1H), 1.68 – 1.56 (m, 1H), 1.50 – 1.01 (m, 67H), 0.94 – 0.79 (m, 8H), 0.70 (td, $J$ = 8.3, 4.5 Hz, 1H), -0.04 (q, $J$ = 5.3 Hz, 1H).

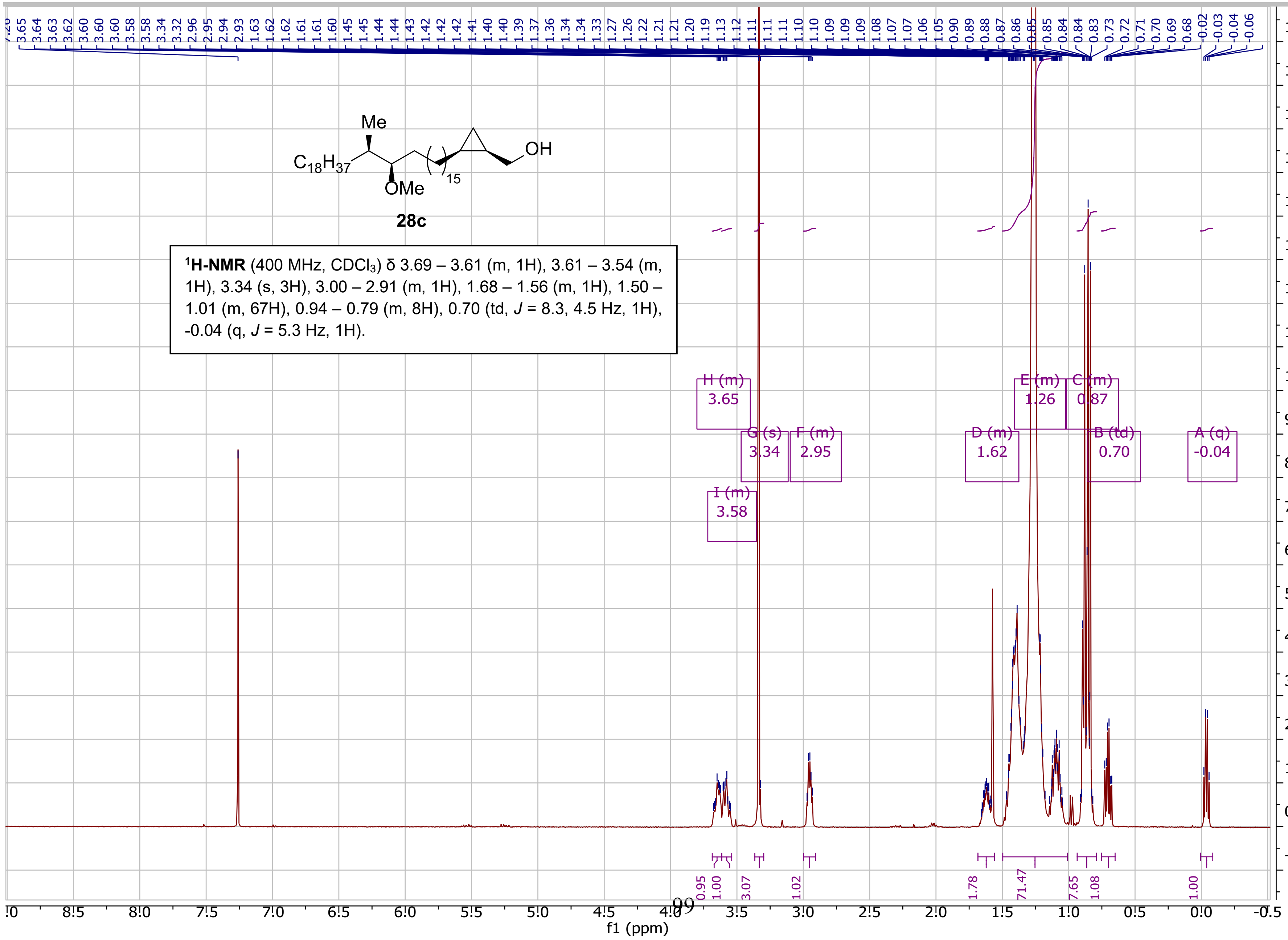

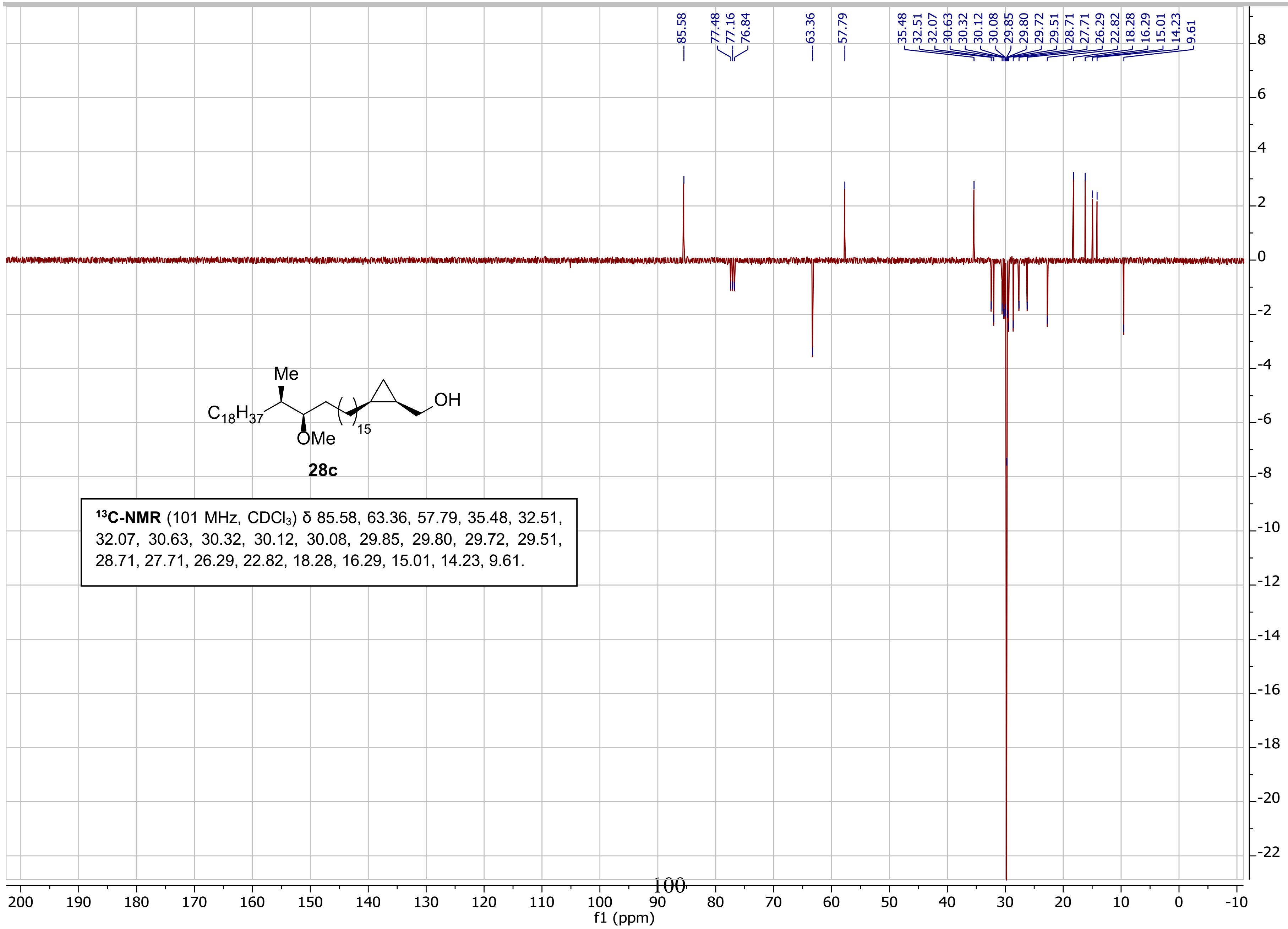

28c

$^{13}$**C-NMR** (101 MHz, $CDCl_3$) δ 85.58, 63.36, 57.79, 35.48, 32.51, 32.07, 30.63, 30.32, 30.12, 30.08, 29.85, 29.80, 29.72, 29.51, 28.71, 27.71, 26.29, 22.82, 18.28, 16.29, 15.01, 14.23, 9.61.

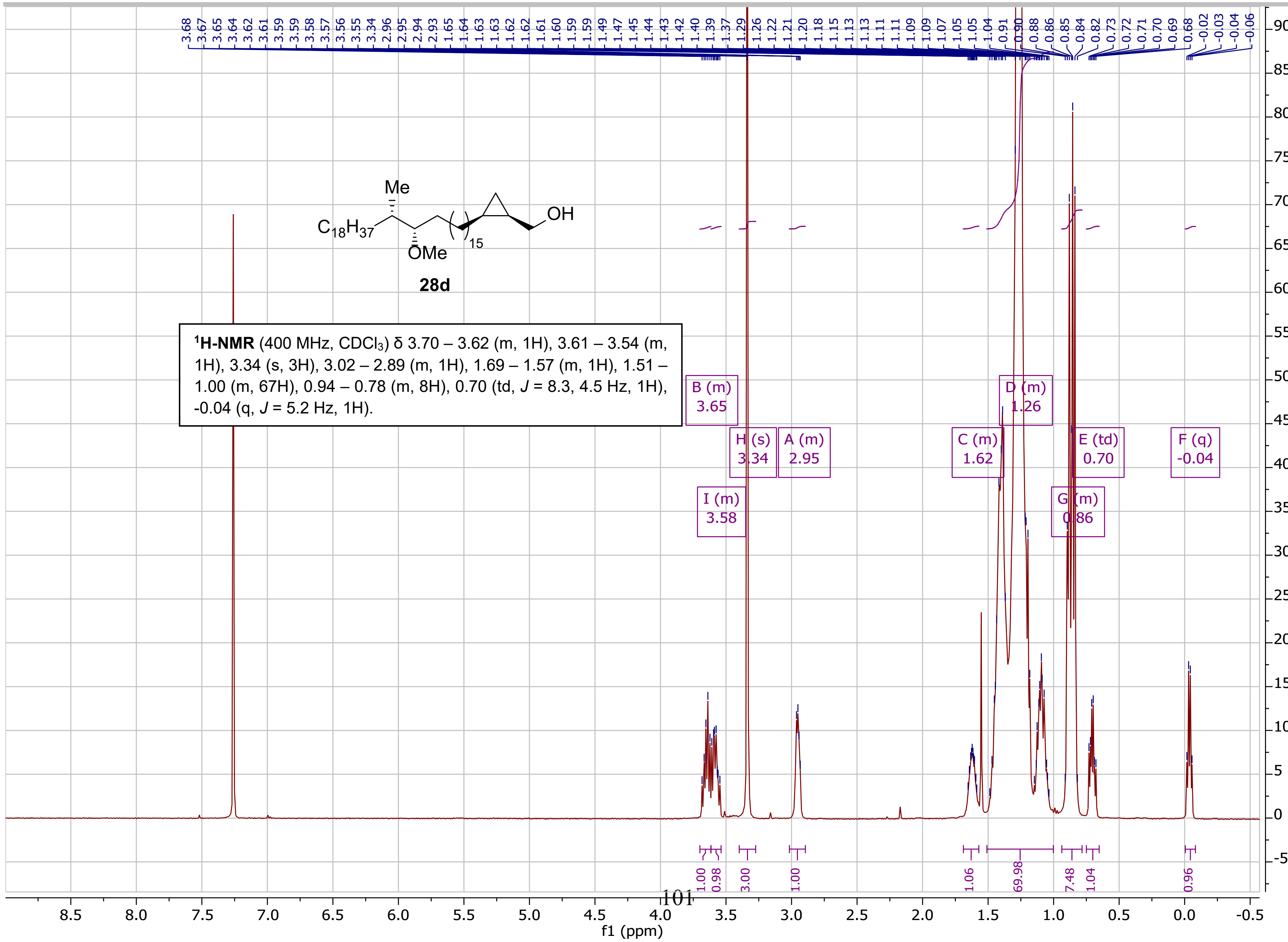


[1]**H-NMR** (400 MHz, $CDCl_3$) δ 3.70 – 3.62 (m, 1H), 3.61 – 3.54 (m, 1H), 3.34 (s, 3H), 3.02 – 2.89 (m, 1H), 1.69 – 1.57 (m, 1H), 1.51 – 1.00 (m, 67H), 0.94 – 0.78 (m, 8H), 0.70 (td, *J* = 8.3, 4.5 Hz, 1H), -0.04 (q, *J* = 5.2 Hz, 1H).

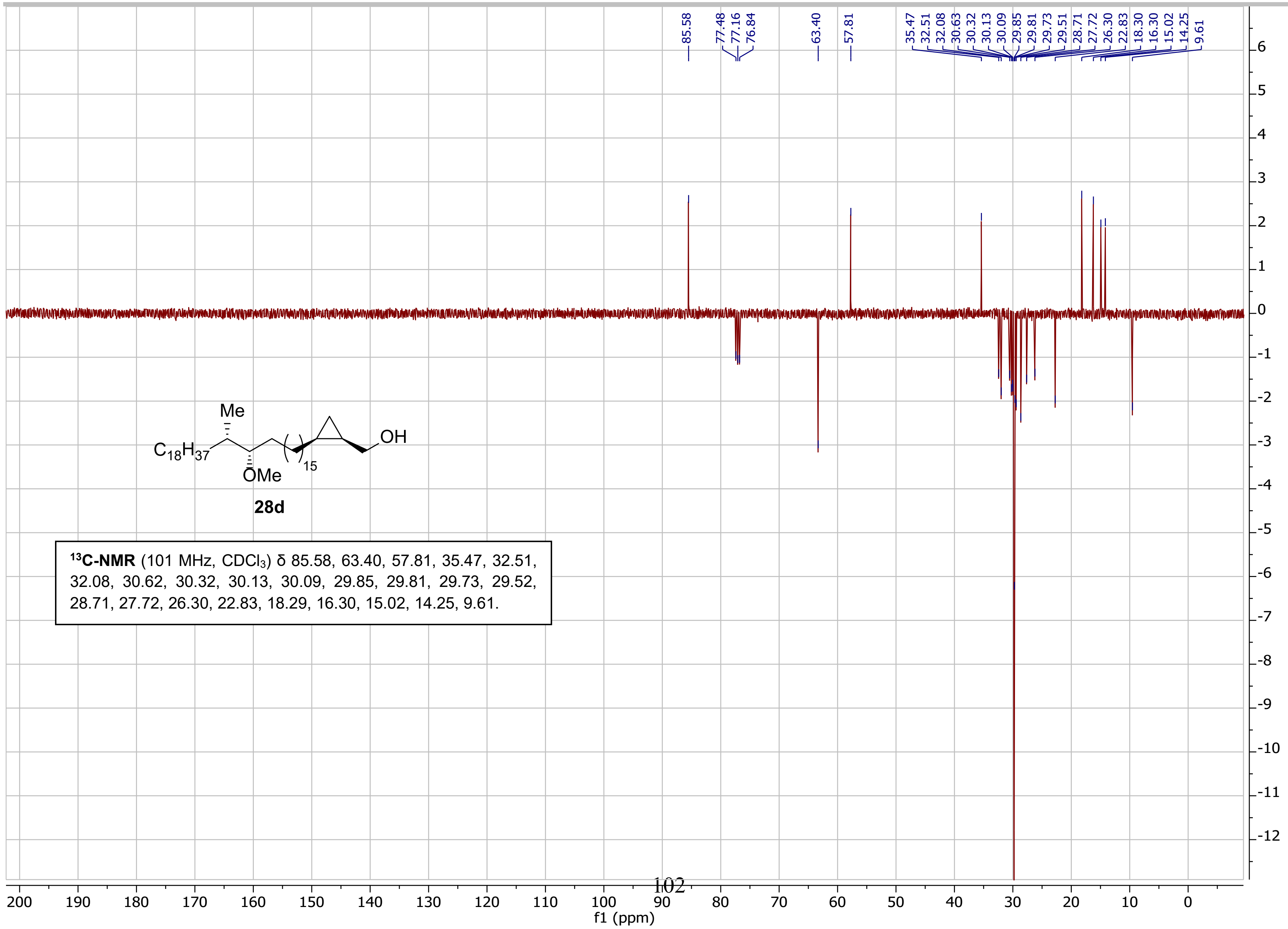


**28d**

**$^{13}$C-NMR** (101 MHz, $CDCl_3$) δ 85.58, 63.40, 57.81, 35.47, 32.51, 32.08, 30.62, 30.32, 30.13, 30.09, 29.85, 29.81, 29.73, 29.52, 28.71, 27.72, 26.30, 22.83, 18.29, 16.30, 15.02, 14.25, 9.61.

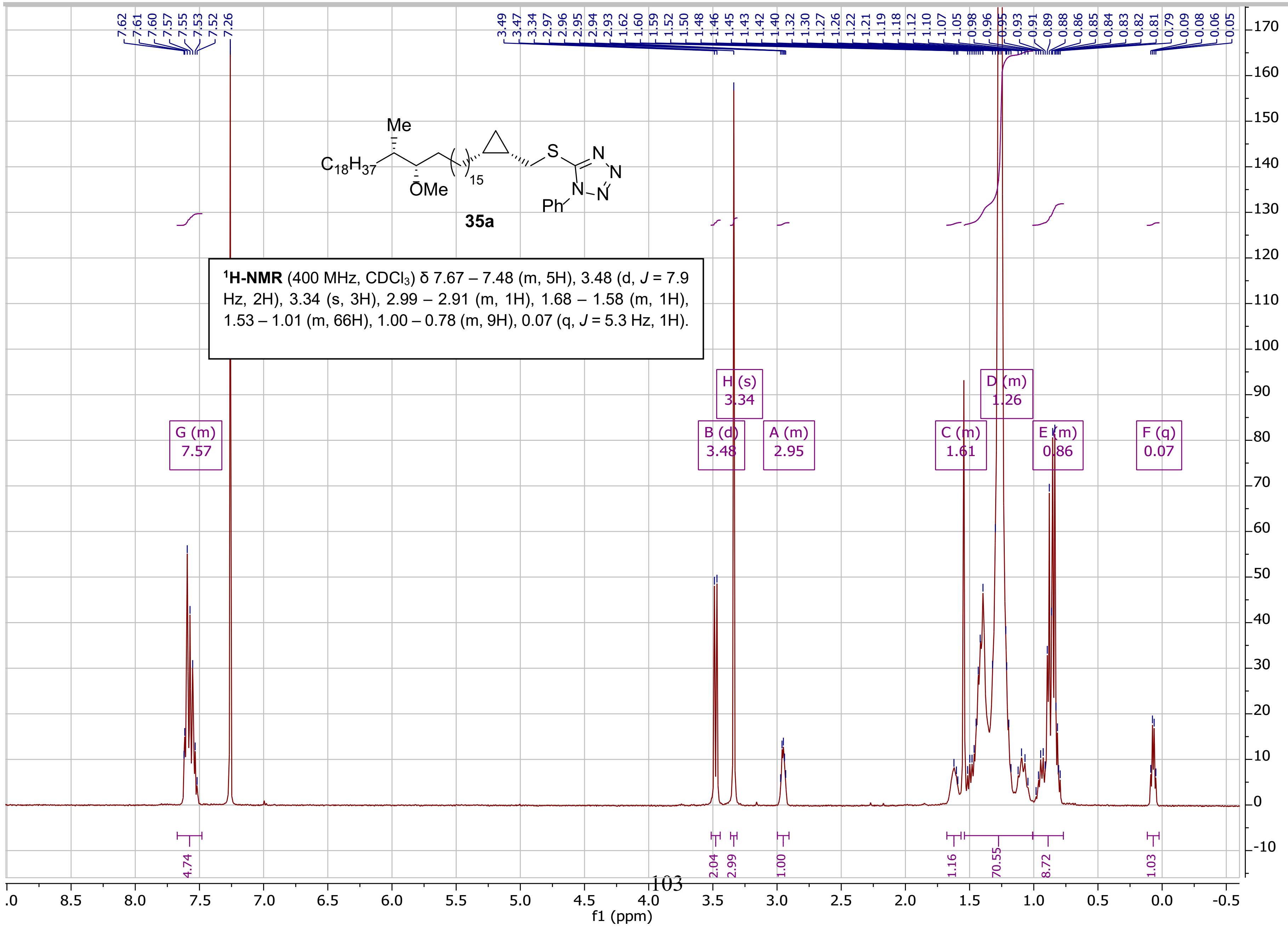


**¹H-NMR** (400 MHz, $CDCl_3$) δ 7.67 – 7.48 (m, 5H), 3.48 (d, *J* = 7.9 Hz, 2H), 3.34 (s, 3H), 2.99 – 2.91 (m, 1H), 1.68 – 1.58 (m, 1H), 1.53 – 1.01 (m, 66H), 1.00 – 0.78 (m, 9H), 0.07 (q, *J* = 5.3 Hz, 1H).

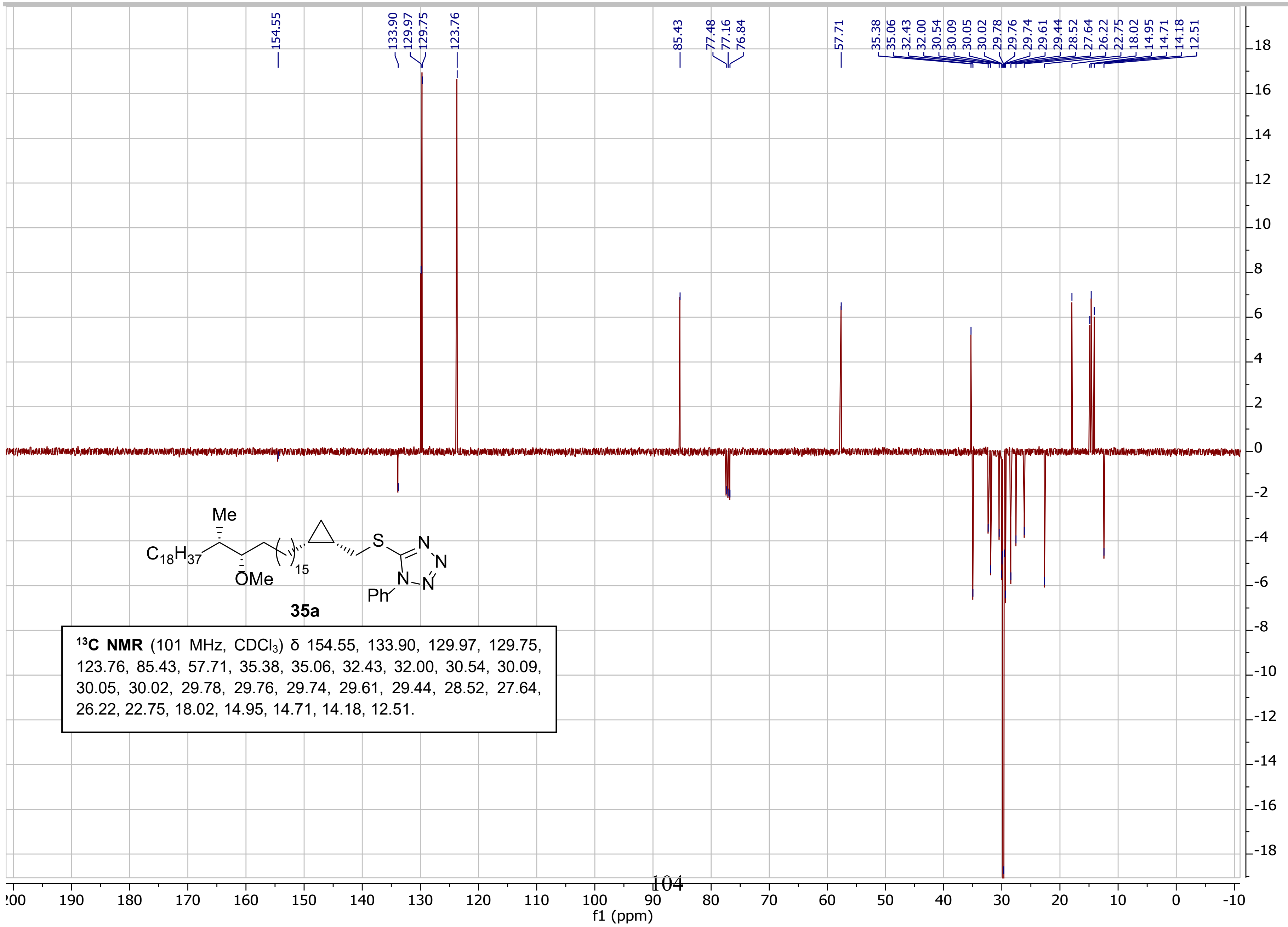


**35a**

**$^{13}$C NMR** (101 MHz, $CDCl_3$) δ 154.55, 133.90, 129.97, 129.75, 123.76, 85.43, 57.71, 35.38, 35.06, 32.43, 32.00, 30.54, 30.09, 30.05, 30.02, 29.78, 29.76, 29.74, 29.61, 29.44, 28.52, 27.64, 26.22, 22.75, 18.02, 14.95, 14.71, 14.18, 12.51.

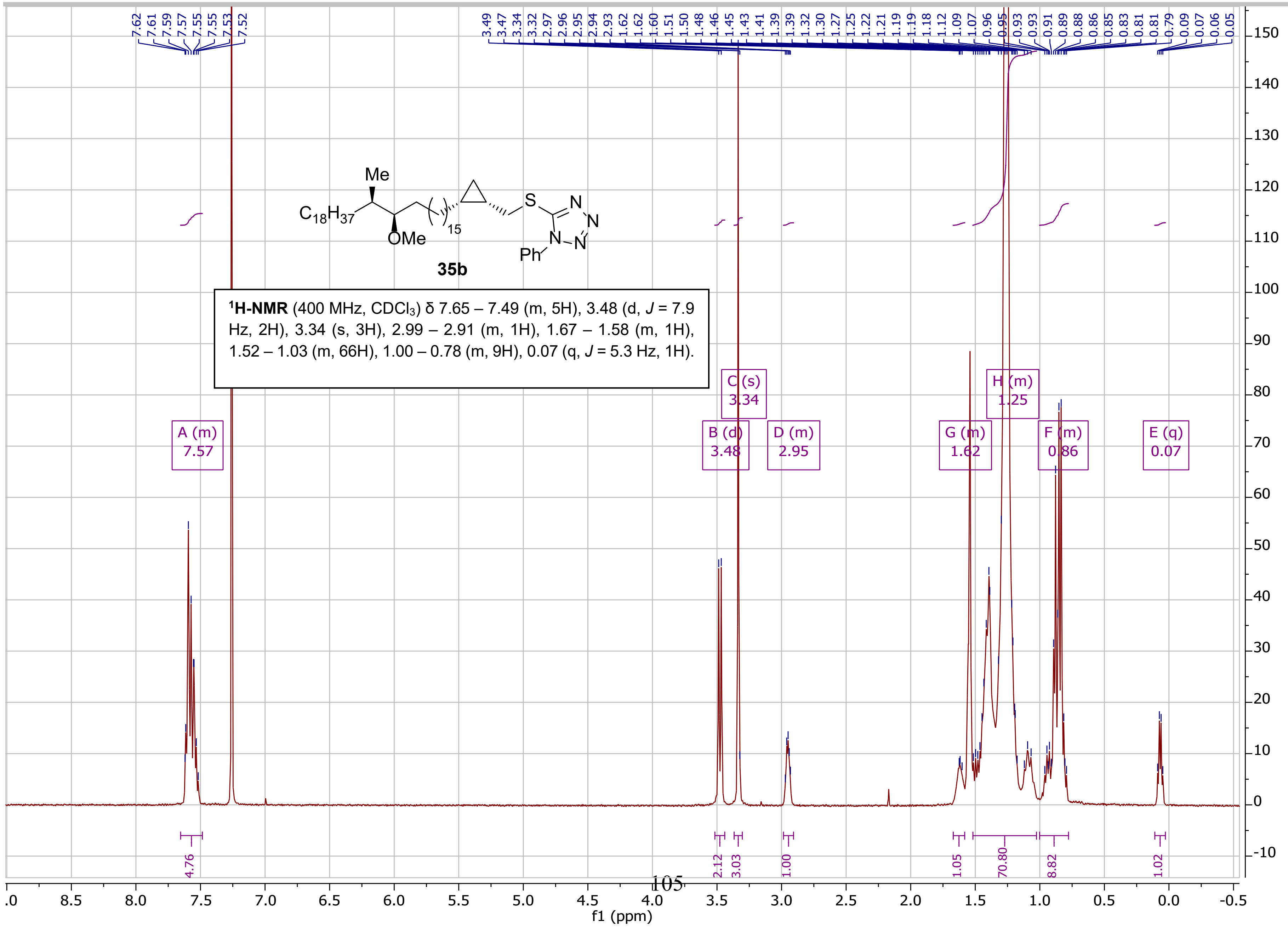


**$^1$H-NMR** (400 MHz, $CDCl_3$) δ 7.65 – 7.49 (m, 5H), 3.48 (d, *J* = 7.9 Hz, 2H), 3.34 (s, 3H), 2.99 – 2.91 (m, 1H), 1.67 – 1.58 (m, 1H), 1.52 – 1.03 (m, 66H), 1.00 – 0.78 (m, 9H), 0.07 (q, *J* = 5.3 Hz, 1H).

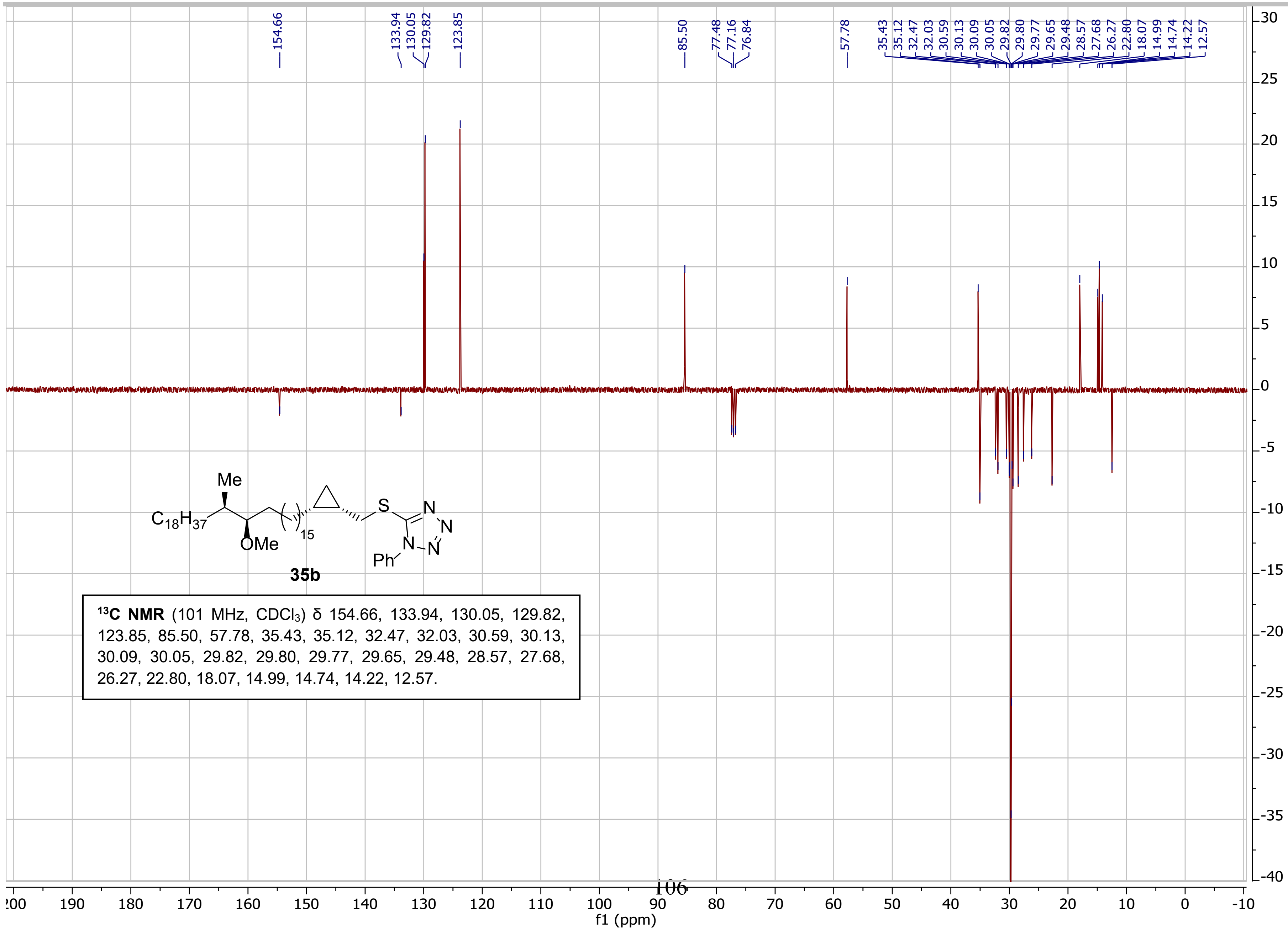

**$^{13}$C NMR** (101 MHz, $CDCl_3$) δ 154.66, 133.94, 130.05, 129.82, 123.85, 85.50, 57.78, 35.43, 35.12, 32.47, 32.03, 30.59, 30.13, 30.09, 30.05, 29.82, 29.80, 29.77, 29.65, 29.48, 28.57, 27.68, 26.27, 22.80, 18.07, 14.99, 14.74, 14.22, 12.57.

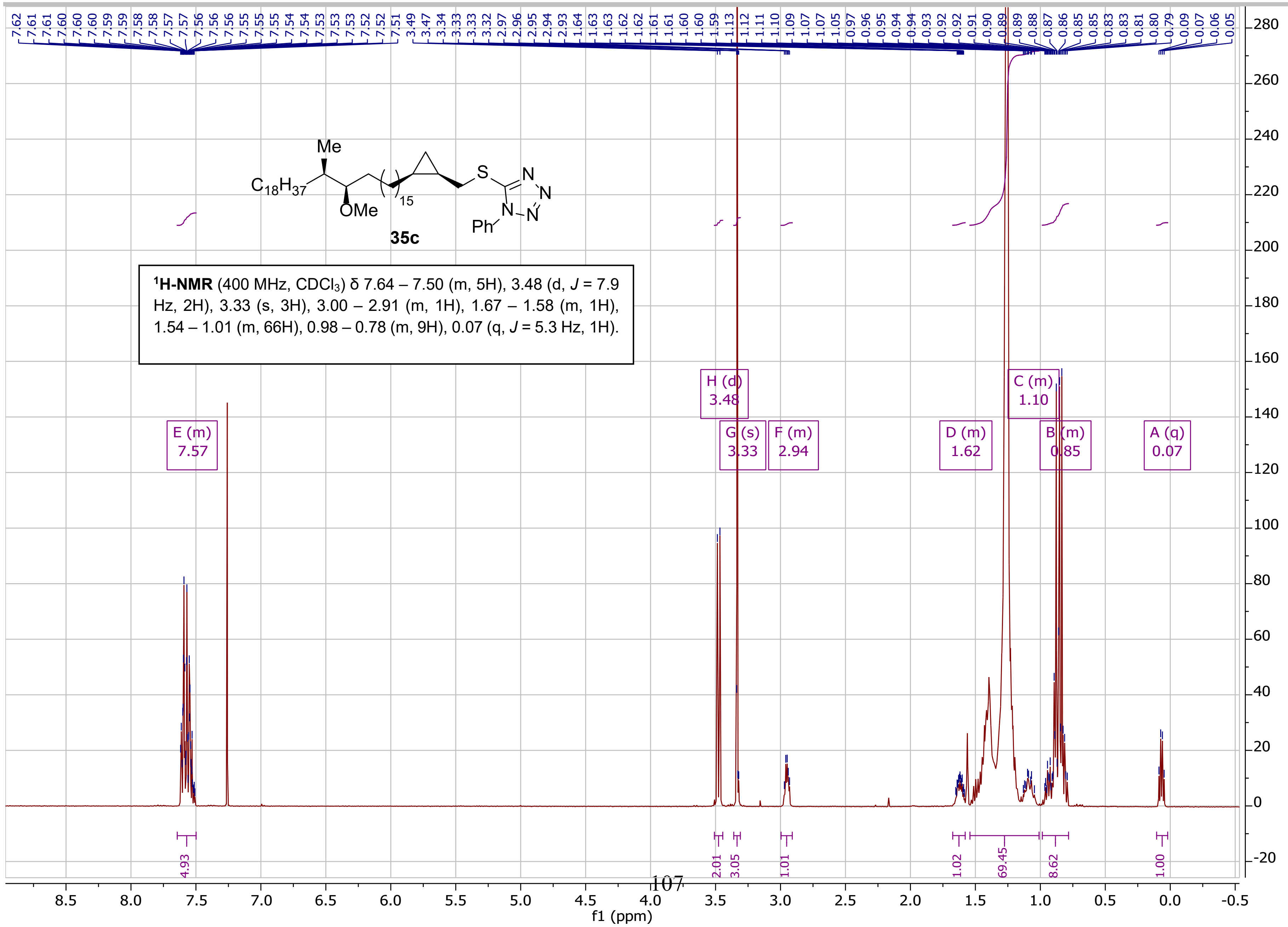


**$^1$H-NMR** (400 MHz, $CDCl_3$) δ 7.64 – 7.50 (m, 5H), 3.48 (d, *J* = 7.9 Hz, 2H), 3.33 (s, 3H), 3.00 – 2.91 (m, 1H), 1.67 – 1.58 (m, 1H), 1.54 – 1.01 (m, 66H), 0.98 – 0.78 (m, 9H), 0.07 (q, *J* = 5.3 Hz, 1H).

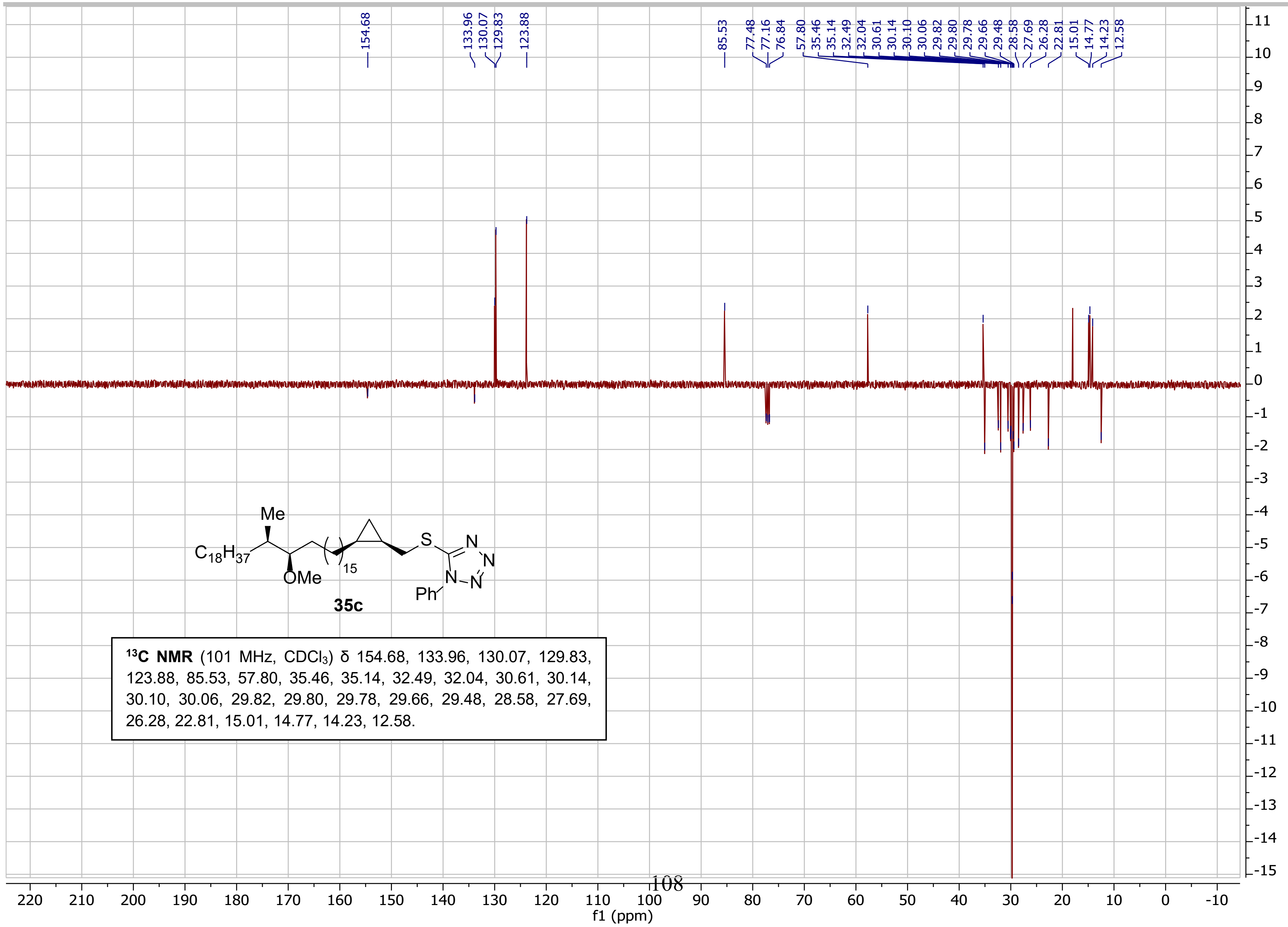


**$^{13}$C NMR** (101 MHz, $CDCl_3$) δ 154.68, 133.96, 130.07, 129.83, 123.88, 85.53, 57.80, 35.46, 35.14, 32.49, 32.04, 30.61, 30.14, 30.10, 30.06, 29.82, 29.80, 29.78, 29.66, 29.48, 28.58, 27.69, 26.28, 22.81, 15.01, 14.77, 14.23, 12.58.

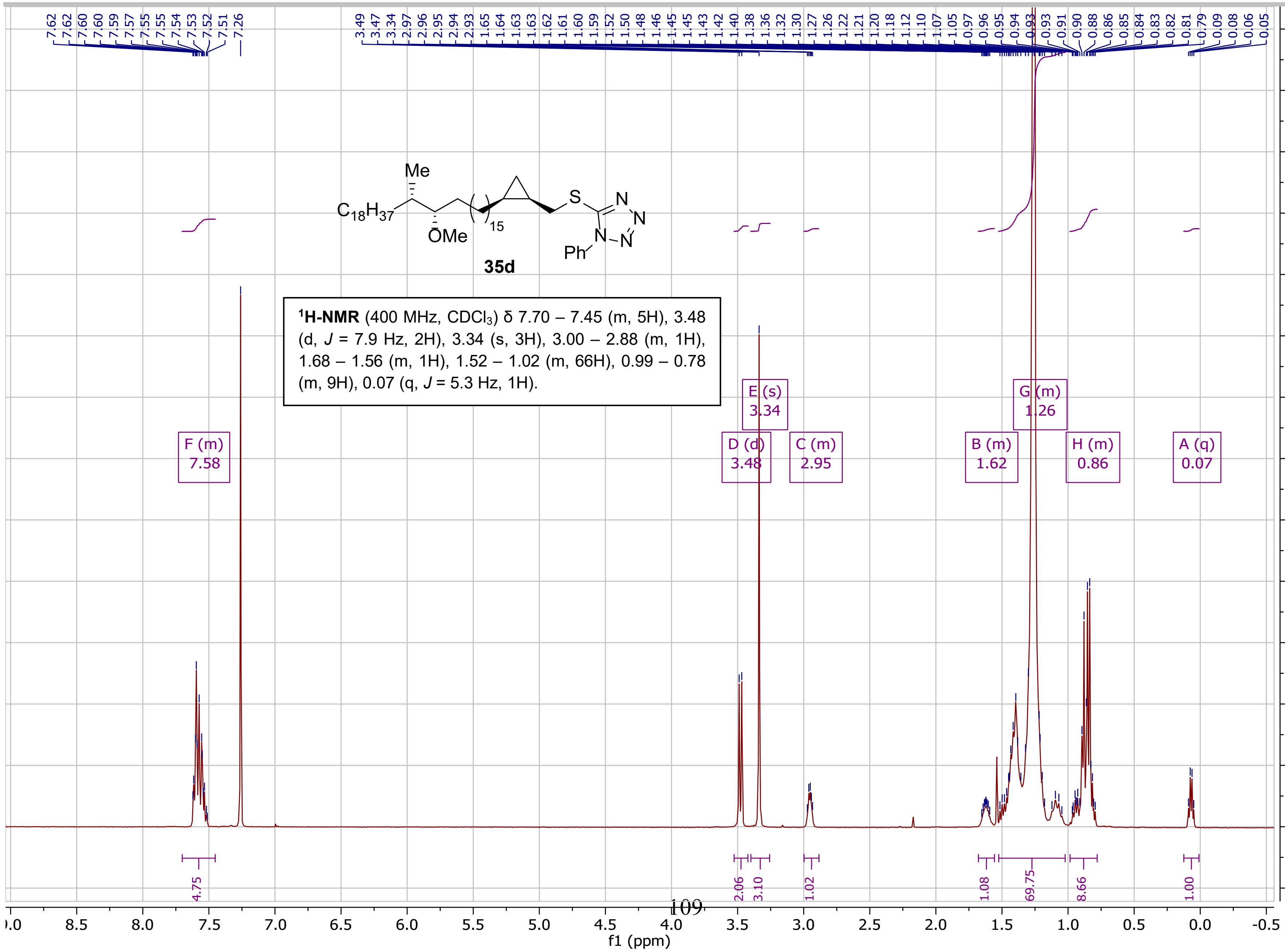
Me
$C_{18}H_{37}$
OMe
15
S
N
Ph
35d
$^{1}$H-NMR (400 MHz, $CDCl_3$) δ 7.70 – 7.45 (m, 5H), 3.48 (d, J = 7.9 Hz, 2H), 3.34 (s, 3H), 3.00 – 2.88 (m, 1H), 1.68 – 1.56 (m, 1H), 1.52 – 1.02 (m, 66H), 0.99 – 0.78 (m, 9H), 0.07 (q, J = 5.3 Hz, 1H).
F (m) 7.58
E (s) 3.34
D (d) 3.48
C (m) 2.95
B (m) 1.62
G (m) 1.26
H (m) 0.86
A (q) 0.07
f1 (ppm)

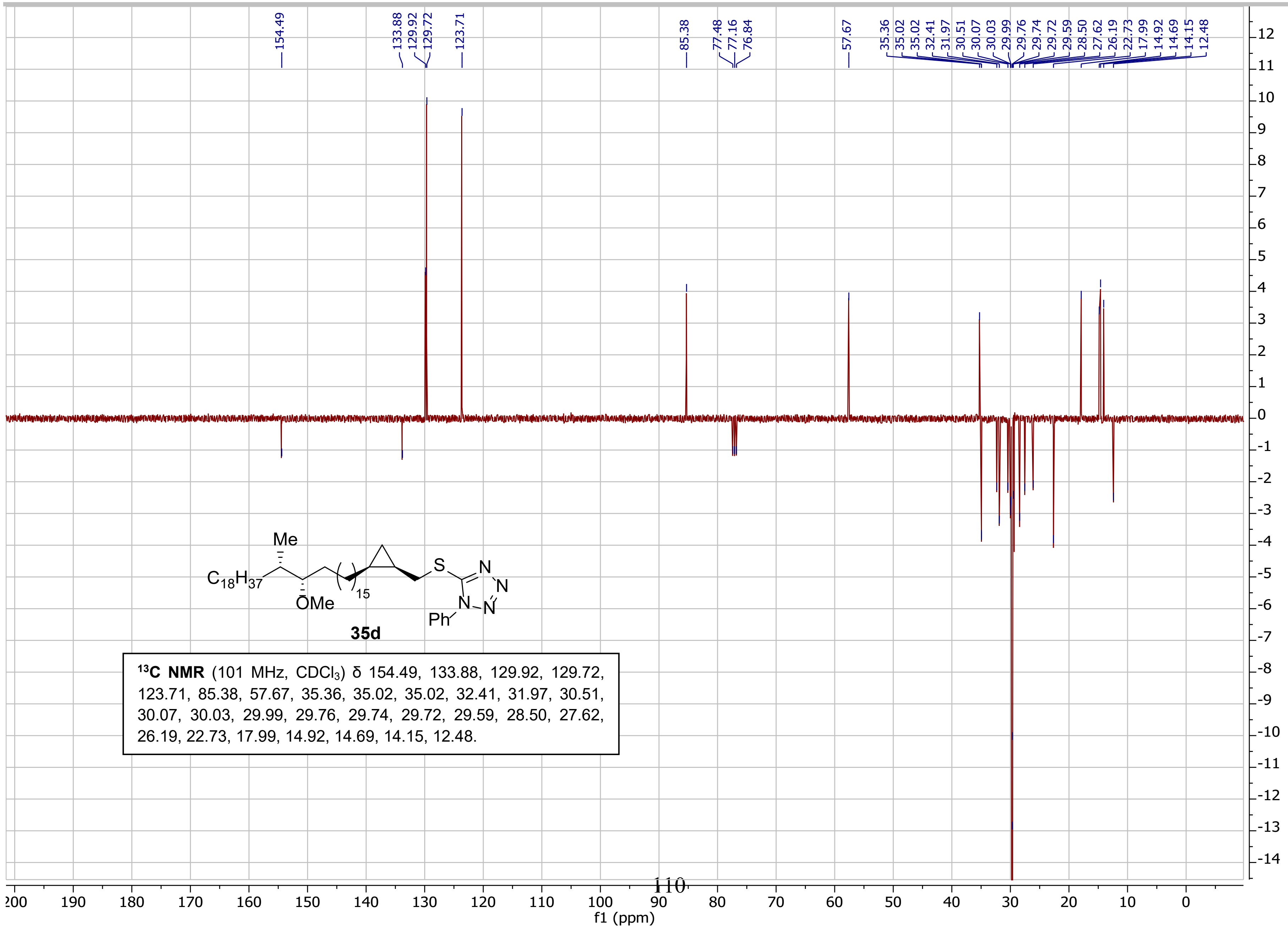

35d

**$^{13}$C NMR** (101 MHz, $CDCl_3$) δ 154.49, 133.88, 129.92, 129.72, 123.71, 85.38, 57.67, 35.36, 35.02, 35.02, 32.41, 31.97, 30.51, 30.07, 30.03, 29.99, 29.76, 29.74, 29.72, 29.59, 28.50, 27.62, 26.19, 22.73, 17.99, 14.92, 14.69, 14.15, 12.48.

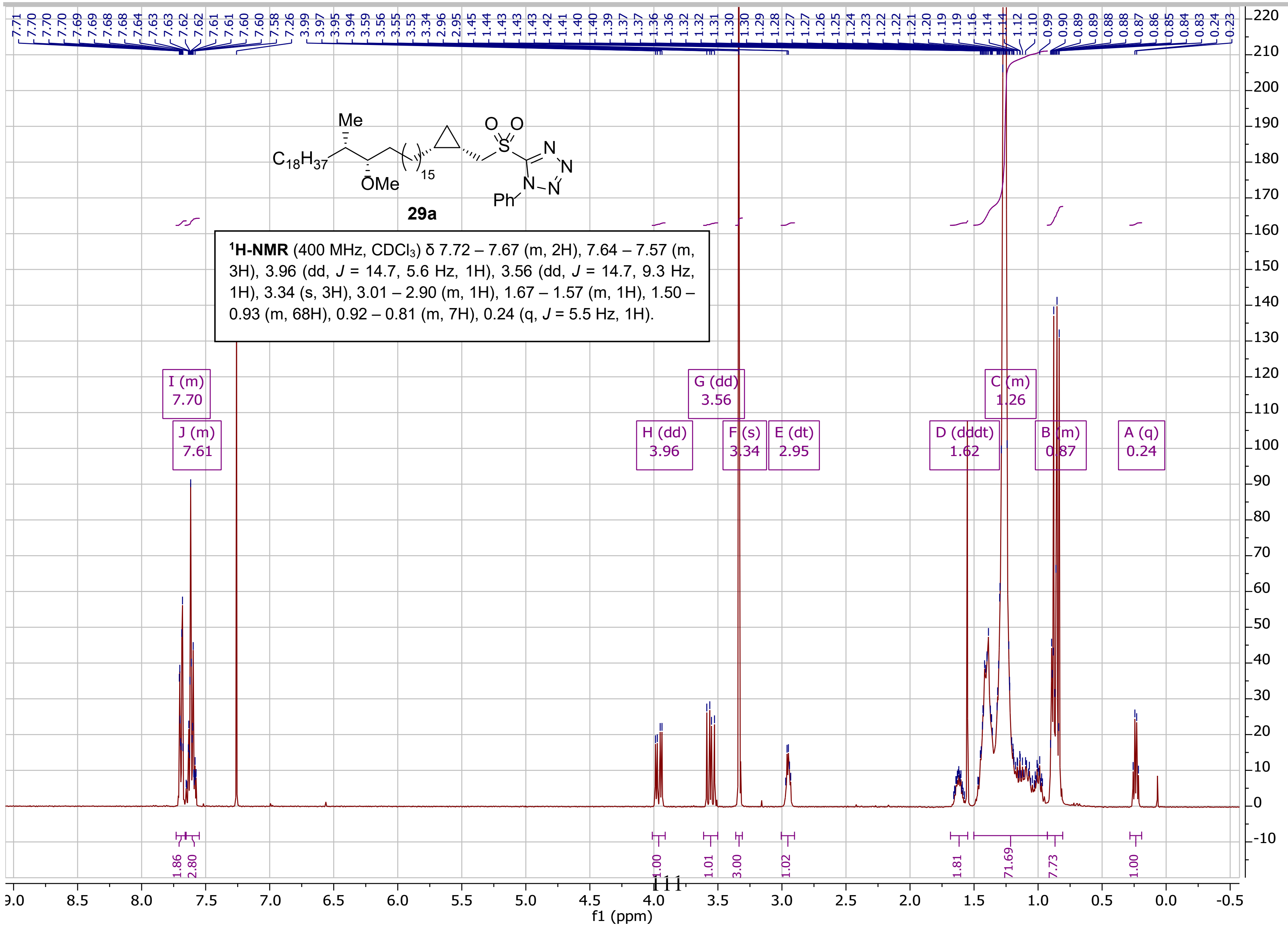

**29a**

**$^{1}$H-NMR** (400 MHz, $CDCl_3$) δ 7.72 – 7.67 (m, 2H), 7.64 – 7.57 (m, 3H), 3.96 (dd, *J* = 14.7, 5.6 Hz, 1H), 3.56 (dd, *J* = 14.7, 9.3 Hz, 1H), 3.34 (s, 3H), 3.01 – 2.90 (m, 1H), 1.67 – 1.57 (m, 1H), 1.50 – 0.93 (m, 68H), 0.92 – 0.81 (m, 7H), 0.24 (q, *J* = 5.5 Hz, 1H).

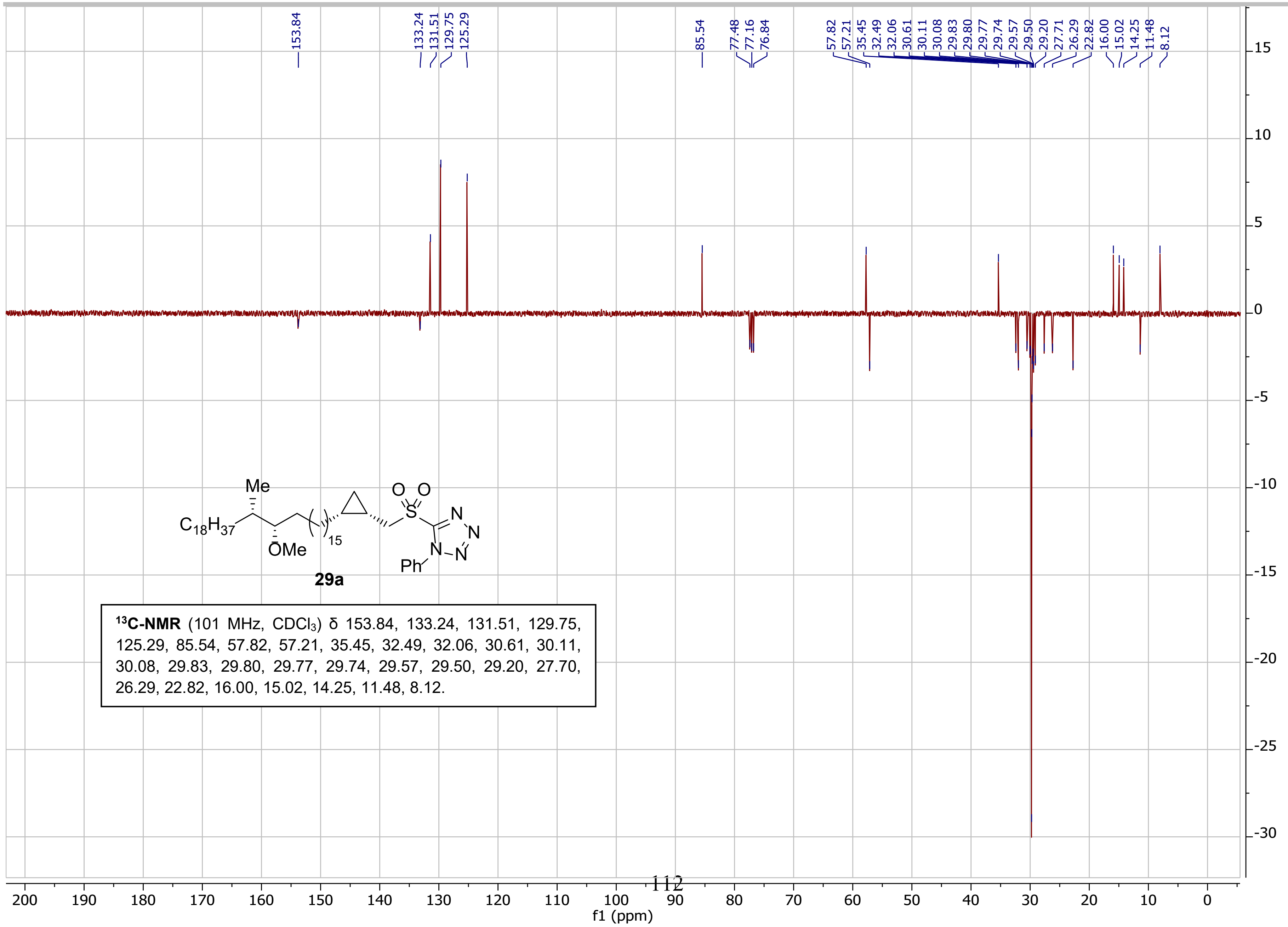


**29a**

**$^{13}$C-NMR** (101 MHz, $CDCl_3$) δ 153.84, 133.24, 131.51, 129.75, 125.29, 85.54, 57.82, 57.21, 35.45, 32.49, 32.06, 30.61, 30.11, 30.08, 29.83, 29.80, 29.77, 29.74, 29.57, 29.50, 29.20, 27.70, 26.29, 22.82, 16.00, 15.02, 14.25, 11.48, 8.12.

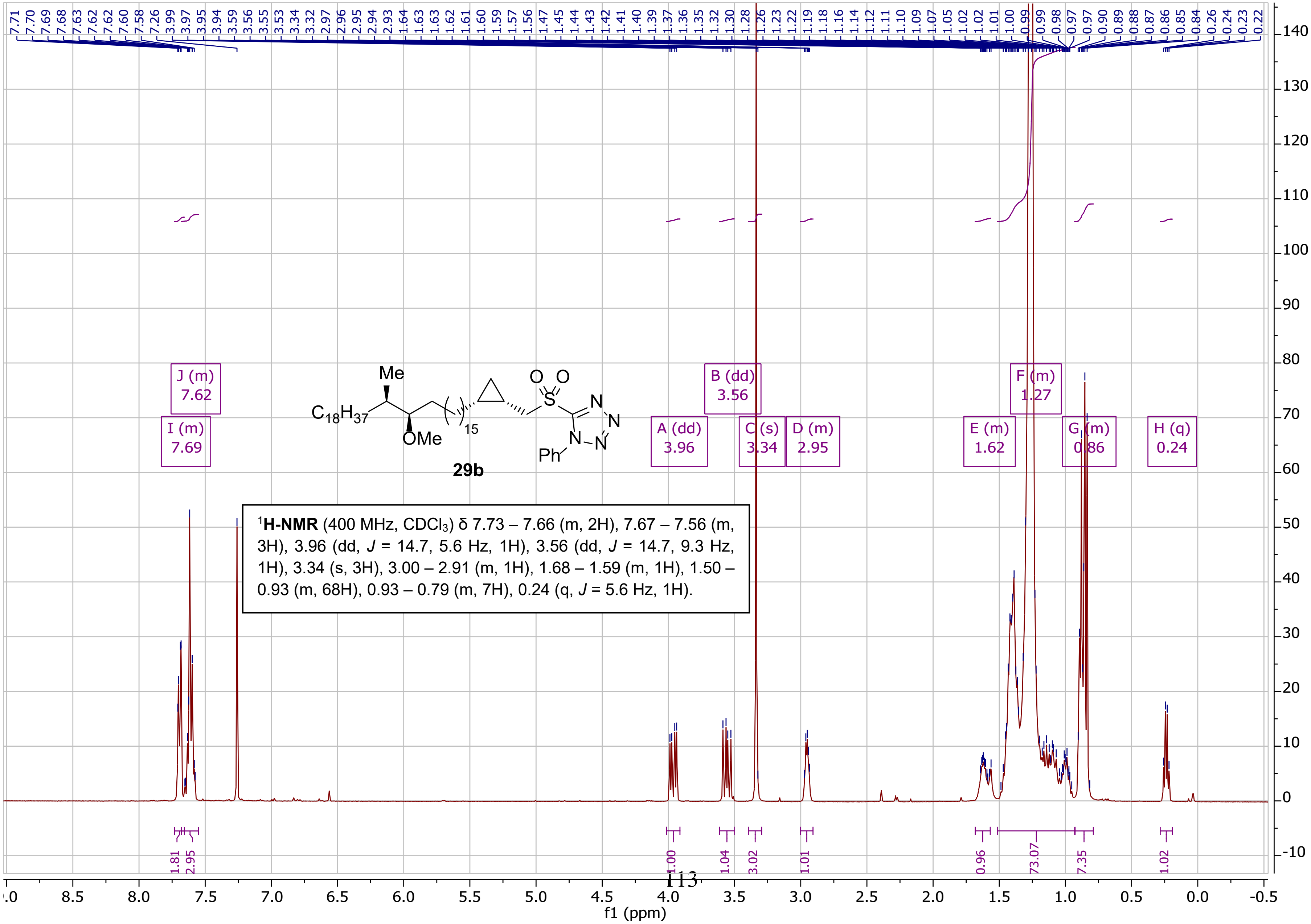


**$^1$H-NMR** (400 MHz, $CDCl_3$) δ 7.73 – 7.66 (m, 2H), 7.67 – 7.56 (m, 3H), 3.96 (dd, *J* = 14.7, 5.6 Hz, 1H), 3.56 (dd, *J* = 14.7, 9.3 Hz, 1H), 3.34 (s, 3H), 3.00 – 2.91 (m, 1H), 1.68 – 1.59 (m, 1H), 1.50 – 0.93 (m, 68H), 0.93 – 0.79 (m, 7H), 0.24 (q, *J* = 5.6 Hz, 1H).

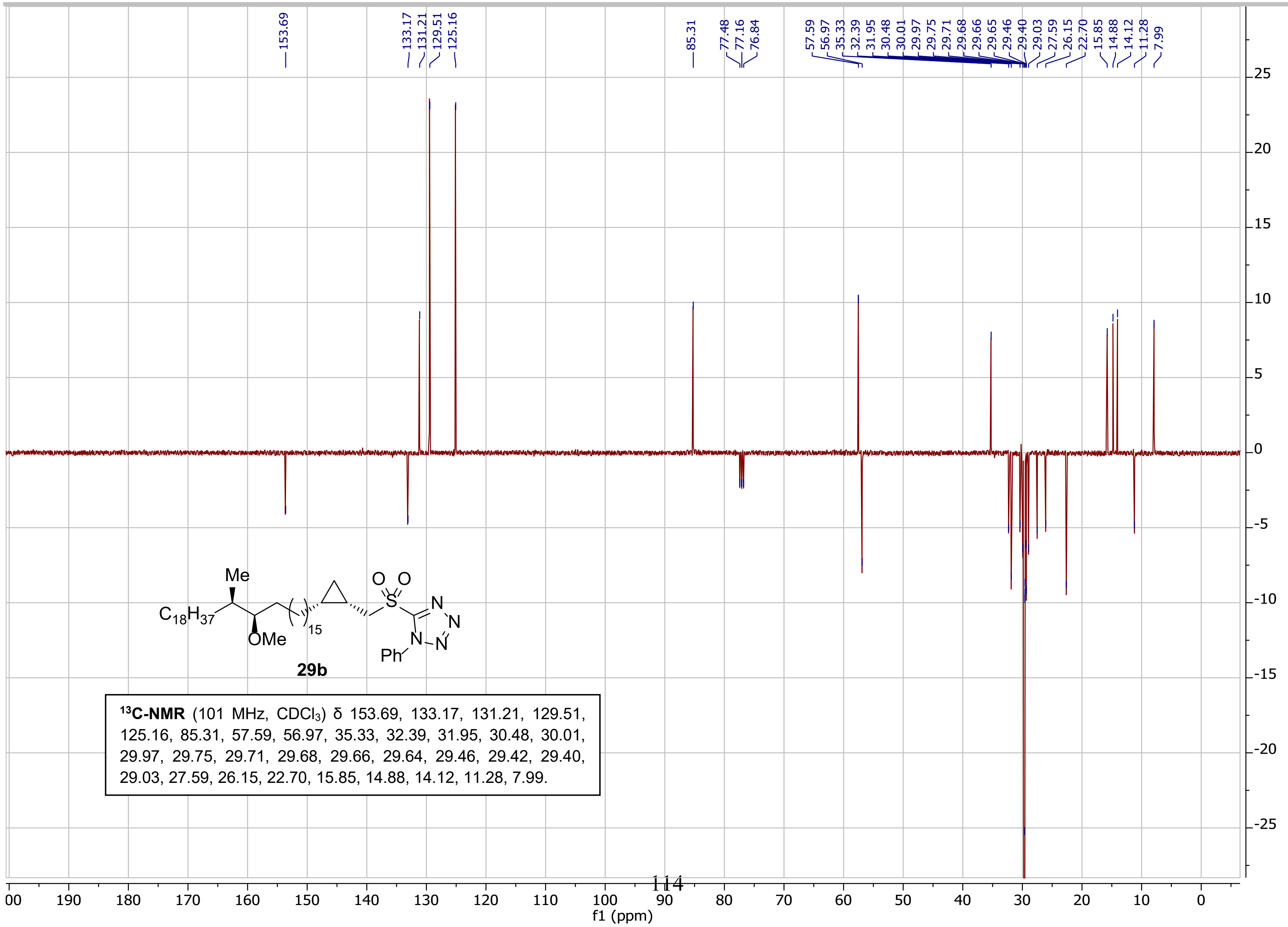


**$^{13}$C-NMR** (101 MHz, $CDCl_3$) δ 153.69, 133.17, 131.21, 129.51, 125.16, 85.31, 57.59, 56.97, 35.33, 32.39, 31.95, 30.48, 30.01, 29.97, 29.75, 29.71, 29.68, 29.66, 29.64, 29.46, 29.42, 29.40, 29.03, 27.59, 26.15, 22.70, 15.85, 14.88, 14.12, 11.28, 7.99.

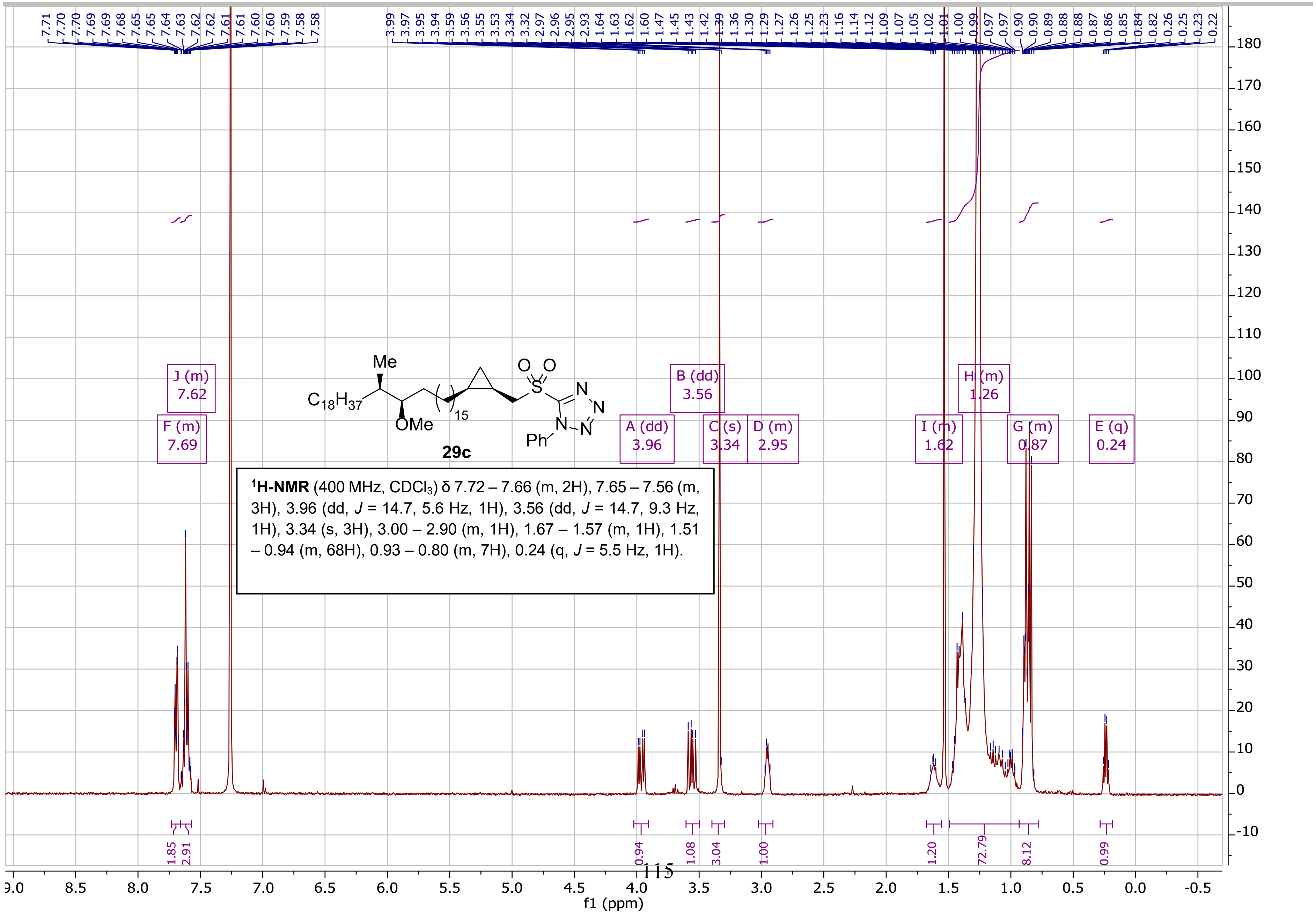

**29c**

**$^{1}$H-NMR** (400 MHz, $CDCl_3$) δ 7.72 – 7.66 (m, 2H), 7.65 – 7.56 (m, 3H), 3.96 (dd, *J* = 14.7, 5.6 Hz, 1H), 3.56 (dd, *J* = 14.7, 9.3 Hz, 1H), 3.34 (s, 3H), 3.00 – 2.90 (m, 1H), 1.67 – 1.57 (m, 1H), 1.51 – 0.94 (m, 68H), 0.93 – 0.80 (m, 7H), 0.24 (q, *J* = 5.5 Hz, 1H).

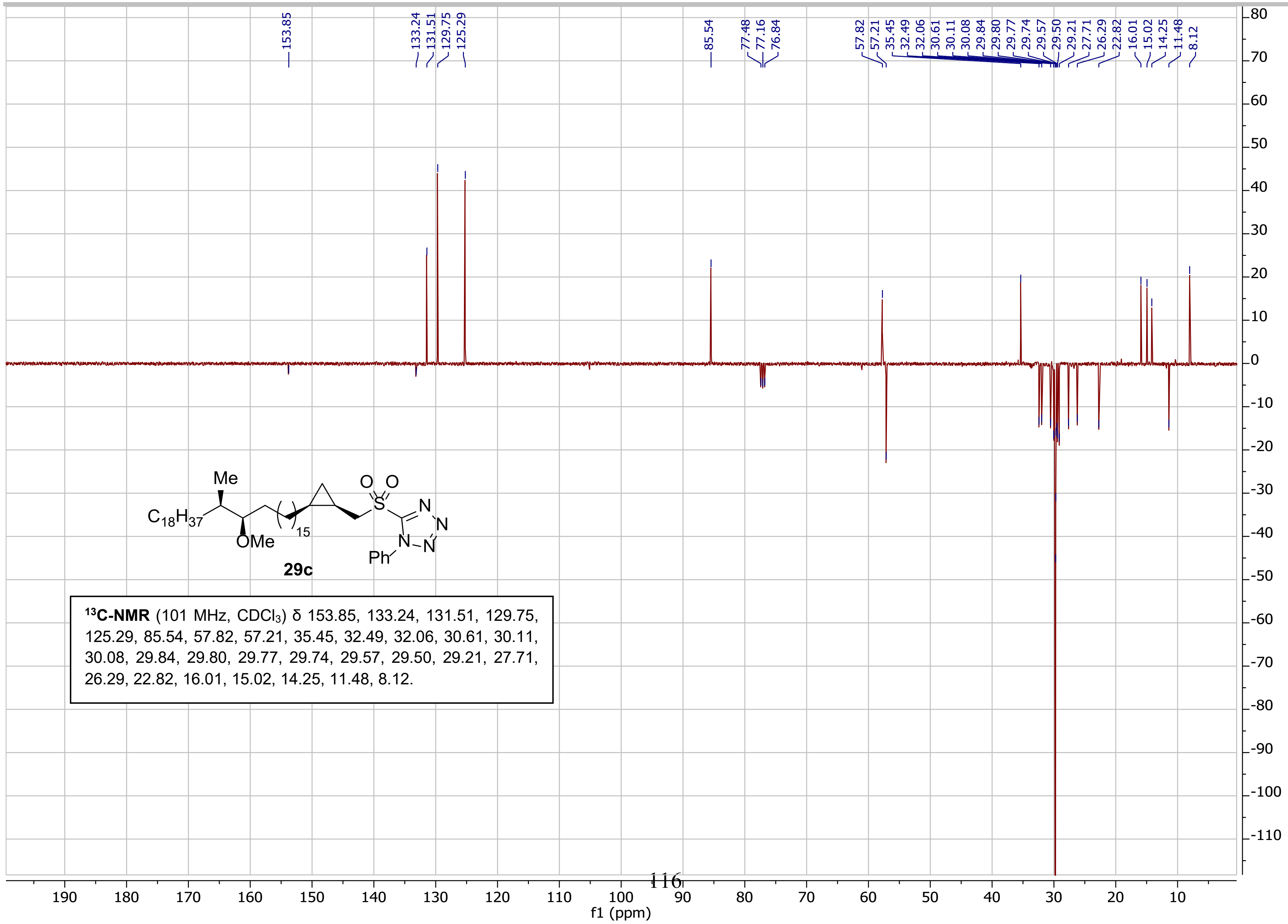


**29c**

**$^{13}$C-NMR** (101 MHz, $CDCl_3$) δ 153.85, 133.24, 131.51, 129.75, 125.29, 85.54, 57.82, 57.21, 35.45, 32.49, 32.06, 30.61, 30.11, 30.08, 29.84, 29.80, 29.77, 29.74, 29.57, 29.50, 29.21, 27.71, 26.29, 22.82, 16.01, 15.02, 14.25, 11.48, 8.12.

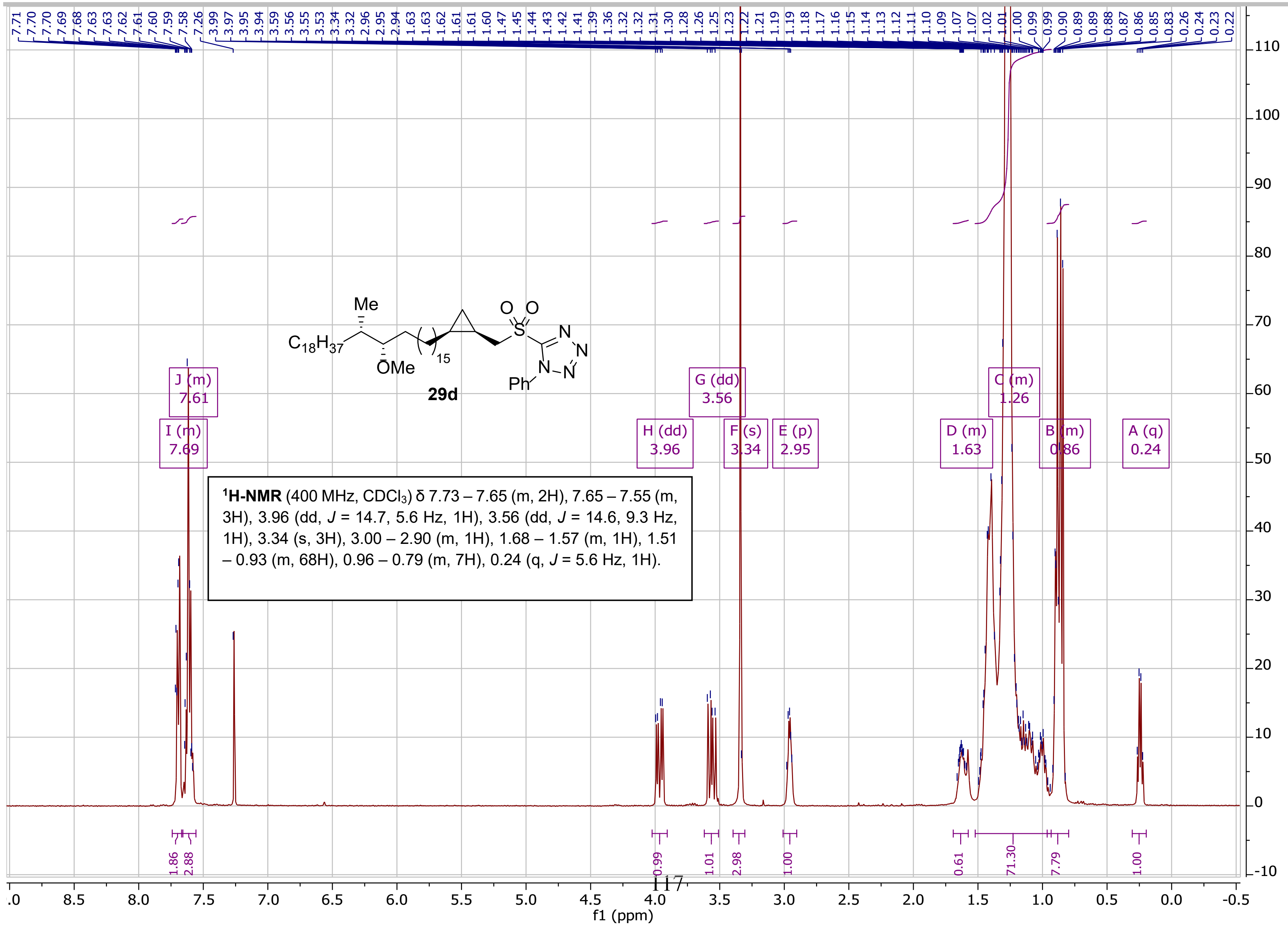

29d

$^{1}$**H-NMR** (400 MHz, $CDCl_3$) δ 7.73 – 7.65 (m, 2H), 7.65 – 7.55 (m, 3H), 3.96 (dd, $J$ = 14.7, 5.6 Hz, 1H), 3.56 (dd, $J$ = 14.6, 9.3 Hz, 1H), 3.34 (s, 3H), 3.00 – 2.90 (m, 1H), 1.68 – 1.57 (m, 1H), 1.51 – 0.93 (m, 68H), 0.96 – 0.79 (m, 7H), 0.24 (q, $J$ = 5.6 Hz, 1H).

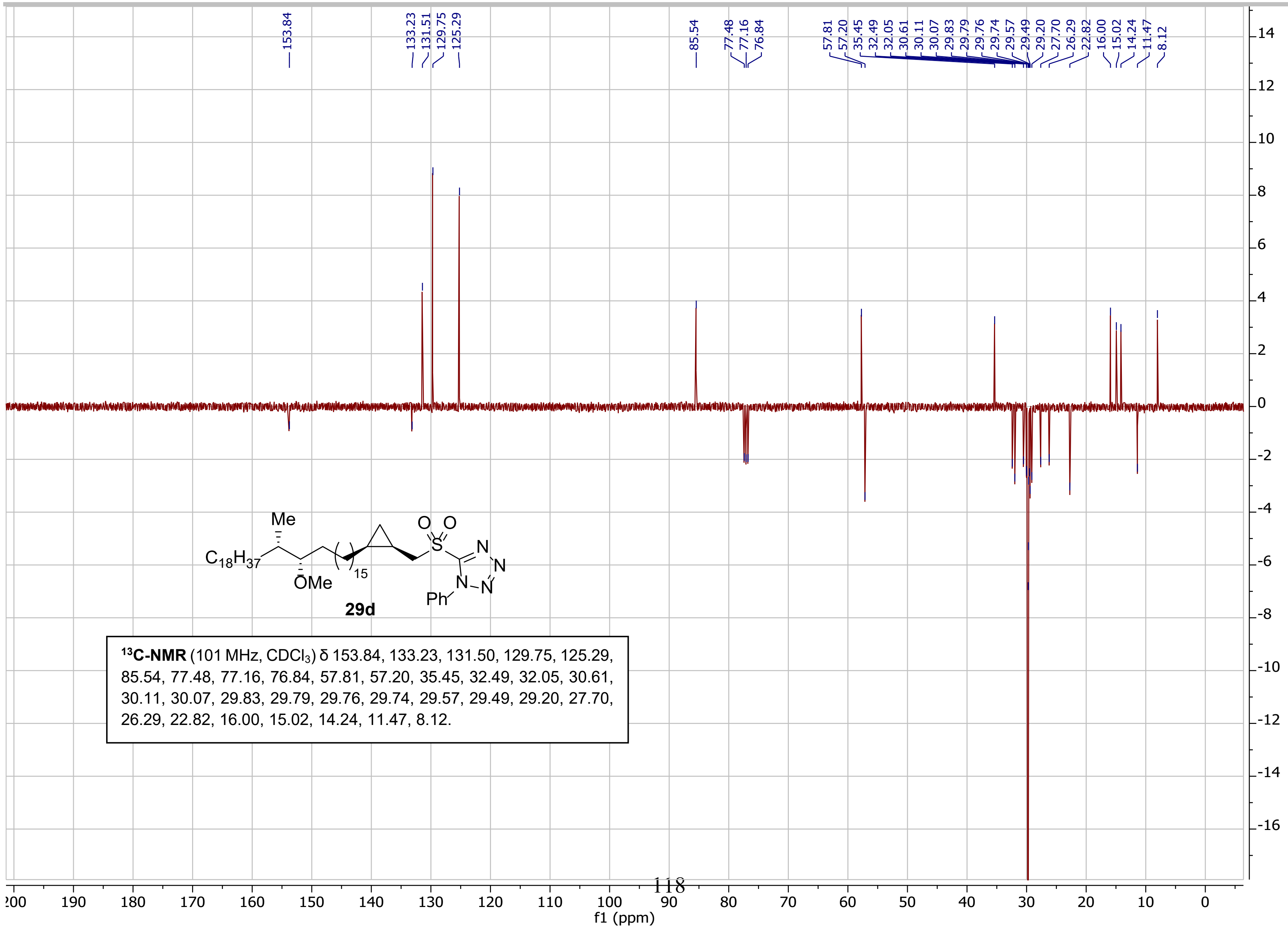

29d

$^{13}$**C-NMR** (101 MHz, $CDCl_3$) δ 153.84, 133.23, 131.50, 129.75, 125.29, 85.54, 77.48, 77.16, 76.84, 57.81, 57.20, 35.45, 32.49, 32.05, 30.61, 30.11, 30.07, 29.83, 29.79, 29.76, 29.74, 29.57, 29.49, 29.20, 27.70, 26.29, 22.82, 16.00, 15.02, 14.24, 11.47, 8.12.

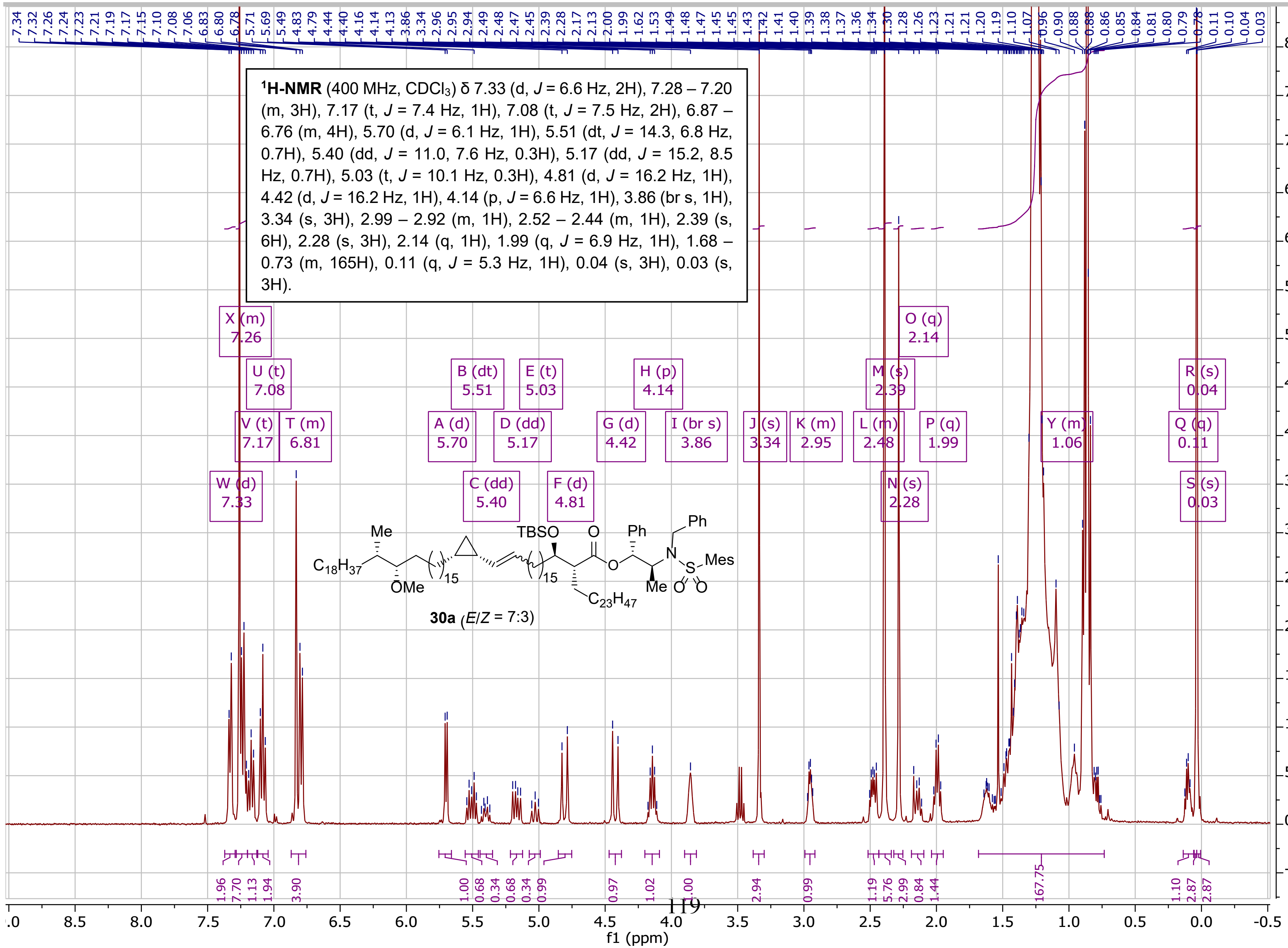

¹H-NMR (400 MHz, CDCl₃) δ 7.33 (d, J = 6.6 Hz, 2H), 7.28 – 7.20 (m, 3H), 7.17 (t, J = 7.4 Hz, 1H), 7.08 (t, J = 7.5 Hz, 2H), 6.87 – 6.76 (m, 4H), 5.70 (d, J = 6.1 Hz, 1H), 5.51 (dt, J = 14.3, 6.8 Hz, 0.7H), 5.40 (dd, J = 11.0, 7.6 Hz, 0.3H), 5.17 (dd, J = 15.2, 8.5 Hz, 0.7H), 5.03 (t, J = 10.1 Hz, 0.3H), 4.81 (d, J = 16.2 Hz, 1H), 4.42 (d, J = 16.2 Hz, 1H), 4.14 (p, J = 6.6 Hz, 1H), 3.86 (br s, 1H), 3.34 (s, 3H), 2.99 – 2.92 (m, 1H), 2.52 – 2.44 (m, 1H), 2.39 (s, 6H), 2.28 (s, 3H), 2.14 (q, 1H), 1.99 (q, J = 6.9 Hz, 1H), 1.68 – 0.73 (m, 165H), 0.11 (q, J = 5.3 Hz, 1H), 0.04 (s, 3H), 0.03 (s, 3H).
30a (E/Z = 7:3)
f1 (ppm)

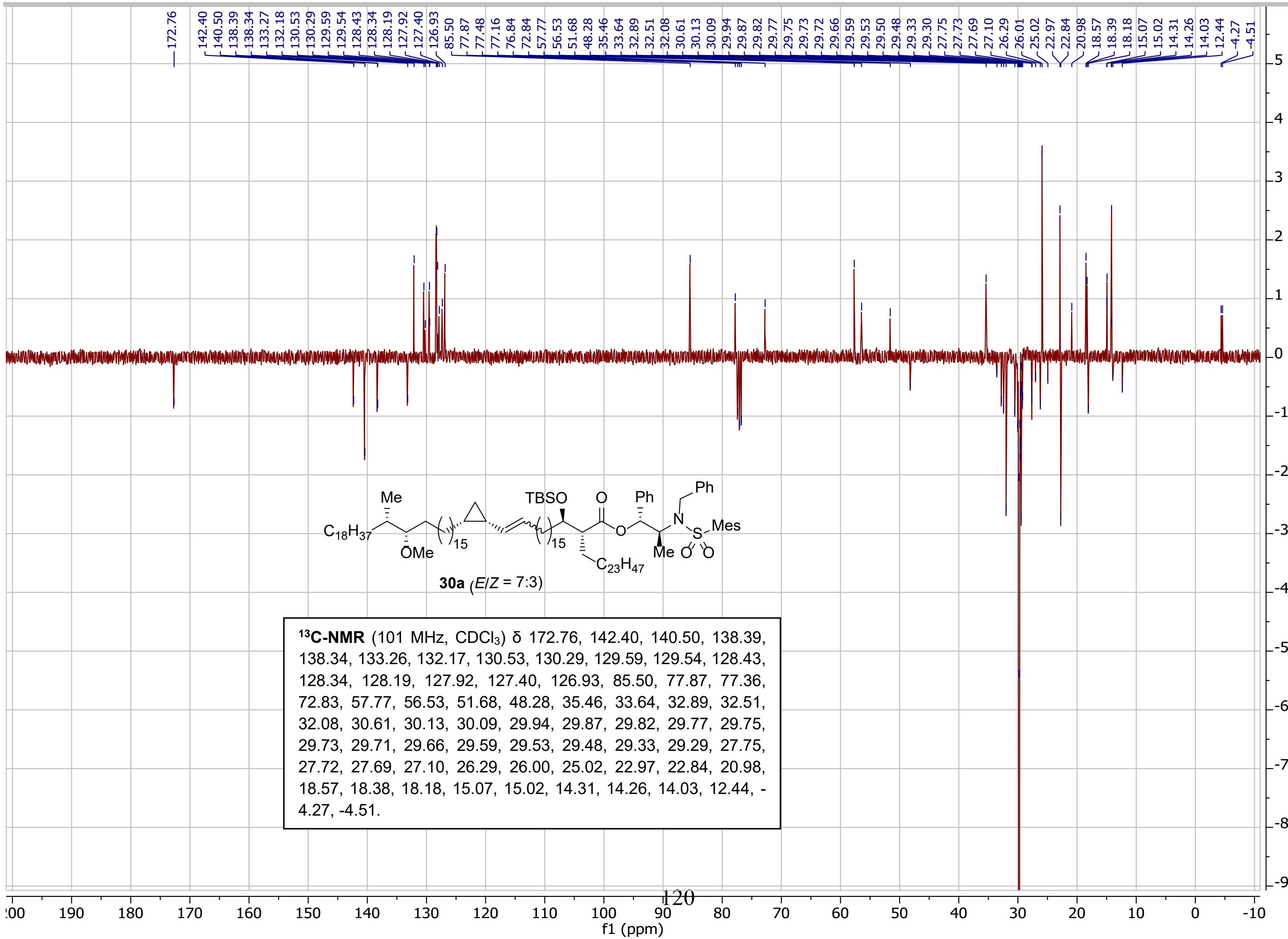


**$^{13}$C-NMR** (101 MHz, $CDCl_3$) δ 172.76, 142.40, 140.50, 138.39, 138.34, 133.26, 132.17, 130.53, 130.29, 129.59, 129.54, 128.43, 128.34, 128.19, 127.92, 127.40, 126.93, 85.50, 77.87, 77.36, 72.83, 57.77, 56.53, 51.68, 48.28, 35.46, 33.64, 32.89, 32.51, 32.08, 30.61, 30.13, 30.09, 29.94, 29.87, 29.82, 29.77, 29.75, 29.73, 29.71, 29.66, 29.59, 29.53, 29.48, 29.33, 29.29, 27.75, 27.72, 27.69, 27.10, 26.29, 26.00, 25.02, 22.97, 22.84, 20.98, 18.57, 18.38, 18.18, 15.07, 15.02, 14.31, 14.26, 14.03, 12.44, -4.27, -4.51.

**$^{1}$H-NMR** (400 MHz, $CDCl_3$) δ 7.33 (d, *J* = 6.5 Hz, 2H), 7.28 – 7.20 (m, 3H), 7.17 (t, *J* = 7.4 Hz, 1H), 7.08 (t, *J* = 7.5 Hz, 2H), 6.87 – 6.76 (m, 4H), 5.70 (d, *J* = 6.1 Hz, 1H), 5.51 (dt, *J* = 14.3, 6.8 Hz, 0.7H), 5.40 (dt, *J* = 10.7, 7.3 Hz, 0.3H), 5.17 (dd, *J* = 15.2, 8.5 Hz, 0.7H), 5.03 (t, *J* = 10.1 Hz, 0.3H), 4.81 (d, *J* = 16.2 Hz, 1H), 4.42 (d, *J* = 16.2 Hz, 1H), 4.14 (p, *J* = 6.7 Hz, 1H), 3.86 (br s, *J* = 6.1, 4.9 Hz, 1H), 3.34 (s, 3H), 3.02 – 2.90 (m, 1H), 2.48 (dt, *J* = 10.6, 5.6 Hz, 1H), 2.39 (s, 6H), 2.28 (s, 3H), 2.14 (q, *J* = 7.6, 7.1 Hz, 0.6H), 1.99 (q, *J* = 6.9 Hz, 1.4H), 1.70 – 0.73 (m, 165H), 0.11 (q, *J* = 5.3 Hz, 1H), 0.04 (s, 3H), 0.04 (s, 3H).

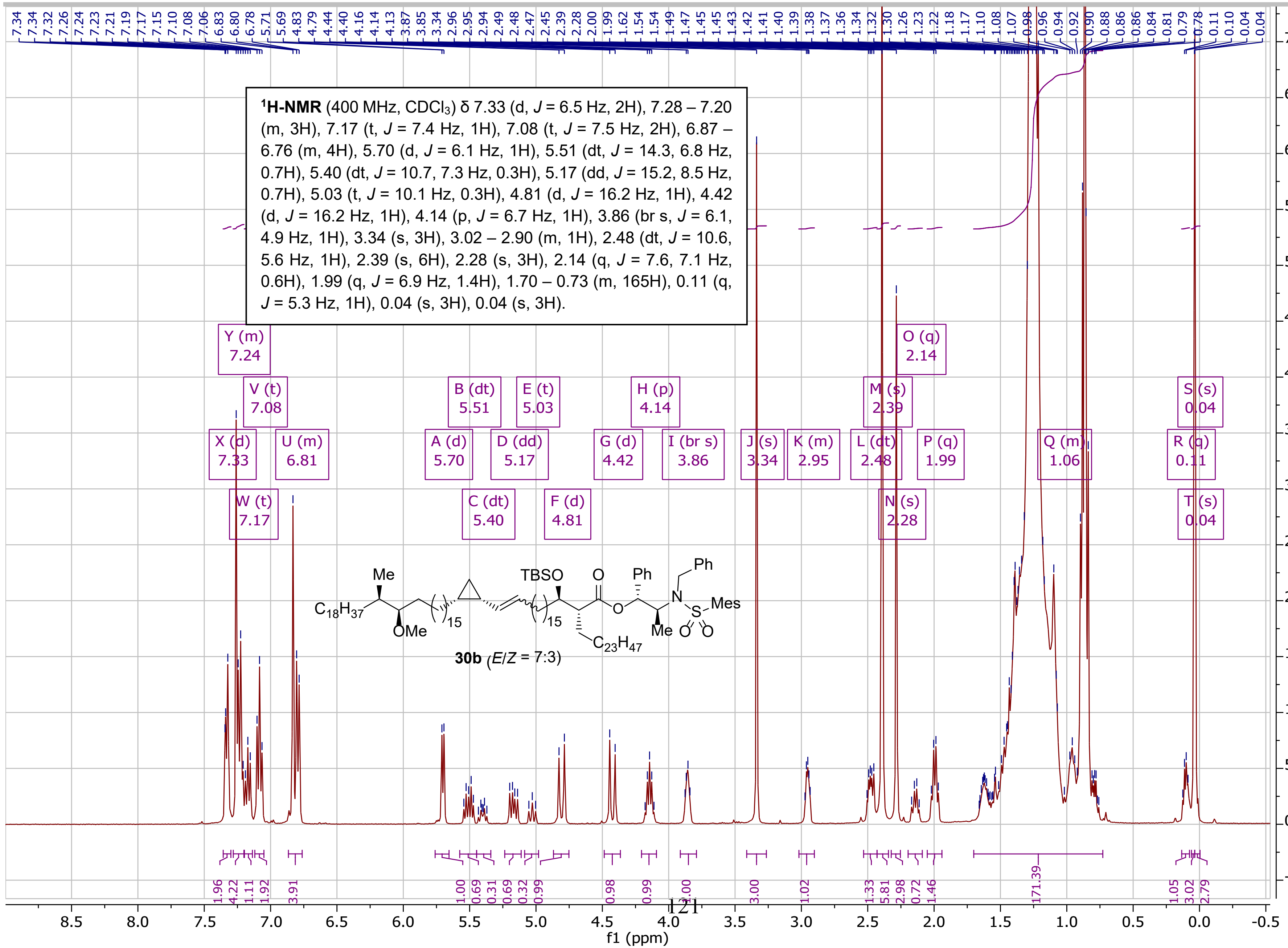

**30b** (*E*/*Z* = 7:3)

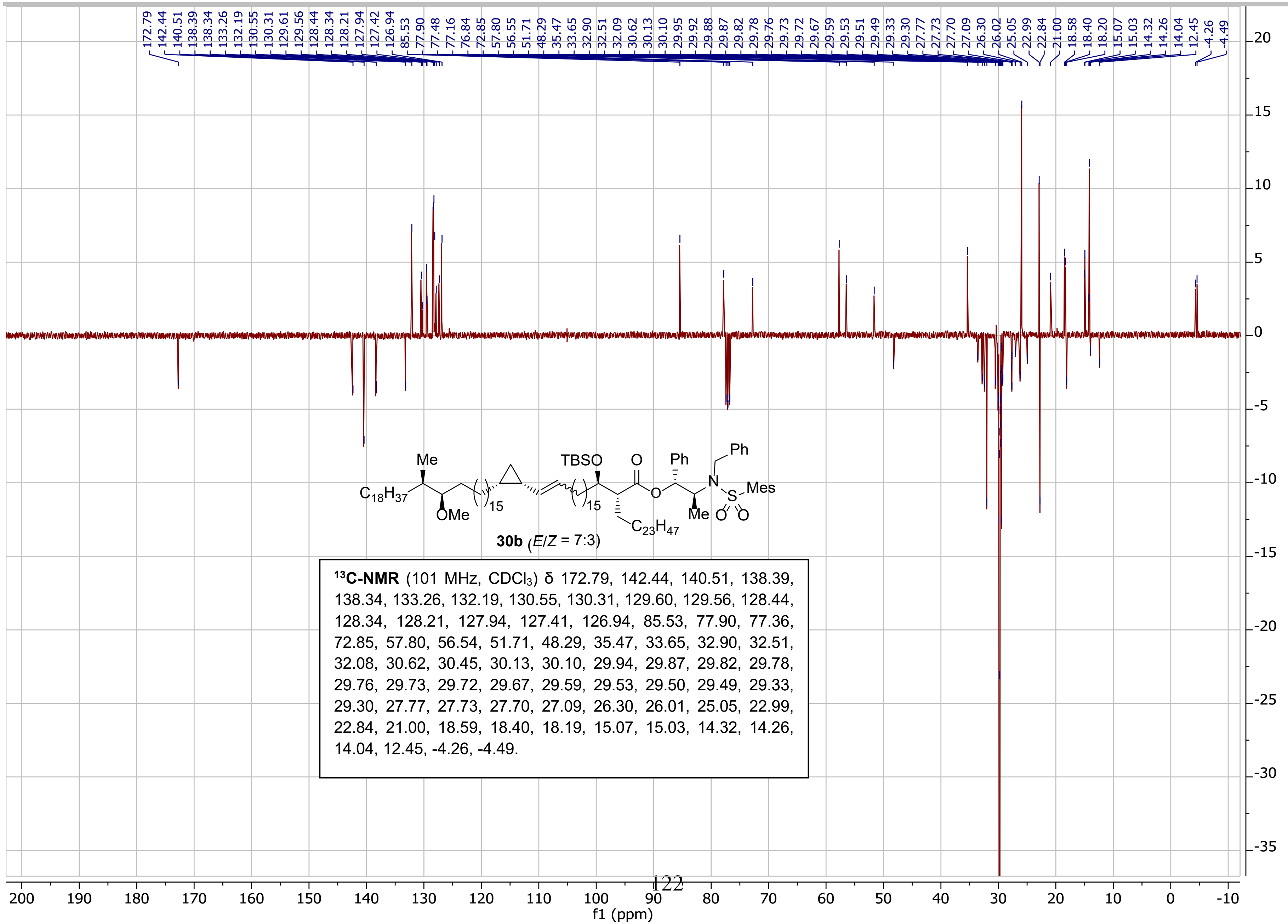


**30b** (*E*/*Z* = 7:3)

**$^{13}$C-NMR** (101 MHz, $CDCl_3$) δ 172.79, 142.44, 140.51, 138.39, 138.34, 133.26, 132.19, 130.55, 130.31, 129.60, 129.56, 128.44, 128.34, 128.21, 127.94, 127.41, 126.94, 85.53, 77.90, 77.36, 72.85, 57.80, 56.54, 51.71, 48.29, 35.47, 33.65, 32.90, 32.51, 32.08, 30.62, 30.45, 30.13, 30.10, 29.94, 29.87, 29.82, 29.78, 29.76, 29.73, 29.72, 29.67, 29.59, 29.53, 29.50, 29.49, 29.33, 29.30, 27.77, 27.73, 27.70, 27.09, 26.30, 26.01, 25.05, 22.99, 22.84, 21.00, 18.59, 18.40, 18.19, 15.07, 15.03, 14.32, 14.26, 14.04, 12.45, -4.26, -4.49.

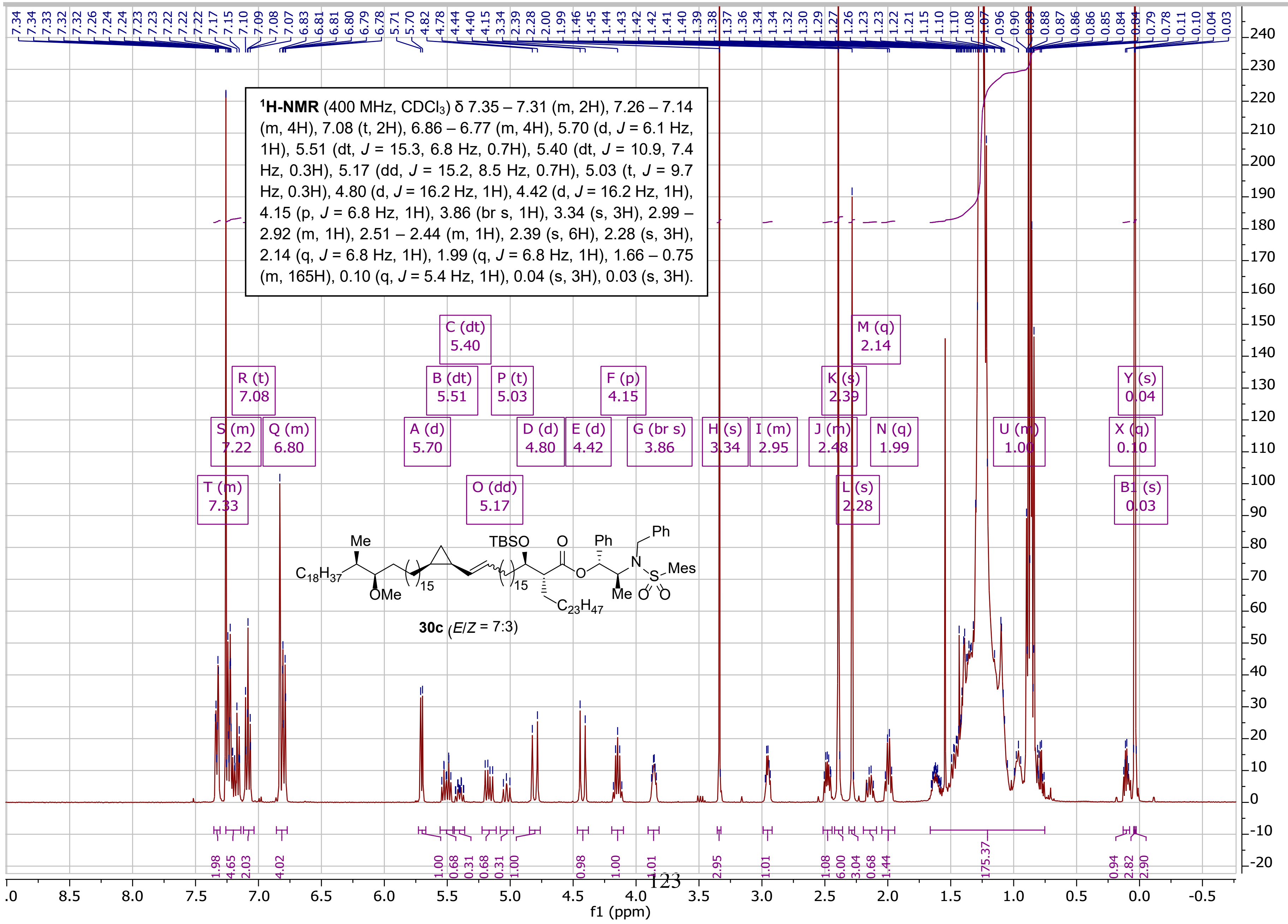
**¹H-NMR** (400 MHz, CDCl₃) δ 7.35 – 7.31 (m, 2H), 7.26 – 7.14 (m, 4H), 7.08 (t, 2H), 6.86 – 6.77 (m, 4H), 5.70 (d, J = 6.1 Hz, 1H), 5.51 (dt, J = 15.3, 6.8 Hz, 0.7H), 5.40 (dt, J = 10.9, 7.4 Hz, 0.3H), 5.17 (dd, J = 15.2, 8.5 Hz, 0.7H), 5.03 (t, J = 9.7 Hz, 0.3H), 4.80 (d, J = 16.2 Hz, 1H), 4.42 (d, J = 16.2 Hz, 1H), 4.15 (p, J = 6.8 Hz, 1H), 3.86 (br s, 1H), 3.34 (s, 3H), 2.99 – 2.92 (m, 1H), 2.51 – 2.44 (m, 1H), 2.39 (s, 6H), 2.28 (s, 3H), 2.14 (q, J = 6.8 Hz, 1H), 1.99 (q, J = 6.8 Hz, 1H), 1.66 – 0.75 (m, 165H), 0.10 (q, J = 5.4 Hz, 1H), 0.04 (s, 3H), 0.03 (s, 3H).
**30c** (E/Z = 7:3)
f1 (ppm)

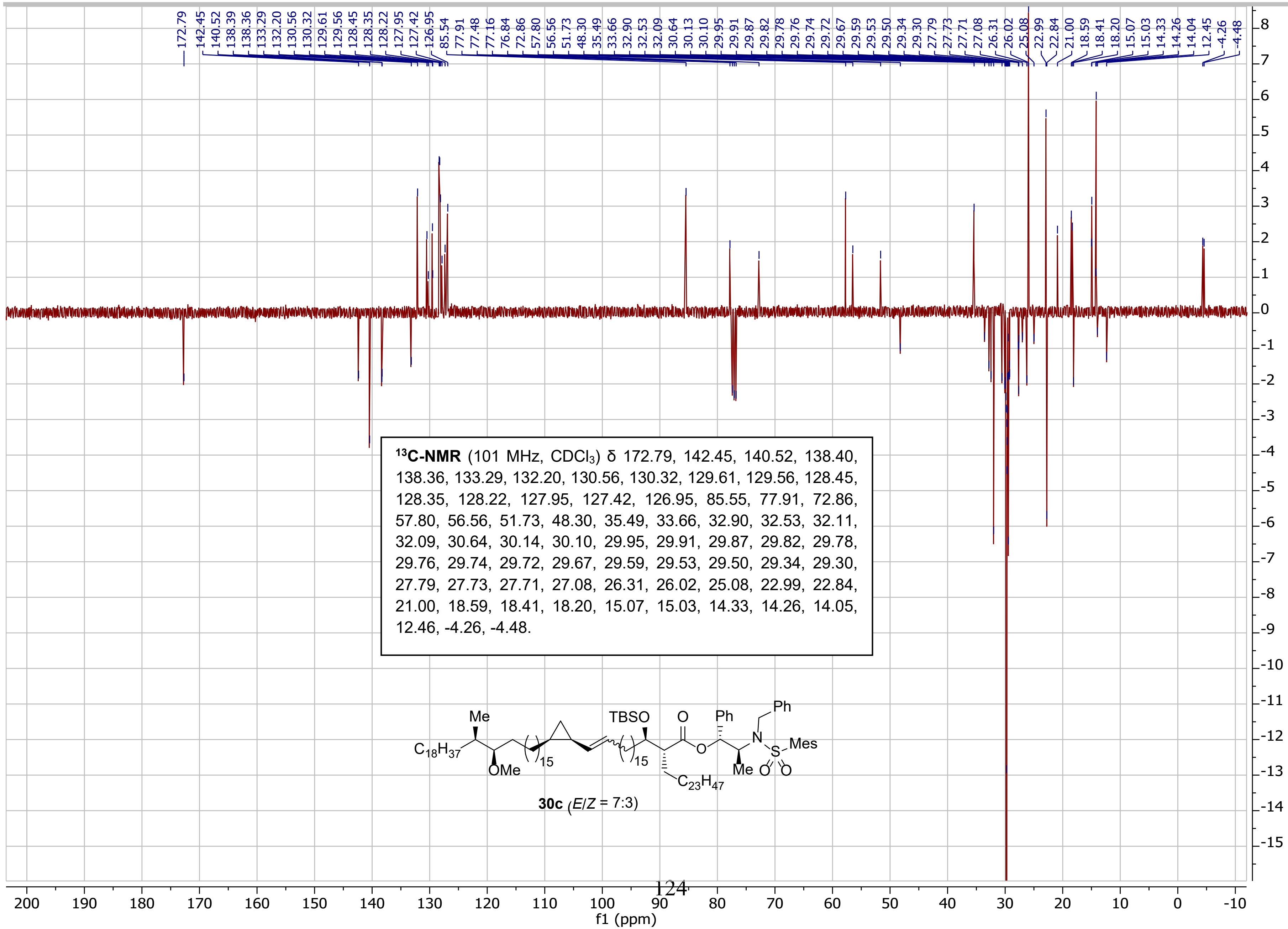


**30c** ($E/Z$ = 7:3)

**$^{13}$C-NMR** (101 MHz, $CDCl_3$) δ 172.79, 142.45, 140.52, 138.40, 138.36, 133.29, 132.20, 130.56, 130.32, 129.61, 129.56, 128.45, 128.35, 128.22, 127.95, 127.42, 126.95, 85.55, 77.91, 72.86, 57.80, 56.56, 51.73, 48.30, 35.49, 33.66, 32.90, 32.53, 32.11, 32.09, 30.64, 30.14, 30.10, 29.95, 29.91, 29.87, 29.82, 29.78, 29.76, 29.74, 29.72, 29.67, 29.59, 29.53, 29.50, 29.34, 29.30, 27.79, 27.73, 27.71, 27.08, 26.31, 26.02, 25.08, 22.99, 22.84, 21.00, 18.59, 18.41, 18.20, 15.07, 15.03, 14.33, 14.26, 14.05, 12.46, -4.26, -4.48.

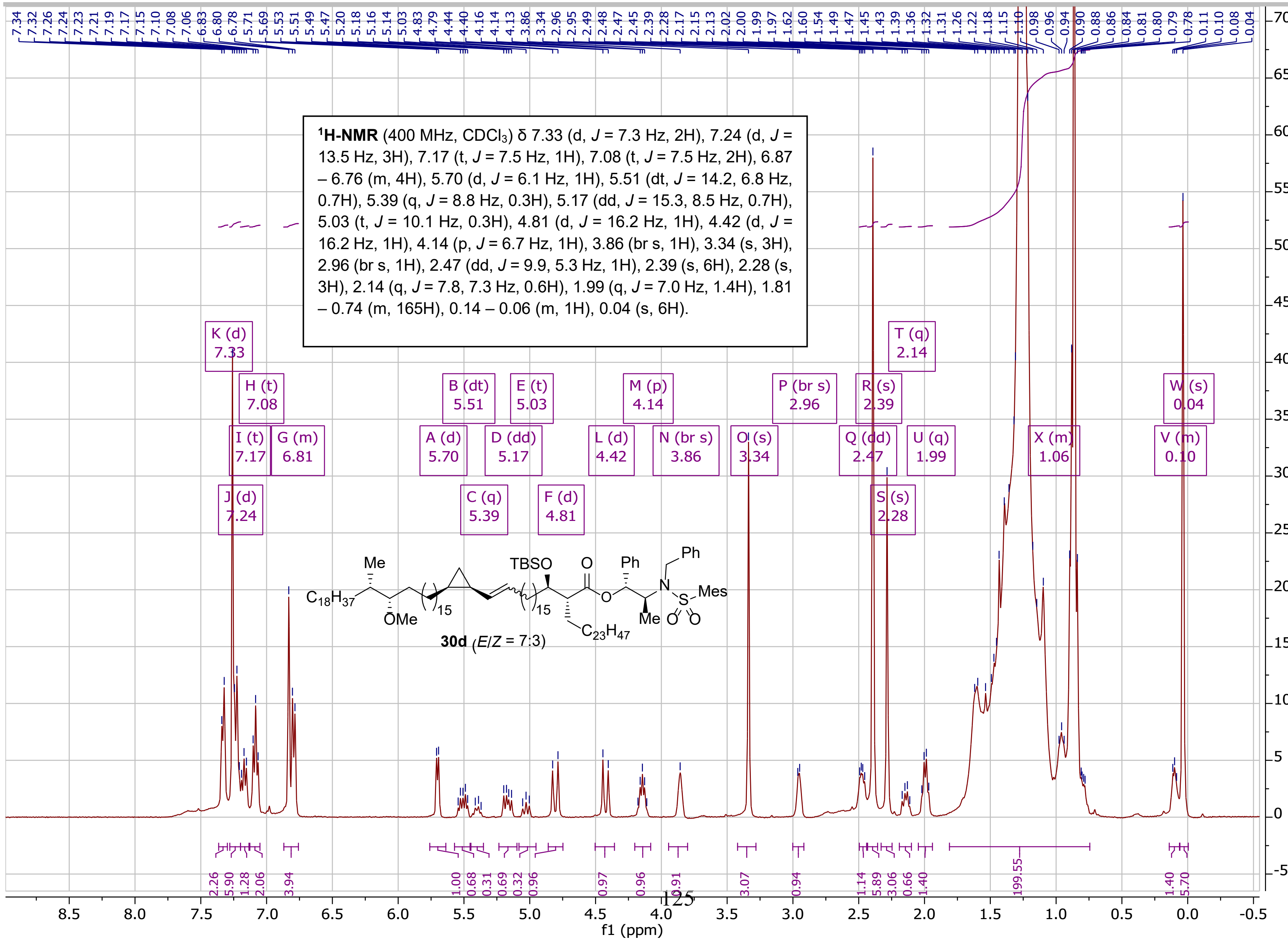
¹H-NMR (400 MHz, CDCl₃) δ 7.33 (d, J = 7.3 Hz, 2H), 7.24 (d, J = 13.5 Hz, 3H), 7.17 (t, J = 7.5 Hz, 1H), 7.08 (t, J = 7.5 Hz, 2H), 6.87 – 6.76 (m, 4H), 5.70 (d, J = 6.1 Hz, 1H), 5.51 (dt, J = 14.2, 6.8 Hz, 0.7H), 5.39 (q, J = 8.8 Hz, 0.3H), 5.17 (dd, J = 15.3, 8.5 Hz, 0.7H), 5.03 (t, J = 10.1 Hz, 0.3H), 4.81 (d, J = 16.2 Hz, 1H), 4.42 (d, J = 16.2 Hz, 1H), 4.14 (p, J = 6.7 Hz, 1H), 3.86 (br s, 1H), 3.34 (s, 3H), 2.96 (br s, 1H), 2.47 (dd, J = 9.9, 5.3 Hz, 1H), 2.39 (s, 6H), 2.28 (s, 3H), 2.14 (q, J = 7.8, 7.3 Hz, 0.6H), 1.99 (q, J = 7.0 Hz, 1.4H), 1.81 – 0.74 (m, 165H), 0.14 – 0.06 (m, 1H), 0.04 (s, 6H).
30d (E/Z = 7:3)
f1 (ppm)

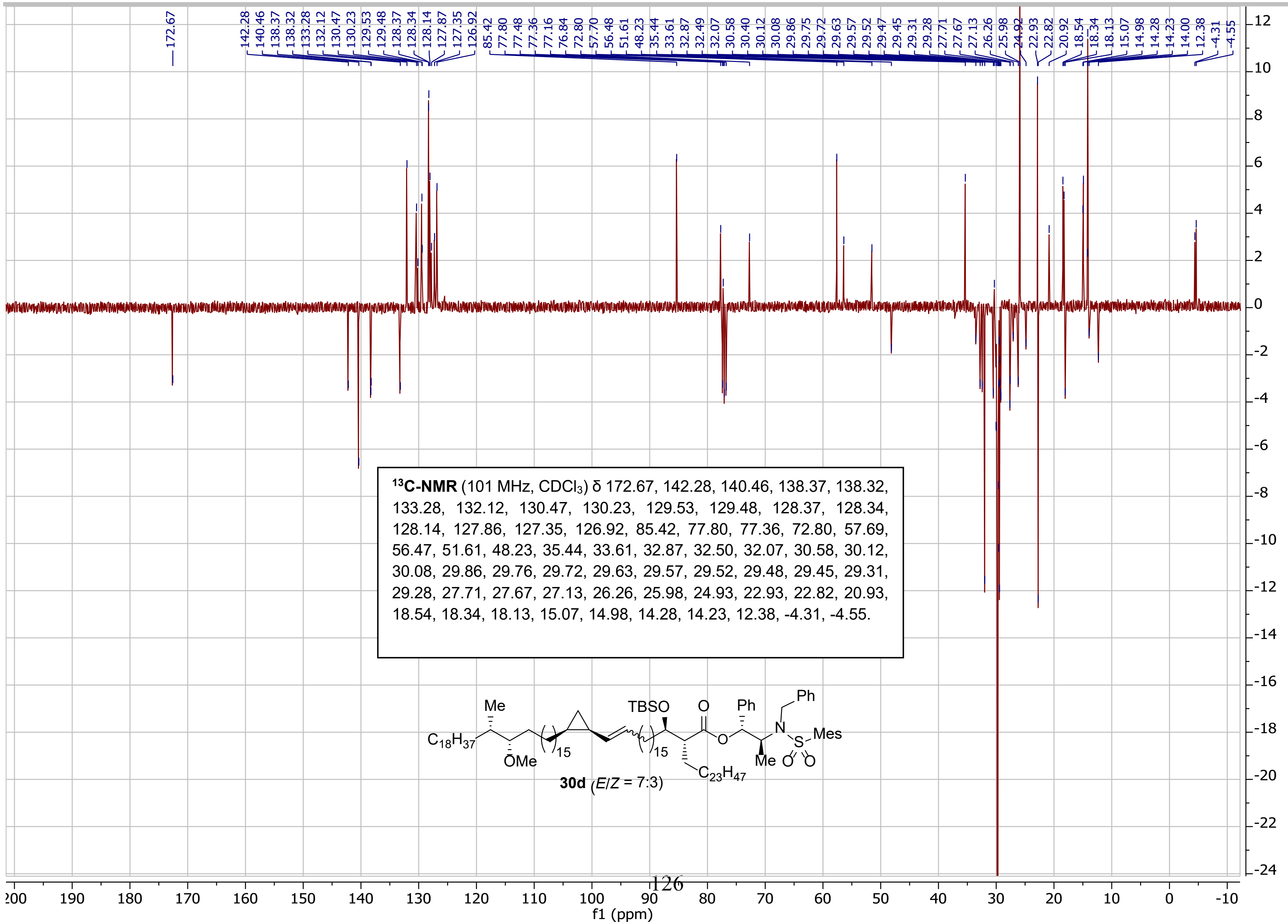


**$^{13}$C-NMR** (101 MHz, $CDCl_3$) δ 172.67, 142.28, 140.46, 138.37, 138.32, 133.28, 132.12, 130.47, 130.23, 129.53, 129.48, 128.37, 128.34, 128.14, 127.86, 127.35, 126.92, 85.42, 77.80, 77.36, 72.80, 57.69, 56.47, 51.61, 48.23, 35.44, 33.61, 32.87, 32.50, 32.07, 30.58, 30.12, 30.08, 29.86, 29.76, 29.72, 29.63, 29.57, 29.52, 29.48, 29.45, 29.31, 29.28, 27.71, 27.67, 27.13, 26.26, 25.98, 24.93, 22.93, 22.82, 20.93, 18.54, 18.34, 18.13, 15.07, 14.98, 14.28, 14.23, 12.38, -4.31, -4.55.



**30d** (*E*/*Z* = 7:3)

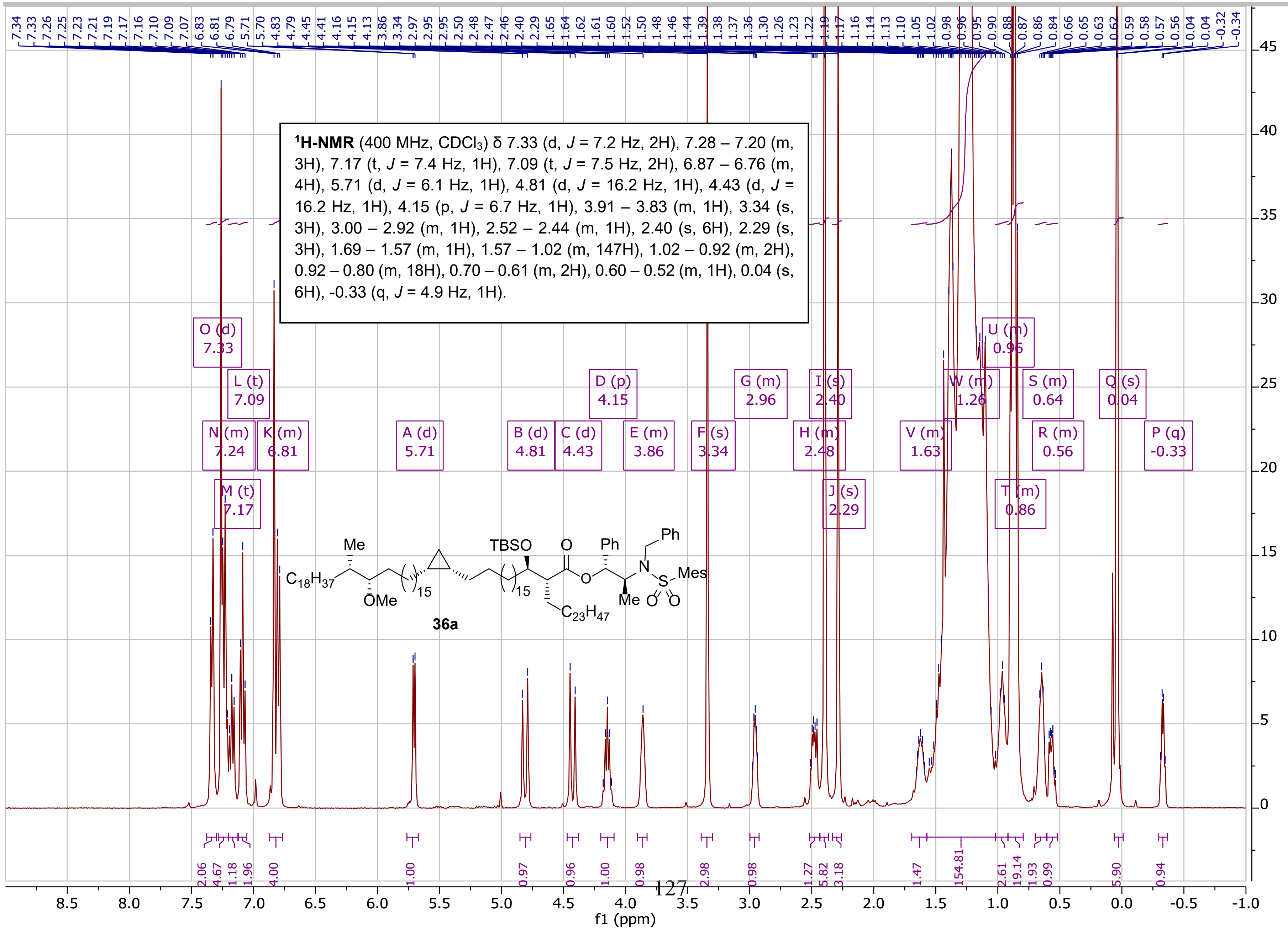
[1]**H-NMR** (400 MHz, $CDCl_3$) δ 7.33 (d, *J* = 7.2 Hz, 2H), 7.28 – 7.20 (m, 3H), 7.17 (t, *J* = 7.4 Hz, 1H), 7.09 (t, *J* = 7.5 Hz, 2H), 6.87 – 6.76 (m, 4H), 5.71 (d, *J* = 6.1 Hz, 1H), 4.81 (d, *J* = 16.2 Hz, 1H), 4.43 (d, *J* = 16.2 Hz, 1H), 4.15 (p, *J* = 6.7 Hz, 1H), 3.91 – 3.83 (m, 1H), 3.34 (s, 3H), 3.00 – 2.92 (m, 1H), 2.52 – 2.44 (m, 1H), 2.40 (s, 6H), 2.29 (s, 3H), 1.69 – 1.57 (m, 1H), 1.57 – 1.02 (m, 147H), 1.02 – 0.92 (m, 2H), 0.92 – 0.80 (m, 18H), 0.70 – 0.61 (m, 2H), 0.60 – 0.52 (m, 1H), 0.04 (s, 6H), -0.33 (q, *J* = 4.9 Hz, 1H).
36a
f1 (ppm)

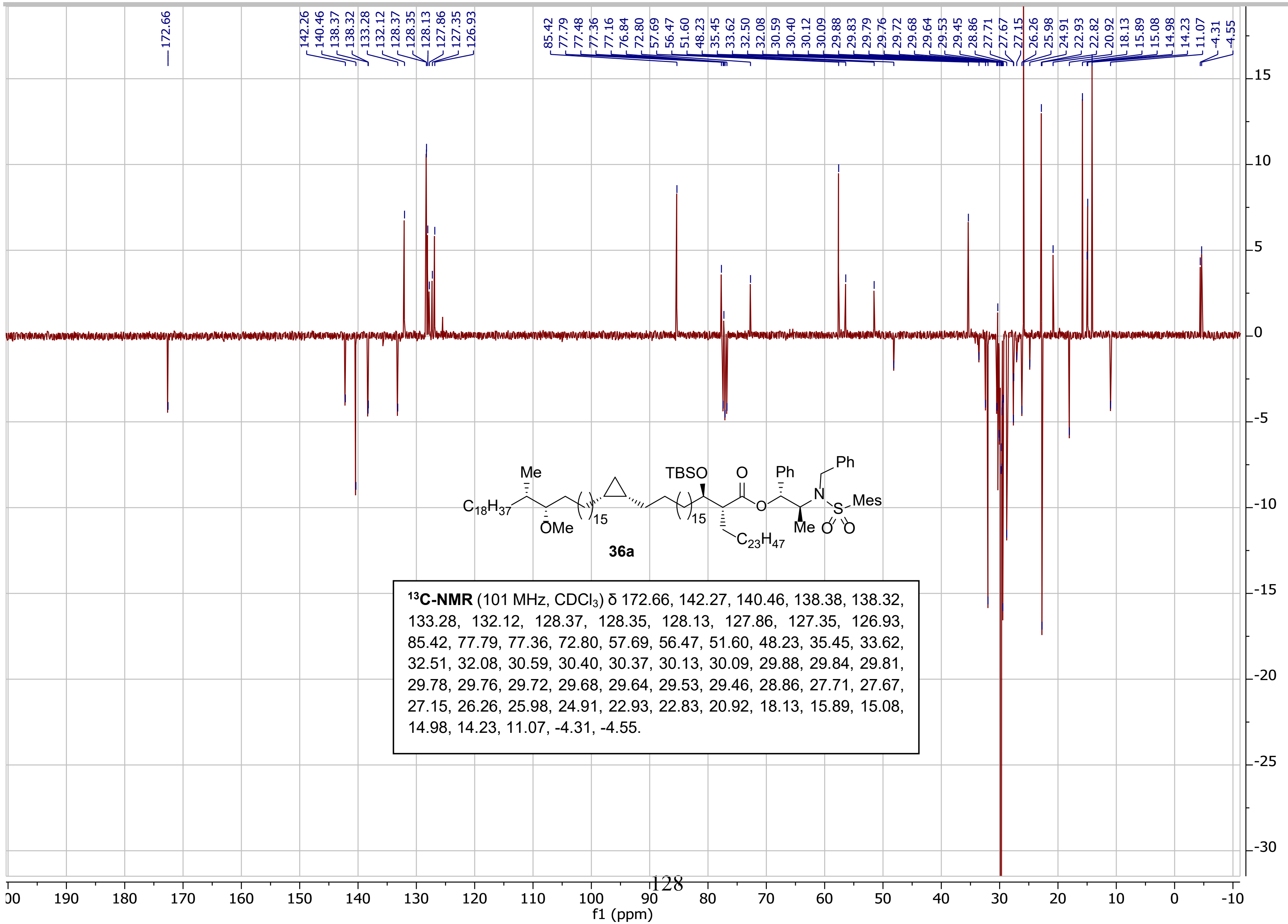


**36a**

**$^{13}$C-NMR** (101 MHz, $CDCl_3$) δ 172.66, 142.27, 140.46, 138.38, 138.32, 133.28, 132.12, 128.37, 128.35, 128.13, 127.86, 127.35, 126.93, 85.42, 77.79, 77.36, 72.80, 57.69, 56.47, 51.60, 48.23, 35.45, 33.62, 32.51, 32.08, 30.59, 30.40, 30.37, 30.13, 30.09, 29.88, 29.84, 29.81, 29.78, 29.76, 29.72, 29.68, 29.64, 29.53, 29.46, 28.86, 27.71, 27.67, 27.15, 26.26, 25.98, 24.91, 22.93, 22.83, 20.92, 18.13, 15.89, 15.08, 14.98, 14.23, 11.07, -4.31, -4.55.

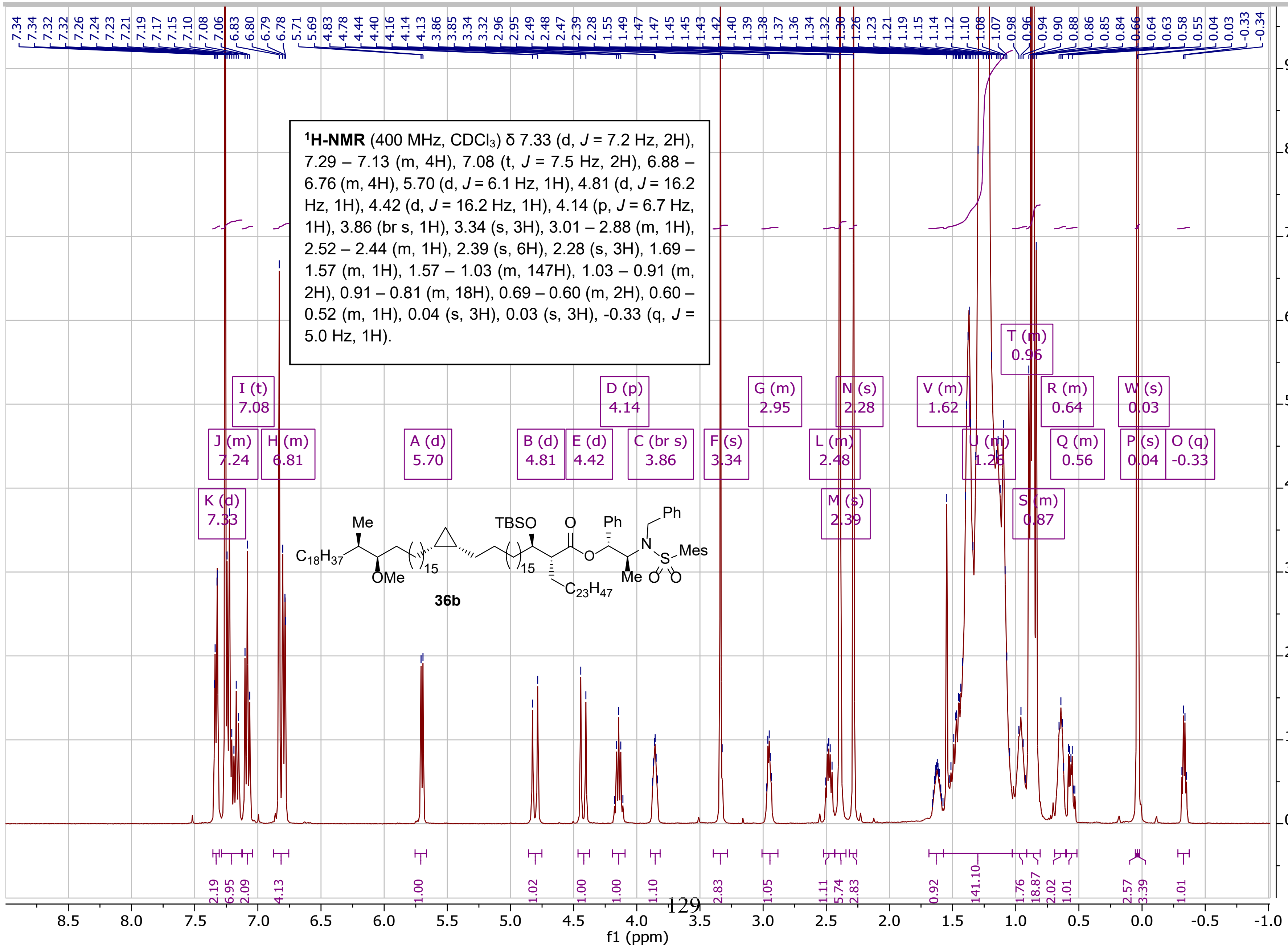


**$^1$H-NMR** (400 MHz, $CDCl_3$) δ 7.33 (d, *J* = 7.2 Hz, 2H), 7.29 – 7.13 (m, 4H), 7.08 (t, *J* = 7.5 Hz, 2H), 6.88 – 6.76 (m, 4H), 5.70 (d, *J* = 6.1 Hz, 1H), 4.81 (d, *J* = 16.2 Hz, 1H), 4.42 (d, *J* = 16.2 Hz, 1H), 4.14 (p, *J* = 6.7 Hz, 1H), 3.86 (br s, 1H), 3.34 (s, 3H), 3.01 – 2.88 (m, 1H), 2.52 – 2.44 (m, 1H), 2.39 (s, 6H), 2.28 (s, 3H), 1.69 – 1.57 (m, 1H), 1.57 – 1.03 (m, 147H), 1.03 – 0.91 (m, 2H), 0.91 – 0.81 (m, 18H), 0.69 – 0.60 (m, 2H), 0.60 – 0.52 (m, 1H), 0.04 (s, 3H), 0.03 (s, 3H), -0.33 (q, *J* = 5.0 Hz, 1H).

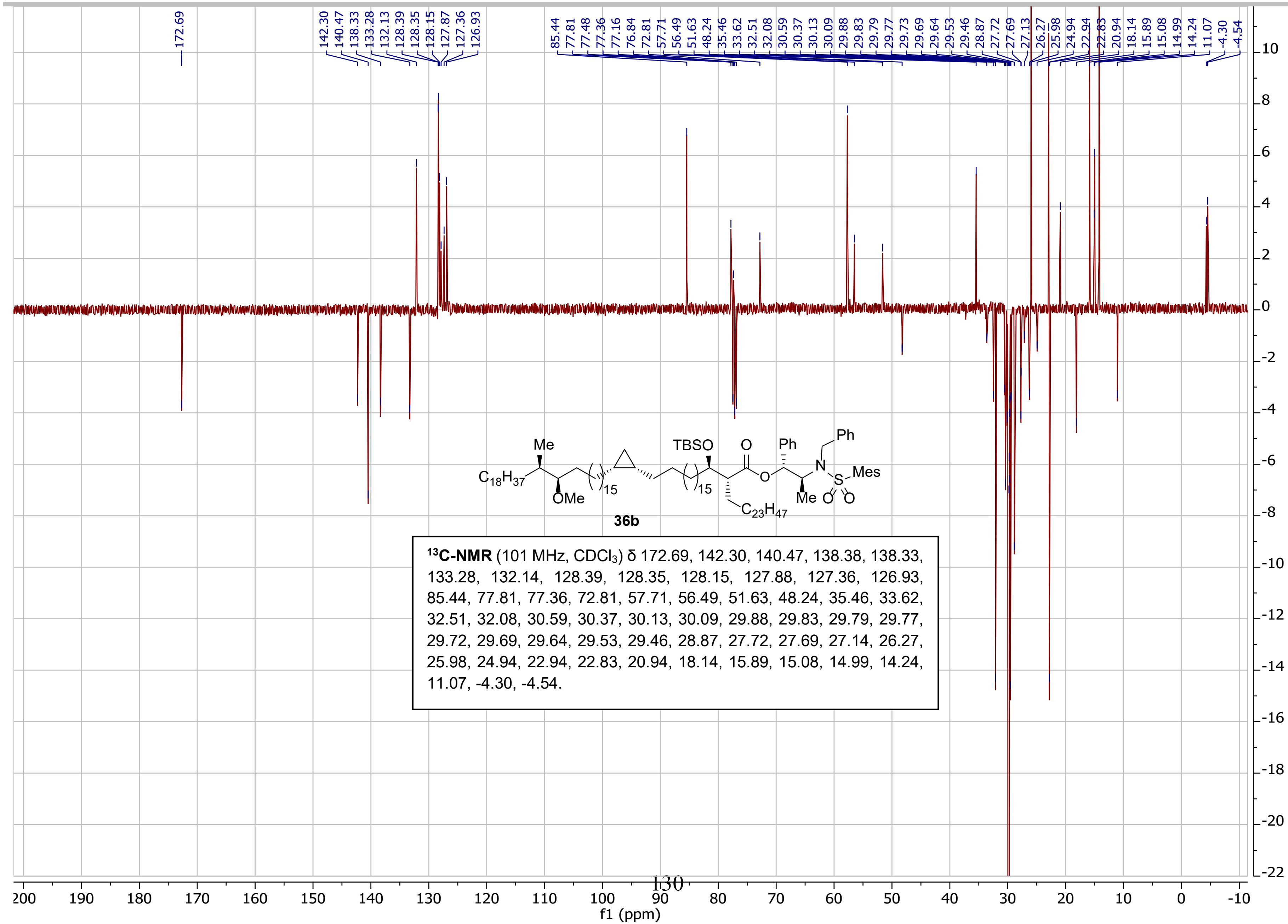

**36b**

**$^{13}$C-NMR** (101 MHz, $CDCl_3$) δ 172.69, 142.30, 140.47, 138.38, 138.33, 133.28, 132.14, 128.39, 128.35, 128.15, 127.88, 127.36, 126.93, 85.44, 77.81, 77.36, 72.81, 57.71, 56.49, 51.63, 48.24, 35.46, 33.62, 32.51, 32.08, 30.59, 30.37, 30.13, 30.09, 29.88, 29.83, 29.79, 29.77, 29.72, 29.69, 29.64, 29.53, 29.46, 28.87, 27.72, 27.69, 27.14, 26.27, 25.98, 24.94, 22.94, 22.83, 20.94, 18.14, 15.89, 15.08, 14.99, 14.24, 11.07, -4.30, -4.54.

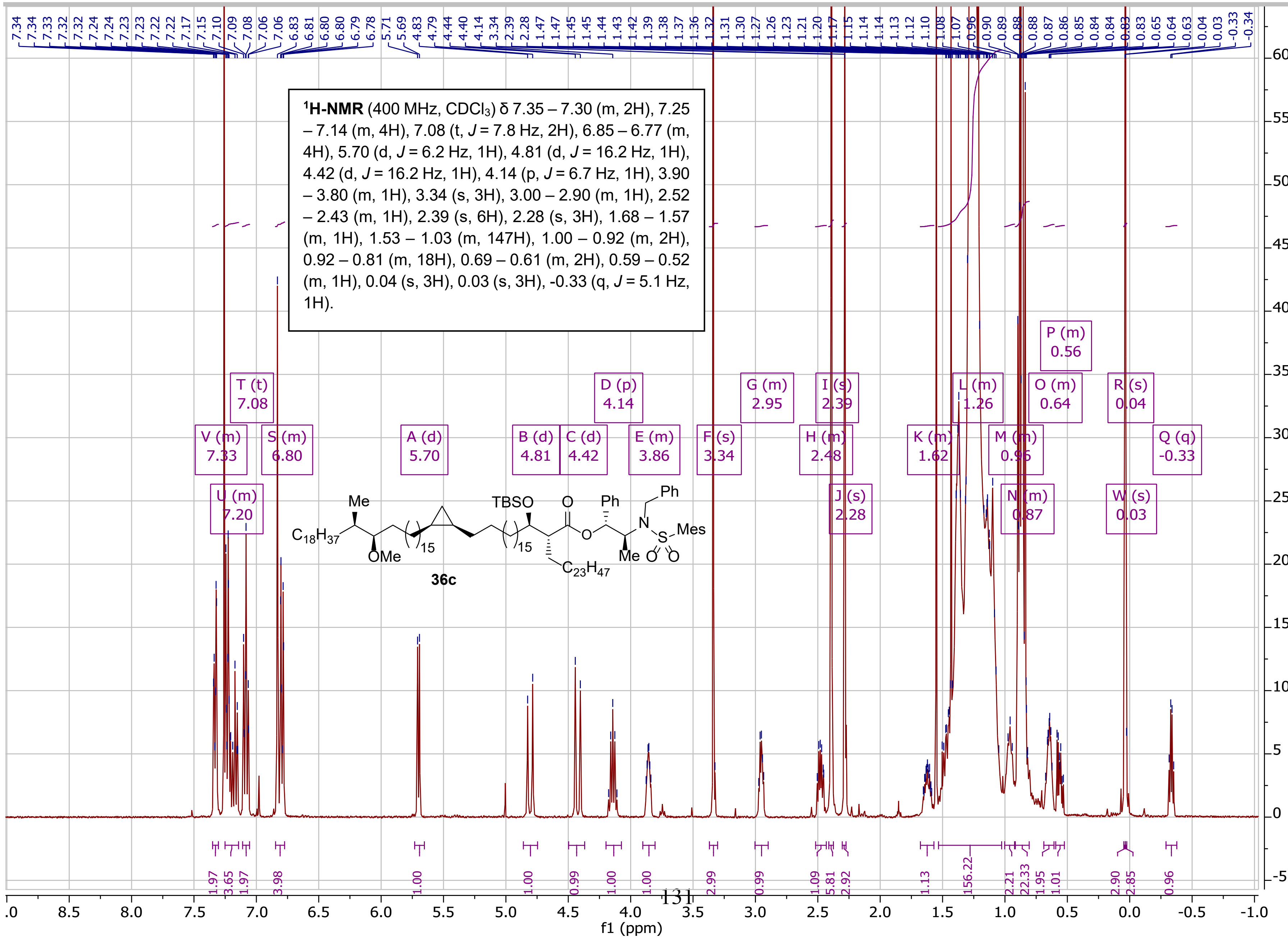
$^{1}$H-NMR (400 MHz, CDCl3) δ 7.35 – 7.30 (m, 2H), 7.25 – 7.14 (m, 4H), 7.08 (t, J = 7.8 Hz, 2H), 6.85 – 6.77 (m, 4H), 5.70 (d, J = 6.2 Hz, 1H), 4.81 (d, J = 16.2 Hz, 1H), 4.42 (d, J = 16.2 Hz, 1H), 4.14 (p, J = 6.7 Hz, 1H), 3.90 – 3.80 (m, 1H), 3.34 (s, 3H), 3.00 – 2.90 (m, 1H), 2.52 – 2.43 (m, 1H), 2.39 (s, 6H), 2.28 (s, 3H), 1.68 – 1.57 (m, 1H), 1.53 – 1.03 (m, 147H), 1.00 – 0.92 (m, 2H), 0.92 – 0.81 (m, 18H), 0.69 – 0.61 (m, 2H), 0.59 – 0.52 (m, 1H), 0.04 (s, 3H), 0.03 (s, 3H), -0.33 (q, J = 5.1 Hz, 1H).
36c
f1 (ppm)

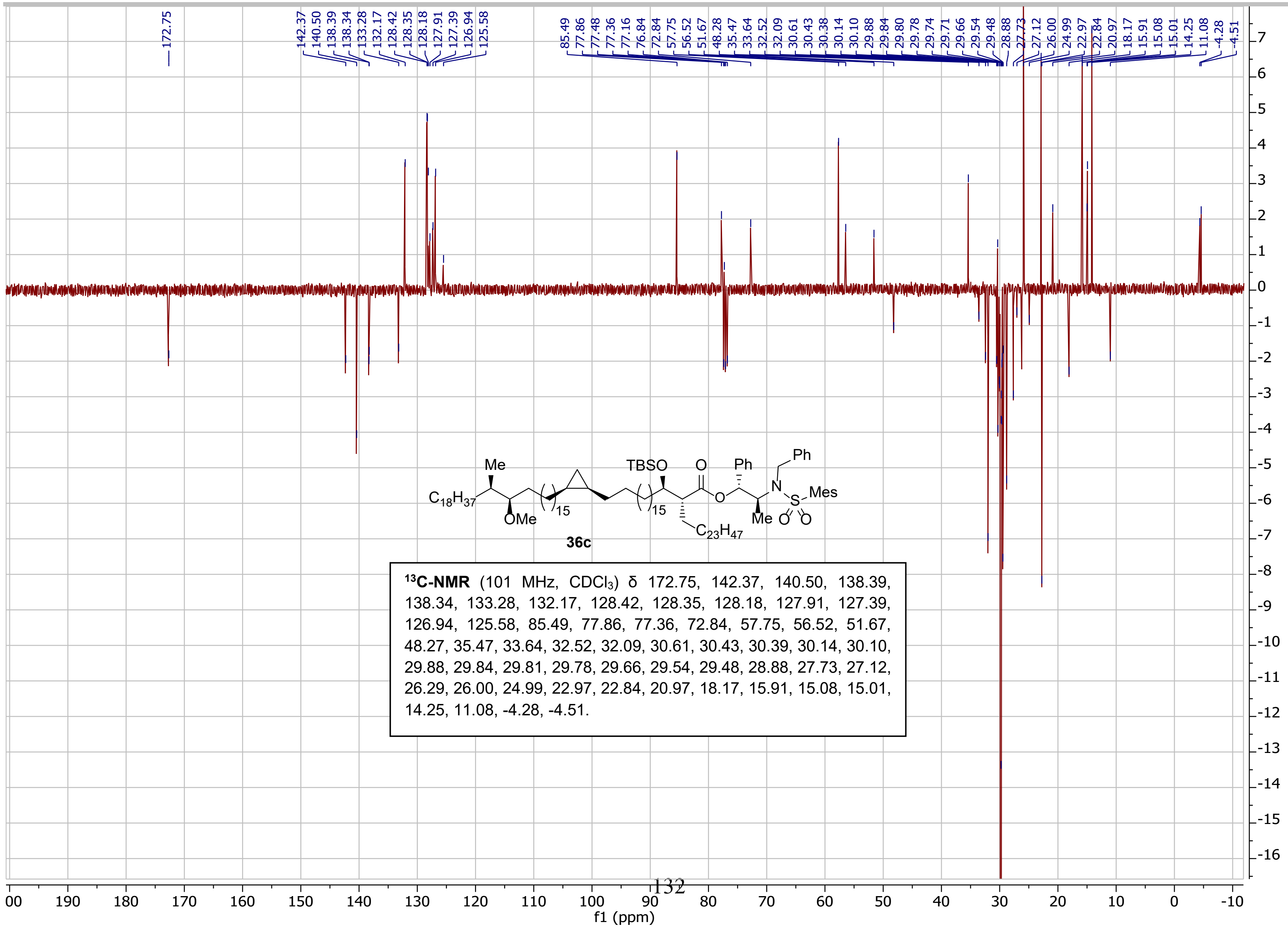


**$^{13}$C-NMR** (101 MHz, $CDCl_3$) δ 172.75, 142.37, 140.50, 138.39, 138.34, 133.28, 132.17, 128.42, 128.35, 128.18, 127.91, 127.39, 126.94, 125.58, 85.49, 77.86, 77.36, 72.84, 57.75, 56.52, 51.67, 48.27, 35.47, 33.64, 32.52, 32.09, 30.61, 30.43, 30.39, 30.14, 30.10, 29.88, 29.84, 29.81, 29.78, 29.66, 29.54, 29.48, 28.88, 27.73, 27.12, 26.29, 26.00, 24.99, 22.97, 22.84, 20.97, 18.17, 15.91, 15.08, 15.01, 14.25, 11.08, -4.28, -4.51.

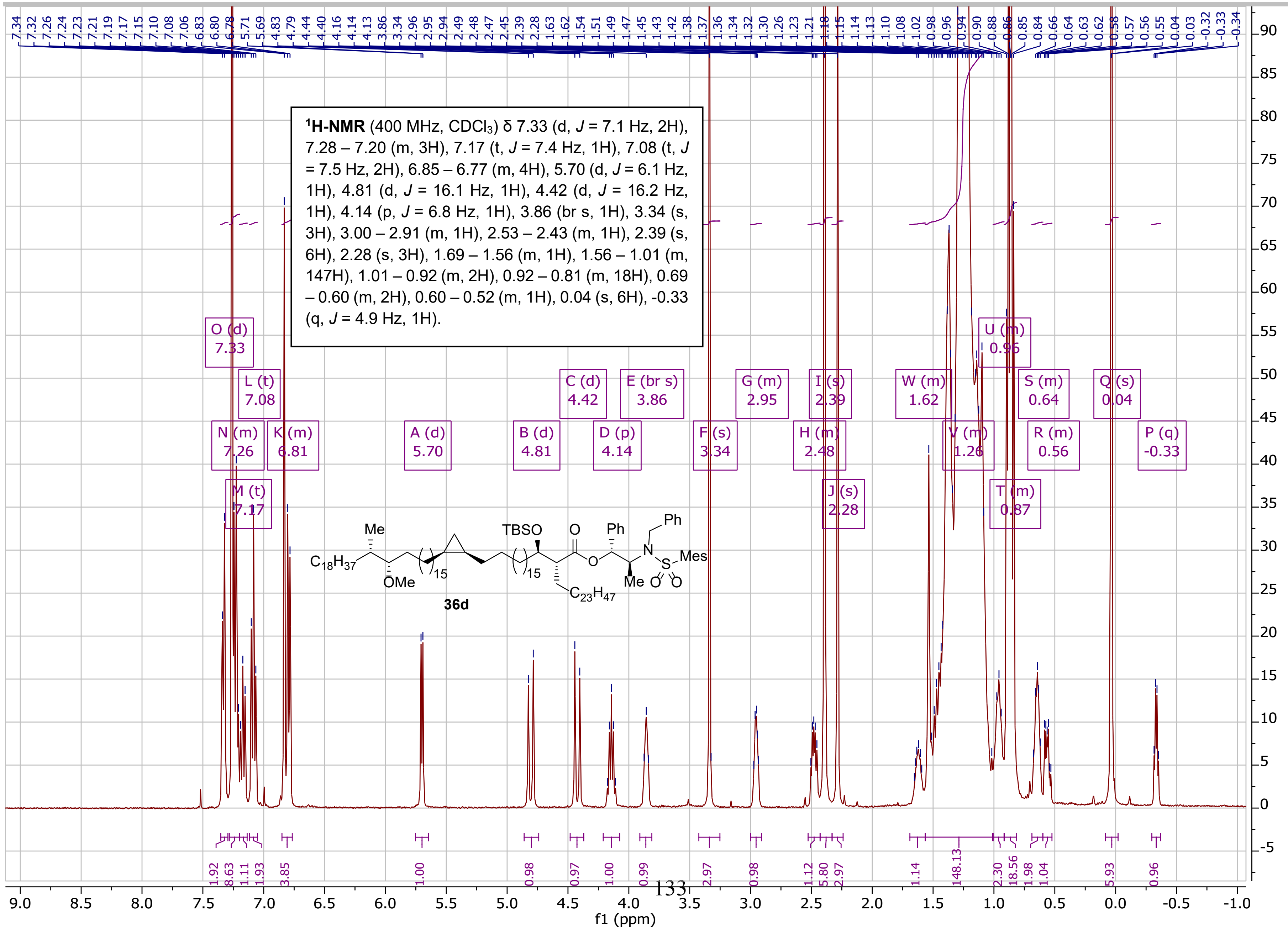
¹H-NMR (400 MHz, CDCl3) δ 7.33 (d, J = 7.1 Hz, 2H), 7.28 – 7.20 (m, 3H), 7.17 (t, J = 7.4 Hz, 1H), 7.08 (t, J = 7.5 Hz, 2H), 6.85 – 6.77 (m, 4H), 5.70 (d, J = 6.1 Hz, 1H), 4.81 (d, J = 16.1 Hz, 1H), 4.42 (d, J = 16.2 Hz, 1H), 4.14 (p, J = 6.8 Hz, 1H), 3.86 (br s, 1H), 3.34 (s, 3H), 3.00 – 2.91 (m, 1H), 2.53 – 2.43 (m, 1H), 2.39 (s, 6H), 2.28 (s, 3H), 1.69 – 1.56 (m, 1H), 1.56 – 1.01 (m, 147H), 1.01 – 0.92 (m, 2H), 0.92 – 0.81 (m, 18H), 0.69 – 0.60 (m, 2H), 0.60 – 0.52 (m, 1H), 0.04 (s, 6H), -0.33 (q, J = 4.9 Hz, 1H).
36d
f1 (ppm)

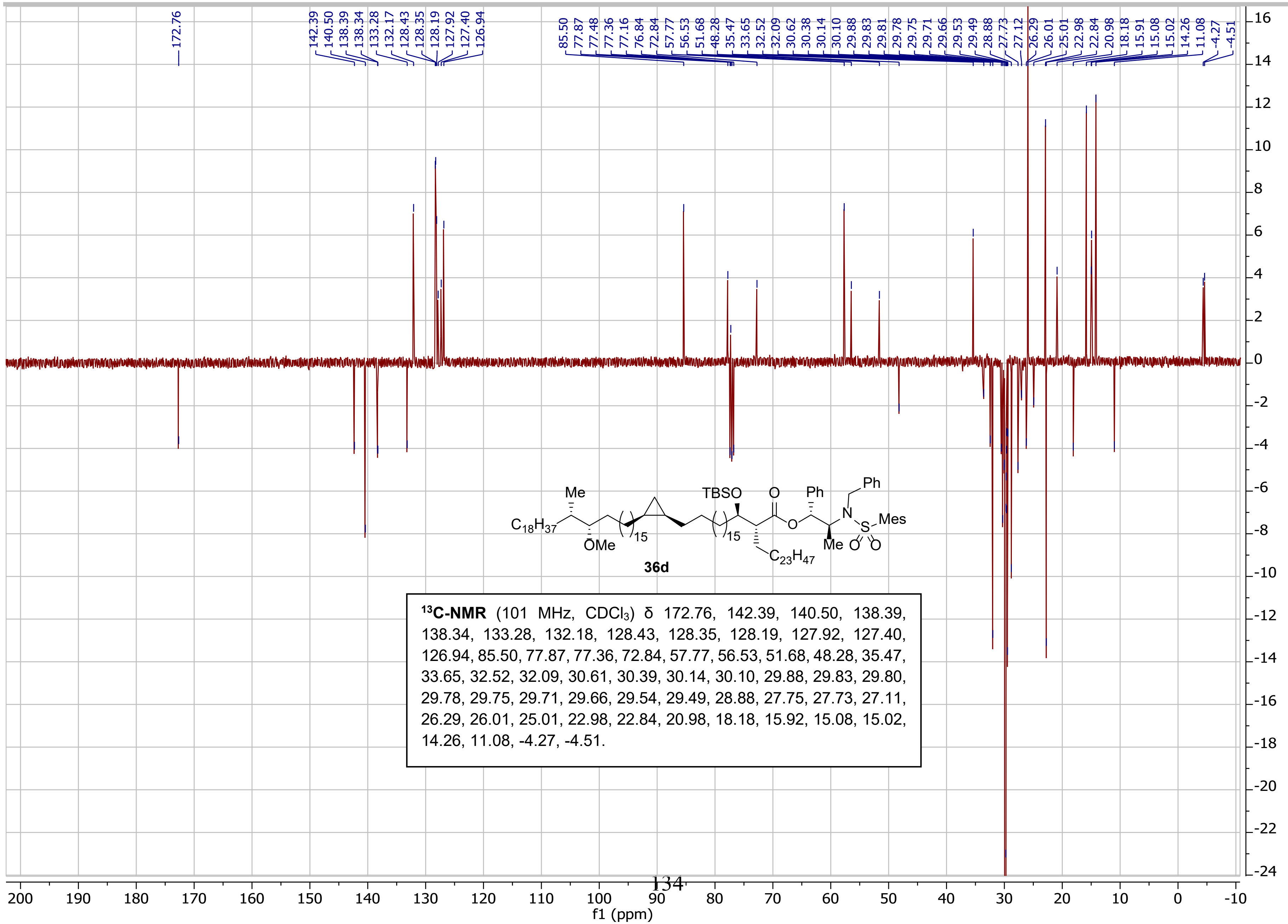


36d

**$^{13}$C-NMR** (101 MHz, $CDCl_3$) δ 172.76, 142.39, 140.50, 138.39, 138.34, 133.28, 132.18, 128.43, 128.35, 128.19, 127.92, 127.40, 126.94, 85.50, 77.87, 77.36, 72.84, 57.77, 56.53, 51.68, 48.28, 35.47, 33.65, 32.52, 32.09, 30.61, 30.39, 30.14, 30.10, 29.88, 29.83, 29.80, 29.78, 29.75, 29.71, 29.66, 29.54, 29.49, 28.88, 27.75, 27.73, 27.11, 26.29, 26.01, 25.01, 22.98, 22.84, 20.98, 18.18, 15.92, 15.08, 15.02, 14.26, 11.08, -4.27, -4.51.

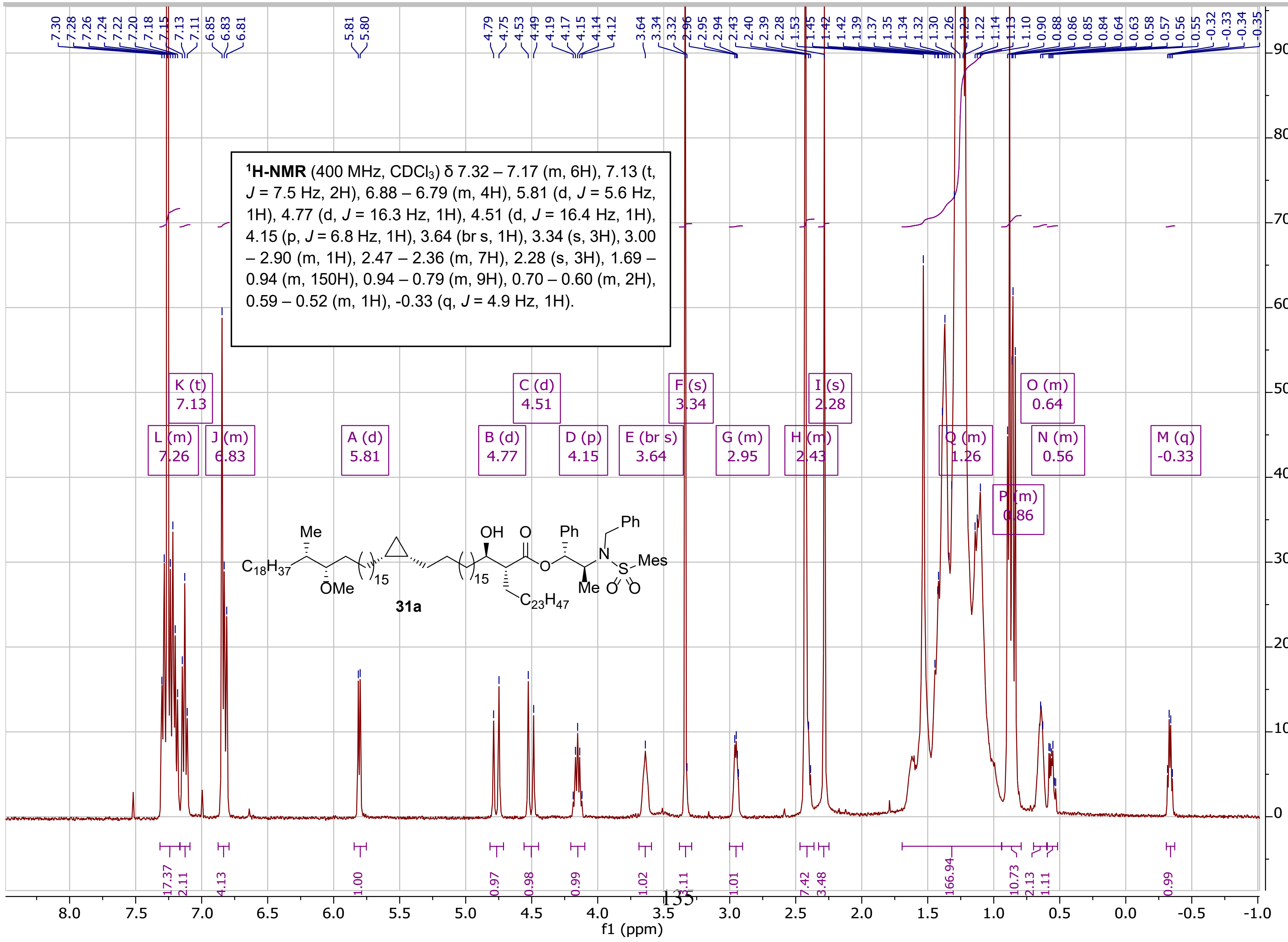
1H-NMR (400 MHz, CDCl3) δ 7.32 – 7.17 (m, 6H), 7.13 (t, J = 7.5 Hz, 2H), 6.88 – 6.79 (m, 4H), 5.81 (d, J = 5.6 Hz, 1H), 4.77 (d, J = 16.3 Hz, 1H), 4.51 (d, J = 16.4 Hz, 1H), 4.15 (p, J = 6.8 Hz, 1H), 3.64 (br s, 1H), 3.34 (s, 3H), 3.00 – 2.90 (m, 1H), 2.47 – 2.36 (m, 7H), 2.28 (s, 3H), 1.69 – 0.94 (m, 150H), 0.94 – 0.79 (m, 9H), 0.70 – 0.60 (m, 2H), 0.59 – 0.52 (m, 1H), -0.33 (q, J = 4.9 Hz, 1H).
K (t) 7.13
C (d) 4.51
F (s) 3.34
I (s) 2.28
O (m) 0.64
L (m) 7.26
J (m) 6.83
A (d) 5.81
B (d) 4.77
D (p) 4.15
E (br s) 3.64
G (m) 2.95
H (m) 2.43
Q (m) 1.26
N (m) 0.56
M (q) -0.33
P (m) 0.86
31a
f1 (ppm)

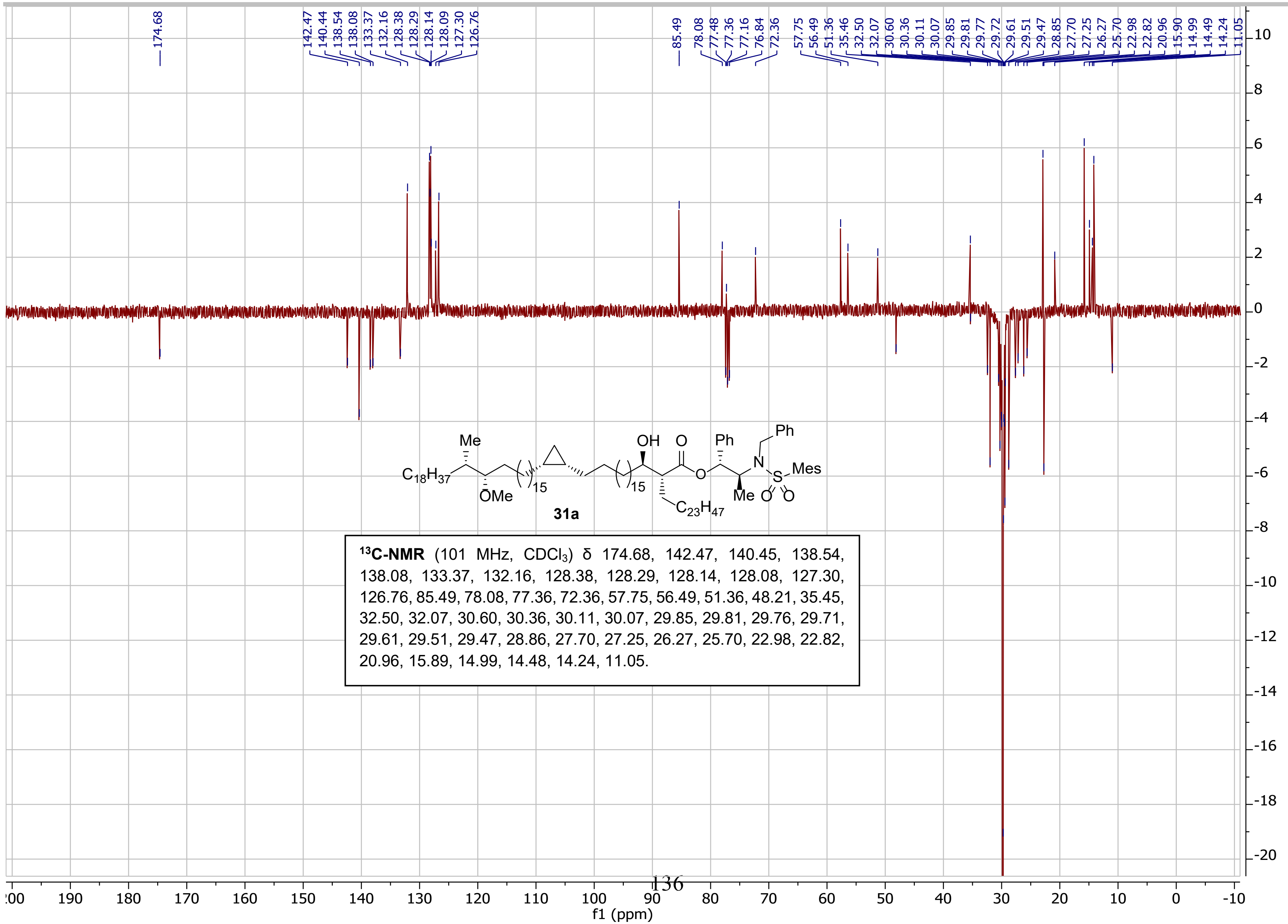

31a

**$^{13}$C-NMR** (101 MHz, $CDCl_3$) δ 174.68, 142.47, 140.45, 138.54, 138.08, 133.37, 132.16, 128.38, 128.29, 128.14, 128.08, 127.30, 126.76, 85.49, 78.08, 77.36, 72.36, 57.75, 56.49, 51.36, 48.21, 35.45, 32.50, 32.07, 30.60, 30.36, 30.11, 30.07, 29.85, 29.81, 29.76, 29.71, 29.61, 29.51, 29.47, 28.86, 27.70, 27.25, 26.27, 25.70, 22.98, 22.82, 20.96, 15.89, 14.99, 14.48, 14.24, 11.05.

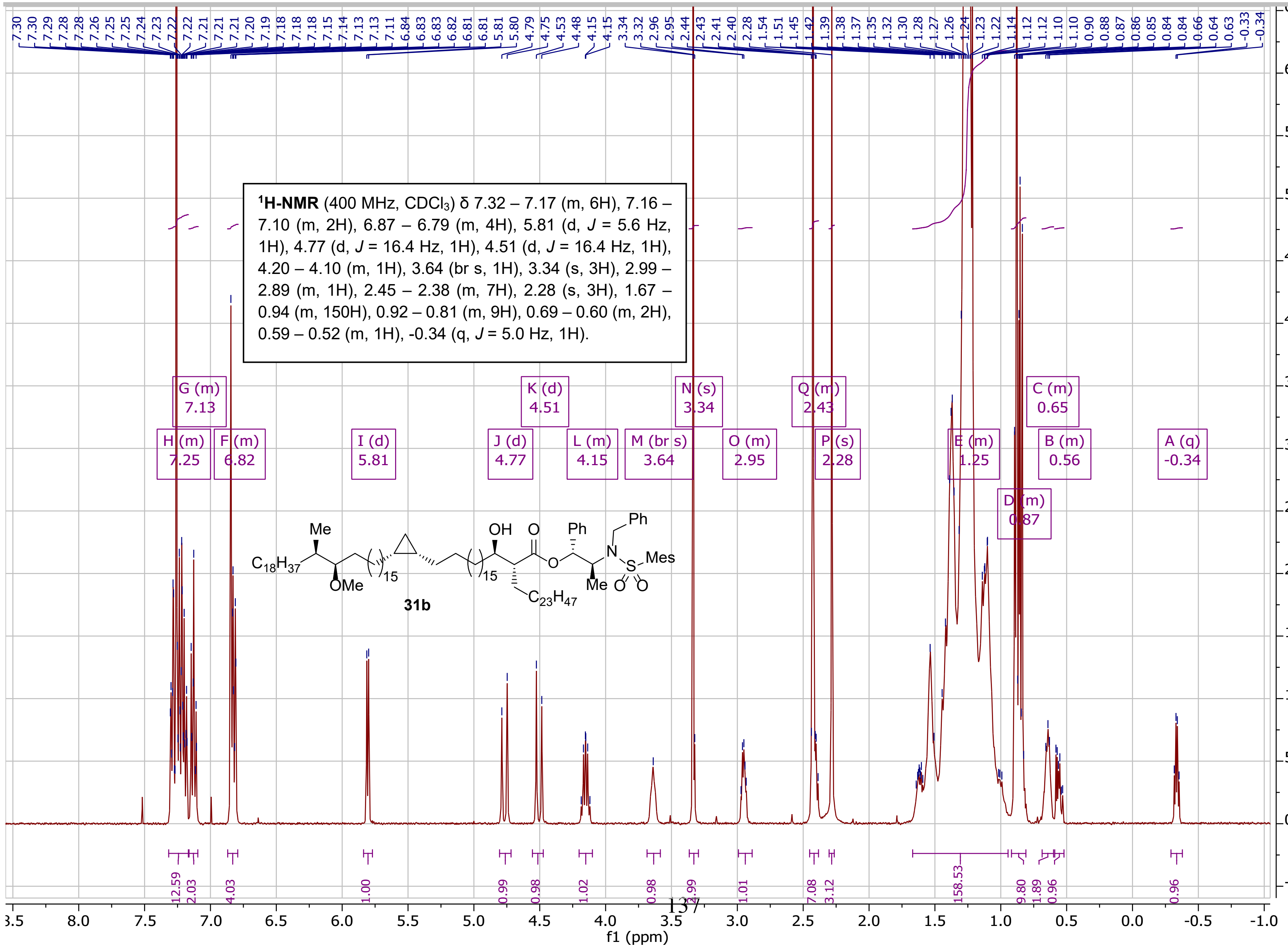
1H-NMR (400 MHz, CDCl3) δ 7.32 – 7.17 (m, 6H), 7.16 – 7.10 (m, 2H), 6.87 – 6.79 (m, 4H), 5.81 (d, J = 5.6 Hz, 1H), 4.77 (d, J = 16.4 Hz, 1H), 4.51 (d, J = 16.4 Hz, 1H), 4.20 – 4.10 (m, 1H), 3.64 (br s, 1H), 3.34 (s, 3H), 2.99 – 2.89 (m, 1H), 2.45 – 2.38 (m, 7H), 2.28 (s, 3H), 1.67 – 0.94 (m, 150H), 0.92 – 0.81 (m, 9H), 0.69 – 0.60 (m, 2H), 0.59 – 0.52 (m, 1H), -0.34 (q, J = 5.0 Hz, 1H).
31b
f1 (ppm)

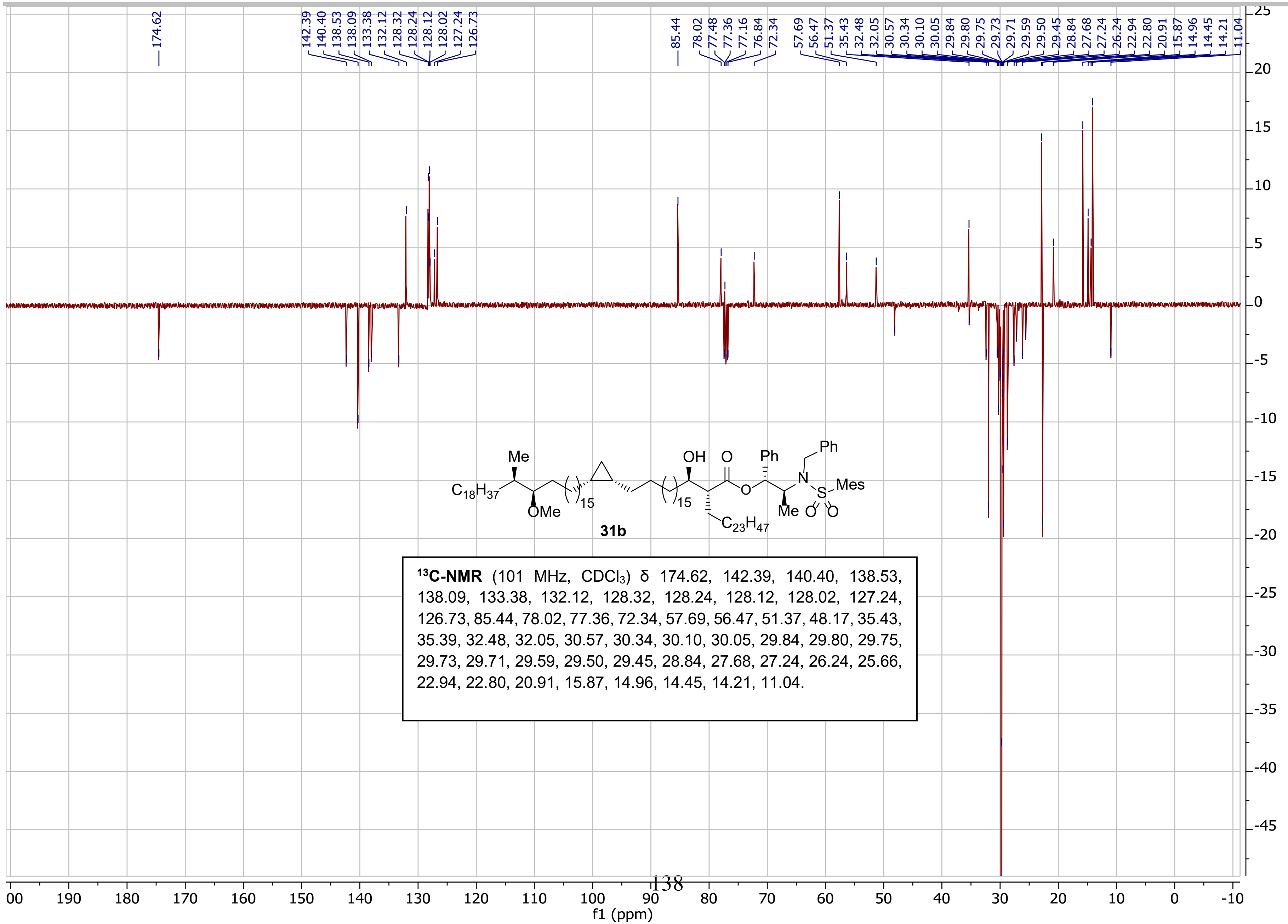


**$^{13}$C-NMR** (101 MHz, $CDCl_3$) δ 174.62, 142.39, 140.40, 138.53, 138.09, 133.38, 132.12, 128.32, 128.24, 128.12, 128.02, 127.24, 126.73, 85.44, 78.02, 77.36, 72.34, 57.69, 56.47, 51.37, 48.17, 35.43, 35.39, 32.48, 32.05, 30.57, 30.34, 30.10, 30.05, 29.84, 29.80, 29.75, 29.73, 29.71, 29.59, 29.50, 29.45, 28.84, 27.68, 27.24, 26.24, 25.66, 22.94, 22.80, 20.91, 15.87, 14.96, 14.45, 14.21, 11.04.

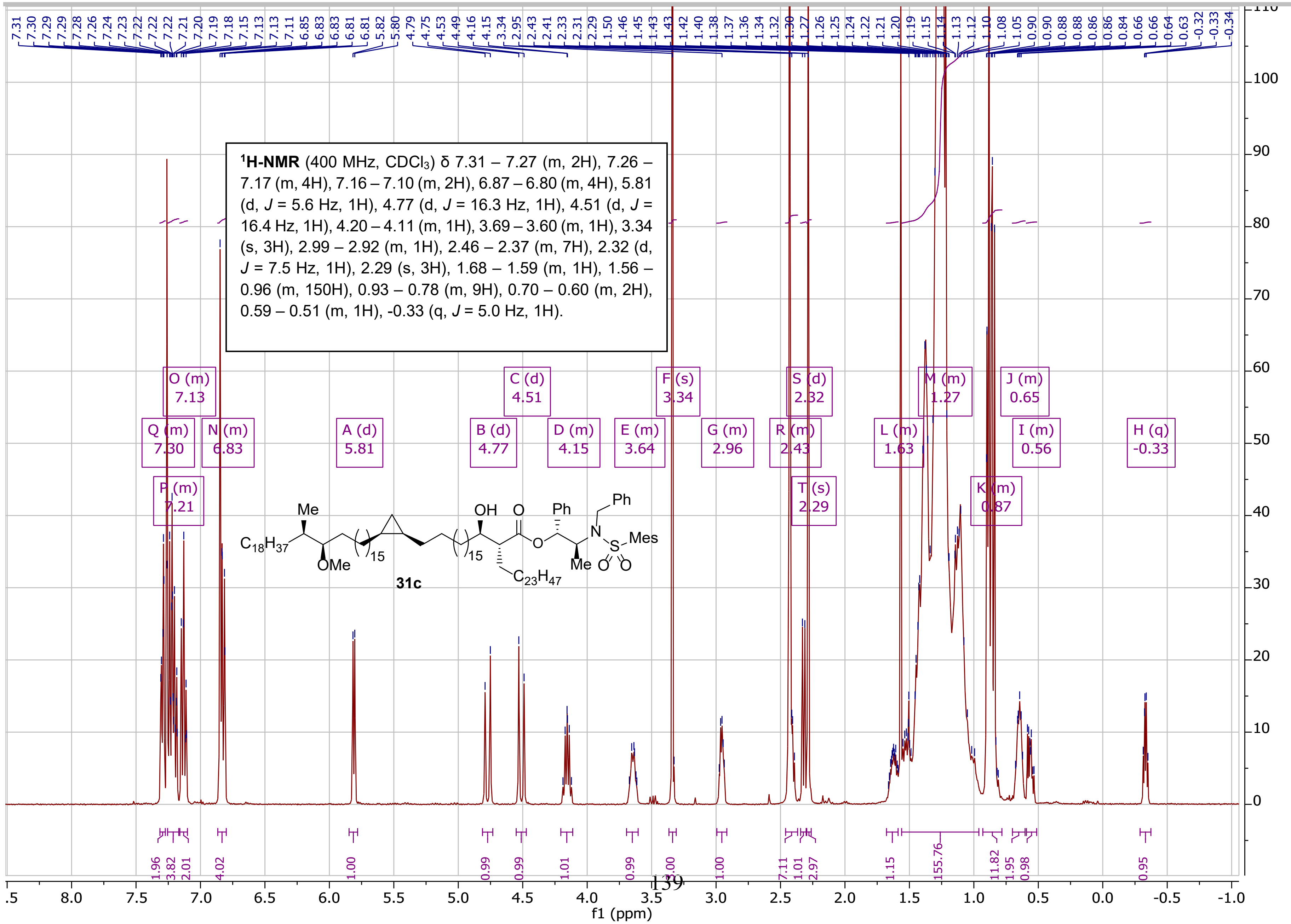
1H-NMR (400 MHz, CDCl3) δ 7.31 – 7.27 (m, 2H), 7.26 – 7.17 (m, 4H), 7.16 – 7.10 (m, 2H), 6.87 – 6.80 (m, 4H), 5.81 (d, J = 5.6 Hz, 1H), 4.77 (d, J = 16.3 Hz, 1H), 4.51 (d, J = 16.4 Hz, 1H), 4.20 – 4.11 (m, 1H), 3.69 – 3.60 (m, 1H), 3.34 (s, 3H), 2.99 – 2.92 (m, 1H), 2.46 – 2.37 (m, 7H), 2.32 (d, J = 7.5 Hz, 1H), 2.29 (s, 3H), 1.68 – 1.59 (m, 1H), 1.56 – 0.96 (m, 150H), 0.93 – 0.78 (m, 9H), 0.70 – 0.60 (m, 2H), 0.59 – 0.51 (m, 1H), -0.33 (q, J = 5.0 Hz, 1H).
31c
f1 (ppm)

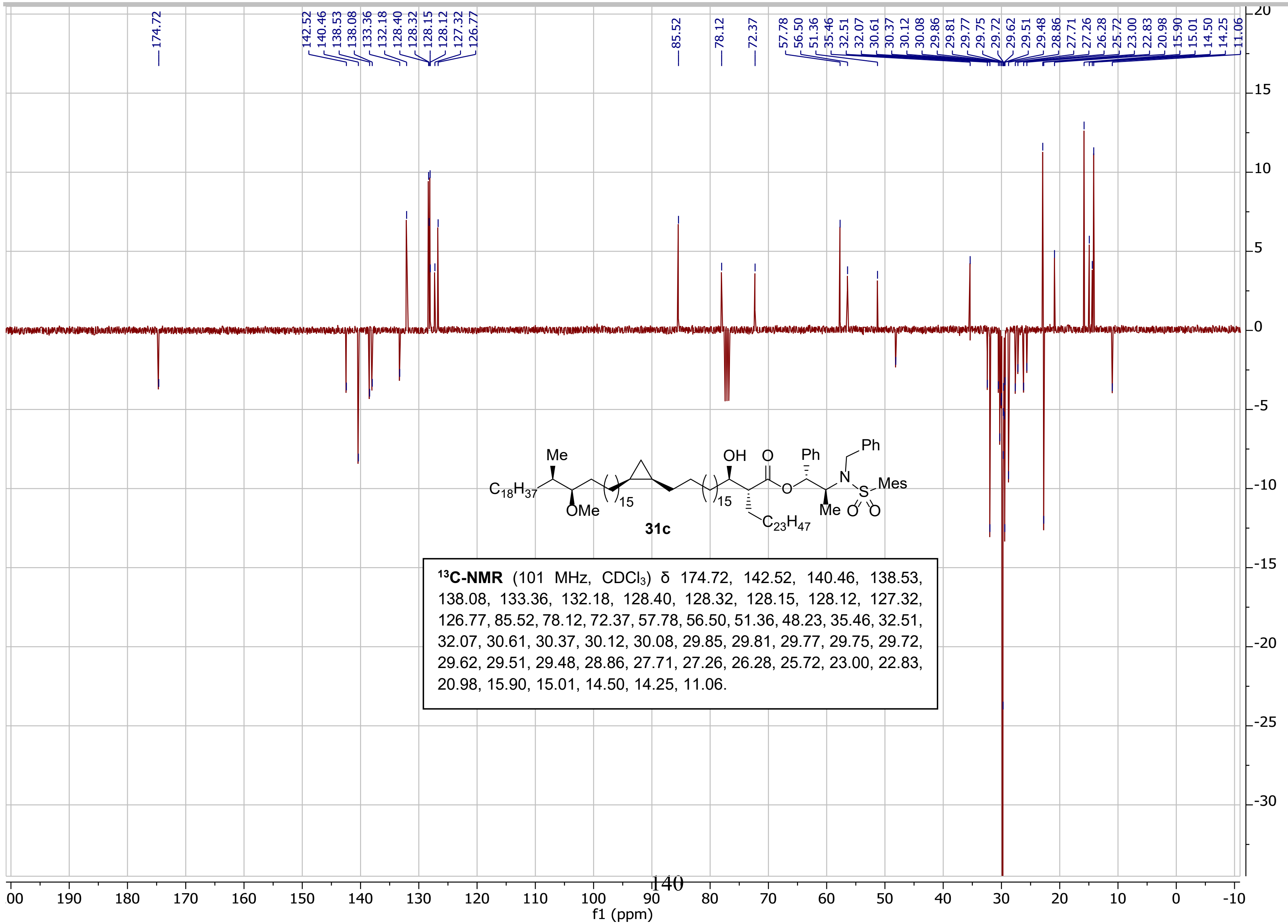


**31c**

**$^{13}$C-NMR** (101 MHz, $CDCl_3$) δ 174.72, 142.52, 140.46, 138.53, 138.08, 133.36, 132.18, 128.40, 128.32, 128.15, 128.12, 127.32, 126.77, 85.52, 78.12, 72.37, 57.78, 56.50, 51.36, 48.23, 35.46, 32.51, 32.07, 30.61, 30.37, 30.12, 30.08, 29.85, 29.81, 29.77, 29.75, 29.72, 29.62, 29.51, 29.48, 28.86, 27.71, 27.26, 26.28, 25.72, 23.00, 22.83, 20.98, 15.90, 15.01, 14.50, 14.25, 11.06.

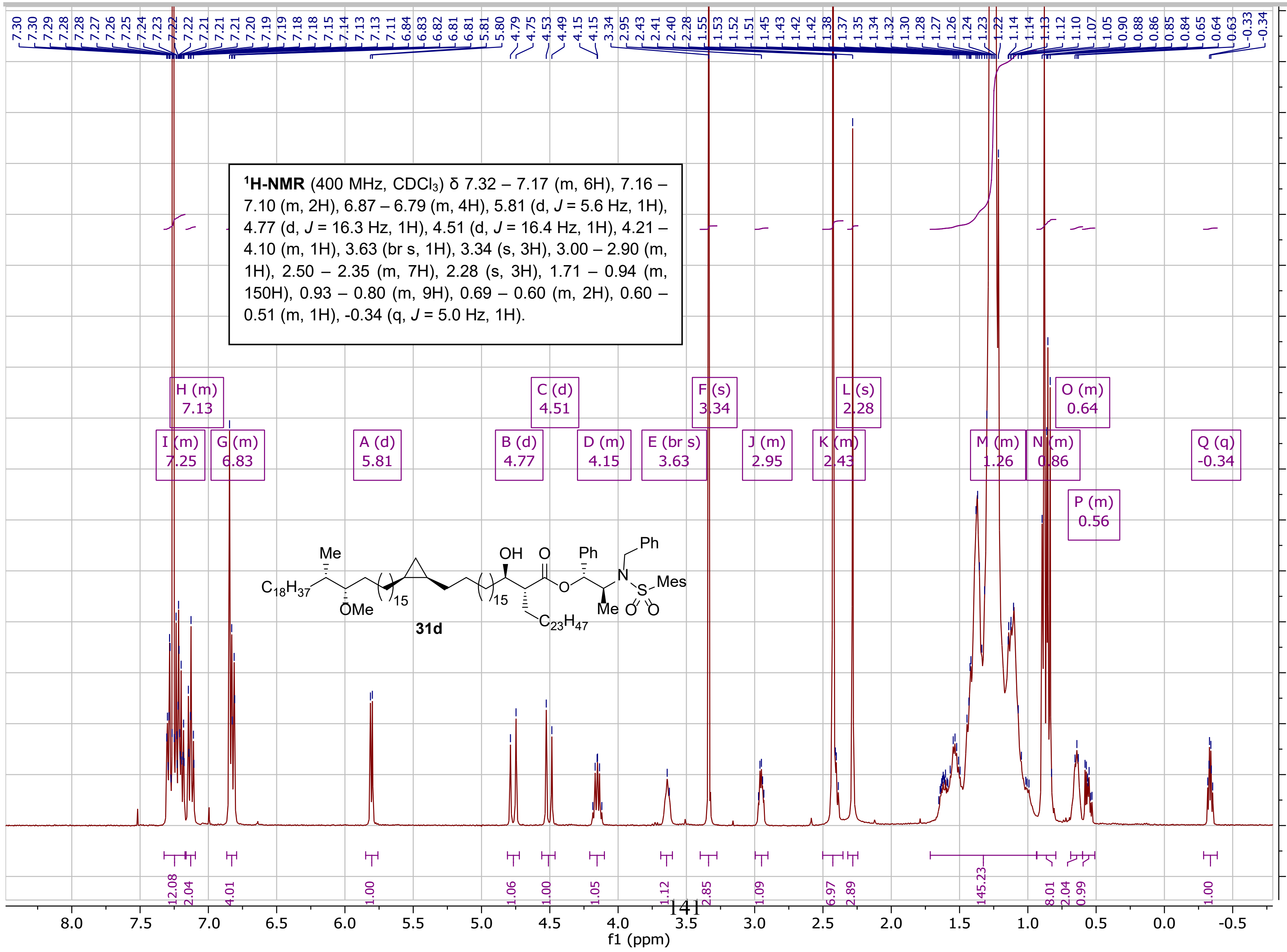

1H-NMR (400 MHz, CDCl3) δ 7.32 – 7.17 (m, 6H), 7.16 – 7.10 (m, 2H), 6.87 – 6.79 (m, 4H), 5.81 (d, J = 5.6 Hz, 1H), 4.77 (d, J = 16.3 Hz, 1H), 4.51 (d, J = 16.4 Hz, 1H), 4.21 – 4.10 (m, 1H), 3.63 (br s, 1H), 3.34 (s, 3H), 3.00 – 2.90 (m, 1H), 2.50 – 2.35 (m, 7H), 2.28 (s, 3H), 1.71 – 0.94 (m, 150H), 0.93 – 0.80 (m, 9H), 0.69 – 0.60 (m, 2H), 0.60 – 0.51 (m, 1H), -0.34 (q, J = 5.0 Hz, 1H).
H (m) 7.13
I (m) 7.25
G (m) 6.83
A (d) 5.81
B (d) 4.77
C (d) 4.51
D (m) 4.15
E (br s) 3.63
F (s) 3.34
J (m) 2.95
K (m) 2.43
L (s) 2.28
M (m) 1.26
N (m) 0.86
O (m) 0.64
P (m) 0.56
Q (q) -0.34
Me
C18H37
OMe
15
OH
O
Ph
N
S
Mes
Me
C23H47
31d
f1 (ppm)

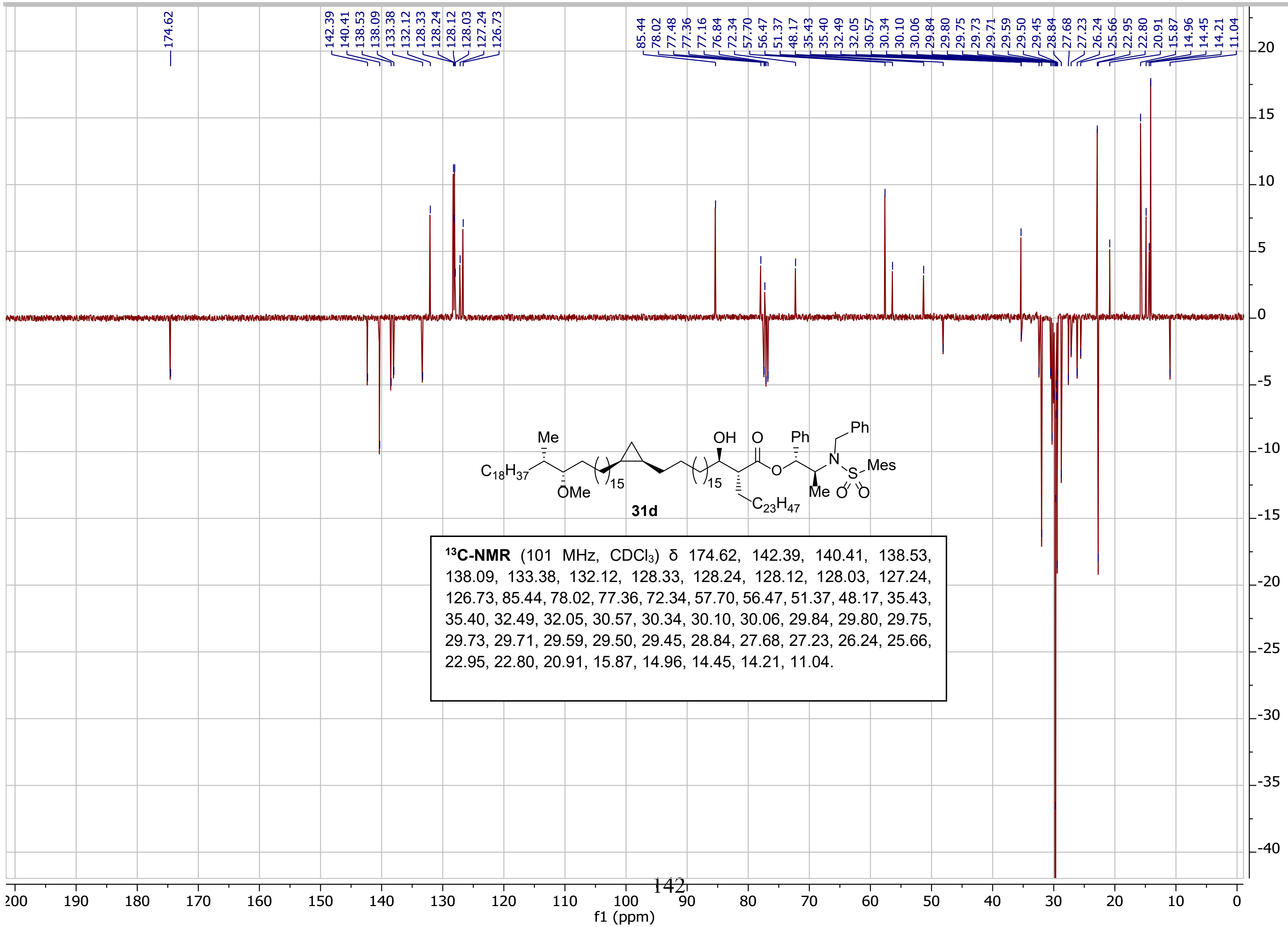


**$^{13}$C-NMR** (101 MHz, $CDCl_3$) δ 174.62, 142.39, 140.41, 138.53, 138.09, 133.38, 132.12, 128.33, 128.24, 128.12, 128.03, 127.24, 126.73, 85.44, 78.02, 77.36, 72.34, 57.70, 56.47, 51.37, 48.17, 35.43, 35.40, 32.49, 32.05, 30.57, 30.34, 30.10, 30.06, 29.84, 29.80, 29.75, 29.73, 29.71, 29.59, 29.50, 29.45, 28.84, 27.68, 27.23, 26.24, 25.66, 22.95, 22.80, 20.91, 15.87, 14.96, 14.45, 14.21, 11.04.

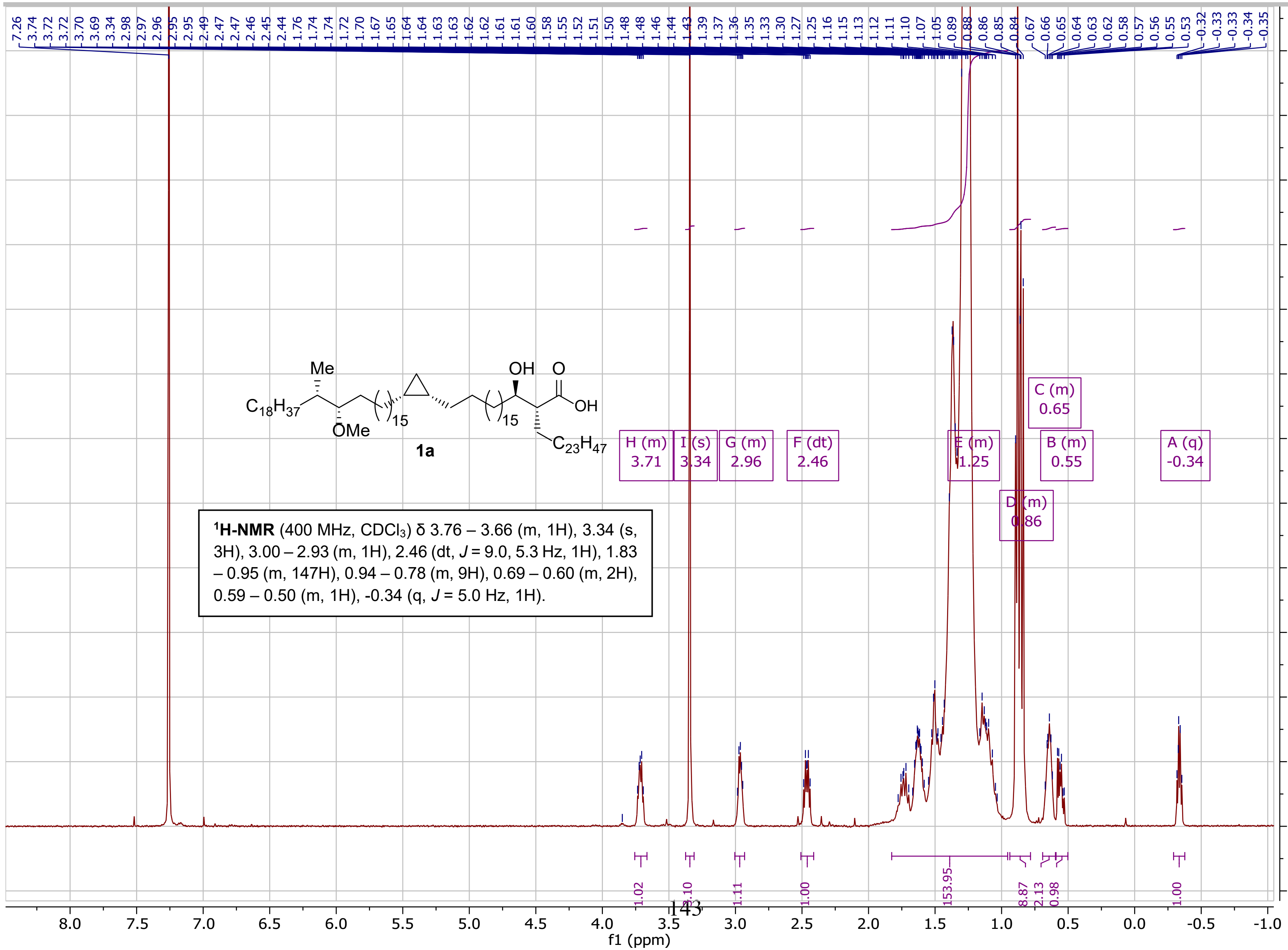

**$^1$H-NMR** (400 MHz, $CDCl_3$) δ 3.76 – 3.66 (m, 1H), 3.34 (s, 3H), 3.00 – 2.93 (m, 1H), 2.46 (dt, *J* = 9.0, 5.3 Hz, 1H), 1.83 – 0.95 (m, 147H), 0.94 – 0.78 (m, 9H), 0.69 – 0.60 (m, 2H), 0.59 – 0.50 (m, 1H), -0.34 (q, *J* = 5.0 Hz, 1H).

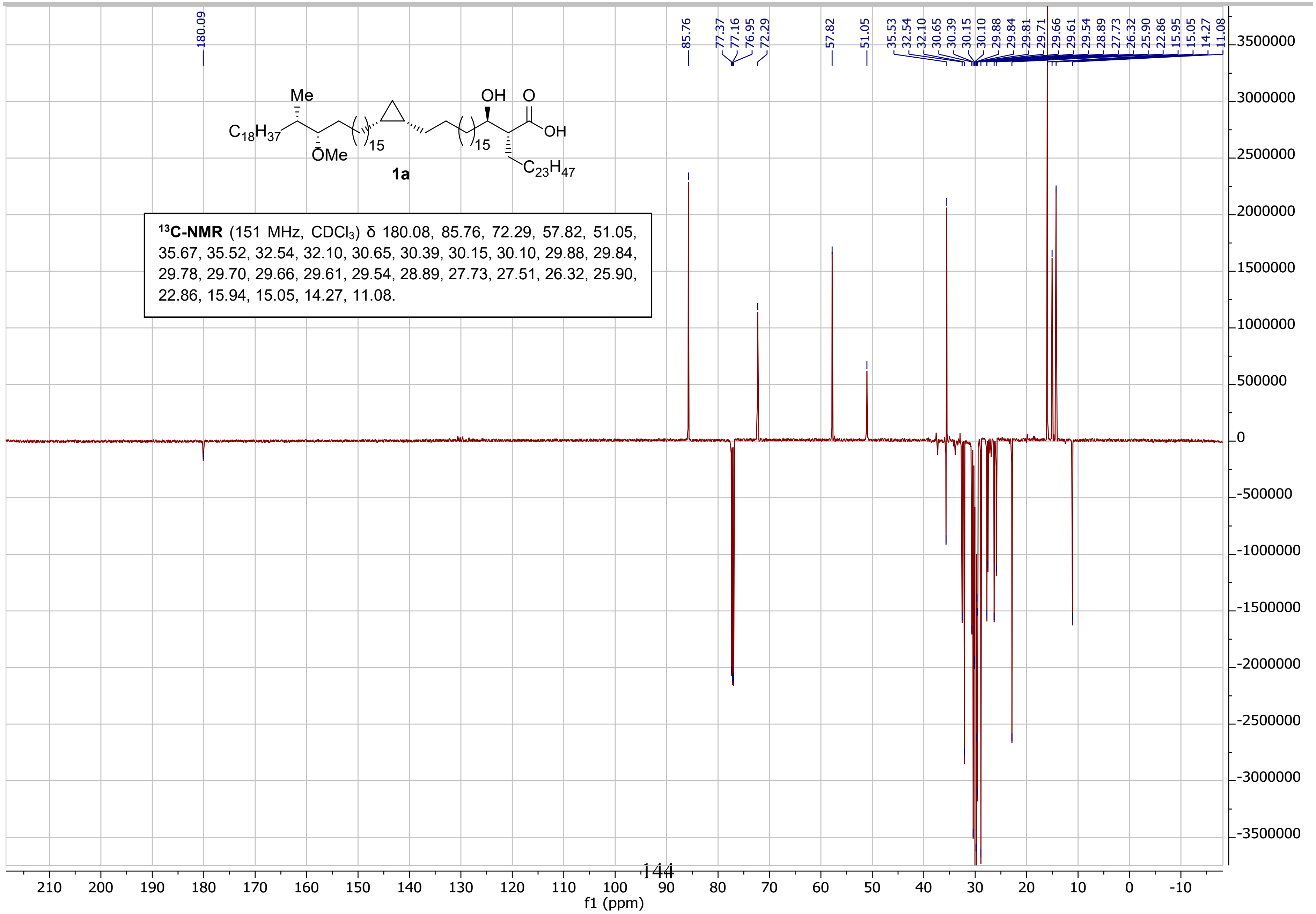


$^{13}$**C-NMR** (151 MHz, $CDCl_3$) δ 180.08, 85.76, 72.29, 57.82, 51.05, 35.67, 35.52, 32.54, 32.10, 30.65, 30.39, 30.15, 30.10, 29.88, 29.84, 29.78, 29.70, 29.66, 29.61, 29.54, 28.89, 27.73, 27.51, 26.32, 25.90, 22.86, 15.94, 15.05, 14.27, 11.08.

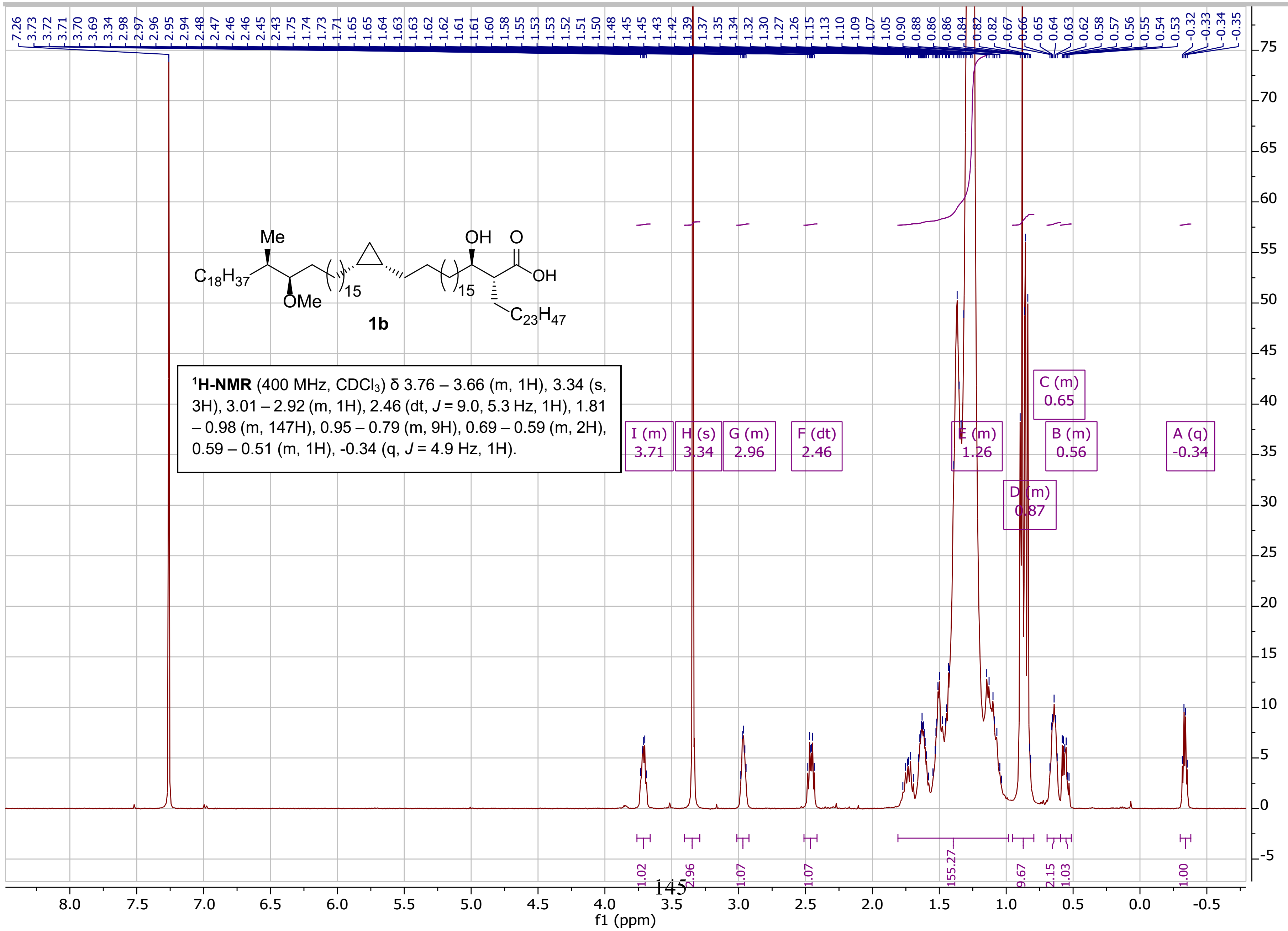
Me
OH O
$C_{18}H_{37}$
OMe
15
15
OH
$C_{23}H_{47}$
1b
$^1$H-NMR (400 MHz, $CDCl_3$) δ 3.76 – 3.66 (m, 1H), 3.34 (s, 3H), 3.01 – 2.92 (m, 1H), 2.46 (dt, $J$ = 9.0, 5.3 Hz, 1H), 1.81 – 0.98 (m, 147H), 0.95 – 0.79 (m, 9H), 0.69 – 0.59 (m, 2H), 0.59 – 0.51 (m, 1H), -0.34 (q, $J$ = 4.9 Hz, 1H).
I (m) 3.71
H (s) 3.34
G (m) 2.96
F (dt) 2.46
E (m) 1.26
D (m) 0.87
C (m) 0.65
B (m) 0.56
A (q) -0.34
1.02
2.96
1.07
1.07
155.27
9.67
2.15
1.03
1.00
f1 (ppm)

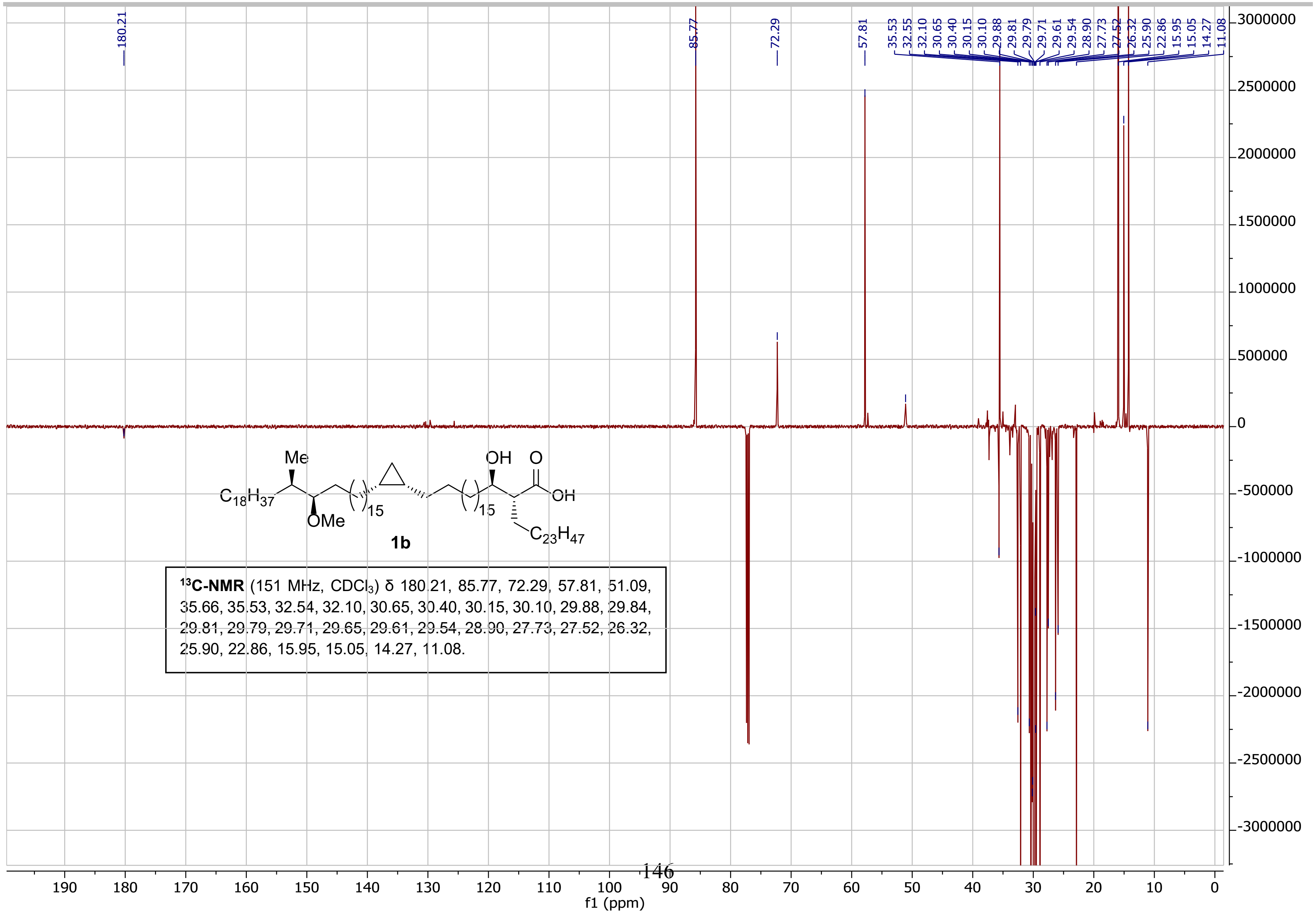
180.21
85.77
72.29
57.81
35.53
32.55
32.10
30.65
30.40
30.15
30.10
29.88
29.81
29.79
29.71
29.61
29.54
28.90
27.73
27.52
26.32
25.90
22.86
15.95
15.05
14.27
11.08
Me
OH O
$C_{18}H_{37}$
OMe
15
15
OH
$C_{23}H_{47}$
1b
**$^{13}$C-NMR** (151 MHz, $CDCl_3$) δ 180.21, 85.77, 72.29, 57.81, 51.09, 35.66, 35.53, 32.54, 32.10, 30.65, 30.40, 30.15, 30.10, 29.88, 29.84, 29.81, 29.79, 29.71, 29.65, 29.61, 29.54, 28.90, 27.73, 27.52, 26.32, 25.90, 22.86, 15.95, 15.05, 14.27, 11.08.
f1 (ppm)

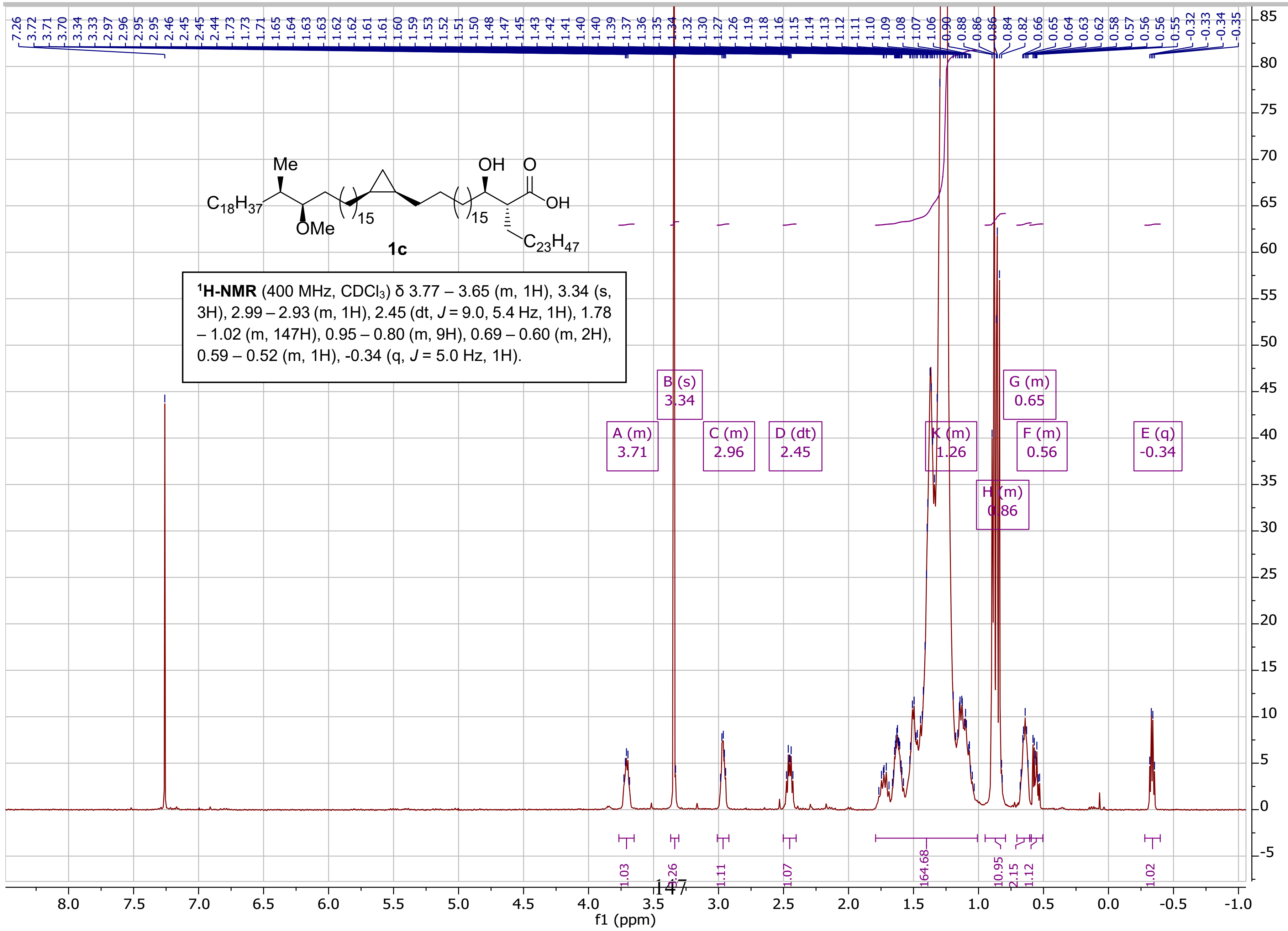
Me
OH
O
$C_{18}H_{37}$
OH
15
15
OMe
$C_{23}H_{47}$
1c
**$^{1}$H-NMR** (400 MHz, $CDCl_3$) δ 3.77 – 3.65 (m, 1H), 3.34 (s, 3H), 2.99 – 2.93 (m, 1H), 2.45 (dt, *J* = 9.0, 5.4 Hz, 1H), 1.78 – 1.02 (m, 147H), 0.95 – 0.80 (m, 9H), 0.69 – 0.60 (m, 2H), 0.59 – 0.52 (m, 1H), -0.34 (q, *J* = 5.0 Hz, 1H).
A (m) 3.71
B (s) 3.34
C (m) 2.96
D (dt) 2.45
K (m) 1.26
H (m) 0.86
G (m) 0.65
F (m) 0.56
E (q) -0.34
1.03
3.26
1.11
1.07
164.68
10.95
2.15
1.12
1.02
f1 (ppm)

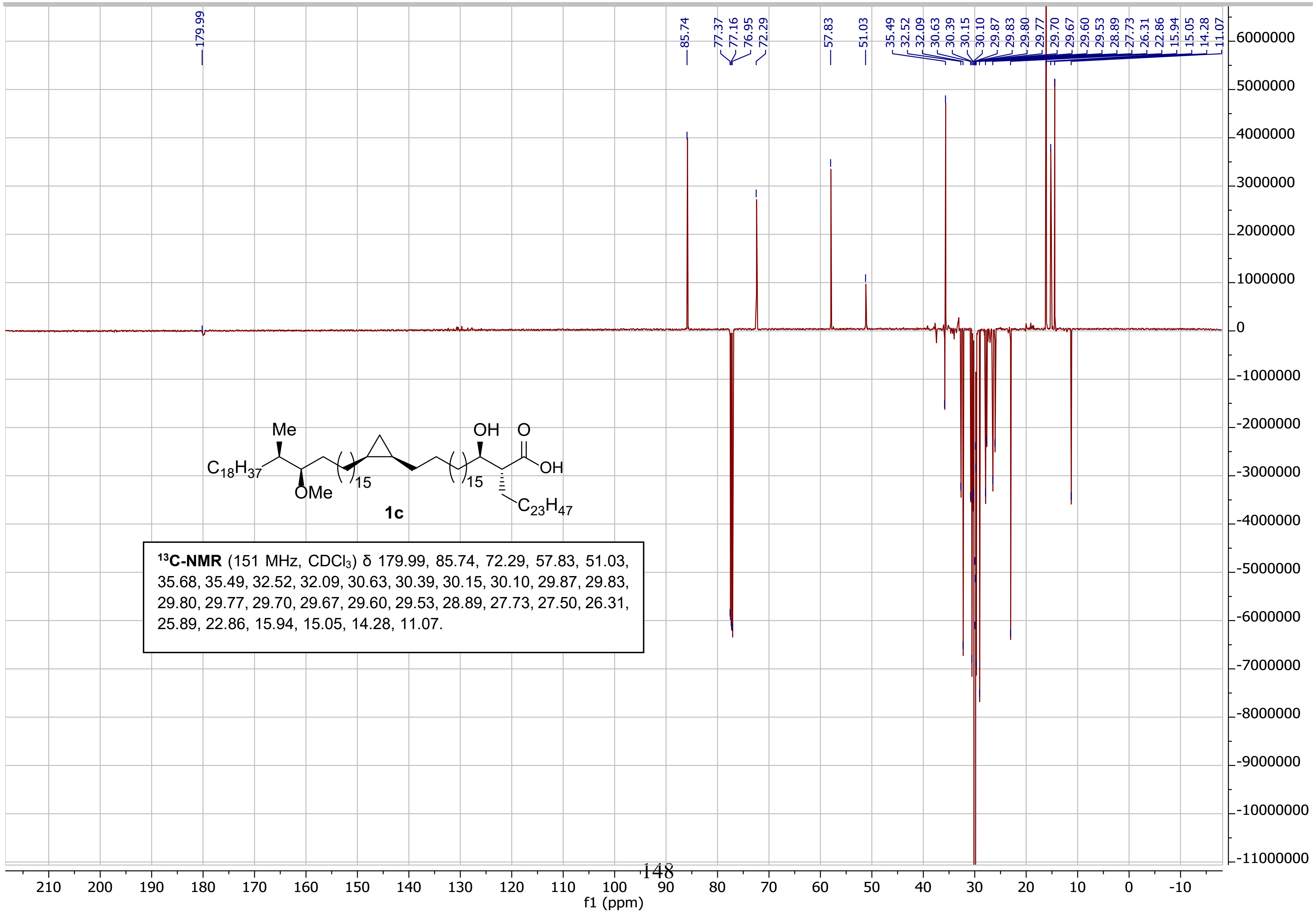


**$^{13}$C-NMR** (151 MHz, $CDCl_3$) δ 179.99, 85.74, 72.29, 57.83, 51.03, 35.68, 35.49, 32.52, 32.09, 30.63, 30.39, 30.15, 30.10, 29.87, 29.83, 29.80, 29.77, 29.70, 29.67, 29.60, 29.53, 28.89, 27.73, 27.50, 26.31, 25.89, 22.86, 15.94, 15.05, 14.28, 11.07.

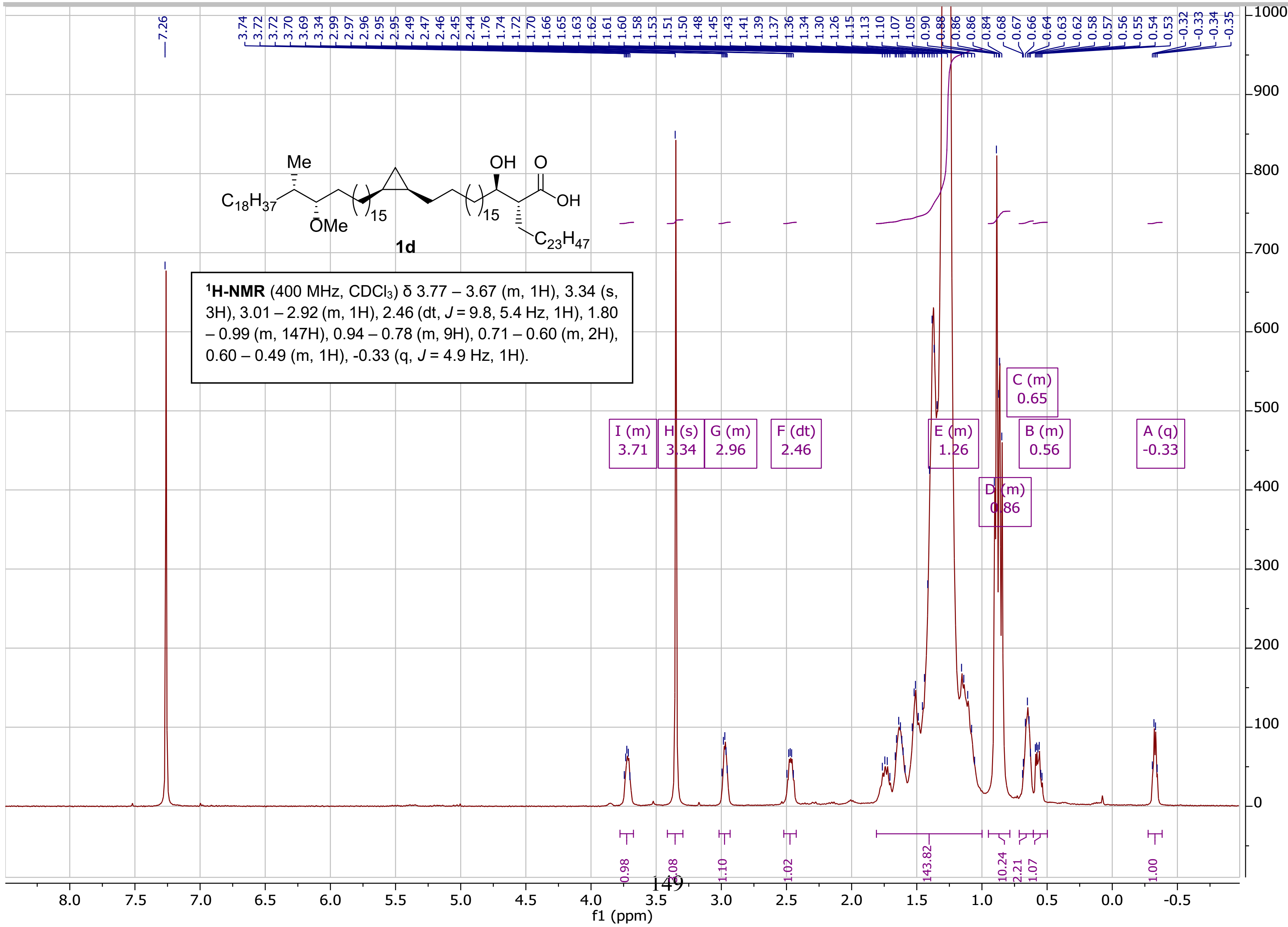


**$^1$H-NMR** (400 MHz, $CDCl_3$) δ 3.77 – 3.67 (m, 1H), 3.34 (s, 3H), 3.01 – 2.92 (m, 1H), 2.46 (dt, *J* = 9.8, 5.4 Hz, 1H), 1.80 – 0.99 (m, 147H), 0.94 – 0.78 (m, 9H), 0.71 – 0.60 (m, 2H), 0.60 – 0.49 (m, 1H), -0.33 (q, *J* = 4.9 Hz, 1H).

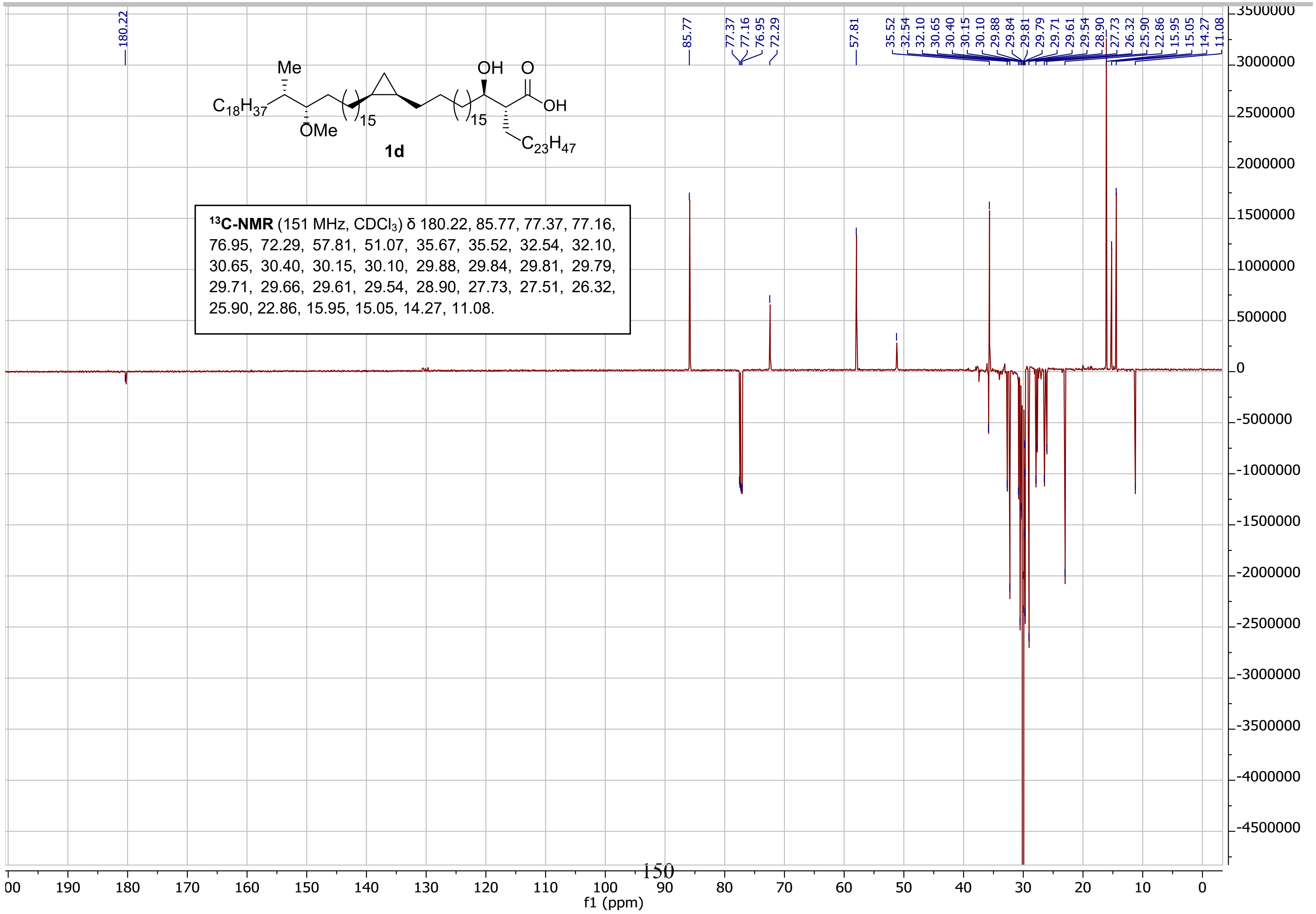


**$^{13}$C-NMR** (151 MHz, $CDCl_3$) δ 180.22, 85.77, 77.37, 77.16, 76.95, 72.29, 57.81, 51.07, 35.67, 35.52, 32.54, 32.10, 30.65, 30.40, 30.15, 30.10, 29.88, 29.84, 29.81, 29.79, 29.71, 29.66, 29.61, 29.54, 28.90, 27.73, 27.51, 26.32, 25.90, 22.86, 15.95, 15.05, 14.27, 11.08.

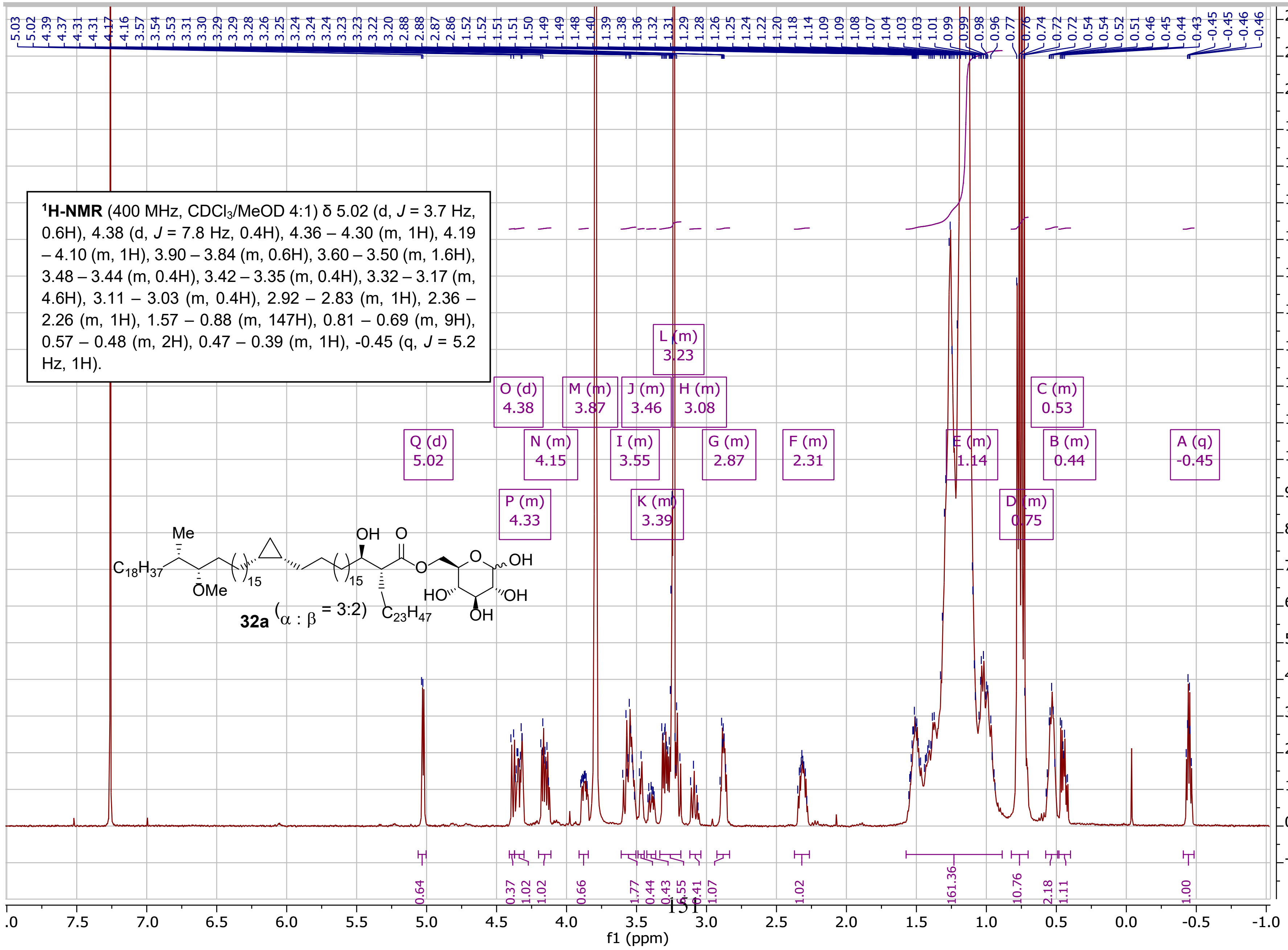


**$^{1}$H-NMR** (400 MHz, $CDCl_3$/MeOD 4:1) δ 5.02 (d, *J* = 3.7 Hz, 0.6H), 4.38 (d, *J* = 7.8 Hz, 0.4H), 4.36 – 4.30 (m, 1H), 4.19 – 4.10 (m, 1H), 3.90 – 3.84 (m, 0.6H), 3.60 – 3.50 (m, 1.6H), 3.48 – 3.44 (m, 0.4H), 3.42 – 3.35 (m, 0.4H), 3.32 – 3.17 (m, 4.6H), 3.11 – 3.03 (m, 0.4H), 2.92 – 2.83 (m, 1H), 2.36 – 2.26 (m, 1H), 1.57 – 0.88 (m, 147H), 0.81 – 0.69 (m, 9H), 0.57 – 0.48 (m, 2H), 0.47 – 0.39 (m, 1H), -0.45 (q, *J* = 5.2 Hz, 1H).

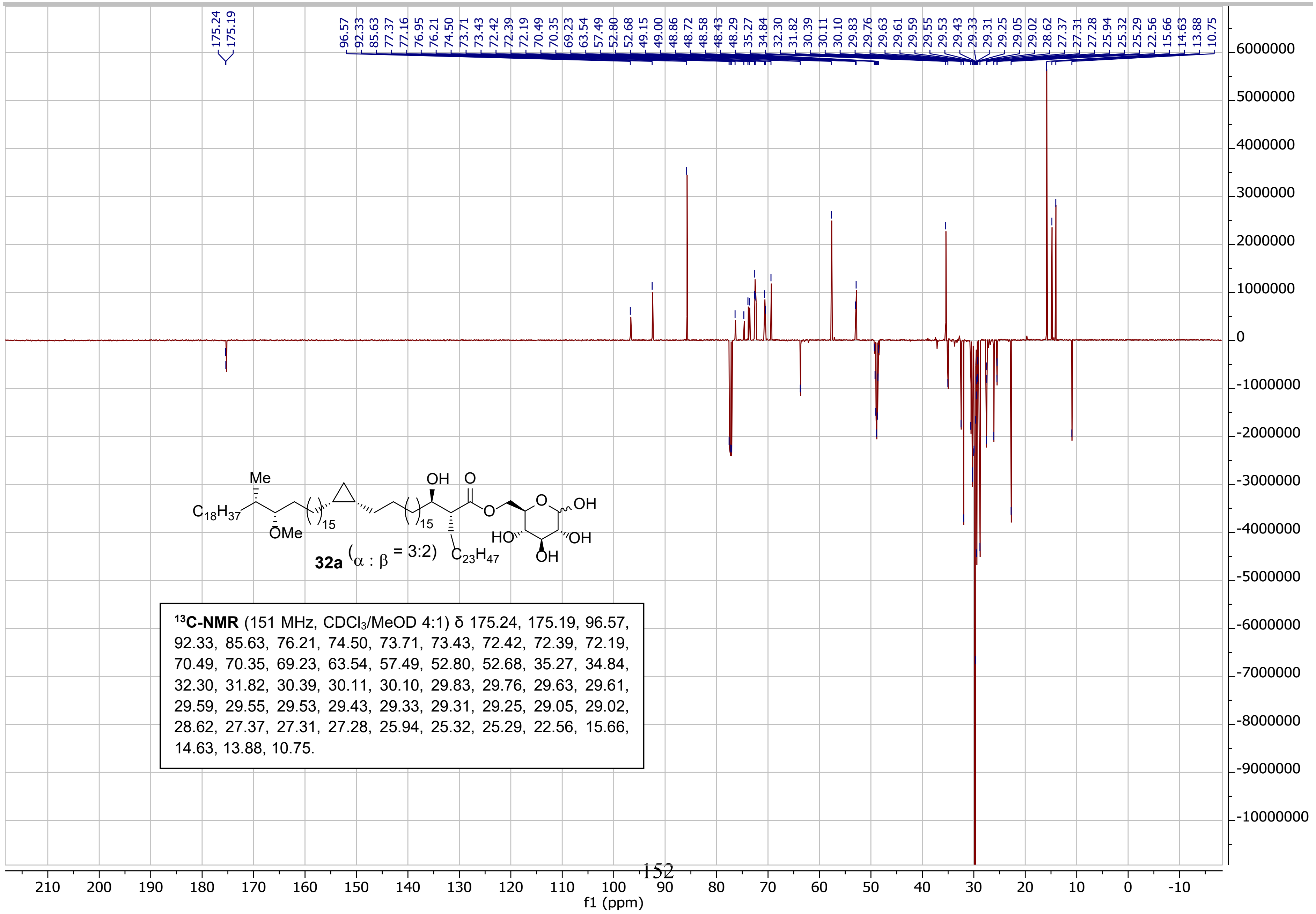


**$^{13}$C-NMR** (151 MHz, $CDCl_3$/MeOD 4:1) δ 175.24, 175.19, 96.57, 92.33, 85.63, 76.21, 74.50, 73.71, 73.43, 72.42, 72.39, 72.19, 70.49, 70.35, 69.23, 63.54, 57.49, 52.80, 52.68, 35.27, 34.84, 32.30, 31.82, 30.39, 30.11, 30.10, 29.83, 29.76, 29.63, 29.61, 29.59, 29.55, 29.53, 29.43, 29.33, 29.31, 29.25, 29.05, 29.02, 28.62, 27.37, 27.31, 27.28, 25.94, 25.32, 25.29, 22.56, 15.66, 14.63, 13.88, 10.75.

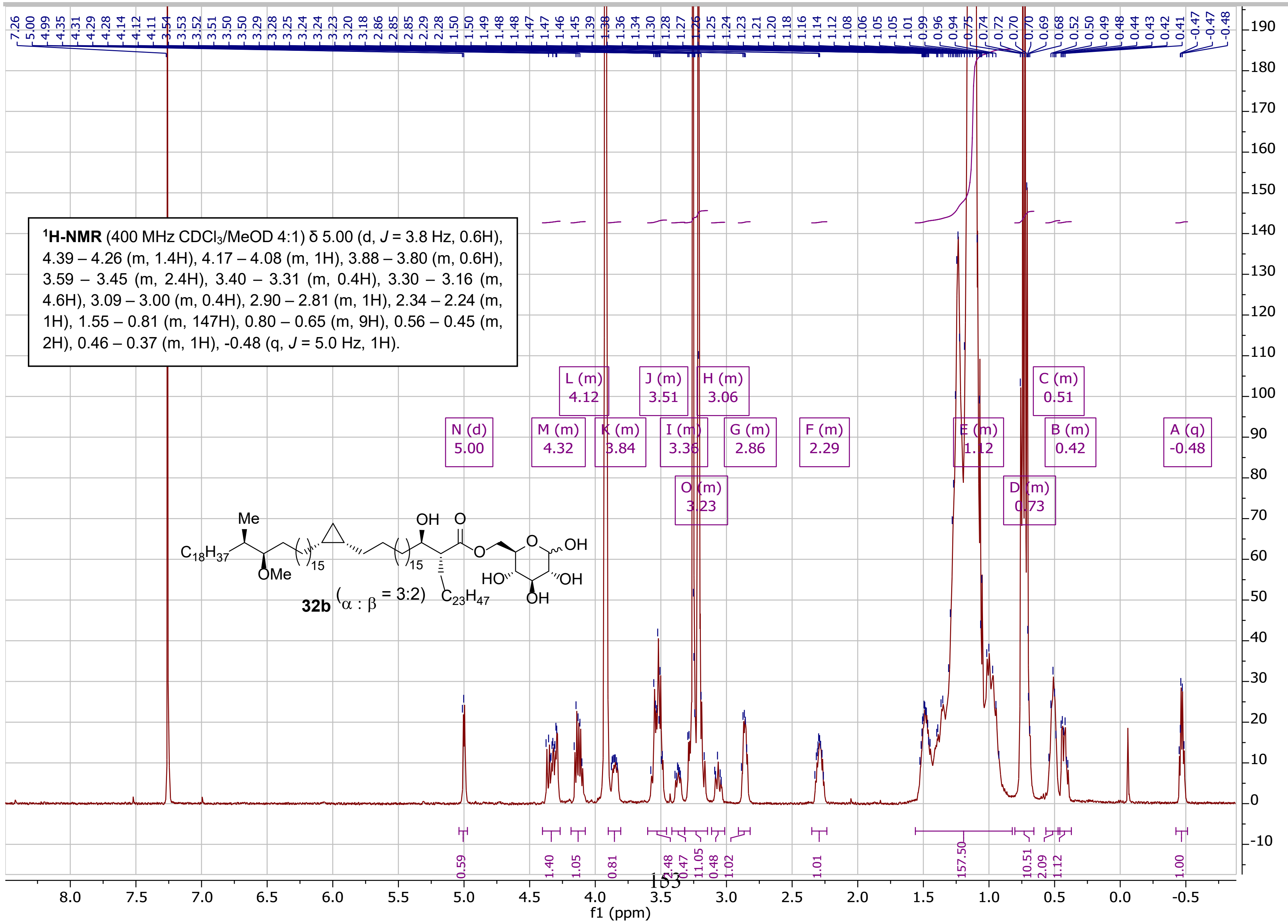


**$^1$H-NMR** (400 MHz $CDCl_3$/MeOD 4:1) δ 5.00 (d, *J* = 3.8 Hz, 0.6H), 4.39 – 4.26 (m, 1.4H), 4.17 – 4.08 (m, 1H), 3.88 – 3.80 (m, 0.6H), 3.59 – 3.45 (m, 2.4H), 3.40 – 3.31 (m, 0.4H), 3.30 – 3.16 (m, 4.6H), 3.09 – 3.00 (m, 0.4H), 2.90 – 2.81 (m, 1H), 2.34 – 2.24 (m, 1H), 1.55 – 0.81 (m, 147H), 0.80 – 0.65 (m, 9H), 0.56 – 0.45 (m, 2H), 0.46 – 0.37 (m, 1H), -0.48 (q, *J* = 5.0 Hz, 1H).

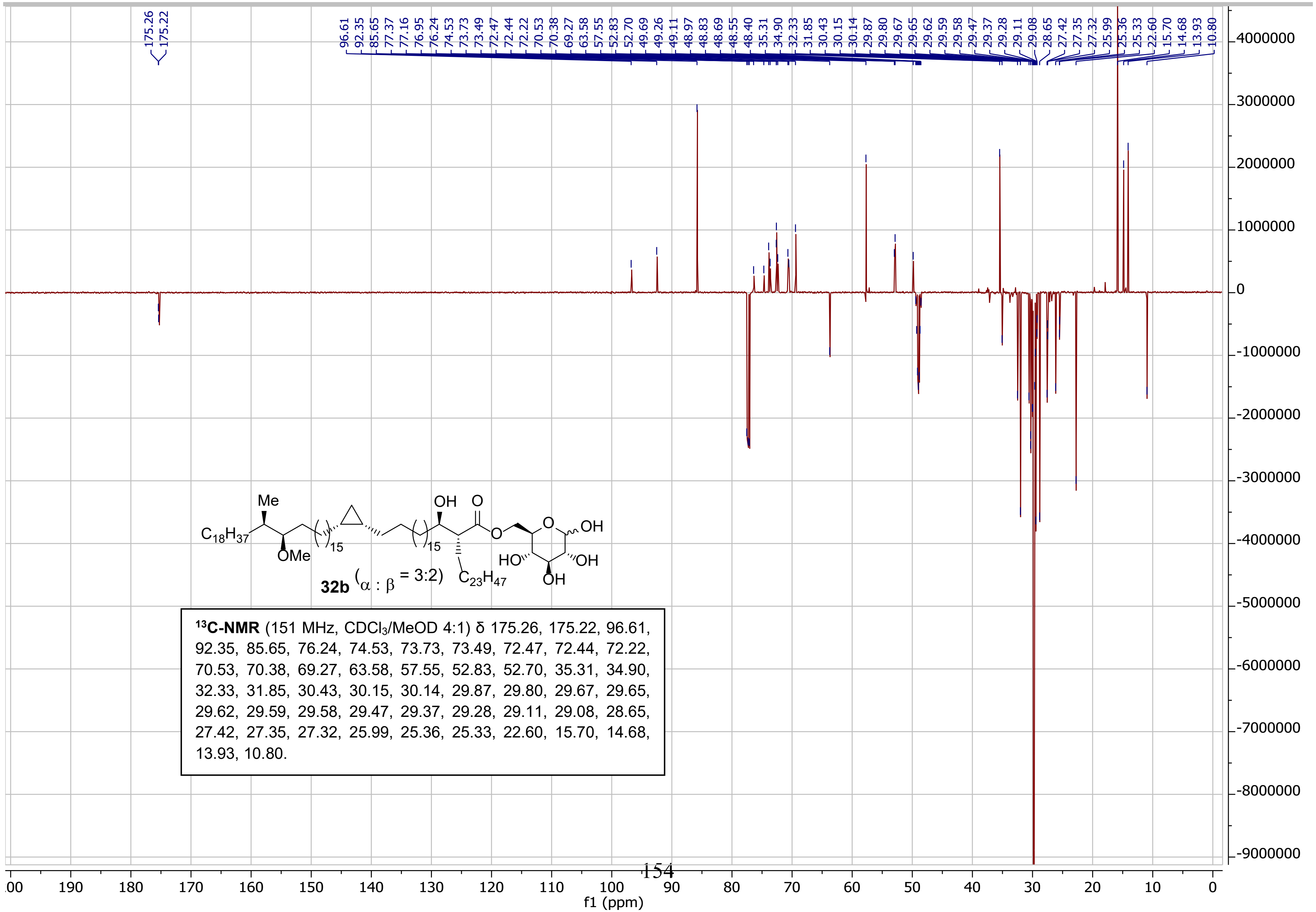

**32b** ($\alpha : \beta$ = 3:2)

**$^{13}$C-NMR** (151 MHz, $CDCl_3$/MeOD 4:1) δ 175.26, 175.22, 96.61, 92.35, 85.65, 76.24, 74.53, 73.73, 73.49, 72.47, 72.44, 72.22, 70.53, 70.38, 69.27, 63.58, 57.55, 52.83, 52.70, 35.31, 34.90, 32.33, 31.85, 30.43, 30.15, 30.14, 29.87, 29.80, 29.67, 29.65, 29.62, 29.59, 29.58, 29.47, 29.37, 29.28, 29.11, 29.08, 28.65, 27.42, 27.35, 27.32, 25.99, 25.36, 25.33, 22.60, 15.70, 14.68, 13.93, 10.80.

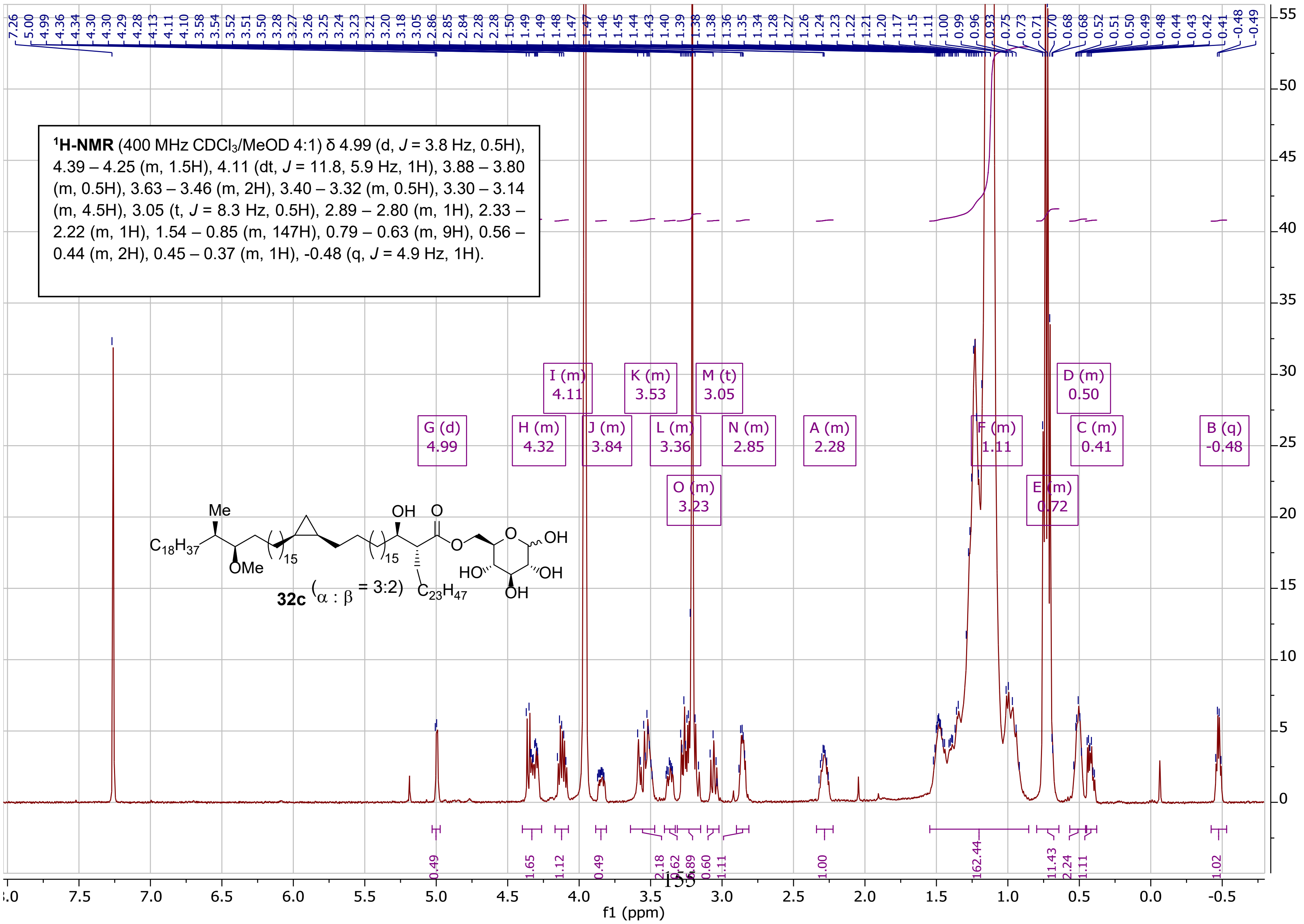
1H-NMR (400 MHz CDCl3/MeOD 4:1) δ 4.99 (d, J = 3.8 Hz, 0.5H), 4.39 – 4.25 (m, 1.5H), 4.11 (dt, J = 11.8, 5.9 Hz, 1H), 3.88 – 3.80 (m, 0.5H), 3.63 – 3.46 (m, 2H), 3.40 – 3.32 (m, 0.5H), 3.30 – 3.14 (m, 4.5H), 3.05 (t, J = 8.3 Hz, 0.5H), 2.89 – 2.80 (m, 1H), 2.33 – 2.22 (m, 1H), 1.54 – 0.85 (m, 147H), 0.79 – 0.63 (m, 9H), 0.56 – 0.44 (m, 2H), 0.45 – 0.37 (m, 1H), -0.48 (q, J = 4.9 Hz, 1H).
32c (α : β = 3:2)
f1 (ppm)

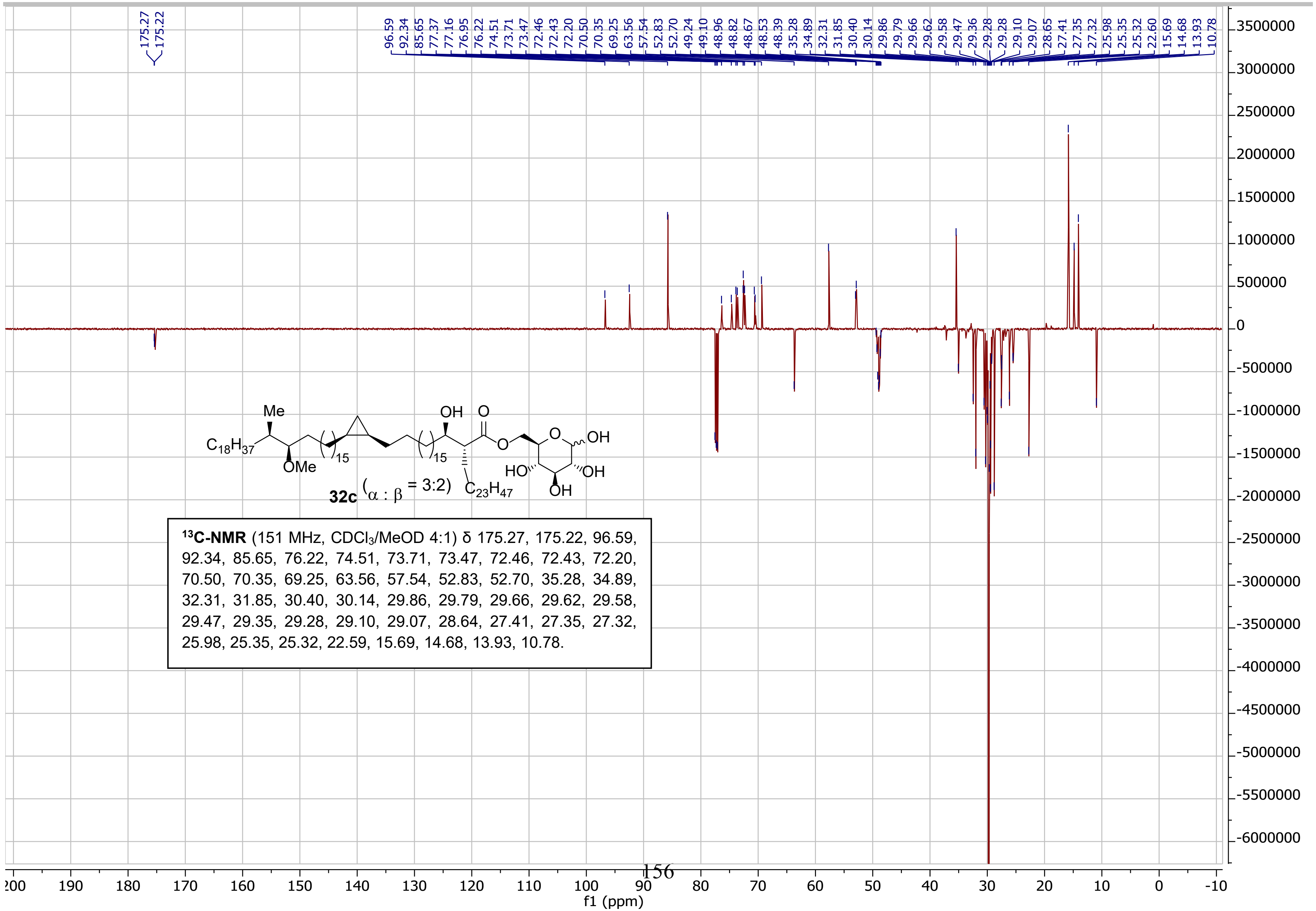

$^{13}$**C-NMR** (151 MHz, $CDCl_3$/MeOD 4:1) δ 175.27, 175.22, 96.59, 92.34, 85.65, 76.22, 74.51, 73.71, 73.47, 72.46, 72.43, 72.20, 70.50, 70.35, 69.25, 63.56, 57.54, 52.83, 52.70, 35.28, 34.89, 32.31, 31.85, 30.40, 30.14, 29.86, 29.79, 29.66, 29.62, 29.58, 29.47, 29.35, 29.28, 29.10, 29.07, 28.64, 27.41, 27.35, 27.32, 25.98, 25.35, 25.32, 22.59, 15.69, 14.68, 13.93, 10.78.

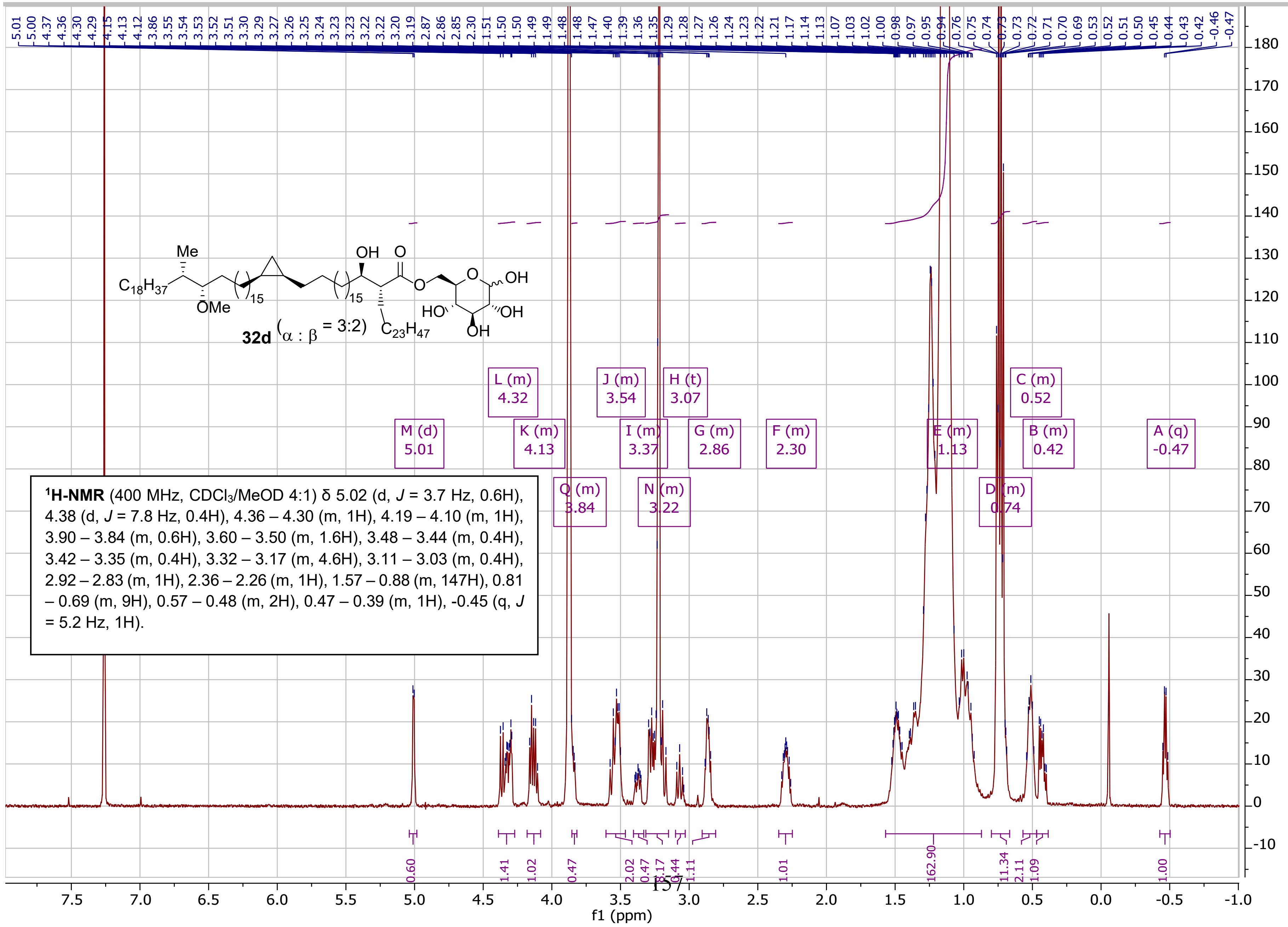


**$^1$H-NMR** (400 MHz, $CDCl_3$/MeOD 4:1) δ 5.02 (d, *J* = 3.7 Hz, 0.6H), 4.38 (d, *J* = 7.8 Hz, 0.4H), 4.36 – 4.30 (m, 1H), 4.19 – 4.10 (m, 1H), 3.90 – 3.84 (m, 0.6H), 3.60 – 3.50 (m, 1.6H), 3.48 – 3.44 (m, 0.4H), 3.42 – 3.35 (m, 0.4H), 3.32 – 3.17 (m, 4.6H), 3.11 – 3.03 (m, 0.4H), 2.92 – 2.83 (m, 1H), 2.36 – 2.26 (m, 1H), 1.57 – 0.88 (m, 147H), 0.81 – 0.69 (m, 9H), 0.57 – 0.48 (m, 2H), 0.47 – 0.39 (m, 1H), -0.45 (q, *J* = 5.2 Hz, 1H).

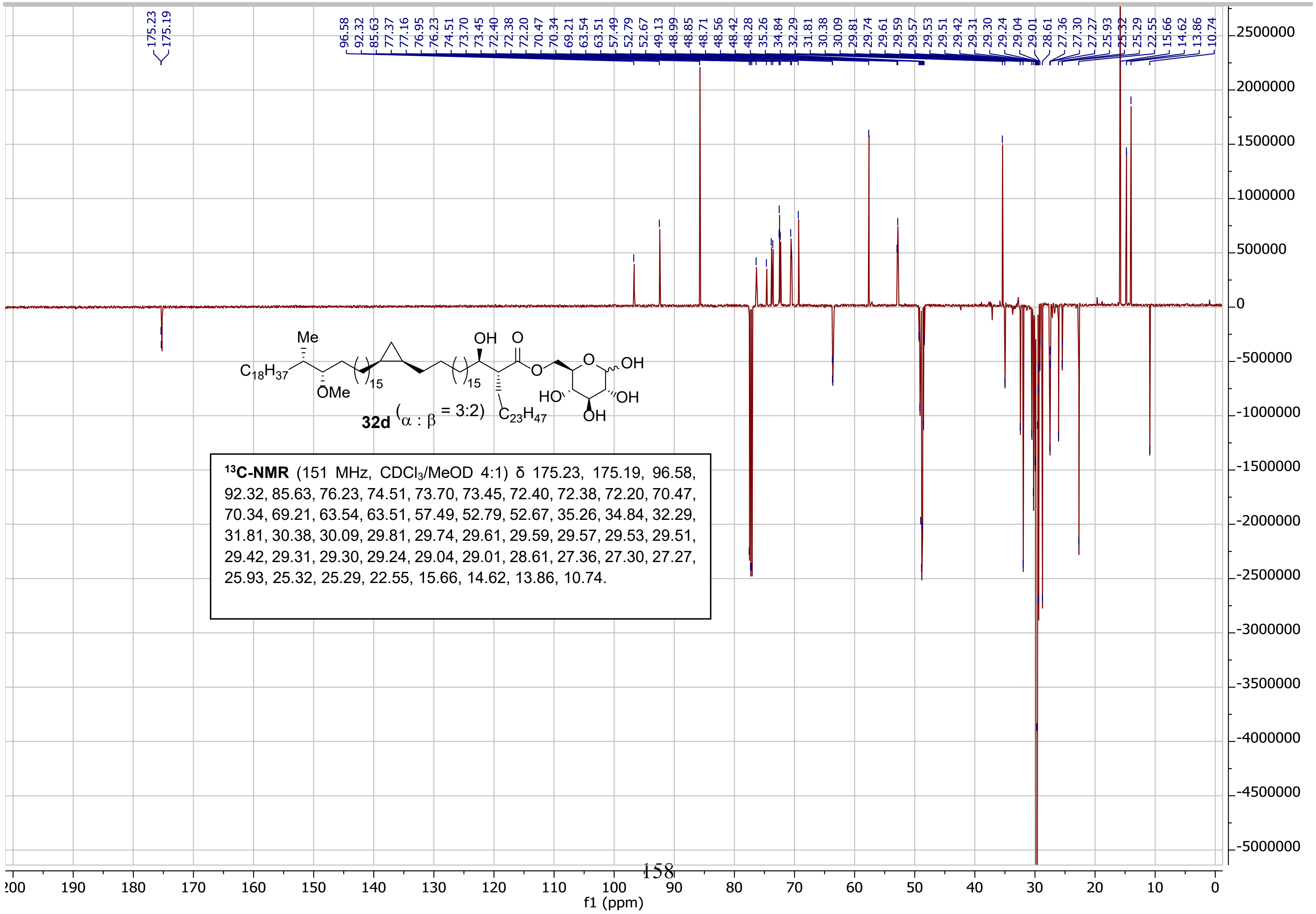


**$^{13}$C-NMR** (151 MHz, $CDCl_3$/MeOD 4:1) δ 175.23, 175.19, 96.58, 92.32, 85.63, 76.23, 74.51, 73.70, 73.45, 72.40, 72.38, 72.20, 70.47, 70.34, 69.21, 63.54, 63.51, 57.49, 52.79, 52.67, 35.26, 34.84, 32.29, 31.81, 30.38, 30.09, 29.81, 29.74, 29.61, 29.59, 29.57, 29.53, 29.51, 29.42, 29.31, 29.30, 29.24, 29.04, 29.01, 28.61, 27.36, 27.30, 27.27, 25.93, 25.32, 25.29, 22.55, 15.66, 14.62, 13.86, 10.74.

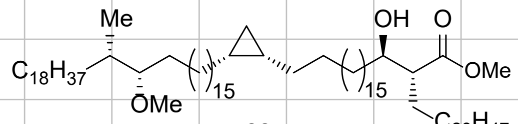

**33a**

**$^1$H-NMR** (400 MHz $CDCl_3$) δ 3.71 (s, 3H), 3.68 – 3.60 (m, 1H), 3.34 (s, 3H), 3.00 – 2.90 (m, 1H), 2.48 – 2.35 (m, 2H), 1.76 – 1.58 (m, 3H), 1.52 – 1.01 (m, 144H), 0.96 – 0.80 (m, 9H), 0.69 – 0.59 (m, 2H), 0.60 – 0.50 (m, 1H), -0.34 (q, *J* = 4.9 Hz, 1H)

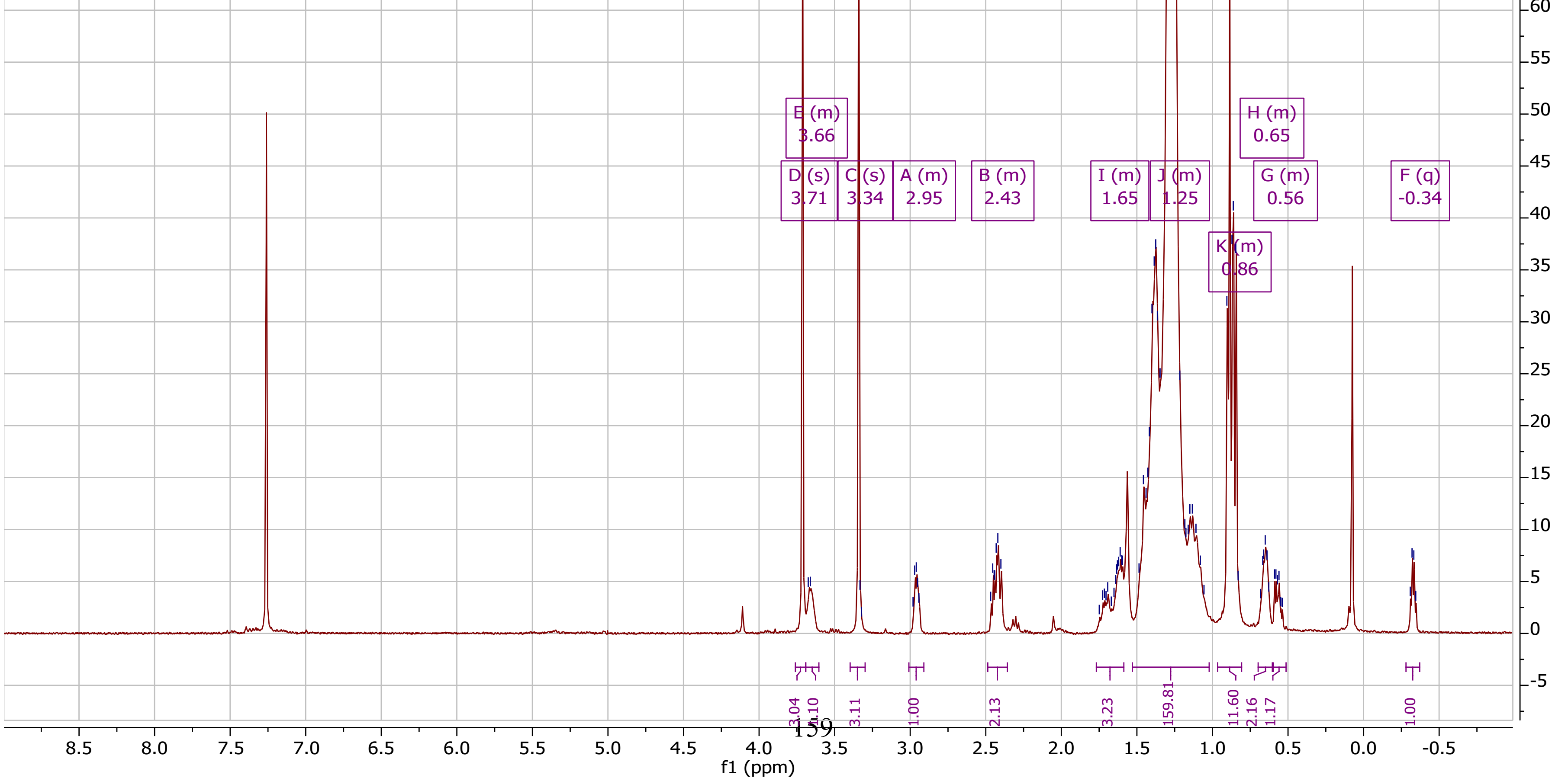

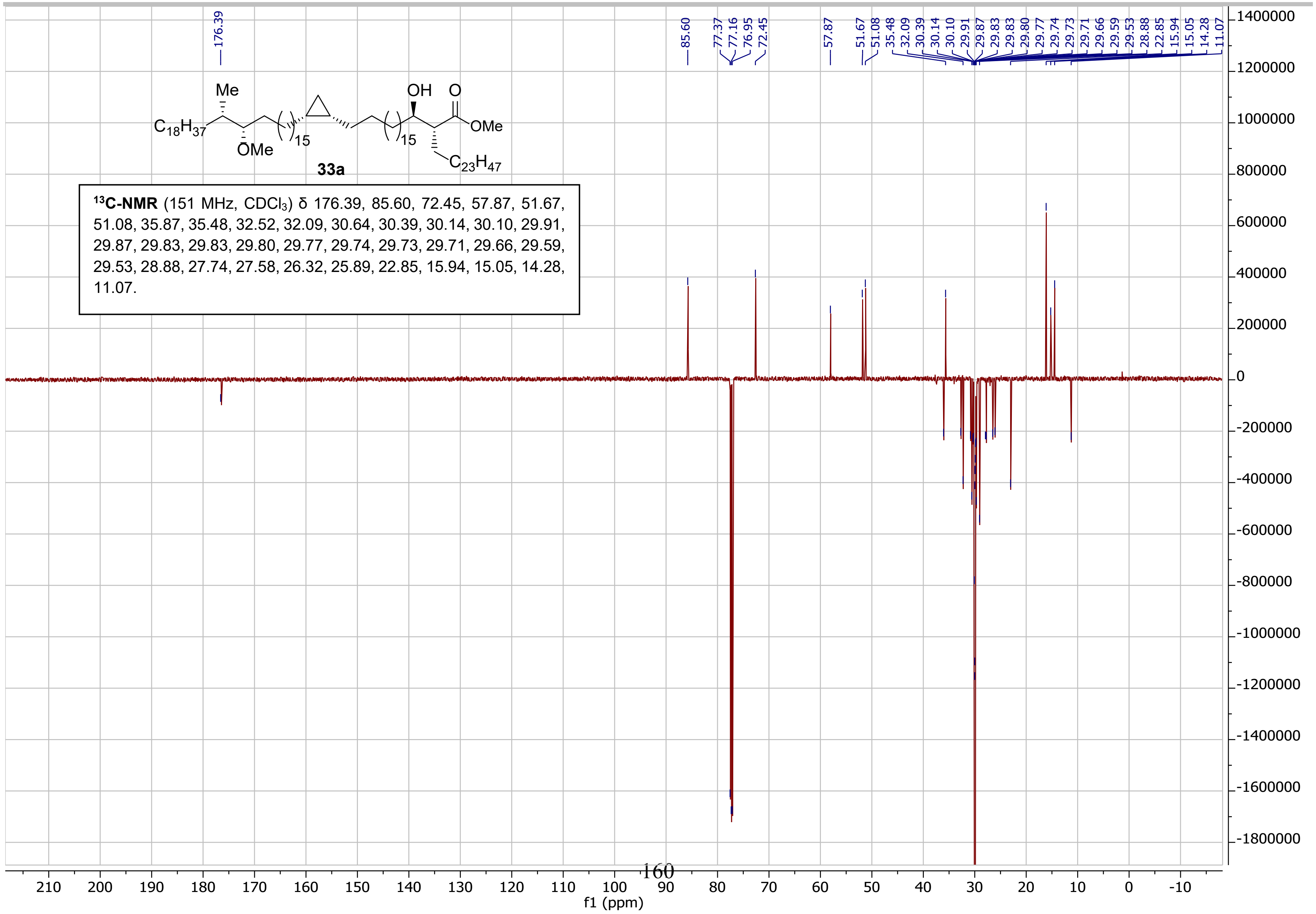


**$^{13}$C-NMR** (151 MHz, $CDCl_3$) δ 176.39, 85.60, 72.45, 57.87, 51.67, 51.08, 35.87, 35.48, 32.52, 32.09, 30.64, 30.39, 30.14, 30.10, 29.91, 29.87, 29.83, 29.83, 29.80, 29.77, 29.74, 29.73, 29.71, 29.66, 29.59, 29.53, 28.88, 27.74, 27.58, 26.32, 25.89, 22.85, 15.94, 15.05, 14.28, 11.07.

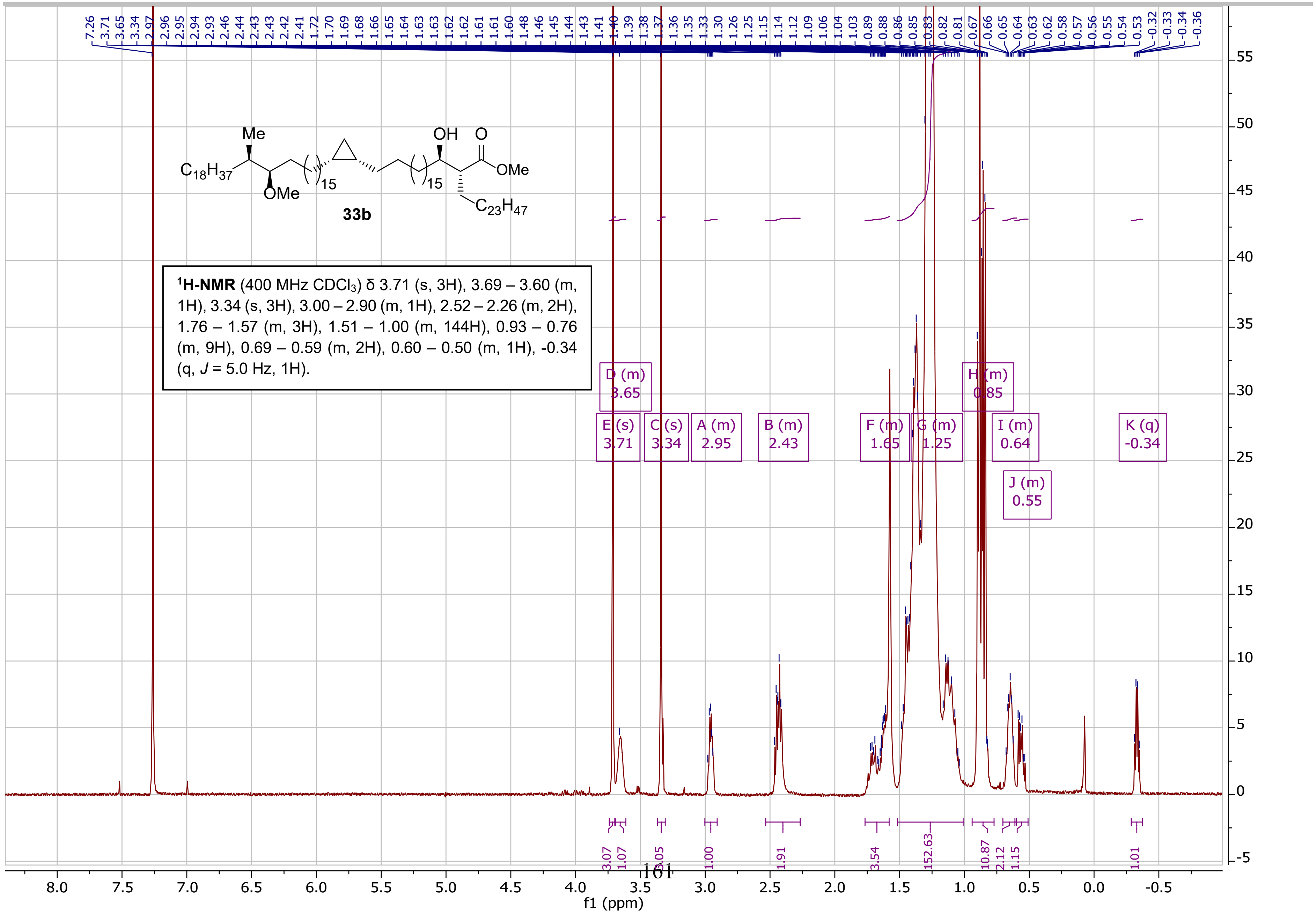
33b
¹H-NMR (400 MHz CDCl3) δ 3.71 (s, 3H), 3.69 – 3.60 (m, 1H), 3.34 (s, 3H), 3.00 – 2.90 (m, 1H), 2.52 – 2.26 (m, 2H), 1.76 – 1.57 (m, 3H), 1.51 – 1.00 (m, 144H), 0.93 – 0.76 (m, 9H), 0.69 – 0.59 (m, 2H), 0.60 – 0.50 (m, 1H), -0.34 (q, J = 5.0 Hz, 1H).
f1 (ppm)

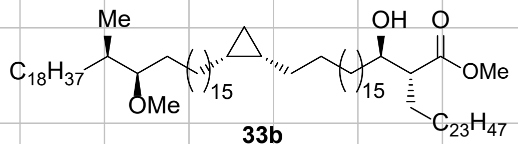


**33b**

$^{13}$**C-NMR** (151 MHz, $CDCl_3$) δ 176.39, 85.60, 77.37, 77.16, 76.95, 72.46, 57.87, 51.67, 51.09, 35.86, 35.48, 32.52, 32.09, 30.64, 30.39, 30.14, 30.10, 29.91, 29.86, 29.83, 29.82, 29.80, 29.79, 29.77, 29.74, 29.73, 29.71, 29.66, 29.59, 29.52, 28.88, 27.74, 27.58, 26.32, 25.89, 22.85, 15.93, 15.05, 14.28, 11.07.

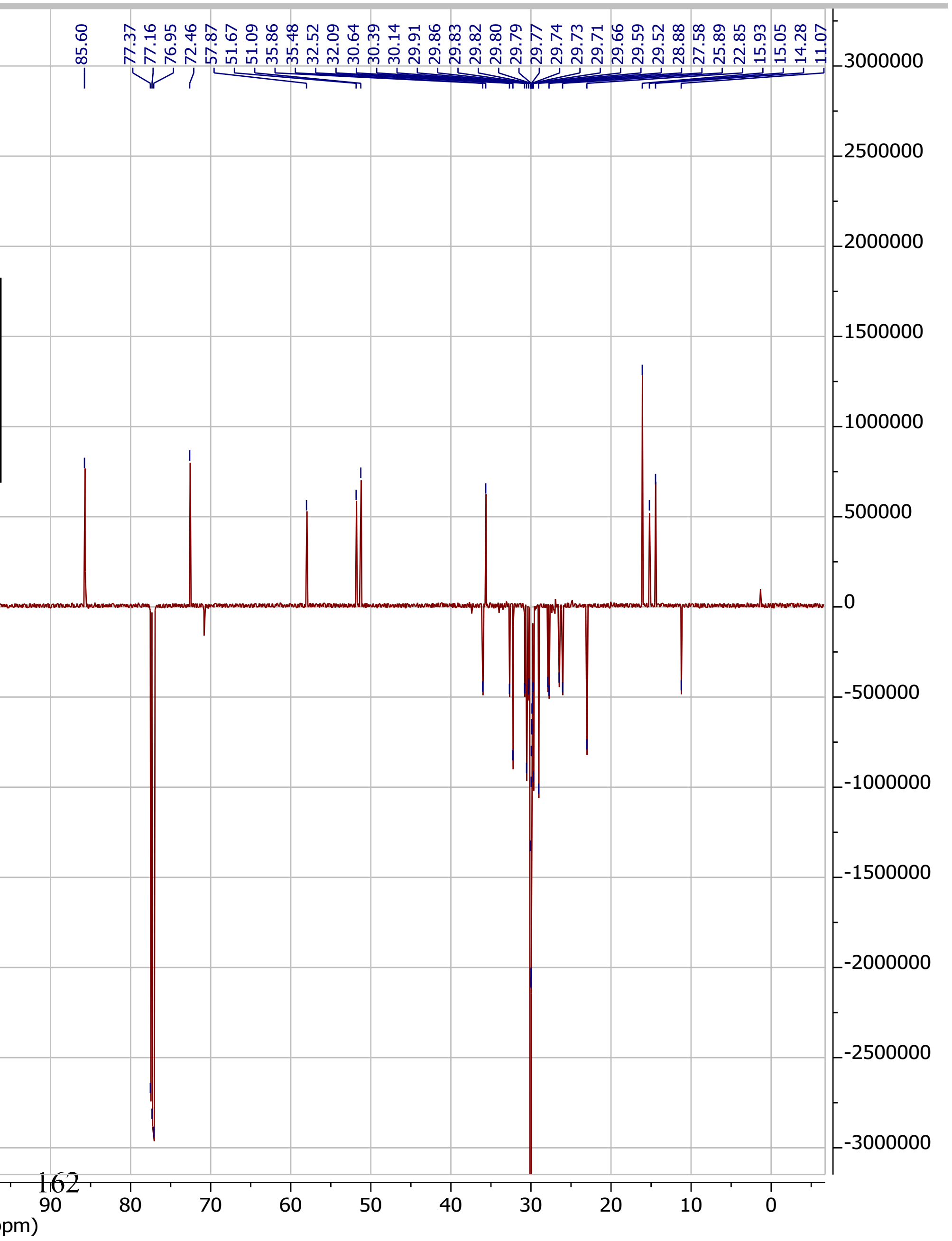

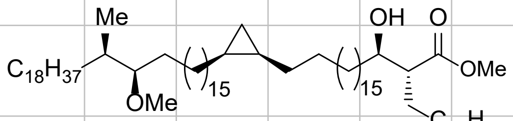

**33c**

**$^{1}$H-NMR** (400 MHz $CDCl_3$) δ 3.71 (s, 3H), 3.69 – 3.60 (m, 1H), 3.34 (s, 3H), 3.00 – 2.90 (m, 1H), 2.52 – 2.26 (m, 2H), 1.76 – 1.57 (m, 3H), 1.51 – 1.00 (m, 144H), 0.93 – 0.76 (m, 9H), 0.69 – 0.59 (m, 2H), 0.60 – 0.50 (m, 1H), -0.34 (q, *J* = 5.0 Hz, 1H).

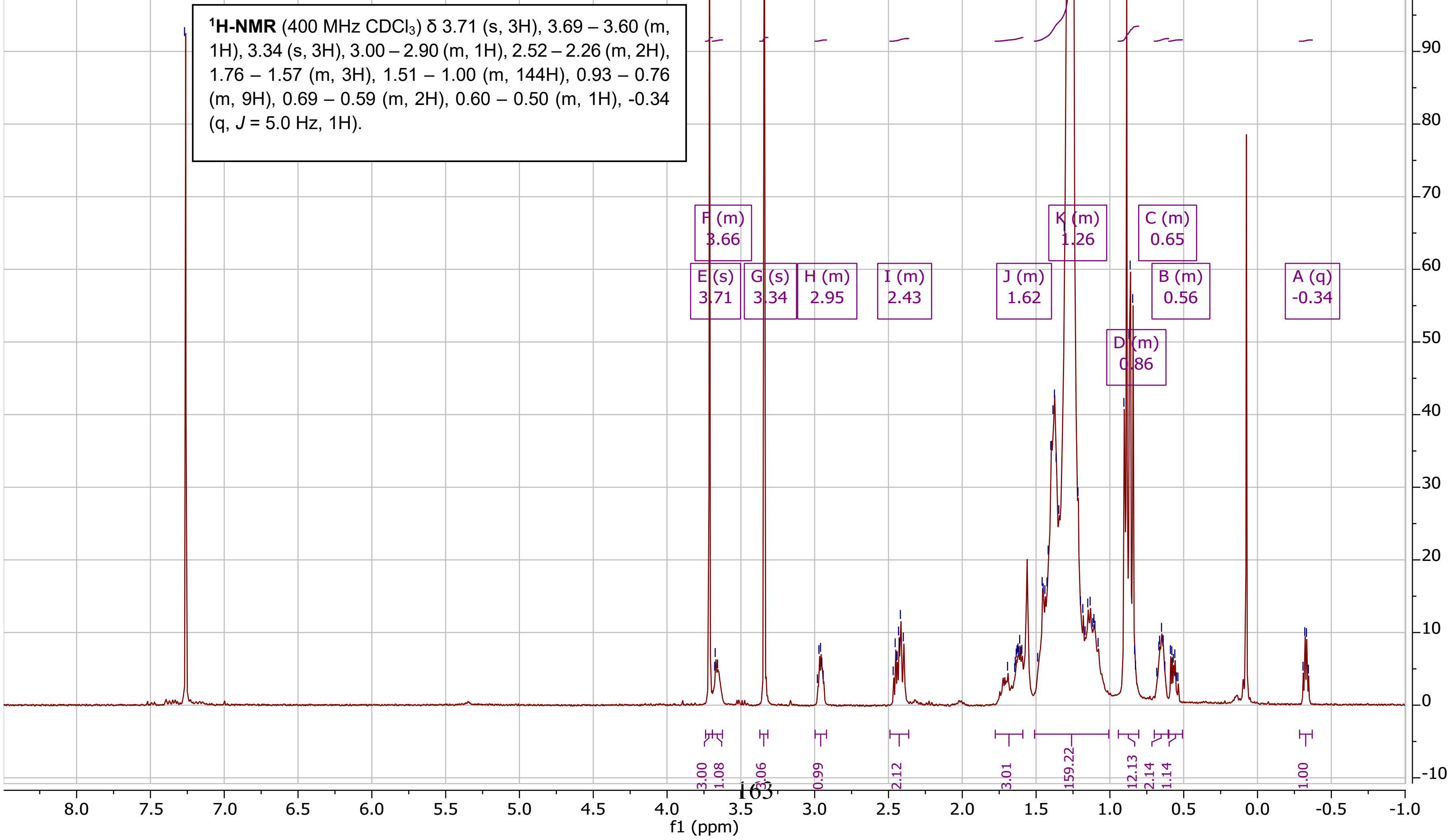

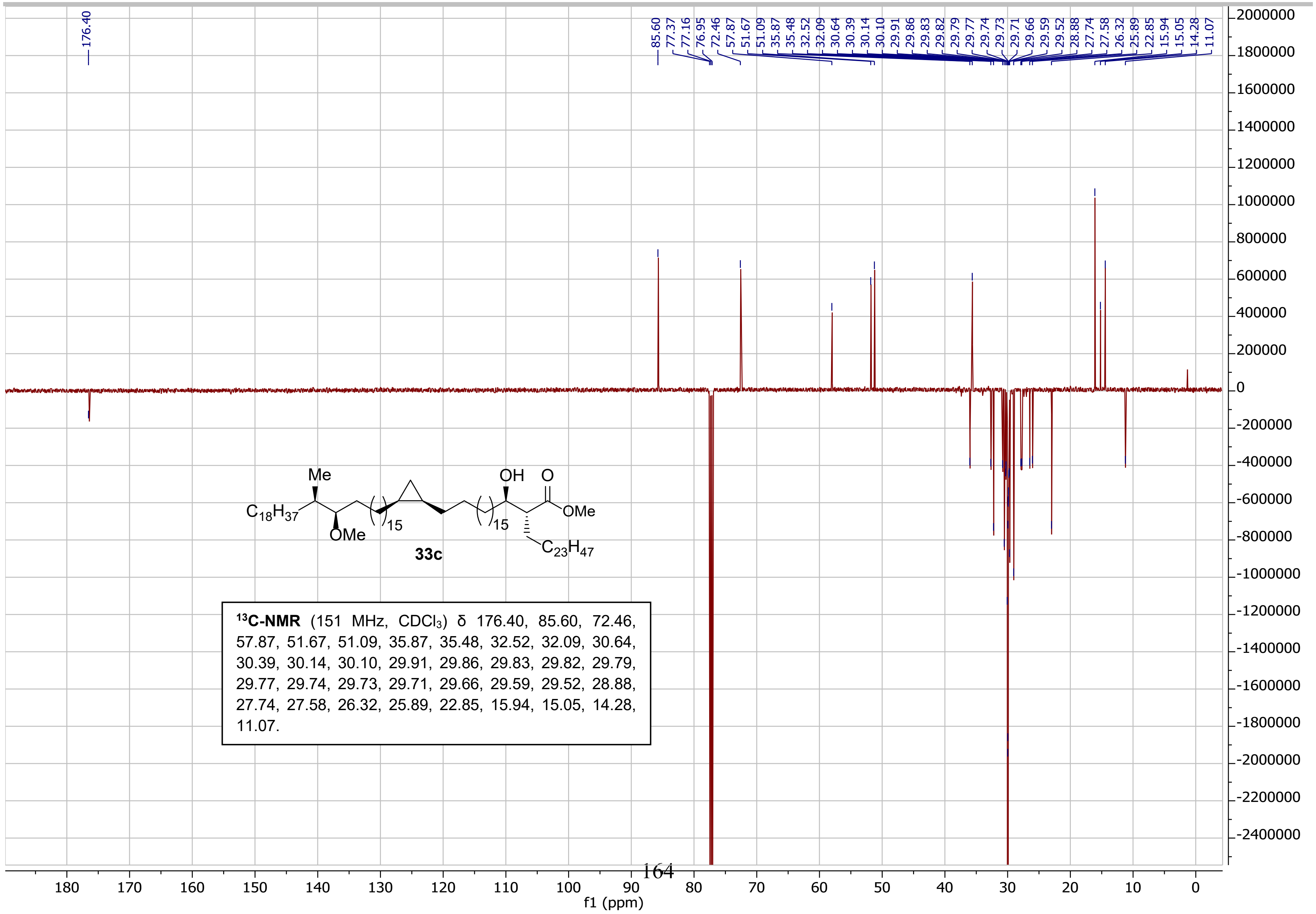


$^{13}$**C-NMR** (151 MHz, $CDCl_3$) δ 176.40, 85.60, 72.46, 57.87, 51.67, 51.09, 35.87, 35.48, 32.52, 32.09, 30.64, 30.39, 30.14, 30.10, 29.91, 29.86, 29.83, 29.82, 29.79, 29.77, 29.74, 29.73, 29.71, 29.66, 29.59, 29.52, 28.88, 27.74, 27.58, 26.32, 25.89, 22.85, 15.94, 15.05, 14.28, 11.07.

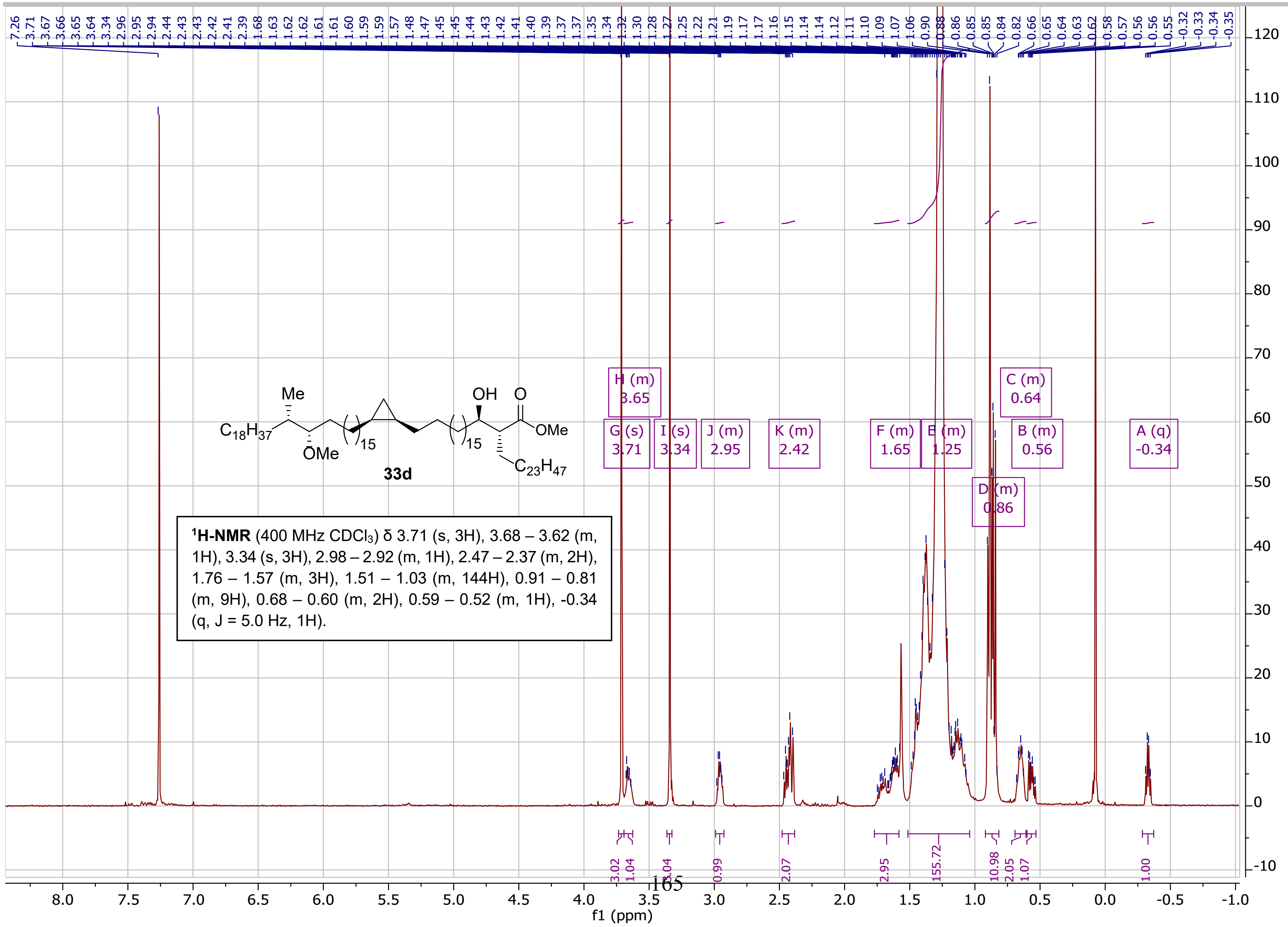


$^{1}$H-NMR (400 MHz $CDCl_3$) δ 3.71 (s, 3H), 3.68 – 3.62 (m, 1H), 3.34 (s, 3H), 2.98 – 2.92 (m, 1H), 2.47 – 2.37 (m, 2H), 1.76 – 1.57 (m, 3H), 1.51 – 1.03 (m, 144H), 0.91 – 0.81 (m, 9H), 0.68 – 0.60 (m, 2H), 0.59 – 0.52 (m, 1H), -0.34 (q, J = 5.0 Hz, 1H).

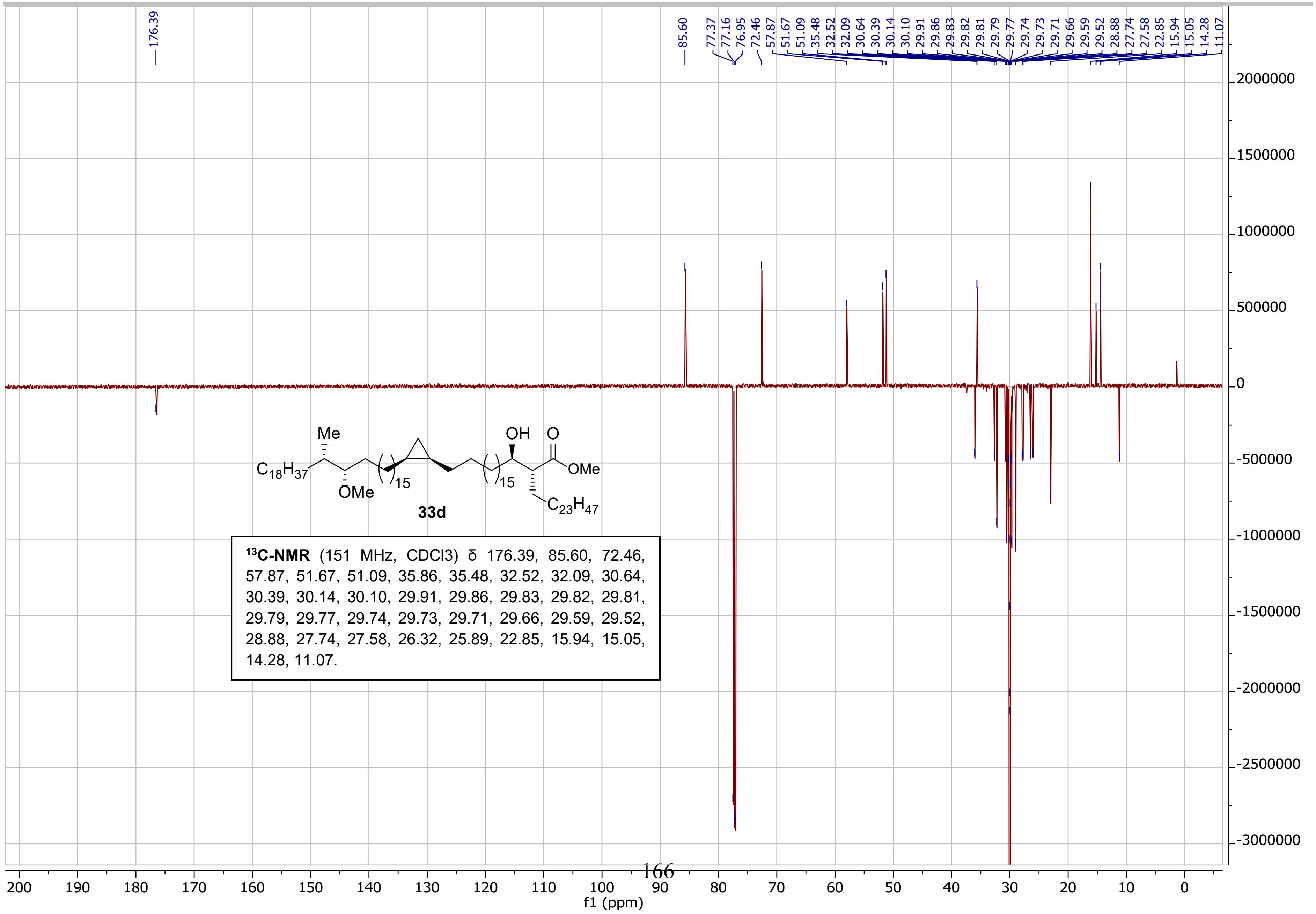


**$^{13}$C-NMR** (151 MHz, CDCl3) δ 176.39, 85.60, 72.46, 57.87, 51.67, 51.09, 35.86, 35.48, 32.52, 32.09, 30.64, 30.39, 30.14, 30.10, 29.91, 29.86, 29.83, 29.82, 29.81, 29.79, 29.77, 29.74, 29.73, 29.71, 29.66, 29.59, 29.52, 28.88, 27.74, 27.58, 26.32, 25.89, 22.85, 15.94, 15.05, 14.28, 11.07.
